# Access to Axially Chiral Aryl Aldehydes via Carbene-Catalyzed Nitrile Formation and Desymmetrization Reaction

**DOI:** 10.34133/research.0293

**Published:** 2024-01-16

**Authors:** Yuanlin Cai, Ya Lv, Liangzhen Shu, Zhichao Jin, Yonggui Robin Chi, Tingting Li

**Affiliations:** ^1^ National Key Laboratory of Green Pesticide, Key Laboratory of Green Pesticide and Agricultural Bioengineering, Ministry of Education, Guizhou University, Guiyang 550025, China.; ^2^ School of Chemistry, Chemical Engineering, and Biotechnology, Nanyang Technological University, Singapore 637371, Singapore.

## Abstract

An approach utilizing N-heterocyclic carbene for nitrile formation and desymmetrization reaction is developed. The process involves kinetic resolution, with the axially chiral aryl monoaldehydes obtained in moderate yields with excellent optical purities. These axially chiral aryl monoaldehydes can be conveniently transformed into functionalized molecules, showing great potential as catalysts in organic chemistry.

## Introduction

**Fig. 1.**
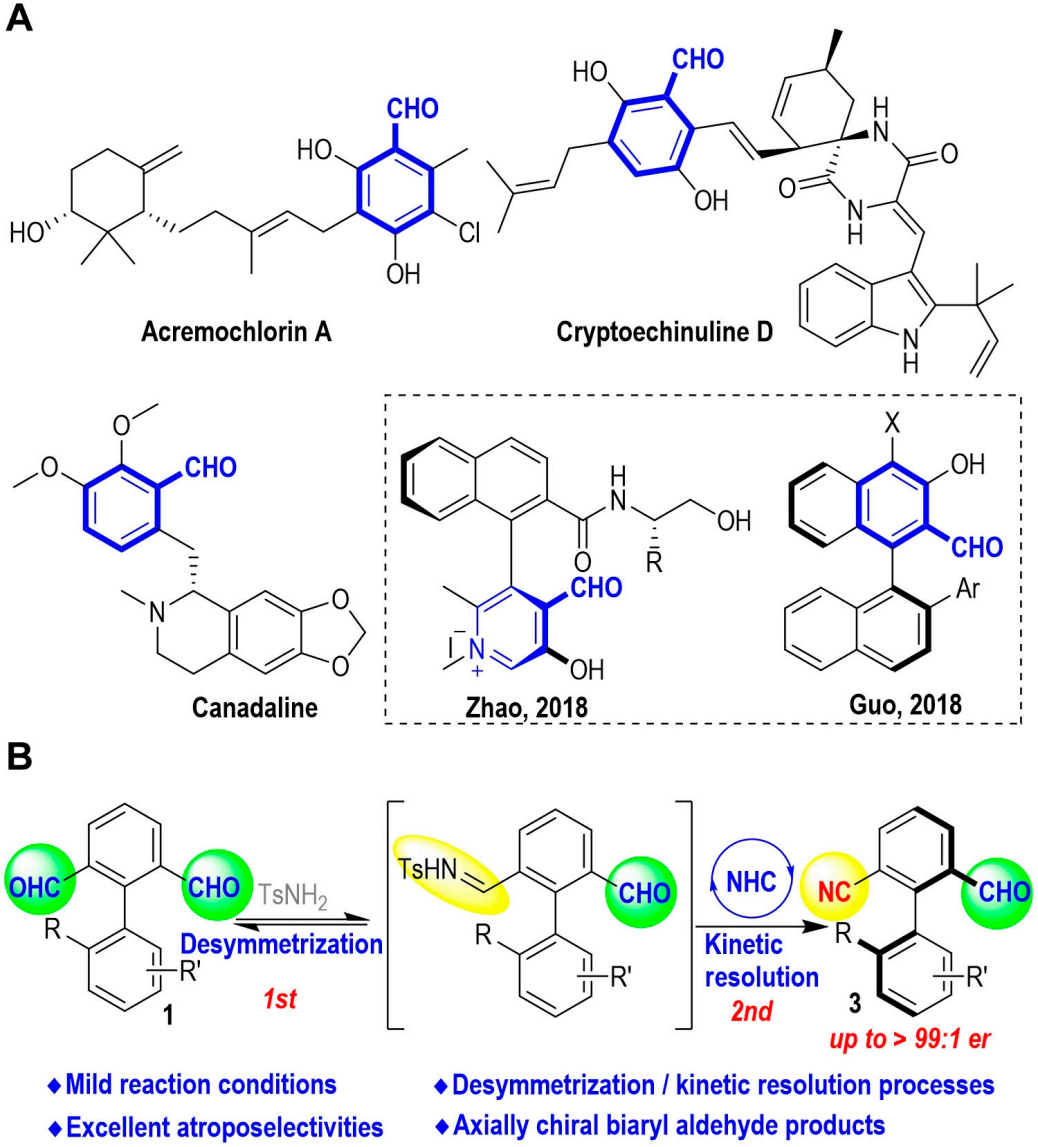
Bioactive molecules and catalysts containing aryl aldehyde moiety and NHC-catalyzed atropoenantioselective nitrile formation and desymmetrization reaction. (A) Bioactive molecules and catalysts containing aryl aldehyde moieties. (B) This work: atroposelective synthesis of axially chiral biaryl aldehydes.

Aryl aldehydes bearing chiral axis have received increasing attention in recent years [1–5]. In particular, these molecules have been found to be excellent chiral catalysts for enantioselective reactions, as demonstrated by Zhao et al. [6–10], Guo et al. [11–14], and others [15,16]. In a broader picture, aldehyde moieties have versatile applications in chemical synthesis [17–21] and exhibit various functions in natural products and bioactive molecules [22–25] (Fig. 1A). A number of synthetic approaches are now available to prepare axially chiral aryl aldehydes via enzymatic catalysis [26], transition metal catalysis [17,19–21,27–29], or organic catalysis [18,30,31]. Elegant examples include metal-catalyzed C-H activation or cross-coupling reactions, as demonstrated by Liao et al. [27], Chen et al. [17], Yang et al. [20], and others [19,21,28,29]. In the area of organic catalysis, Sparr et al. [18,30] reported an innovative approach to prepare axially chiral aryl aldehydes via an amine-catalyzed cascade reaction to set up new aryl cores and the chiral axis. Wu et al. [32] disclosed N-heterocyclic carbene (NHC)-catalyzed aldehyde-to-ester transformation with formal desymmetrization to prepare axially chiral aryl aldehyde.

We are interested in employing NHC organic catalysts for unconventional transformations and functional molecules [33–46]. Research from Chen et al. [47], our laboratory [48], and Sun et al. [49] have shown that under the influence of NHC catalysts, aldehydes can be converted to nitriles (via the corresponding imine intermediates) under mild conditions. Fier and Maloney [50] developed this nitrile formation process as a protocol for deamination of primary sulfonamides. We recently converted monoaldehydes to the corresponding nitriles with axial chirality controlled [51].

Here, we disclose that through NHC-catalyzed nitrile formation, a desymmetrization/dynamic kinetic resolution process can be achieved and transfers symmetric dicarbaldehydes to axially chiral aryl monoaldehydes (Fig. 1B). Products from our approach containing cyanide and aldehyde groups are potential catalysts in organocatalysis. In contrast to earlier more conventional aldehyde conversions (such as ester formation), we hope our study encourages further exploration in innovating the NHC-catalyzed aldehyde-to-nitrile process for new applications.

## Results

We initially employed biaryl dialdehyde (**1a**) and readily available *p*-toluenesulfinate (TsNH_2_, **2**) as model substrates to screen reaction conditions for this transformation (Table). Subsequently, we performed a screening of various indanol-derived NHC pre-catalysts in the presence of NHEt_2_ in dichloroethane (DCE) solvent (Table, entries 1 to 3). Notably, the utilization of NHC pre-catalyst **A** [52] featuring the N-Mes group in this process could only generate a trace amount of the axially chiral aryl monoaldehyde **3a** (entry 1). However, the product **3a** could be given in moderate yields while utilizing NHC pre-catalysts **B** and **C** [53,54] (entries 2 and 3). Additionally, we observed the formation of a by-product biaryl dicarbonitrile **4** during this transformation process. Considering the impact of bases on nitrile formation during NHC catalysis, we conducted additional screening of various bases. However, when NHC pre-catalyst **B** was employed, the use of alternative bases resulted in a notable decrease on the product enantiomeric ratio (er) values (entries 4 to 7). Regrettably, our attempts to improve the results by screening various nonpolar and polar organic solvents did not yield positive outcomes (entries 8 to 11). However, it is worth noting that increasing the quantity of substrate **2** (TsNH_2_) resulted in a slight increase in yields and importantly improved the er values of product **3a**. Moreover, in these cases, a nonchiral dicarbonitrile by-product **4** was obtained, with yields ranging from 18% to 21% (entries 12 and 13). Finally, when a 4-Å molecular sieve (MS) was used as the additive in the 1.0 mL of DCE, the target axially chiral product **3a** was obtained in 63% yield and >99:1 er value (entry 14).

**Table.** Optimized conditions. Unless otherwise specified, the reactions were carried out using 1a (0.12 mmol), 2 (0.10 mmol), NHC (0.02 mmol), base (0.12 mmol), 4 Å MS (50 mg), and solvent (2.0 ml) at 40 °C under N_2_ for 24 h.

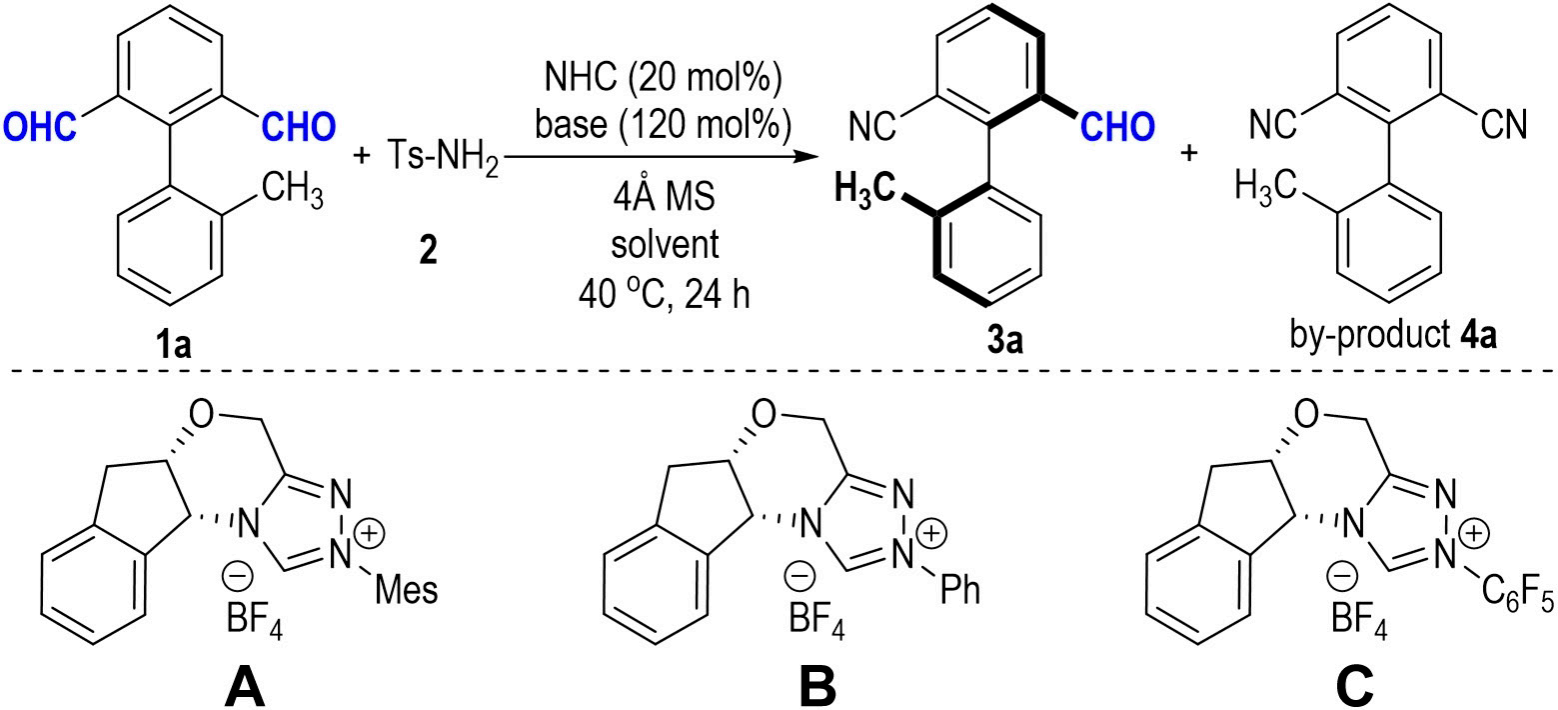
Entry	NHC	Base	Solvent	**3a** [%]^a^	Er^b^	**4a** [%]
1	**A**	NHEt_2_	DCE	Trace	-	-
2	**B**	NHEt_2_	DCE	50	96:4	16
3	**C**	NHEt_2_	DCE	45	65:35	22
4	**B**	Cs_2_CO_3_	DCE	43	63:37	15
5	**B**	NaHCO_3_	DCE	Trace	-	-
6	**B**	DBU	DCE	44	79:21	19
7	**B**	NEt_3_	DCE	55	82:18	21
8	**B**	NHEt_2_	DCM	48	93:7	16
9	**B**	NHEt_2_	PhCH_3_	42	90:10	16
10	**B**	NHEt_2_	CHCl_3_	56	92:8	15
11	**B**	NHEt_2_	THF	40	93:7	11
12^c^	**B**	NHEt_2_	DCE	54	98:2	18
13^d^	**B**	NHEt_2_	DCE	57	99:1	21
14^d,e^	**B**	NHEt_2_	DCE	63	>99:1	15

DCE, 1,2-dichloroethane; DCM, dichloromethane.

^a^ Isolated yield of **3a.** ^b^ The er values of **3a** were determined via HPLC on chiral stationary phase. ^c^ **1a** (0.10 mmol), **2** (0.20 mmol). ^d^ **1a** (0.10 mmol), **2** (0.30 mmol). ^e^ **1a** (0.10 mmol), **2** (0.30 mmol), 4 Å MS (50 mg), and solvent (1.0 ml).

**Fig. 2.**
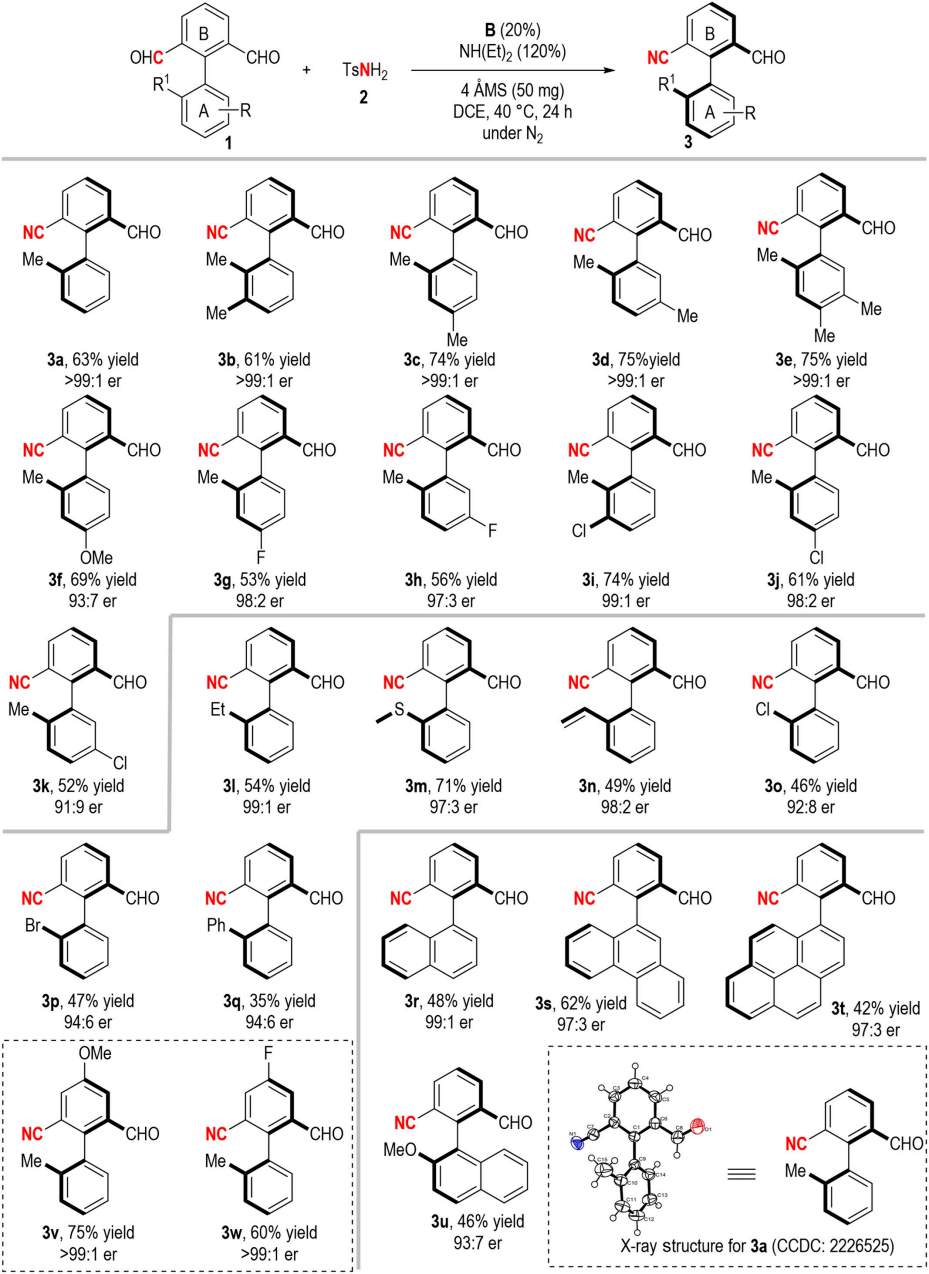
Scope of dialdehydes 1.

When the optimized reaction condition was obtained (Table, entry 14), we further explored the substrate scope of the atropoenantioselective nitrile formation process using a variety of biaryl dialdehydes **1** (Fig. 2). Whether one or two methyl groups are installed in ring A, with the corresponding aryl monoaldehydes obtained in improved yields with no erosion of the product er values (**3b** to **3e**). However, the electron-donating group 4′-OCH_3_ introduced in ring A led to a lower enantioselectivity (**3f**). The halogen (F, Cl) could be attached to ring A of the benzaldehydes **1** to give corresponding products in moderate yields with retaining high optical purities (**3g** to **3j**). Notably, the presence of 5′-Cl of ring A in the biaryl dialdehyde resulted in a lower er value of aryl aldehyde product **3k**, albeit with a moderate yield. The 2′-methyl group on ring A played an important role in restricting axial rotation. For instance, when ethyl, -SMe, or alkenyl groups were incorporated at the 2′-position of ring A, the product yields of the aryl monoaldehydes were slightly changed (**3l** to **3n**). Moreover, an obvious reduction in both product yields and er values could be observed when the 2′-halogen or 2′-phenyl group was introduced to ring A (**3o** to **3q**). Furthermore, the NHC-catalyzed atropoenantioselective reaction exhibited promising performance even in the presence of aromatic fused rings in the dialdehydes, and all the corresponding aryl monoaldehydes could be obtained with excellent optical purities (**3r** to **3t**). However, a slight decrease in the product er value occurred when 2′-CH_3_O-naphthyl was used to replace ring A (**3u**). In addition, we also tested substrate scope on the dialdehyde aromatic ring (ring B). Electron-donating (OMe) and halogen (F) groups were tolerated well and given corresponding aryl monoaldehyde products **3v** and **3w** in moderate yields and excellent er values.

**Fig. 3.**
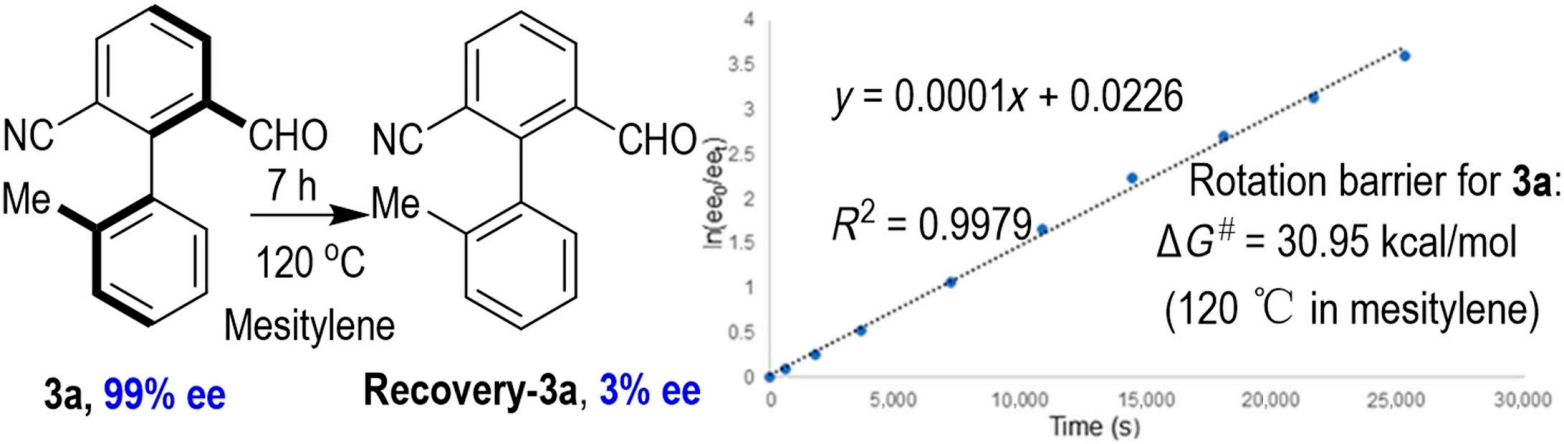
Evaluation of the stereochemical stability of 3a.

The stereochemical stability of the axially chiral biaryl aldehyde product **3a** was then evaluated through the previous experimental approach [55–57] (Fig. 3). The procedure involved stirring the compound in mesitylene at 120 °C for 7 h. Consequently, a nearly racemic mixture of the targeted product **3a** was obtained. By analyzing the rate of change in product enantiomeric excess (ee) values at 120 °C, it was possible to ascertain the rotational barrier to the axially chiral compound **3a**. On the basis of the changing rate of product ee values at 120 °C, the rotational barrier of the axially chiral compound **3a** was determined (Δ*G*^#^ = 30.95 kcal/mol). Additionally, based on this barrier energy, a half-life to racemization at 120 °C was calculated and the half-life of product **3a** is 1.92 h.

**Fig. 4.**
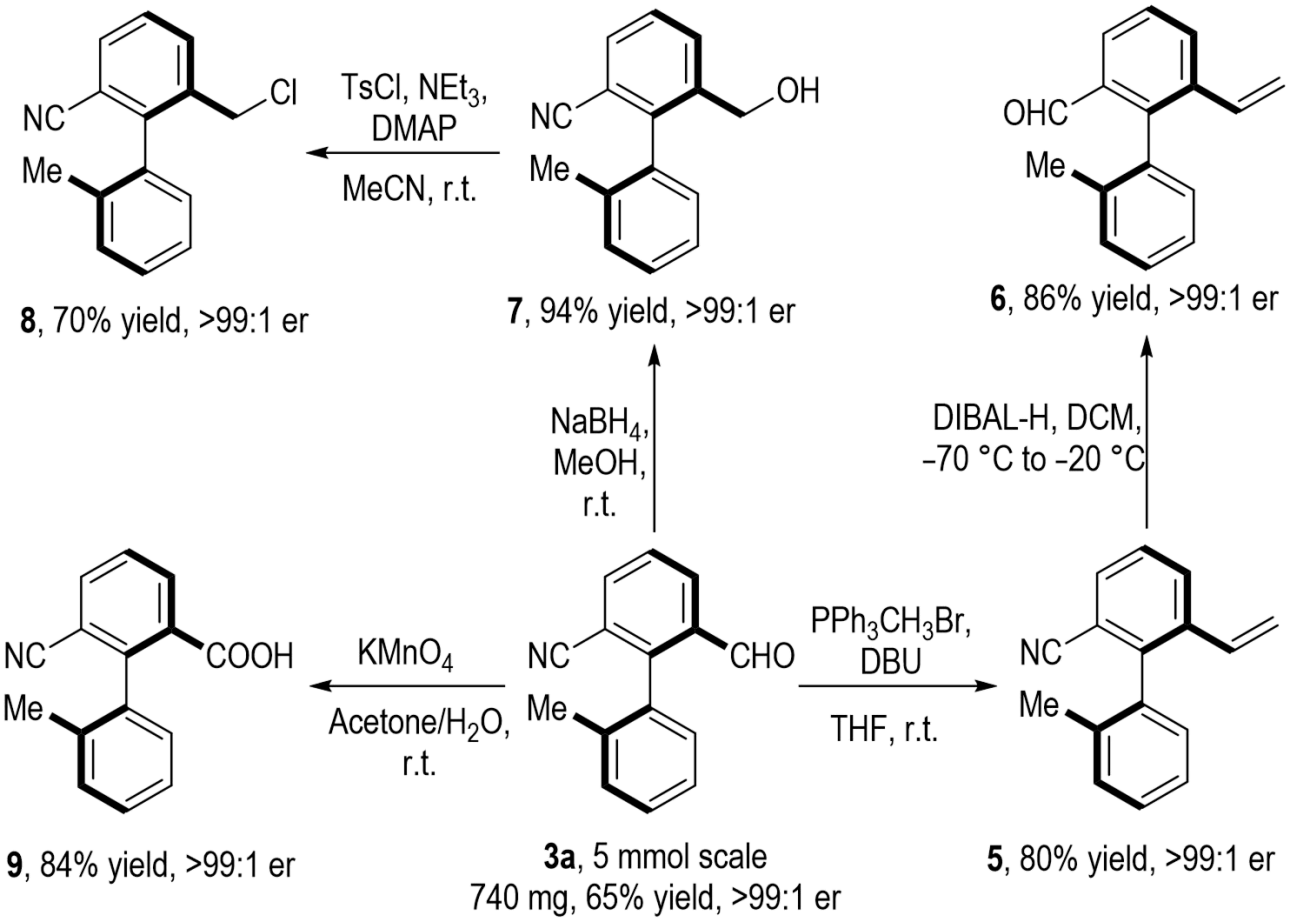
Large-scale synthesis and synthetic transformations of 3a.

It is worth noting that this transformation can be put into practice at 5-mmol scales, resulting in the aryl aldehyde product **3a** in 65% yield and >99:1 er value. The enantiomerically enriched axially chiral product **3a**, containing both aldehyde and nitrile units, can serve as a potential catalyst in the aldehyde-catalyzed asymmetric reaction and could also be easily derived into a variety of functionalized molecules through simple protocols (Fig. 4). Aryl aldehyde product **3a** could react with Wittig reagent to generate the axially chiral alkene product **5**. Subsequently, the CN group of compound **5** can be reduced to an aldehyde group in 86% yield and >99:1 er value. Moreover, the optically enriched **3a** could be efficiently reduced by NaBH_4_ to give alcohol **7** in quantitative yield with no erosion of optical purities. Then, the OH group of **7** can be substituted by the chloro group to form an unexpected product **8** in 70% yield with excellent enantioselectivity in this transformation. Furthermore, in the presence of potassium permanganate, compound **3a** undergoes oxidation process to give axially chiral carboxylic acid **9** in good yield while maintaining its excellent optical purity. This axially chiral carboxylic acid **9** can be effectively employed as an organic catalyst for acid-catalyzed asymmetric reactions [58–64].

**Fig. 5.**
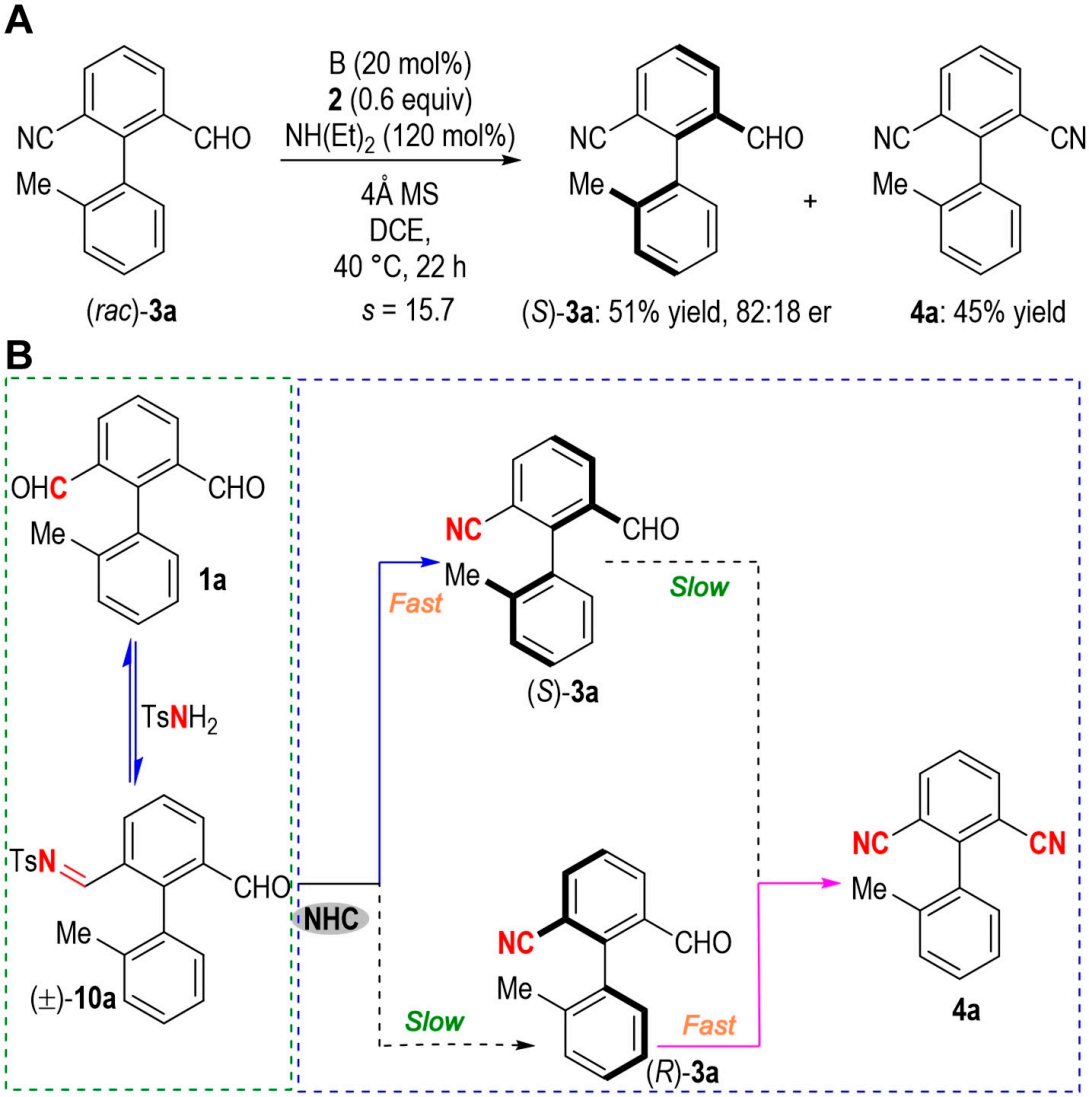
Mechanistic study. (A) Control experiment. (B) Proposed reaction mechanism.

It is worth noting that the formation of the intermediate (±)-**10a** is a reversible process from TsNH_2_ with aldehydes [65]. The formation of by-product **4a** could affect the target product er values in this process (Table, entries 2, 12, and 13). Subsequently, we performed a control experiment using (*rac*)-**3a** as the starting material under standard conditions. In this control experiment (Fig. 5A), (*S*)-**3a** was obtained with a yield of 51% and an 82:18 er value. It is worth noting that by-product **4a** was also generated in 45% yield. This result demonstrated that the kinetic resolution is critical for the product optical purity. Based on the experimental observation together with the reported literature [32,66–71], a proposed reaction pathway is depicted in Fig. 5B. First of all, the condensation of biaryl dialdehyde **1a** with TsNH_2_ is generated to afford a racemic mixture of imines (±)-**10a**, then (*S*)-**10a** undergoes a faster desulfonylation reaction to give the corresponding atropisomeric benzaldehyde product (*S*)-**3a** in excellent enantioselectivity enabled by a chiral NHC catalyst. On the other hand, (*R*)-**10a** was dynamically hydrolyzed at a slower reaction rate than the starting material **1a**. This process is desymmetrization/dynamic kinetic resolution, leading to the formation of the by-product **4a** and improved enantioselectivity of product **3a**, accompanied by the consumption of (*R*)-**3a**.

## Discussion

We have developed an atropoenantioselective NHC-catalyzed nitrile formation and desymmetrization reaction. Various substituents can be tolerated well, with the corresponding aryl monoaldehydes afforded in moderate to good yields and good to excellent er values. The stereochemical stability of aryl aldehyde has been assessed using thermal dynamic methods. Notably, this synthetic protocol is well-suited for large-scale synthesis, and the resulting products, containing both aldehyde and nitrile functional groups, can be conveniently transformed into diverse functional molecules without marked loss of optical purities. As we continue our research, we aim to explore further synthetic applications for these atropoisomerically enriched aryl monoaldehydes.

## Methods

In a glove box, dicarbaldehyde **1** (0.10 mmol), NHC pre-catalyst **B** (0.02 mmol, 7.5 mg), TsNH_2_ **2** (0.30 mmol), and 4 Å MS (50 mg) were added into a 4.0-ml vial containing a magnetic stir bar. Subsequently, anhydrous DCE (1.0 ml) and *N,N*-diethylamine (NHEt_2_) (0.12 mmol, 12.4 μl) were added into the vial using a syringe. The resulting reaction mixture was stirred at 40 °C for 24 h. The mixture was directly purified by column chromatography (silica gel, eluent: petroleum ether/ethyl acetate = 20:1) to obtain the target product **3**.

## Data Availability

The data that support the findings of this study are available from the corresponding author upon reasonable request.
