# Supplementary material for: Access to Axially Chiral Aryl Aldehydes via Carbene-Catalyzed Nitrile Formation and Desymmetrization Reaction
Source: Research (Wash D C). 2024 Jan 16;7:0293. doi: 10.34133/research.0293 (PMC11020146; doi:10.34133/research.0293)
Supplement: Supplementary 1 — Figs. S1 and S2 Tables S1 to S8 [file research.0293.f1.pdf]

Supplementary Information

Access to Axially Chiral Aryl Aldehydes via Carbene-Catalyzed Nitrile Formation and Desymmetrization Reaction

Yuanlin Cai,[1,†] Ya Lv,[1,†] Liangzhen Shu,[1] Zhichao Jin,[1] Yonggui Robin Chi,[1,2] Tingting Li[1,*]

[1] National Key Laboratory of Green Pesticide, Key Laboratory of Green Pesticide and Agricultural Bioengineering, Ministry of Education, Guizhou University, Guiyang, 550025, China.

[2] School of Chemistry, Chemical Engineering, and Biotechnology, Nanyang Technological University, Singapore, 637371, Singapore.

†These authors contributed equally to this work.

*Corresponding authors. E-mails: ttli@gzu.edu.cn.

Table of Contents

I. General information S1

II. Synthesis of substrates S3

III. Condition optimization S6

IV. General procedure for the catalytic reactions S7

V. Large-scale synthesis and synthetic transformations of **3a** S8

VI. Barriers to rotation of compound **3a** S11

VII. Control experiment S12

VIII. *X*-ray crystallography of compound **3a** S14

IX. References S19

X. Characterization of substrates and products S20

XI. ^{1}H NMR, ^{13}C NMR, ^{19}F NMR, and HPLC/UPLC spectra S44

I. General information

Commercially available materials purchased from Energy Chemical were used as received. Unless otherwise specified, all reactions were prepared using 4.0 mL vial under N_2 atmosphere in glove-box from UNILAB SP. NMR spectra were recorded on a Brüker ASCEND 400 (400 MHz) spectrometer (^{1}H: 400 MHz, ^{13}C: 101 MHz, ^{19}F: 377 MHz). Chemical shifts (δ) for ^{1}H and ^{13}C NMR spectra are given in ppm relative to TMS. The residual solvent signals were used as references for ^{1}H and ^{13}C NMR spectra and the chemical shifts converted to the TMS scale ($CDCl_3$: δH = 7.26 ppm, δC = 77.16 ppm; CD_3OD: δH = 3.31 ppm, δC = 49.15 ppm). The following abbreviations were used to explain the multiplicities: s = singlet, d = doublet, t = triplet, q = quartet, m = multiplet, and etc. All first-order splitting patterns were assigned on the base of the appearance of the multiplet. Splitting patterns that could not be easily interpreted are designated as multiplet (m) or broad (br). High resolution mass spectrometer analysis (HRMS) was performed on Thermo Fisher Q Exactive mass spectrometer. HPLC analyses were measured on Waters systems with Empower 3 system controller, Alliance column heater, 2998 Diode Array Waters 2489 UV/Vis detector, and Shimadzu LC-20AT instrument. Chiralcel brand chiral columns from Daicel Chemical Industries were used with models IB, ID, AD-H, AS-H, OD-H or OJ-H in 4.6 x 250 mm size. UPLC analyses were measured on Waters systems with Empower 3 system controller, Waters UPLC H-Class, and Waters ACQUITY UPLC PDA detector. Chiralcel brand chiral columns from Daicel Chemical Industries were used with models OD-3 in 3.0 x 100 mm size. Infrared spectra (IR) were obtained on a Thermo Fisher FT/IR-Nicolet iS50 spectrometer, and the absorptions have been reported in wavenumbers (cm^{-1}). Optical rotations were measured on a Insmark IP-digi Polarimeter in a 1 dm cuvette. The concentration (*c*) is given in g/100 mL. Melting point (m.p.): melting points were measured on a Beijing Tech Instrument X-4 digital display micro melting point apparatus and are uncorrected. Analytical thin-layer chromatography (TLC) was carried out on pre-coated silica gel plate (0.2 mm thickness). Visualization was performed

using a UV lamp.

II. Synthesis of substrates

2.1 General procedure for the synthesis of substrates 1a-1s and 1w. [1]

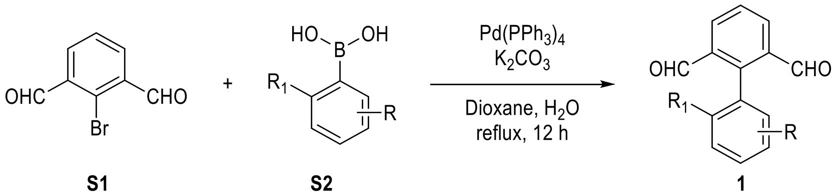

A 100 mL round-bottom Schlenk flask was added 2-bromoisophthalaldehyde **S1** (2.35 mmol, 1.0 equiv.), 2-Substituted phenylboronic acid derivatives **S2** (5.40 mmol, 2.3 equiv.), K_2CO_3 (16.20 mmol, 6.9 equiv.) and $Pd(PPh_3)_4$ (46.90 μmol, 0.02 equiv.), and charged with nitrogen three times. Then degassed 1,4-dioxane (30 mL) and water (4 mL) were added and the reaction was heated at 95 °C for 12 hours under nitrogen atmosphere. After cooling to room temperature, the mixture was poured into water and extracted with DCM three times. The organic layer was washed with brine and dried over anhydrous Na_2SO_4. The solvent was removed under vacuum. The residue was purified by silica gel column chromatography (PE:EA = 20:1 to 10:1).

2.2 Procedure for the synthesis of substrates 1v and 1w. [1-2]

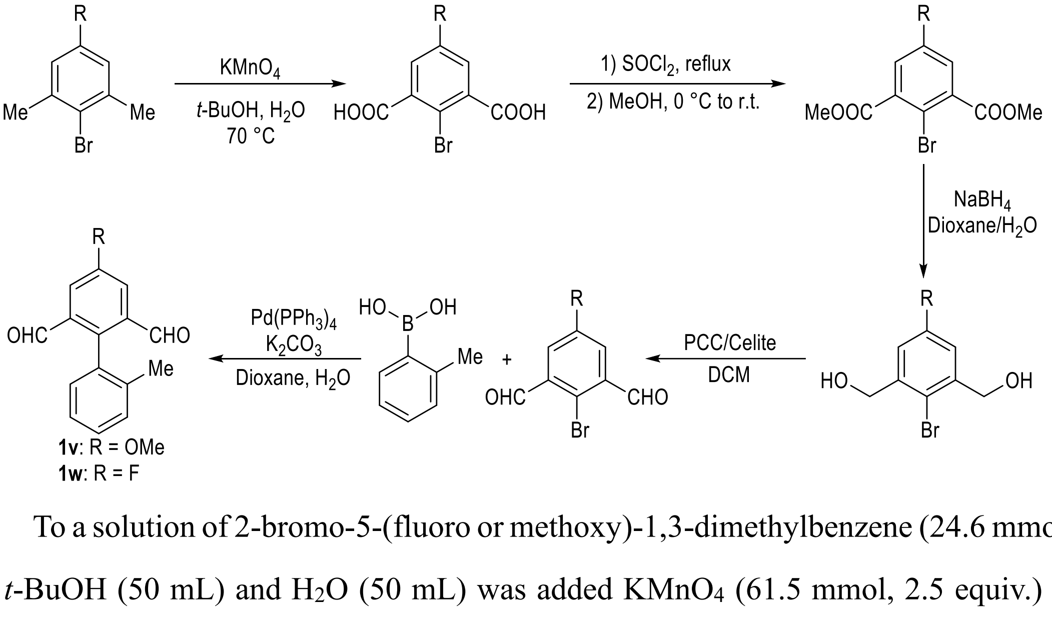

To a solution of 2-bromo-5-(fluoro or methoxy)-1,3-dimethylbenzene (24.6 mmol) in *t*-BuOH (50 mL) and H_2O (50 mL) was added $KMnO_4$ (61.5 mmol, 2.5 equiv.) in portions while stirring at room temperature. The mixture was attired at 70 °C for 2 hours before it was cooled to room temperature. A second batch of $KMnO_4$ (61.5 mmol, 2.5 equiv.) was added as before. After stirring at 70 °C for 10 hours, the hot reaction mixture

was filtered and the residue was washed with water. After concentration to100 mL, the filtrate was acidified in ice-bath to pH=2 with concd HCl to get white precipitate. After extraction with EtOAc, the organic phase was dried with Na_2SO_4 and concentrated in vacuo to give the corresponding benzenedicarboxylic acid compound (R = OMe, 75% yield. R = F, 71% yield).

Benzenedicarboxylic acid (15 mmol) in $SOCl_2$ (30 mL) was gradually heated to 100 °C for a period of 5 hours and stirred at 100 °C for another 4 hours. After $SOCl_2$ was evaporated in vacuo and the flask was cooled to 0 °C, methanol (20 mL) and triethylamine (10 mL) were added slowly while stirring. The reaction mixture was stirred at room temperature for 2 hours and was concentrated in vacuo. The residue was extracted with EtOAc, dried with Na_2SO_4 and concentrated in vacuo to obtain corresponding isophthalic acid dimethyl ester compound (R = OMe, 92% yield. R = F, 92% yield).

Isophthalic acid dimethyl ester (13.6 mmol) was dissolved in 70 mL 1,4-dioxane/H_2O (3:2, 70 mL) and cooled to 0 °C. To this mixture was added $NaBH_4$ (136 mmol) and stirred at room temperature for 2 daies before it was quenched with 6 M HCl in ice-bath, extracted with EtOAc, washed with saturated $NaHCO_3$ and brine, dried over Na_2SO_4 and concentrated in vacuo to give corresponding bis(hydroxymethyl) bromobenzene compound (R = OMe, 70% yield. R = F, 68% yield).

A mixture of bis(hydroxymethyl) bromobenzene (9.3 mmol), PCC (27.9 mmol) and Celite (9 g) in DCM was stirred at room temperature overnight. The reaction mixture was filtered through Celite and silica gel pad and the filtrate was evaporated in vacuo to obtain a white solid which was purified by flash column chromatography (PE:EA = 20:1 to 10:1) to give the corresponding bromobenzene dicarbaldehyde compound. (R = OMe, 85% yield. R = F, 80% yield)

A 100 mL round-bottom Schlenk flask was added 2-bromo-5-(methoxy or fluoro) isophthalaldehyde (2.35 mmol, 1.0 equiv.), 2-biphenylboronic acid (5.40 mmol, 2.3 equiv.), K_2CO_3 (16.20 mmol, 6.9 equiv.) and $Pd(PPh_3)_4$ (46.90 μmol, 0.02 equiv.), and charged with nitrogen three times. Then degassed 1,4-dioxane (30 mL) and water (4 mL) were added and the reaction was heated at 95 °C for 12 hours under nitrogen

atmosphere. After cooling to room temperature, the mixture was poured into water and extracted with DCM three times. The organic layer was washed with brine and dried over anhydrous Na_2SO_4. The solvent was removed under vacuum. The crude mixture was purified by silica gel column chromatography (PE:EA = 20:1 to 10:1) to give corresponding biphenyldialdehyde compound. (R = OMe, 45% yield. R = F, 70% yield)

III. Condition optimization

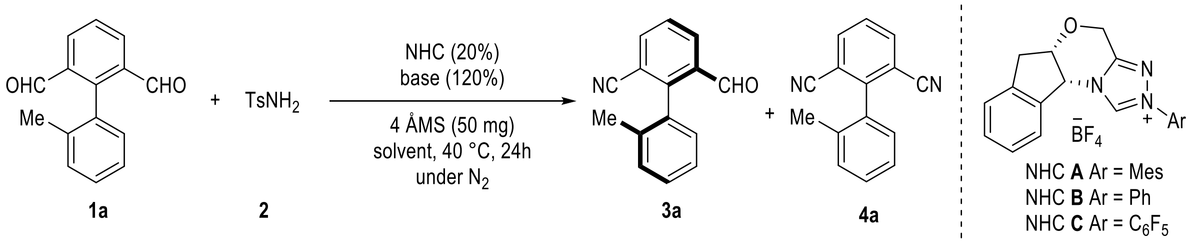

Entry	NHC	base	solvent	**3a** (%)[b]	er[c]	**4a** (%)
1	A	$NHEt_2$	DCE	trace	--	--
2	B	$NHEt_2$	DCE	50	96:4	16
3	C	$NHEt_2$	DCE	45	65:35	22
4	B	Cs_2CO_3	DCE	43	63:37	15
5	B	$NaHCO_3$	DCE	trace	--	--
6	B	DBU	DCE	44	79:21	19
7	B	NEt_3	DCE	55	82:18	21
8	B	$NHEt_2$	DCM	48	93:7	16
9	B	$NHEt_2$	$PhCH_3$	42	90:10	16
10	B	$NHEt_2$	$CHCl_3$	56	92:8	15
11	B	$NHEt_2$	THF	40	93:7	11
12[d]	B	$NHEt_2$	DCE	54	98:2	18
13[e]	B	$NHEt_2$	DCE	57	99:1	21
14[e,f]	B	$NHEt_2$	DCE	63	>99:1	15

[a] Unless otherwise specified, the reactions were carried using **1a** (0.12 mmol), **2** (0.10 mmol), NHC (0.02 mmol), base (0.12 mmol), 4Å MS (50 mg) and solvent (2.0 mL) at 40 °C under N_2 for 24 hours. [b] Isolated yield of **3a**. [c] The er values of **3a** were determined *via* HPLC on chiral stationary phase. [d] **1a** (0.10 mmol), **2** (0.20 mmol). [e] **1a** (0.10 mmol), **2** (0.30 mmol). [f] **1a** (0.10 mmol), **2** (0.30 mmol), 4Å MS (50 mg) and solvent (1.0 mL).

IV. General procedure for the catalytic reactions

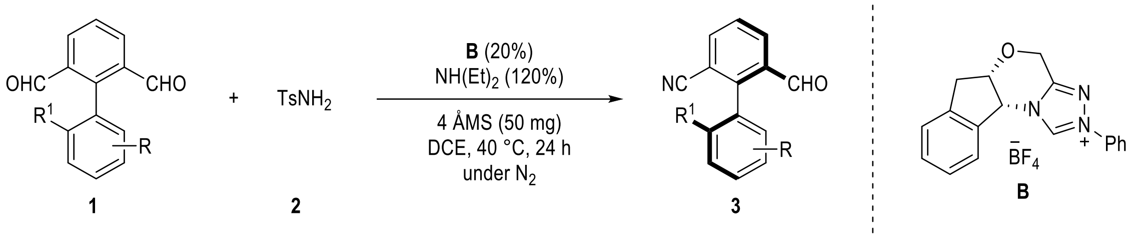

To a 4.0 mL oven-dried vial equipped with a magnetic stir bar was added chiral NHC pre-catalyst **B** (0.02 mmol, 7.5 mg), 4 Å molecular sieves (50 mg), substrates **1** (0.10 mmol) and **2** (0.30 mmol) in a glove box under N_2 atmosphere. Then anhydrous DCE (1.0 mL) and $NHEt_2$ (0.12 mmol, 12.4 μL) was added via syringe. The reaction mixture was stirred for 24 hours at 40 °C and subjected to column chromatography on silica gel (PE:EA = 20:1) directly to give the desired pure products **3** in 35% to 75% isolated yields.

V. Large-scale synthesis and synthetic transformations of 3a

Large-scale synthesis:

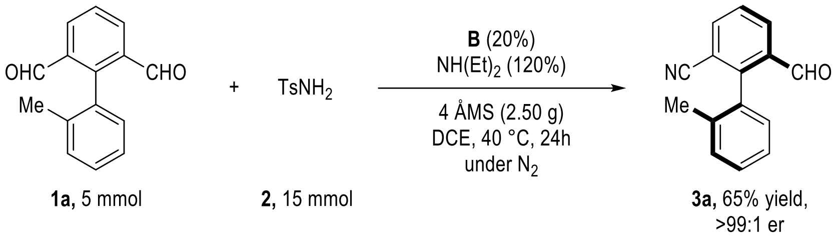

To a round-bottom Schlenk flask equipped with a magnetic stir bar, was added chiral NHC pre-catalyst **B** (0.10 mmol, 0.4 g), 4 Å molecular sieves (2.5 g), substrates **1a** (5.00 mmol, 1.12 g) and **2a** (1.50 mmol, 2.6 g). The Schlenk flask was sealed with a septum, evacuated and refilled with nitrogen three times. Anhydrous DCE (50 mL) and $NHEt_2$ (6.00 mmol, 0.6 mL) was added via syringe under N_2 atmosphere. Then the reaction mixture was stirred at 40 °C for 24 hours. The mixture was concentrated under reduced pressure and purified via column chromatography on silica gel (PE/EA = 20:1) to give the desired pure products **3a** in 65% yield (740 mg) with > 99:1 er value.

General procedure for the enantioselective synthesis of 5[3]

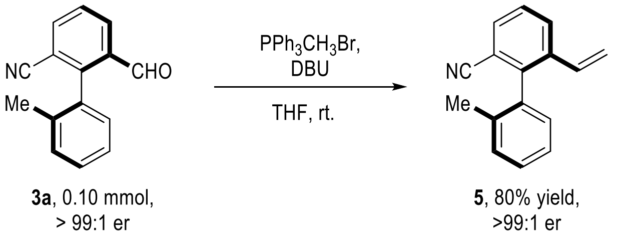

To a solution of **3a** (0.10 mmol, 22.1 mg) in dry THF was added PPh_3CH_3Br (0.20 mmol, 71.0 mg) and DBU (0.22 mmol, 33 μL), the reaction mixture was stirred at r.t. for 12 hours. Then the reaction mixture was diluted with water (5.0 ml) and extracted with EtOAc (5.0 mL× 3), the combined organic phase was washed with brine, dried over Na_2SO_4, and concentrated in vacuum. The crude product was purified by column chromatography on silica gel (PE:EA = 50:1) to give **5** in 80% yield (17.5 mg) with >99:1 er value.

General procedure for the enantioselective synthesis of 6[4]

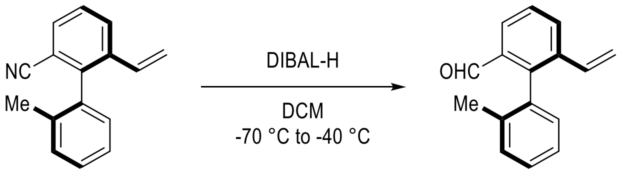

5, 0.10 mmol, >99:1 er

6, 86% yield, >99:1 er

To a Schlenk tube equipped with a magnetic stir bar, was added **5** (0.10 mmol, 21.9 mg). The Schlenk tube was sealed with a septum, evacuated and refilled with nitrogen three times. Anhydrous DCM (6.0 mL) was added via syringe under N_2 atmosphere, and the solution was cooled to -70 °C, whereupon DIBAL-H (0.12 mmol, 80 μL, 1.5M solution in hexanes) was added dropwise via a syringe. The solution was allowed to warm to -40 °C over a period of 1 h. And then HCl aqueous solution (1.0 mL, 1M solution in water) was added for hydrolysis. The reaction mixture was extracted with DCM (5.0 mL× 3), then the combined organic phase was washed with brine, dried over Na_2SO_4, and concentrated in vacuum. The crude product was purified by column chromatography on silica gel (PE:EA = 20:1) to give **6** in 86% yield (19.2 mg) with >99:1 er value.

General procedure for the enantioselective synthesis of 7[5]

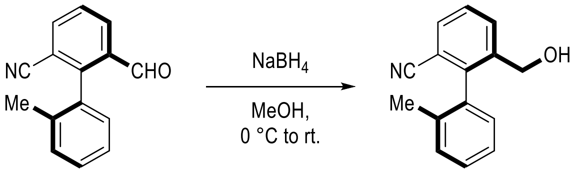

3a, 0.10 mmol, > 99:1 er

7, 94% yield, >99:1 er

To a solution of **3a** (0.10 mmol, 22.1 mg) in dry MeOH was added $NaBH_4$ (0.10 mmol, 3.8 mg) at 0 °C, then the reaction mixture was stirred at rt. for 1 h. The reaction mixture was quenched with ammonium chloride solution (2.0 mL) and extracted with EA (5.0 mL× 3), then the combined organic phase was washed with brine, dried over Na_2SO_4, and concentrated in vacuum. The crude product was purified by column chromatography on silica gel (PE:EA = 5:1) to give **7** in 94% yield (21.0 mg) with >99:1 er value.

General procedure foe the enantioselective synthesis of (8)[6]

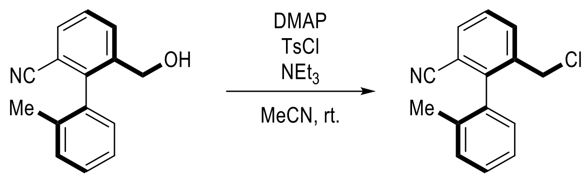

7, 0.10 mmol, >99:1 er → **8**, 70% yield, >99:1 er

To a solution of **7** (0.10 mmol, 22.1 mg) in MeCN was added DMAP (0.01 mmol, 1.2 mg) TsCl (0.20 mmol, 38.1 mg) and NEt_3 (0.20 mmol, 27.8 μL), the reaction mixture was stirred at rt. for 12 h. The reaction mixture was diluted with water (5.0 ml) and extracted with EA (5 mL × 3), then the combined organic phase was washed with brine, dried over Na_2SO_4, and concentrated in vacuum. The crude product was purified by column chromatography on silica gel (PE:EA = 50:1) to give **8** in 70% yield (17.0 mg) with >99:1 er value.

General procedure for the enantioselective synthesis of 9[7]

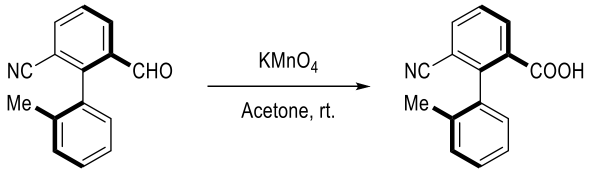

3a, 0.10 mmol, > 99:1 er → **9**, 84% yield, >99:1 er

The compound **3a** was solved in acetone and a suspension of 1.0 N $KMnO_4$ (0.12 mmol, 1.2 mL) was added in one portion and the mixture was stirred 24 hours. Then HCl aqueous solution (1 M solution in water) was added to adjust to pH = 2-3 and extracted with EA (5 mL × 3). The organic layer was dried over Na_2SO_4 and concentrated under reduced pressure. The crude product was purified by column chromatography on silica gel (PE:EA = 2:1) to give **9** in 84% yield (20.0 mg) with >99:1 er value.

VI. Barriers to rotation of compound 3a

Determination of $t_{1/2rac}$ for **3a**

The barrier to rotation of 3a was determined according to the literature method ($t_{1/2rac}$ = 1.92 h at 120 °C (393.15 K), mesitylene; $\Delta G^{\neq}$ = 30.95 kcal/mol[8].

Table 1. Change of enantiomer ratio with time (racemization)

Time (second)	ee	ln(ee_0/ee_t)
0	99.76	0
600	90.32	0.095189
1800	77.8	0.244407
3600	58.74	0.525427
7200	34.16	1.067493
10800	19.06	1.650956
14400	10.60	2.237694
18000	6.72	2.69346
21600	4.36	3.126076
25200	2.72	3.597916

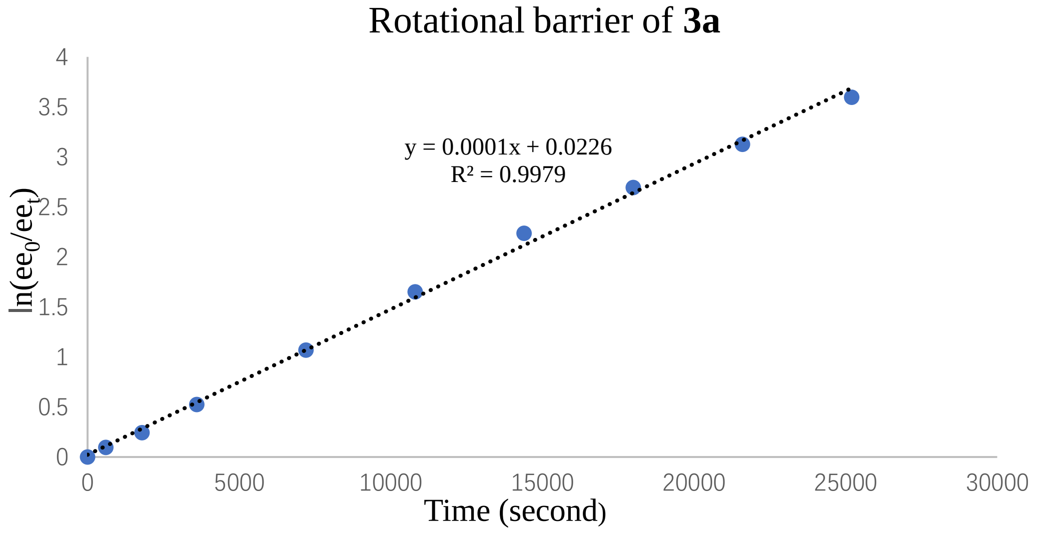

Figure 1. Racemization data and calculation of the barrier to rotation

$\ln(ee_0/ee_t) = 2k_{ent}t + C$

Therefore, k_{ent} = 1/2 slope = 0.00005 s^{-1}

$K_{rac} = 2k_{ent}$ = 0.0001 s^{-1}

$t_{1/2rac} = ln2/k_{rac}$ = 6931.47 s = 115.52 min = 1.92 h

$\Delta G^{\neq} = -RT\ln(k_{ent}h/k_B T)$ = 30.95 kcal/mol

VII. Control experiment

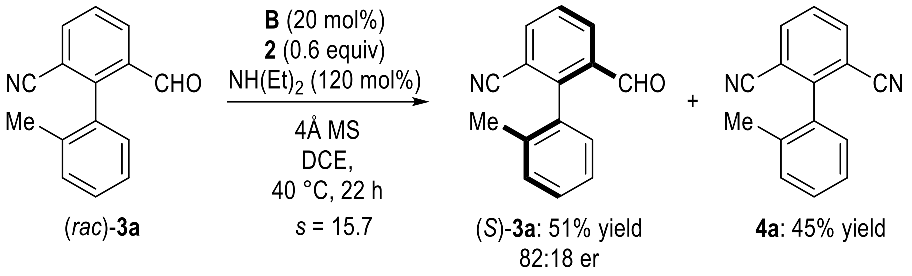

To a 4.0 mL oven-dried vial equipped with a magnetic stir bar was added chiral NHC pre-catalyst **B** (0.02 mmol, 7.5 mg), 4 Å molecular sieves (50 mg), (*Rac)*-**3a** (0.10 mmol) and **2** (0.06 mmol) in a glove box under N_2 atmosphere. Then dried DCE (1.0 mL) and $NHEt_2$ (0.12 mmol, 12.4 μL) was added via syringe. The reaction mixture was stirred for 22 hours at 40 °C and subjected to column chromatography on silica gel (PE:EA = 20:1 to 10:1) directly to give the desired pure product (*S*)-**3a** in 51% isolated yield with 82:12 er value and by-product **4a** 45% isolated yield.

<Chromatogram>

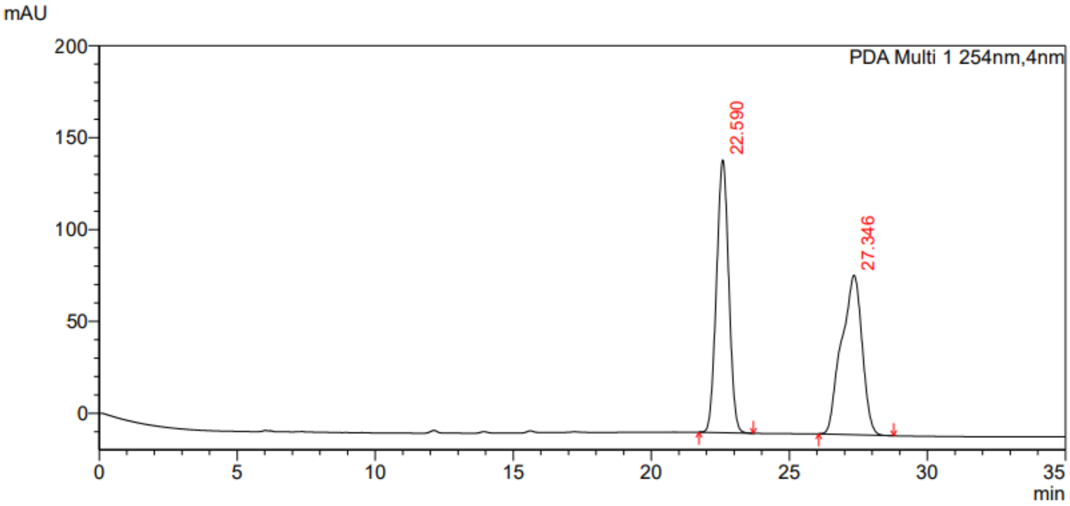

<Peak Table>

PDA Ch1 254nm

Ret. Time	Area	Height	Area%
22.590	4591714	148449	50.398
27.346	4519102	86783	49.602
	9110816	235232	100.000

<Chromatogram>

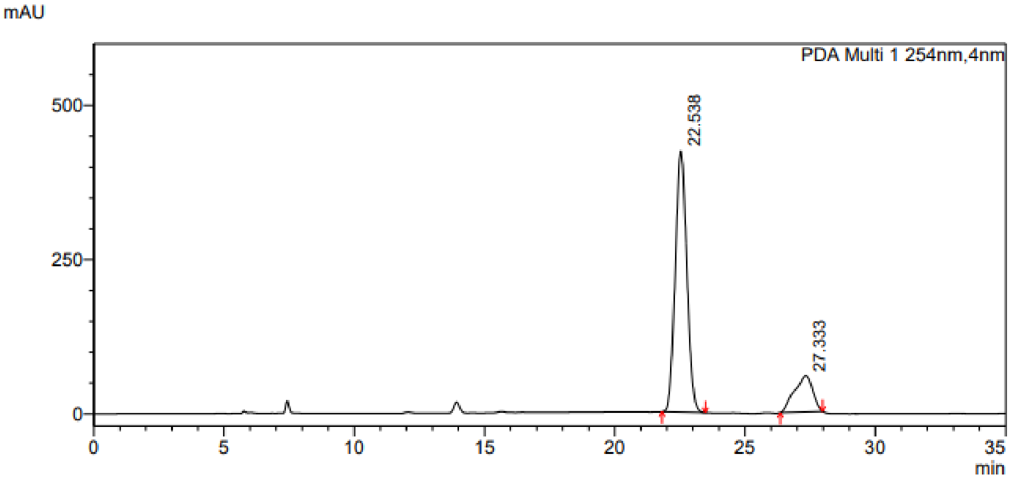

<Peak Table>

PDA Ch1 254nm

Ret. Time	Area	Height	Area%
22.538	13112327	423426	82.131
27.333	2852840	58411	17.869
	15965167	481837	100.000

VIII. *X*-ray crystallography of compound 3a

Good quality crystal of **3a** (colourless block crystals) was obtained by vaporization of a isopropanol / N-hexane solution of compound **3a**. Single colourless block crystals of **3a** were used as supplied. A suitable crystal with dimensions 0.33 × 0.31 × 0.28 mm^3 was selected and mounted on a Xcalibur, Eos, Gemini diffractometer. The crystal was kept at a steady T = 221(3) K during data collection. CCDC 2226525 contains the supplementary crystallographic data for this paper. These data can be obtained free of charge from the Cambridge Crystallographic Data Centre *via* https://www.ccdc.cam.ac.uk/.

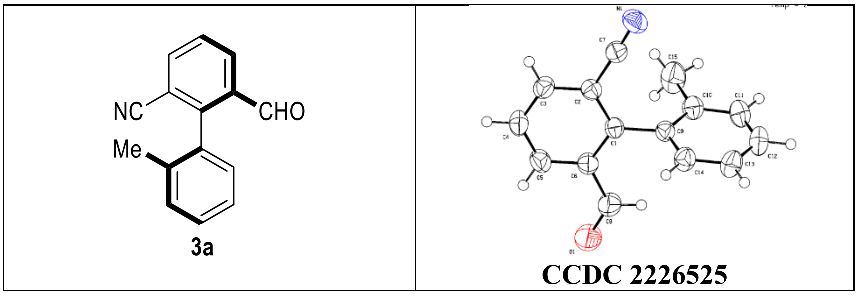

Figure 2. X-ray crystallography of compound **3a**

Crystal Data. $C_{15}H_{11}NO$, M_r = 221.25, monoclinic, $P2_1$ (No. 4), a = 8.2162(4) Å, b = 7.3886(4) Å, c = 10.0327(5)Å, β = 94.632(4)°, V = 607.06(5) Å^3, Z = 2, T = 221(3) K, μ(Cu K_a) = 0.641 mm^{-1}, Dcalc = 1.210 g/cm^3, 4512 reflections measured (8.842° ≤ 2Θ ≤ 142.128°), 2214 unique (R_{int} = 0.0249, R_{sigma} = 0.0284) which were used in all calculations. The final R_1 was 0.0382 ((I > 2σ(I)) and wR_2 was 0.01111 (all data).

Table 2. Crystal data and structure refinement for **3a**.

Empirical formula	$C_{15}H_{11}NO$
Formula weight	221.25
Temperature/K	221(3)
Crystal system	monoclinic
Space group	$P2_1$
a/Å	8.2162(4)
b/Å	7.3886(4)
c/Å	10.0327(5)
α/°	90
β/°	94.632(4)
γ/°	90
Volume/$Å^3$	607.06(5)
Z	2
$\rho_{calc}g/cm^3$	1.210
μ/mm^{-1}	0.604
F(000)	232.0
Crystal size/mm^3	0.33 × 0.31 × 0.28
Radiation	Cu Kα (λ = 1.54184)
2Θ range for data collection/°	8.842 to 142.128
Index ranges	$-8 \le h \le 10$, $-8 \le k \le 8$, $-12 \le l \le 10$
Reflections collected	4512
Independent reflections	2214 [R_{int} = 0.0249, R_{sigma} = 0.0284]
Data/restraints/parameters	2214/1/156
Goodness-of-fit on F^2	1.059
Final R indexes [I>=2σ (I)]	R_1 = 0.0382, wR_2 = 0.1079
Final R indexes [all data]	R_1 = 0.0409, wR_2 = 0.1111
Largest diff. peak/hole / e $Å^{-3}$	0.16/-0.12
Flack parameter	0.5(3

Table 3. Fractional Atomic Coordinates (×10^4) and Equivalent Isotropic Displacement Parameters (Å^2×10^3) for 3a. U_{eq} is defined as 1/3 of of the trace of the orthogonalised U_{IJ} tensor.

Atom	x	y	z	U(eq)
C1	-6850(3)	-2152(3)	-3595(2)	35.4(5)
C6	-8056(3)	-2328(4)	-4656(2)	42.4(6)
C7	-3910(3)	-2056(4)	-2863(2)	43.7(6)
C2	-5217(3)	-2254(3)	-3901(2)	38.0(5)
C5	-7633(3)	-2583(4)	-5961(2)	48.7(7)
C8	-9811(3)	-2291(6)	-4412(3)	59.0(8)
O1	-10880(2)	-2283(6)	-5303(2)	92.5(10)
C9	-7277(3)	-1789(3)	-2198(2)	36.6(5)
N1	-2833(3)	-1890(4)	-2074(2)	58.8(7)
C3	-4815(3)	-2508(4)	-5213(2)	43.8(6)
C4	-6026(3)	-2670(4)	-6236(2)	47.6(6)
C10	-7196(3)	-3138(4)	-1229(3)	44.9(6)
C14	-7803(3)	-50(4)	-1883(3)	46.8(6)
C11	-7660(4)	-2715(4)	37(3)	53.4(7)
C12	-8168(4)	-1000(5)	347(3)	57.4(8)
C13	-8238(4)	343(4)	-613(3)	54.5(7)
C15	-6643(5)	-5026(5)	-1527(3)	69.4(10)

Table 4. Anisotropic Displacement Parameters (Å^2×10^3) for 3a. The Anisotropic displacement factor exponent takes the form: $-2\pi^2[h^2a^{*2}U_{11}+2hka^*b^*U_{12}+\ldots]$.

Atom	U_{11}	U_{22}	U_{33}	U_{23}	U_{13}	U_{12}
C1	37.4(11)	35.0(12)	34.0(11)	0.3(9)	3.6(8)	-2.2(9)
C6	36.2(12)	52.0(15)	38.8(12)	-2.4(11)	2.4(9)	-4.1(11)
C7	38.1(12)	50.5(15)	42.7(12)	-0.3(11)	4.3(10)	-1.4(10)
C2	37.3(11)	38.9(12)	37.5(11)	0.8(10)	1.4(9)	-3.1(10)
C5	43.8(13)	65.2(19)	36.2(12)	-4.8(12)	-3.1(10)	-6.6(12)
C8	36.1(13)	90(2)	50.6(14)	-12.0(16)	2.4(11)	-3.8(15)
O1	37.5(10)	172(3)	66.6(14)	-26.6(19)	-5.9(9)	-1.3(16)
C9	33.7(11)	42.9(13)	33.4(11)	-1.9(9)	3.8(8)	-2.9(9)
N1	47.3(12)	77.9(19)	49.3(13)	2.3(12)	-6.7(10)	-4.2(11)
C3	38.2(11)	49.7(15)	44.4(13)	-2.4(12)	8.9(9)	-2.0(11)
C4	52.3(14)	57.6(16)	34.0(11)	-3.2(11)	9.8(10)	-6.1(12)
C10	48.0(13)	45.7(14)	41.5(13)	2.7(11)	7.0(10)	-3.5(11)
C14	54.1(15)	43.7(15)	43.5(13)	2.7(11)	10.0(11)	2.1(11)
C11	64.9(16)	57.6(18)	38.2(13)	5.9(12)	8.3(11)	-6.6(13)
C12	68.6(18)	67.7(19)	37.6(13)	-7.9(13)	15.4(13)	-7.0(15)
C13	65.1(18)	48.6(17)	51.8(15)	-9.0(12)	16.2(13)	2.0(13)
C15	103(3)	47.3(18)	60.9(18)	9.7(14)	23.1(19)	6.0(16)

Table 5. Bond Lengths for **3a**.

Atom	Atom	Length/Å	Atom	Atom	Length/Å
C1	C6	1.400(3)	C8	O1	1.201(3)
C1	C2	1.402(3)	C9	C10	1.390(3)
C1	C9	1.497(3)	C9	C14	1.399(4)
C6	C5	1.394(3)	C3	C4	1.376(4)
C6	C8	1.482(3)	C10	C11	1.391(4)
C7	C2	1.442(3)	C10	C15	1.505(4)
C7	N1	1.145(3)	C14	C13	1.382(4)
C2	C3	1.395(3)	C11	C12	1.378(5)
C5	C4	1.373(4)	C12	C13	1.381(5)

Table 6. Bond Angles for **3a**.

Atom	Atom	Atom	Angle/°	Atom	Atom	Atom	Angle/°
C6	C1	C2	117.3(2)	C10	C9	C1	121.6(2)
C6	C1	C9	121.59(19)	C10	C9	C14	119.9(2)
C2	C1	C9	121.03(19)	C14	C9	C1	118.4(2)
C1	C6	C8	120.7(2)	C4	C3	C2	120.2(2)
C5	C6	C1	120.8(2)	C5	C4	C3	119.7(2)
C5	C6	C8	118.5(2)	C9	C10	C11	118.4(3)
N1	C7	C2	177.5(3)	C9	C10	C15	121.6(2)
C1	C2	C7	120.4(2)	C11	C10	C15	120.0(2)
C3	C2	C1	121.2(2)	C13	C14	C9	120.5(2)
C3	C2	C7	118.4(2)	C12	C11	C10	121.5(3)
C4	C5	C6	120.8(2)	C11	C12	C13	120.0(3)
O1	C8	C6	122.7(3)	C12	C13	C14	119.5(3)

Table 7. Torsion Angles for **3a**.

A	B	C	D	Angle/°	A	B	C	D	Angle/°
C1	C6	C5	C4	0.0(5)	C2	C3	C4	C5	-0.1(5)
C1	C6	C8	O1	173.6(4)	C5	C6	C8	O1	-7.9(6)
C1	C2	C3	C4	-0.2(4)	C8	C6	C5	C4	-178.5(3)
C1	C9	C10	C11	-178.0(2)	C9	C1	C6	C5	177.0(3)
C1	C9	C10	C15	1.2(4)	C9	C1	C6	C8	-4.5(4)
C1	C9	C14	C13	178.8(2)	C9	C1	C2	C7	1.7(4)
C6	C1	C2	C7	179.0(2)	C9	C1	C2	C3	-177.0(2)
C6	C1	C2	C3	0.4(4)	C9	C10	C11	C12	-1.0(4)
C6	C1	C9	C10	104.5(3)	C9	C14	C13	C12	-0.6(4)
C6	C1	C9	C14	-74.1(3)	C10	C9	C14	C13	0.2(4)
C6	C5	C4	C3	0.2(5)	C10	C11	C12	C13	0.5(5)
C7	C2	C3	C4	-178.9(3)	C14	C9	C10	C11	0.6(4)
C2	C1	C6	C5	-0.3(4)	C14	C9	C10	C15	179.8(3)
C2	C1	C6	C8	178.2(3)	C11	C12	C13	C14	0.3(5)
C2	C1	C9	C10	-78.3(3)	C15	C10	C11	C12	179.8(3)
C2	C1	C9	C14	103.1(3)					

Table 8. Hydrogen Atom Coordinates (Å×10^4) and Isotropic Displacement Parameters (Å^2×10^3) for **3a**.

Atom	x	y	z	U(eq)
H5	-8459.38	-2697.94	-6660.2	58
H8	-10099.34	-2271.96	-3524.8	71
H3	-3712.89	-2567.93	-5397.84	53
H4	-5753.23	-2839.37	-7119.62	57
H14	-7859.47	858.3	-2541.55	56
H11	-7625.81	-3621.06	697.03	64
H12	-8467.48	-743.82	1212.27	69
H13	-8580.3	1516.19	-404.4	65
H15A	-5556.16	-4981.79	-1838.77	104
H15B	-7392.78	-5559.18	-2214.08	104
H15C	-6623.31	-5754.37	-721.81	104

IX. **References**

[1] Urgel, J. I.; Di Giovannantonio, M.; Eimre, K.; Lohr, T. G.; Liu, J.; Mishra, S.; Sun, Q.; Kinikar, A.; Widmer, R.; Stolz, S.; Bommert, M.; Berger, R.; Ruffieux, P.; Pignedoli, C. A.; Müllen, K.; Feng, X.; Fasel, R. *Angew. Chem. Int. Ed.* **2020**, *59*, 13281.

[2] Ye, L.; Ding, D.; Feng, Y.; Xie, D.; Wu, P.; Guo, H.; Meng, Q.; Zhou, H. *Tetrahedron*. **2009**, *65*, 8738.

[3] Scheidt, F.; Schäfer, M.; Sarie, J. C.; Daniliuc, C. G.; Molloy, J. J.; Gilmour, R. *Angew. Chem. Int. Ed.* **2018**, *57*, 16431.

[4] Raap, J.; Nieuwenhuis, S.; Creemers, A.; Hexspoor, S.; Kragl, U.; Lugtenburg, J. *Eur. J. Org. Chem.* **1999**, *1999*, 2609.

[5] Liu, J.; Zheng, H.-X.; Yao, C.-Z.; Sun, B.-F.; Kang, Y.-B. *J. Am. Chem. Soc.* **2016**, *138*, 3294.

[6] Jones, C. S.; Bull, S. D.; Williams, J. M. *J. Org. Biomol. Chem.* **2016**, *14*, 8452.

[7] Wu, Y.; Li, M.; Sun, J.; Zheng, G.; Zhang, Q. *Angew. Chem. Int. Ed.* **2022**, *61*, e202117340.

[8] (a) Ma, C.; Sheng, F.-T.; Wang, H.-Q.; Deng, S.; Zhang, Y.-C.; Jiao, Y.; Tan, W.; Shi, F. *J. Am. Chem. Soc.* **2020**, *142*, 15686.(b) Wang, Q.; Zhang, W.-W.; Song, H.; Wang, J.; Zheng, C.; Gu, Q.; You, S.-L. *J. Am. Chem. Soc*. **2020**, *142*, 15678.

X. **Characterization of substrates and products**

2'-methyl-[1,1'-biphenyl]-2,6-dicarbaldehyde (1a)

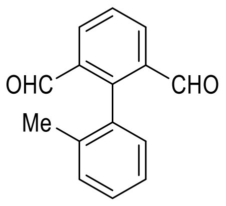

White solid, 80% yield, 420 mg; m.p. 70-71 °C.

^{1}H NMR (400 MHz, $CDCl_3$) δ 9.70 (d, *J* = 0.8 Hz, 2H), 8.27 (d, *J* = 7.8 Hz, 2H), 7.70 – 7.65 (m, 1H), 7.43 (td, *J* = 7.5, 1.5 Hz, 1H), 7.34 (m, 2H), 7.24 (dd, *J* = 7.5, 1.5 Hz, 1H), 2.06 (s, 3H).

^{13}C NMR (101 MHz, $CDCl_3$) δ 190.9, 147.9, 136.8, 134.5, 132.7, 132.2, 130.7, 130.4, 129.4, 128.5, 126.0, 20.4.

IR (thin film): ν_{max} (cm^{-1}) =3060, 2858, 2754, 1684, 1572, 1453,1388,1238, 921, 802, 734, 700, 557.

HRMS (ESI, m/z) calcd. for $C_{15}H_{11}O_2^-$ $[M-H]^-$: 223.0764, found: 223.0763

2',3'-dimethyl-[1,1'-biphenyl]-2,6-dicarbaldehyde (1b)

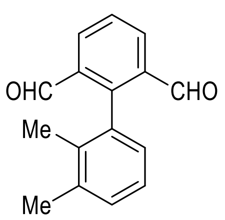

White solid, 98% yield, 549 mg; m.p. 98-99 °C.

^{1}H NMR (400 MHz, $CDCl_3$) δ 9.69 (d, *J* = 0.9 Hz, 2H), 8.26 (d, *J* = 7.7 Hz, 2H), 7.65 (m, 1H), 7.33 – 7.28 (m, 1H), 7.21 (t, *J* = 7.5 Hz, 1H), 7.10 – 7.05 (m, 1H), 2.36 (s, 3H), 1.96 (s, 3H).

^{13}C NMR (101 MHz, $CDCl_3$) δ 191.1, 148.7, 137.7, 135.5, 134.7, 132.5, 132.1, 130.8, 128.6, 128.3, 125.6, 20.4, 17.1.

IR (thin film): ν_{max} (cm^{-1}) = 3063, 2860, 2760, 1677, 1568, 1451, 1388, 1236, 927, 795, ,733, 702, 581,551.

HRMS (ESI, m/z) calcd. for $C_{16}H_{14}O_2Na^+$ $[M+Na]^+$: 261.0886, found 261.0886.

2',4'-dimethyl-[1,1'-biphenyl]-2,6-dicarbaldehyde (1c)

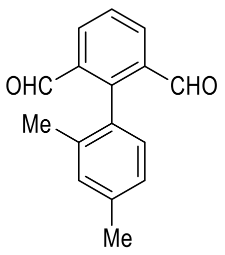

White oil, 86% yield, 480 mg.

^{1}H NMR (400 MHz, $CDCl_3$) δ 9.69 (s, 2H), 8.23 (d, *J* = 7.9 Hz, 2H), 7.63 (t, *J* = 7.9 Hz, 1H), 7.13 (d, *J* = 19.5 Hz, 3H), 2.39 (s, 3H), 2.00 (s, 3H).

^{13}C NMR (101 MHz, $CDCl_3$) δ 191.1, 148.2, 139.2, 136.6, 134.7, 132.6, 131.1, 130.7, 129.0, 128.4, 126.8, 21.2, 20.4.

IR (thin film): ν_{max} (cm^{-1}) =2926, 2856, 2750, 1690, 1567, 1505, 1449, 1382, 1237, 920, 823, 759, 701, 577, 503.

HRMS (ESI, m/z) calcd. for $C_{16}H_{14}O_2Na^+$ [M+Na]$^+$: 261.0886, found 261.0886.

2',5'-dimethyl-[1,1'-biphenyl]-2,6-dicarbaldehyde (1d)

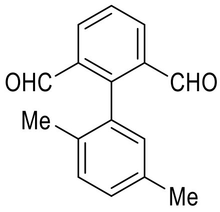

White solid, 89% yield, 500 mg; m.p. 62-63 °C.

^{1}H NMR (400 MHz, $CDCl_3$) δ 9.69 (d, *J* = 0.9 Hz, 2H), 8.25 (d, *J* = 7.9 Hz, 2H), 7.68 – 7.59 (m, 1H), 7.21 (t, *J* = 1.5 Hz, 2H), 7.04 (d, *J* = 1.7 Hz, 1H), 2.35 (s, 3H), 2.00 (s, 3H).

^{13}C NMR (101 MHz, $CDCl_3$) δ 191.1, 148.3, 135.6, 134.5, 133.7, 132.6, 131.9, 131.3, 130.2, 130.1, 128.4, 20.9, 19.9.

IR (thin film): ν_{max} (cm^{-1}) = 3063, 2918, 2859, 2759, 1679, 1570, 1498, 1451, 1386, 1240, 922, 798, 740, 736, 589, 553.

HRMS (ESI, m/z) calcd. for $C_{16}H_{14}O_2Na^+$ [M+Na$^+$: 261.0886, found 261.0886.

2',4',5'-trimethyl-[1,1'-biphenyl]-2,6-dicarbaldehyde (1e)

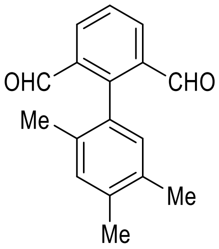

White solid, 95% yield, 562 mg; m.p. 45-46 °C.

^{1}H NMR (400 MHz, $CDCl_3$) δ 9.71 (s, 2H), 8.23 (d, *J* = 7.7 Hz, 2H), 7.62 (t, *J* = 7.7 Hz, 1H), 7.10 (s, 1H), 6.98 (s, 1H), 2.30 (s, 3H), 2.25 (s, 3H), 1.97 (s, 3H).

^{13}C NMR (101 MHz, $CDCl_3$) δ 191.3, 148.4, 137.8, 134.7, 134.2, 134.0, 132.5, 131.9, 131.6, 129.2, 128.2, 19.8, 19.5, 19.3.

IR (thin film): ν_{max} (cm^{-1}) = 2924, 2857, 2746, 1680, 1568, 1500, 1448, 1378, 1233, 926, 794, 699, 610, 565, 505.

HRMS (ESI, m/z) calcd. for $C_{17}H_{16}O_2Na^+$ [M+Na]$^+$: 275.1042, found 275.1042.

4'-methoxy-2'-methyl-[1,1'-biphenyl]-2,6-dicarbaldehyde (1f)

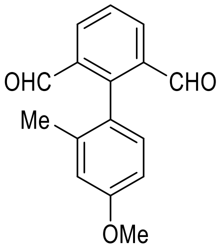

White solid, 77% yield, 460 mg; m.p. 47-48 ºC.

^{1}H NMR (400 MHz, $CDCl_3$) δ 9.72 (d, J = 0.8 Hz, 2H), 8.24 (d, J = 7.7 Hz, 2H), 7.64 (m, 1H), 7.14 (d, J = 8.2 Hz, 1H), 6.90 – 6.83 (m, 2H), 3.86 (s, 3H), 2.02 (s, 3H).

^{13}C NMR (101 MHz, $CDCl_3$) δ 191.2, 160.2, 147.9, 138.4, 135.0, 132.7, 131.8, 128.4, 124.1, 115.8, 111.5, 55.4, 20.7.

IR (thin film): ν_{max} (cm^{-1}) = 2963, 2838, 2727, 1677, 1604, 1498, 1454, 1374, 1293, 1243, 1167, 1127, 1053, 912, 863, 807, 758, 693, 569, 515.

HRMS (ESI, m/z) calcd. for $C_{16}H_{14}O_2Na^+$ $[M+Na]^+$: 277.0835, found 277.0831.

4'-fluoro-2'-methyl-[1,1'-biphenyl]-2,6-dicarbaldehyde (1g)

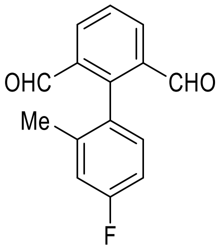

White solid, 59% yield, 336 mg; m.p. 73-74 ºC.

^{1}H NMR (400 MHz, $CDCl_3$) δ 9.70 (d, J = 0.8 Hz, 2H), 8.26 (d, J = 7.7 Hz, 2H), 7.69 (m, 1H), 7.22 (dd, J = 8.4, 5.7 Hz, 1H), 7.12 – 7.01 (m, 2H), 2.05 (s, 3H).

^{13}C NMR (101 MHz, $CDCl_3$) δ 190.6, 163.1 (d, J = 249.1 Hz), 146.7, 139.5 (d, J = 8.1 Hz), 134.8, 133.0, 132.1 (d, J = 8.3 Hz), 128.8, 128.1 (d, J = 3.2 Hz), 117.2 (d, J = 21.6 Hz), 113.3 (d, J = 21.6 Hz), 20.6.

^{19}F NMR (377 MHz, $CDCl_3$) δ -112.38.

IR (thin film): ν_{max} (cm^{-1}) = 3058, 2854, 2750, 1684, 1567, 1492, 1447, 1387, 1230, 1155, 1002, 935, 912804, 693, 577, 508.

HRMS (ESI, m/z) calcd. for $C_{15}H_{11}FNa^+$ $[M+Na]^+$: 265.0635, found 265.0627.

5'-fluoro-2'-methyl-[1,1'-biphenyl]-2,6-dicarbaldehyde (1h)

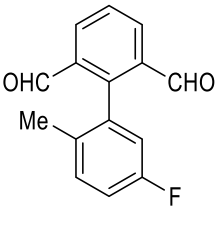

White solid, 95% yield, 540mg; m.p. 91-92 ºC.

^{1}H NMR (400 MHz, $CDCl_3$) δ 9.71 (d, J = 0.8 Hz, 2H), 8.27 (d, J = 7.7 Hz, 2H), 7.72 – 7.67 (m, 1H), 7.33 (dd, J = 8.6, 5.6 Hz, 1H), 7.14 (td, J = 8.4, 2.8 Hz, 1H), 7.00 (dd, J = 8.6, 2.8 Hz, 1H), 2.01 (s, 3H).

^{13}C NMR (101 MHz, $CDCl_3$) δ 190.3, 160.8 (d, J = 247.7 Hz), 146.3, 134.3, 134.0 (d, J = 7.3 Hz), 132.7, 132.6, 131.9 (d, J = 8.1 Hz), 129.0, 117.5 (d, J = 21.9 Hz), 116.3 (d, J = 20.7 Hz), 19.6.

IR (thin film): ν_{max} (cm^{-1}) = 3073, 2856, 2743, 1688, 1571, 1448, 1382, 1237, 1186, 923, 794, 695, 603, 555.

^{19}F NMR (377 MHz, $CDCl_3$) δ -116.46.

HRMS (ESI, m/z) calcd. for $C_{15}H_{11}FNa^+$ $[M+Na]^+$: 265.0635, found 265.0632.

3'-chloro-2'-methyl-[1,1'-biphenyl]-2,6-dicarbaldehyde (1i)

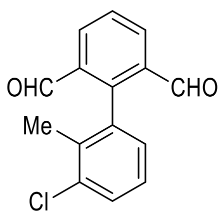

White solid, 95% yield, 562 mg; m.p. 111-112 ºC.

^{1}H NMR (400 MHz, $CDCl_3$) δ 9.70 (d, J = 0.8 Hz, 2H), 8.27 (d, J = 7.7 Hz, 2H), 7.70 (m, 1H), 7.54 (dd, J = 8.0, 1.3 Hz, 1H), 7.28 (m, 1H), 7.16 (dd, J = 7.6, 1.3 Hz, 1H), 2.10 (s, 3H).

^{13}C NMR (101 MHz, $CDCl_3$) δ 190.4, 146.8, 135.9, 135.3, 134.4, 134.2, 133.0, 130.3, 129.2, 128.9, 126.8, 18.0.

IR (thin film): ν_{max} (cm^{-1}) = 3063, 2863, 2763, 1682, 1561, 1454, 1388, 1241, 1030, 910, 801, 728, 698, 584.

HRMS (ESI, m/z) calcd. for $C_{15}H_{10}ClO_2^-$ $[M-H]^-$: 257.0374, found 257.0375.

4'-chloro-2'-methyl-[1,1'-biphenyl]-2,6-dicarbaldehyde (1j)

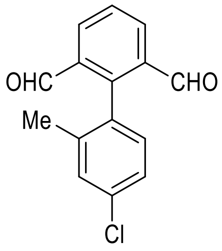

White solid, 76% yield, 462 mg; m.p. 62-64 ºC.

^{1}H NMR (400 MHz, $CDCl_3$) δ 9.70 (d, J = 0.8 Hz, 2H), 8.26 (d, J = 7.7 Hz, 2H), 7.69 (m, 1H), 7.38 (dt, J = 2.2, 0.6 Hz, 1H), 7.33 (m, 1H), 7.18 (d, J = 8.0 Hz, 1H), 2.04 (s, 3H).

^{13}C NMR (101 MHz, $CDCl_3$) δ 190.4, 146.3, 138.8, 135.2, 134.6, 133.1, 131.7, 130.8, 130.4, 128.9, 126.3, 20.4.

IR (thin film): ν_{max} (cm^{-1}) = 3070, 2853, 2749, 1685, 1570, 1487, 1450, 1384, 1235, 1125, 918, 871, 841, 791, 755, 691, 570, 501.

HRMS (ESI, m/z) calcd. for $C_{15}H_{10}ClO_2^-$ $[M-H]^-$: 257.0374, found: 257.0366.

5'-chloro-2'-methyl-[1,1'-biphenyl]-2,6-dicarbaldehyde (1k)

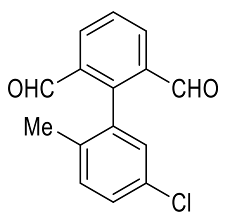

White solid, 86% yield, 522 mg; m.p. 77-78 °C.

^{1}H NMR (400 MHz, $CDCl_3$) δ 9.71 (d, *J* = 0.8 Hz, 2H), 8.28 (d, *J* = 7.7 Hz, 2H), 7.70 (m, 1H), 7.41 (dd, *J* = 8.2, 2.2 Hz, 1H), 7.30 (d, *J* = 8.2 Hz, 1H), 7.27 (d, *J* = 2.4 Hz, 1H), 2.02 (s, 3H).

^{13}C NMR (101 MHz, $CDCl_3$) δ 190.3, 146.1, 135.4, 134.4, 134.1, 133.1, 132.1, 131.6, 130.2, 129.5, 129.0, 19.9.

IR (thin film): ν_{max} (cm^{-1}) = 3061, 2863, 2760, 1683, 1572, 1454, 1396, 1239, 1098, 1015, 925, 818, 797, 740, 617, 645, 587.

HRMS (ESI, m/z) calcd. for $C_{15}H_{10}ClO_2^-$ [M-H] $^-$: 257.0374, found: 257.0375.

2'-ethyl-[1,1'-biphenyl]-2,6-dicarbaldehyde (1l)

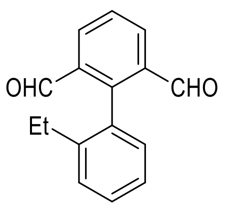

White solid, 84% yield, 497 mg; m.p. 31-32 °C.

^{1}H NMR (400 MHz, $CDCl_3$) δ 9.69 (d, *J* = 0.9 Hz, 2H), 8.25 (dd, *J* = 7.7, 0.8 Hz, 2H), 7.66 (m, 1H), 7.45 (m, 1H), 7.39 (m, 1H), 2.35 (q, *J* = 7.5 Hz, 2H), 1.00 (t, *J* = 7.5 Hz, 3H).

^{13}C NMR (101 MHz, $CDCl_3$) δ 190.9, 147.7, 142.8, 134.7, 132.6, 131.5, 130.7, 129.6, 128.7, 128.5, 125.9, 26.6, 14.6.

IR (thin film): ν_{max} (cm^{-1}) = 2964, 2858, 2756, 1687, 1574, 1494, 1457, 1384, 1240, 916, 801, 701, 571.

HRMS (ESI, m/z) calcd. for $C_{16}H_{14}O_2Na^+$ [M+Na] $^+$: 261.0886, found: 261.0885.

2'-(methylthio)-[1,1'-biphenyl]-2,6-dicarbaldehyde (1m)

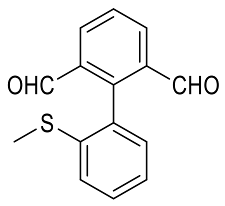

White solid, 93% yield, 560 mg; m.p. 103-104 °C.

^{1}H NMR (400 MHz, $CDCl_3$) δ 9.66 (d, *J* = 0.8 Hz, 2H), 8.27 (d, *J* = 7.7 Hz, 2H), 7.68 (m, 1H), 7.50 (m, 1H), 7.29 (m, 2H), 7.22 (dd, *J* = 7.4, 1.6 Hz, 1H), 2.39 (s, 3H).

^{13}C NMR (101 MHz, $CDCl_3$) δ 190.8, 146.0, 139.3, 134.7, 132.6, 131.3, 130.5, 130.0, 129.0, 124.6, 124.2, 15.2.

IR (thin film): ν_{max} (cm^{-1}) = 3060, 2863, 2758, 1683, 1574, 1428, 1390, 1243, 947, 916,

797, 738, 696, 681, 551.

HRMS (ESI, m/z) calcd. for $C_{15}H_{12}O_2SNa^+$ $[M+Na]^+$: 279.0450, found 279.0452.

2'-vinyl-[1,1'-biphenyl]-2,6-dicarbaldehyde (1n)

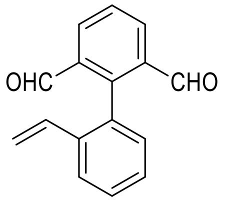

White solid, 54% yield, 300 mg; m.p. 88-89 ºC.

^{1}H NMR (400 MHz, $CDCl_3$) δ 9.68 (s, 2H), 8.27 (d, *J* = 7.7 Hz, 2H), 7.76 – 7.63 (m, 2H), 7.46 (m, 2H), 7.27 (d, *J* = 7.2 Hz, 1H), 6.28 (dd, *J* = 17.4, 10.9 Hz, 1H), 5.67 (d, *J* = 17.4 Hz, 1H), 5.19 (d, *J* = 10.9 Hz, 1H).

^{13}C NMR (101 MHz, $CDCl_3$) δ 190.8, 146.9, 137.9, 134.9, 133.9, 132.6, 131.3, 130.9, 129.6, 128.7, 127.7, 125.7, 117.7.

IR (thin film): ν_{max} (cm^{-1}) = 3091, 3016, 2098, 1684, 1574, 1455, 1396, 1236, 1108, 990, 921, 803, 779, 697, 569.

HRMS (ESI, m/z) calcd. for $C_{16}H_{12}O_2Na^+$ $[M+Na]^+$: 259.0729, found 259.0728.

2'-chloro-[1,1'-biphenyl]-2,6-dicarbaldehyde (1o)

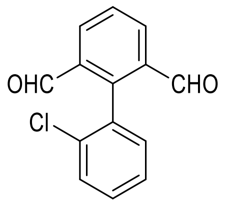

White solid, 85% yield, 489 mg; m.p. 77-78 ºC.

^{1}H NMR (400 MHz, $CDCl_3$) δ 9.71 (d, *J* = 0.9 Hz, 2H), 8.27 (d, *J* = 7.7 Hz, 2H), 7.71 (m, 1H), 7.58 (dd, *J* = 7.9, 1.4 Hz, 1H), 7.49 (m 1H), 7.43 (m, 1H), 7.35 (dd, *J* = 7.5, 1.8 Hz, 1H).

^{13}C NMR (101 MHz, $CDCl_3$) δ 190.2, 144.8, 134.5, 134.2, 132.9, 132.3, 131.9, 130.8, 129.9, 129.2, 127.0.

IR (thin film): ν_{max} (cm^{-1}) = 2857, 2757, 1689, 1575, 1446, 1386, 1230, 1065, 918, 791, 741, 697, 553.

HRMS (ESI, m/z) calcd. for $C_{14}H_9ClO_2Na^+$ $[M+Na]^+$: 267.0183, found 267.0180.

2'-bromo-[1,1'-biphenyl]-2,6-dicarbaldehyde (1p)

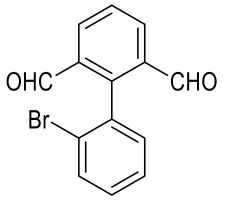

Yellow solid, 90% yield, 611mg; m.p. 96-97 ºC.

^{1}H NMR (400 MHz, $CDCl_3$) δ 9.72 (d, *J* = 0.8 Hz, 2H), 8.28 (d, *J* = 7.7 Hz, 2H), 7.77 (dd, *J* = 8.0, 1.3 Hz, 1H), 7.72 (m, 1H), 7.49 (m, 1H), 7.41 (m, 1H), 7.37 (dd, *J* = 7.4, 1.8 Hz, 1H).

^{13}C NMR (101 MHz, $CDCl_3$) δ 190.2, 146.6, 134.4, 134.0, 133.0, 132.9, 132.2, 130.9, 129.2, 127.6, 124.3.

IR (thin film): ν_{max} (cm^{-1}) = 2859, 2754, 1687, 1574, 1445, 1388, 1235, 1025, 917, 796, 734, 692, 665, 551.

HRMS (ESI, m/z) calcd. for $C_{14}H_8BrO_2^-$ [M-H] $^-$: 286.9713, found 286.9705.

[1,1':2',1''-terphenyl]-2,6-dicarbaldehyde (1q)

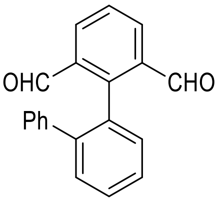

White solid, 87% yield, 585 mg; m.p. 153-154 °C.

^{1}H NMR (400 MHz, $CDCl_3$) δ 9.83 (d, J = 0.8 Hz, 2H), 8.09 (d, J = 7.7 Hz, 2H), 7.63 – 7.58 (m, 1H), 7.56 – 7.48 (m, 3H), 7.39 (dd, J = 7.5, 1.3 Hz, 1H), 7.16 – 7.12 (m, 3H), 6.98 – 6.95 (m, 2H).

^{13}C NMR (101 MHz, $CDCl_3$) δ 190.7, 147.6, 142.8, 139.6, 134.6, 132.6, 131.9, 131.0, 130.3, 129.7, 129.3, 128.4, 128.3, 127.4, 127.4.

IR (thin film): ν_{max} (cm^{-1}) = 3069, 2854, 2752, 1683, 1574, 1480, 1445, 1388, 1239, 919, 753, 701, 559.

HRMS (ESI, m/z) calcd. for $C_{20}H_{14}O_2Na^+$ [M+Na] $^+$: 309.0886, found 309.0881.

2-(naphthalen-1-yl)isophthalaldehyde (1r)

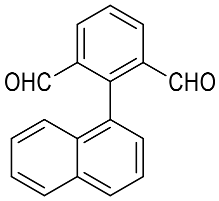

White solid, 90% yield, 550 mg; m.p. 55-56 °C.

^{1}H NMR (400 MHz, $CDCl_3$) δ 9.53 (d, J = 0.8 Hz, 2H), 8.35 (d, J = 7.7 Hz, 2H), 8.03 (dt, J = 8.4, 1.2 Hz, 1H), 8.01 – 7.96 (m, 1H), 7.76 (m, 1H), 7.61 (dd, J = 8.3, 7.0 Hz, 1H), 7.56 (m, 1H), 7.50 – 7.43 (m, 2H), 7.30 (m, 1H).

^{13}C NMR (101 MHz, $CDCl_3$) δ 190.8, 146.6, 135.6, 133.3, 133.2, 132.6, 130.1, 129.7, 129.4, 128.9, 128.7, 127.6, 126.8, 125.6, 124.9.

IR (thin film): ν_{max} (cm^{-1}) = 3060, 2874, 1679, 1566, 1450, 1380, 1240, 926, 805, 783, 717, 643, 570.

HRMS (ESI, m/z) calcd. for $C_{18}H_{12}O_2Na^+$ [M+Na] $^+$: 283.0729, found 283.0726.

2-(phenanthren-9-yl)isophthalaldehyde (1s)

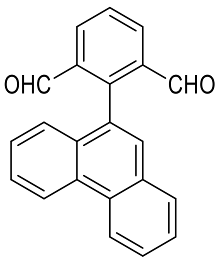

White solid, 82% yield, 600 mg; m.p. 132-134 °C.

^{1}H NMR (400 MHz, $CDCl_3$) δ 9.66 (d, J = 0.9 Hz, 2H), 8.87 – 8.75 (m, 2H), 8.38 (d, J = 7.8 Hz, 2H), 7.91 (dd, J = 8.0, 1.4 Hz, 1H), 7.82 – 7.66 (m, 5H), 7.55 (m, 1H), 7.36 (dd, J = 8.3, 1.3 Hz, 1H).

^{13}C NMR (101 MHz, $CDCl_3$) δ 190.8, 146.5, 135.7, 132.6, 132.3, 130.8, 130.6, 130.5, 130.2, 129.0, 128.9, 128.0, 127.8, 127.6, 127.6, 126.9, 123.4, 122.8.

IR (thin film): ν_{max} (cm^{-1}) = 2860, 2757, 1677, 1574, 1450, 1382, 1241, 927, 755, 714, 634.

HRMS (ESI, m/z) calcd. for $C_{22}H_{14}O_2Na^+$ $[M+Na]^+$: 333.0886, found 333.0881.

2-(pyren-1-yl)isophthalaldehyde (1t)

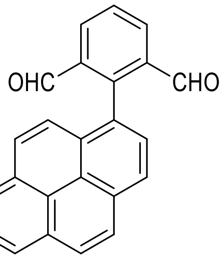

White solid, 30% yield, 300 mg; m.p. 133-134 °C.

^{1}H NMR (400 MHz, $CDCl_3$) δ 9.54 (s, 2H), 8.41 (d, J = 7.8 Hz, 2H), 8.30 (t, J = 6.2 Hz, 2H), 8.24 – 8.03 (m, 5H), 7.98 (d, J = 7.7 Hz, 1H), 7.81 (t, J = 7.8 Hz, 1H), 7.57 (d, J = 9.2 Hz, 1H).

^{13}C NMR (101 MHz, $CDCl_3$) δ 190.7, 147.1, 135.8, 132.7, 132.0, 131.4, 130.9, 130.7, 129.4, 129.0, 128.7, 128.7, 127.3, 126.8, 126.7, 126.3, 126.0, 124.5, 124.3, 124.3, 124.1.

IR (thin film): ν_{max} (cm^{-1}) = 3042, 2875, 2836, 2734, 1675, 1569, 1451, 1378, 1235, 921, 854, 707,605.

HRMS (ESI, m/z) calcd. for $C_{24}H_{15}O_2^+$ $[M+H]^+$: 335.1066, found 335.1064.

2-(2-methoxynaphthalen-1-yl)isophthalaldehyde (1u)

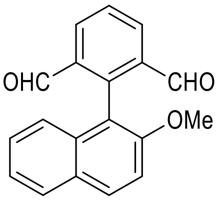

White solid, 23% yield, 160 mg; m.p. 101-102 °C.

^{1}H NMR (400 MHz, $CDCl_3$) δ 9.52 (d, J = 1.0 Hz, 2H), 8.36 (d, J = 7.6 Hz, 2H), 8.05 (dd, J = 9.2, 0.7 Hz, 1H), 7.92 – 7.85 (m, 1H), 7.73 (m, 1H), 7.44 – 7.33 (m, 3H), 7.13 – 7.06 (m, 1H), 3.84 (s, 3H).

^{13}C NMR (101 MHz, $CDCl_3$) δ 191.3, 154.9, 143.5, 135.5, 134.6, 132.7, 131.6, 128.6, 128.6, 128.4, 128.1, 124.3, 124.2, 114.0, 112.2, 56.2.

IR (thin film): ν_{max} (cm^{-1}) = 3076, 2962, 2952, 2761, 1680, 1617, 1508, 1458, 1371, 1275, 1124, 1060, 919, 806, 758, 700, 574.

HRMS (ESI, m/z) calcd. for $C_{19}H_{14}O_3Na^+$ [M+Na]$^+$: 313.0835, found 313.0833.

4-methoxy-2'-methyl-[1,1'-biphenyl]-2,6-dicarbaldehyde (1v)

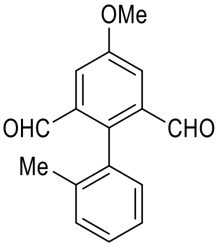

White solid, 45% yield, 270 mg; m.p. 105-106 °C.

^{1}H NMR (400 MHz, $CDCl_3$) δ 9.64 (s, 2H), 7.76 (s, 2H), 7.39 (td, *J* = 7.4, 1.6 Hz, 1H), 7.35 – 7.27 (m, 2H), 7.24 – 7.20 (m, 1H), 3.94 (s, 3H), 2.04 (s, 3H).

^{13}C NMR (101 MHz, $CDCl_3$) δ 190.7, 159.4, 140.6, 137.4, 135.9, 132.1, 131.2, 130.3, 129.2, 126.0, 117.6, 55.9, 20.5.

IR (thin film): ν_{max} (cm^{-1}) = 3076, 2944, 2838, 2740, 1676, 1601, 1470, 1386, 1307, 1280, 1210, 1144, 1054, 941, 875, 779, 748, 659, 590.

HRMS (ESI, m/z) calcd. for $C_{16}H_{14}O_3Na^+$ [M+Na]$^+$: 277.0835, found 277.0841.

4-fluoro-2'-methyl-[1,1'-biphenyl]-2,6-dicarbaldehyde (1w)

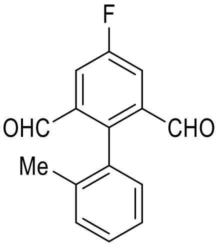

White solid, 70% yield, 400 mg; m.p. 104-105 °C.

^{1}H NMR (400 MHz, $CDCl_3$) δ 9.64 (d, *J* = 3.1 Hz, 2H), 7.93 (d, *J* = 8.0 Hz, 2H), 7.44 (td, *J* = 7.4, 1.5 Hz, 1H), 7.38 – 7.31 (m, 2H), 7.23 (dd, *J* = 7.5, 1.6 Hz, 1H), 2.06 (s, 3H).

^{13}C NMR (101 MHz, $CDCl_3$) δ 189.5 (d, *J* = 2.2 Hz), 162.4 (d, *J* = 252.1 Hz), 143.7 (d, *J* = 3.3 Hz), 137.1, 136.8 (d, *J* = 5.8 Hz), 131.3, 130.9, 130.5, 129.6, 126.2, 119.1 (d, *J* = 22.9 Hz), 20.4.

^{19}F NMR (377 MHz, $CDCl_3$) δ -110.70.

IR (thin film): ν_{max} (cm^{-1}) = 3064, 2866, 2759, 1672, 1600, 1463, 1394, 1299, 1196, 1119, 957, 894, 734, 661, 576.

HRMS (ESI, m/z) calcd. for $C_{15}H_{11}FO_2Na^+$ [M+Na]$^+$: 265.0635, found 265.0643.

2'-methyl-[1,1'-biphenyl]-2,6-dicarbonitrile (4a)

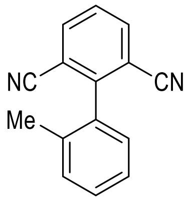

White solid, 15% yield, 3.3 mg; m.p. 130-131 ºC.

^{1}H NMR (400 MHz, $CDCl_3$) δ 7.96 (d, J = 7.9 Hz, 2H), 7.61 (t, J = 7.9 Hz, 1H), 7.44 (m, 1H), 7.41 – 7.32 (m, 2H), 7.21 (dd, J = 7.6, 1.4 Hz, 1H), 2.18 (s, 3H).

^{13}C NMR (101 MHz, $CDCl_3$) δ 149.4, 136.5, 135.7, 134.2, 130.9, 130.3, 129.0, 128.6, 126.4, 116.1, 115.2, 19.5.

IR (thin film): ν_{max} (cm^{-1}) = 3085, 3016, 2930, 2235, 1570, 1448, 1387, 1271, 1120, 812, 769, 522.

HRMS (ESI, m/z) calcd. for $C_{15}H_{10}N_2Na^+$ [M+Na]$^+$: 241.0736, found 241.0724.

(*S*)-6-formyl-2'-methyl-[1,1'-biphenyl]-2-carbonitrile (3a)

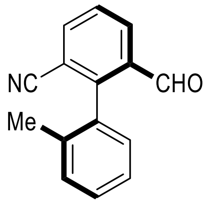

White solid, 63% yield, 14.0 mg; m.p. 91-93 ºC.

$[\alpha]^{20}_D$ = +12.66 (c = 0.1 in MeOH).

^{1}H NMR (400 MHz, $CDCl_3$) δ 9.67 (d, J = 0.8 Hz, 1H), 8.24 (dd, J = 7.9, 1.4 Hz, 1H), 7.98 (dd, J = 7.7, 1.4 Hz, 1H), 7.64 (m, 1H), 7.43 (m, 1H), 7.39 – 7.32 (m, 2H), 7.21 (dd, J = 7.6, 1.4 Hz, 1H), 2.11 (s, 3H).

^{13}C NMR (101 MHz, $CDCl_3$) δ 190.3, 148.8, 137.7, 136.3, 134.7, 133.1, 131.2, 130.7, 129.9, 129.8, 128.6, 126.3, 116.7, 115.0, 20.0.

IR (thin film): ν_{max} (cm^{-1}) = 3082, 2983, 2917, 2877, 2759, 2227, 1691, 1575, 1461, 1389, 1243, 802, 754, 560.

HRMS (ESI, m/z) calcd. for $C_{15}H_{10}NO^-$ [M-H]$^-$: 220.0767, found: 220.0774.

HPLC analysis (Shimadzu): >99:1 er (AS-H column, 40 °C, *n*-hexane / *i*-PrOH = 90 / 10, 0.5 mL / min, λ = 254 nm), Rt (major) = 19.1 min, Rt (minor) = 22.3 min.

(*S*)-6-formyl-2',3'-dimethyl-[1,1'-biphenyl]-2-carbonitrile (3b)

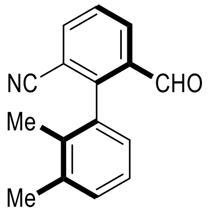

White solid, 61% yield, 14.3 mg; m.p. 101-103 ºC.

$[\alpha]^{20}_D$ = -51.98 (c = 0.1 in MeOH).

^{1}H NMR (400 MHz, $CDCl_3$) δ 9.67 (d, J = 0.8 Hz, 1H), 8.22 (dd, J = 7.9, 1.4 Hz, 1H), 7.97 (dd, J = 7.7, 1.4 Hz, 1H), 7.63 (m, 1H), 7.31

(d, $J = 7.5$ Hz, 1H), 7.23 (t, $J = 7.6$ Hz, 1H), 7.05 (dd, $J = 7.6, 1.4$ Hz, 1H), 2.37 (s, 3H), 2.00 (s, 3H).

^{13}C NMR (101 MHz, $CDCl_3$) δ 190.5, 149.5, 137.9, 137.6, 134.9, 134.8, 133.1, 131.3, 131.1, 128.4, 127.8, 125.8, 116.8, 115.1, 20.5, 16.8.

IR (thin film): ν_{max} (cm^{-1}) = 3084, 2983, 2922, 2876, 2760, 2229, 1691, 1570, 1461, 1389, 1234, 905, 806, 758, 586.

HRMS (ESI, m/z) calcd. for $C_{16}H_{12}NO^-$ [M-H]$^-$: 234.0924, found: 234.0923.

HPLC analysis (Shimadzu): >99:1 er (AS-H column, 40 °C, *n*-hexane / *i*-PrOH = 90 / 10, 0.5 mL / min, λ = 254 nm), Rt (major) = 17.5 min, Rt (minor) = 38.3 min.

(*S*)-6-formyl-2',4'-dimethyl-[1,1'-biphenyl]-2-carbonitrile (3c)

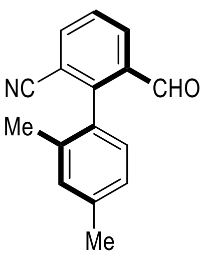

Yellow oil, 74% yield, 17.5 mg .

[α]$^{20}_{D}$ = -14.16 (*c* = 0.6 in MeOH).

^{1}H NMR (400 MHz, $CDCl_3$) δ 9.69 (d, $J = 0.9$ Hz, 1H), 8.22 (dd, $J = 7.9, 1.4$ Hz, 1H), 7.96 (dd, $J = 7.7, 1.4$ Hz, 1H), 7.62 (m, 1H), 7.19 – 7.12 (m, 2H), 7.09 (d, $J = 7.7$ Hz, 1H), 2.40 (s, 3H), 2.07 (s, 3H).

^{13}C NMR (101 MHz, $CDCl_3$) δ 190.6, 149.0, 139.7, 137.7, 136.0, 134.8, 131.4, 131.2, 130.1, 129.8, 128.5, 127.0, 116.9, 115.2, 21.3, 20.0.

IR (thin film): ν_{max} (cm^{-1}) =2927, 2865, 2740, 2235, 1702,1587, 1445, 1388, 1244, 916, 810, 754, 713, 572, 542.

HRMS (ESI, m/z) calcd. for $C_{16}H_{12}NO^-$ [M-H]$^-$: 234.0924, found: 234.0925.

HPLC analysis (Shimadzu): >99:1 er (AS-H column, 40 °C, *n*-hexane / *i*-PrOH = 90 / 10, 0.5 mL / min, λ = 254 nm), Rt (major) = 14.2 min, Rt (minor) = 16.3 min.

(*S*)-6-formyl-2',4'-dimethyl-[1,1'-biphenyl]-2-carbonitrile (3d)

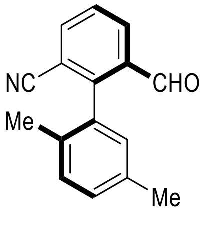

White oil, 75% yield, 17.6 mg .

[α]$^{20}_{D}$ = +5.12 (*c* = 0.5 in MeOH).

^{1}H NMR (400 MHz, $CDCl_3$) δ 9.68 (d, $J = 0.9$ Hz, 1H), 8.22 (dd, $J = 7.9, 1.4$ Hz, 1H), 7.97 (dd, $J = 7.7, 1.4$ Hz, 1H), 7.62 (m, 1H), 7.25 – 7.20 (m, 2H), 7.01 (d, $J = 1.7$ Hz, 1H), 2.37 (s, 3H), 2.06 (s, 3H).

^{13}C NMR (101 MHz, $CDCl_3$) δ 190.6, 149.1, 137.7, 135.8, 134.6, 133.1, 132.8, 131.1, 130.6, 130.5, 130.4, 128.5, 116.8, 115.0, 21.0, 19.5.

IR (thin film): ν_{max} (cm^{-1}) = 3014, 2926, 2868, 2748, 2235, 1691, 1585, 1498, 1444, 1388, 1243, 1138, 920, 822, 773, 599.

HRMS (ESI, m/z) calcd. for $C_{16}H_{12}NO^-$ $[M-H]^-$: 234.0924, found: 234.0930.

HPLC analysis (Shimadzu): >99:1 er (AS-H column, 40 °C, *n*-hexane / *i*-PrOH = 90 / 10, 0.5 mL / min, λ = 254 nm), Rt (major) = 16.2 min, Rt (minor) = 12.5 min.

(*S*)-6-formyl-2',4',5'-trimethyl-[1,1'-biphenyl]-2-carbonitrile (3e)

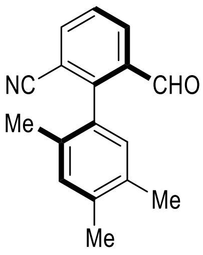

White solid, 75% yield, 17.7 mg; m.p. 68-69 °C.

$[\alpha]^{20}_D$ = +4.36 (c = 0.5 in MeOH).

^{1}H NMR (400 MHz, $CDCl_3$) δ 9.69 (d, J = 0.9 Hz, 1H), 8.21 (dd, J = 7.9, 1.4 Hz, 1H), 7.95 (dd, J = 7.7, 1.4 Hz, 1H), 7.61 (m, 1H), 7.12 (s, 1H), 6.95 (s, 1H), 2.30 (s, 3H), 2.27 (s, 3H), 2.04 (s, 3H).

^{13}C NMR (101 MHz, $CDCl_3$) δ 190.8, 149.2, 138.3, 137.7, 134.8, 134.4, 133.4, 131.9, 131.1, 131.0, 130.3, 128.3, 117.0, 115.1, 19.7, 19.4, 19.3.

IR (thin film): ν_{max} (cm^{-1}) = 3094, 2923, 2872, 2740, 2229, 1699, 1586, 1457, 1384, 1236, 1024, 921, 885, 807, 768, 559.

HRMS (ESI, m/z) calcd. for $C_{17}H_{14}NO^-$ $[M-H]^-$: 248.1080, found: 248.1085.

HPLC analysis (Shimadzu): >99:1 er (AS-H column, 40 °C, *n*-hexane / *i*-PrOH = 90 / 10, 0.5 mL / min, λ = 254 nm), Rt (major) = 16.3 min, Rt (minor) = 11.4 min.

(*S*)-6-formyl-4'-methoxy-2'-methyl-[1,1'-biphenyl]-2-carbonitrile (3f)

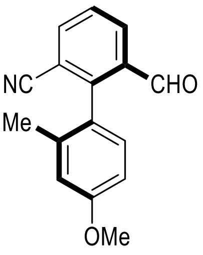

Green oil, 69% yield, 17.4 mg.

$[\alpha]^{20}_D$ = +20.50 (c = 0.2 in MeOH).

^{1}H NMR (400 MHz, $CDCl_3$) δ 9.70 (d, J = 0.8 Hz, 1H), 8.21 (dd, J = 7.9, 1.4 Hz, 1H), 7.96 (dd, J = 7.7, 1.4 Hz, 1H), 7.61 (td, J = 7.8, 0.8 Hz, 1H), 7.13 (d, J = 8.2 Hz, 1H), 6.92 – 6.83 (m, 2H), 3.86 (s, 3H), 2.08 (s, 3H).

^{13}C NMR (101 MHz, $CDCl_3$) δ 190.6, 160.5, 148.7, 137.9, 137.7, 135.1, 131.2, 131.2,

128.4, 125.2, 116.9, 116.0, 115.5, 111.7, 55.3, 20.3.

IR (thin film): ν_{max} (cm^{-1}) = 3075, 3012, 2957, 2922, 2861, 2744, 2233, 1698, 1611, 1387, 1299, 1243, 1125, 1051, 802, 759, 712, 568.

HRMS (ESI, m/z) calcd. for $C_{17}H_{14}NO^-$ [M-H]$^-$: 250.0873, found: 250.0877.

HPLC analysis (Shimadzu): 93:7 er (AS-H column, 40 °C, *n*-hexane / *i*-PrOH = 80 / 20, 0.5 mL / min, λ = 254 nm), Rt (major) = 20.0 min, Rt (minor) = 27.9 min.

(*S*)-4'-fluoro-6-formyl-2'-methyl-[1,1'-biphenyl]-2-carbonitrile (3g)

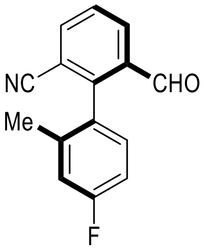

White solid, 53% yield, 12.6 mg; m.p. 132-133 °C.

$[\alpha]^{20}_D$ = -14.49 (c = 0.1 in MeOH).

^{1}H NMR (400 MHz, $CDCl_3$) δ 9.69 (d, J = 0.8 Hz, 1H), 8.24 (dd, J = 7.9, 1.4 Hz, 1H), 7.99 (dd, J = 7.7, 1.4 Hz, 1H), 7.66 (m, 1H), 7.20 (dd, J = 8.4, 5.6 Hz, 1H), 7.12 – 7.03 (m, 2H), 2.11 (s, 3H).

^{13}C NMR (101 MHz, $CDCl_3$) δ 190.0, 163.3 (d, J = 249.3 Hz), 147.6, 139.1 (d, J = 8.1 Hz), 137.8, 134.9, 131.6 (d, J = 9.2 Hz), 131.5, 129.0 (d, J = 3.2 Hz), 128.9, 117.6 (d, J = 21.7 Hz), 116.6, 115.3, 113.5 (d, J = 21.8 Hz), 20.2.

^{19}F NMR (377 MHz, $CDCl_3$) δ -111.70.

IR (thin film): ν_{max} (cm^{-1}) = 3074, 2928, 2847, 272, 2233, 1691, 1590, 1505, 1461, 1391, 1243, 1161, 949, 824, 806, 712, 574, 543.

HRMS (ESI, m/z) calcd. for $C_{15}H_9FNO^-$ [M-H]$^-$: 238.0673, found: 238.0679.

HPLC analysis (Shimadzu): 98:2 er (AS-H column, 40 °C, *n*-hexane / *i*-PrOH = 90 / 10, 0.5 mL / min, λ = 254 nm), Rt (major) = 22.3 min, Rt (minor) = 26.3 min.

(*S*)-5'-fluoro-6-formyl-2'-methyl-[1,1'-biphenyl]-2-carbonitrile (3h)

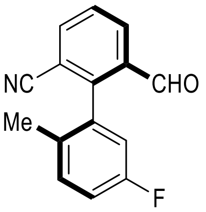

Yellow solid, 56% yield, 13.5 mg; m.p. 102-103 °C.

$[\alpha]^{20}_D$ = +9.33 (c = 0.1 in MeOH).

^{1}H NMR (400 MHz, $CDCl_3$) δ 9.69 (d, J = 0.9 Hz, 1H), 8.24 (dd, J = 8.0, 1.4 Hz, 1H), 7.99 (dd, J = 7.7, 1.4 Hz, 1H), 7.67 (m, 1H), 7.34 (dd, J = 8.6, 5.5 Hz, 1H), 7.14 (m, 1H), 6.96 (dd, J = 8.6, 2.7 Hz, 1H), 2.06 (s, 3H).

^{13}C NMR (101 MHz, $CDCl_3$) δ 189.7, 160.8 (d, J = 247.1 Hz), 147.3, 137.8, 134.6 (d,

J = 7.2 Hz), 134.5, 132.3 (d, *J* = 8.0 Hz), 132.1 (d, *J* = 3.5 Hz), 131.5, 129.1, 116.9 (d, *J* = 3.9 Hz), 116.7 (d, *J* = 5.6 Hz), 116.4, 114.8, 19.2.

^{19}F NMR (377 MHz, $CDCl_3$) δ -116.25.

IR (thin film): ν_{max} (cm^{-1}) = 3080, 2926, 2883, 2752, 2229, 1700, 1588, 1496, 1462, 1244, 1172, 1120, 891, 826, 767, 712, 610.

HRMS (ESI, m/z) calcd. for $C_{15}H_9FNO^-$ $[M-H]^-$: 238.0673, found: 238.0673.

HPLC analysis (Shimadzu): 97:3 er (AS-H column, 40 °C, *n*-hexane / *i*-PrOH = 90 / 10, 0.5 mL / min, λ = 254 nm), Rt (major) = 26.3 min, Rt (minor) = 22.6 min.

(*S*)-3'-chloro-6-formyl-2'-methyl-[1,1'-biphenyl]-2-carbonitrile (3i)

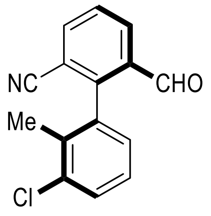

Yellow solid, 74% yield, 19.0 mg; m.p. 122-123 °C.

$[\alpha]^{20}_D$ = -24.41 (*c* = 0.4 in MeOH).

^{1}H NMR (400 MHz, $CDCl_3$) δ 9.68 (d, *J* = 0.8 Hz, 1H), 8.24 (dd, *J* = 7.9, 1.4 Hz, 1H), 7.99 (dd, *J* = 7.7, 1.4 Hz, 1H), 7.67 (td, *J* = 7.8, 0.9 Hz, 1H), 7.54 (dd, *J* = 8.0, 1.3 Hz, 1H), 7.33 – 7.27 (m, 1H), 7.14 (dd, *J* = 7.6, 1.3 Hz, 1H), 2.14 (s, 3H).

^{13}C NMR (101 MHz, $CDCl_3$) δ 189.8, 147.7, 137.8, 135.9, 134.9, 134.8, 134.6, 131.5, 130.8, 129.1, 128.5, 127.2, 116.5, 114.9, 17.7.

IR (thin film): ν_{max} (cm^{-1}) = 3083, 3012, 2876, 2751, 2231, 1694, 1560, 1433, 1387, 1236, 1037, 919, 786, 747, 674.

HRMS (ESI, m/z) calcd. for $C_{15}H_{10}ClNONa^+$ $[M+Na]^+$: 278.0343, found: 278.0337.

HPLC analysis (Shimadzu): 99:1 er (AS-H column, 40 °C, *n*-hexane / *i*-PrOH = 90 / 10, 0.5 mL / min, λ = 254 nm), Rt (major) = 14.3 min, Rt (minor) = 18.3 min.

(*S*)-4'-chloro-6-formyl-2'-methyl-[1,1'-biphenyl]-2-carbonitrile (3j)

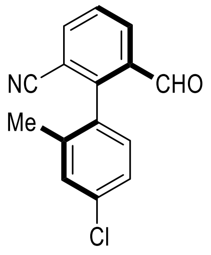

Yellow solid, 61% yield, 15.5 mg; m.p. 77-78 °C.

$[\alpha]^{20}_D$ = +45.33 (*c* = 0.1 in MeOH).

^{1}H NMR (400 MHz, $CDCl_3$) δ 9.68 (d, *J* = 0.8 Hz, 1H), 8.24 (dd, *J* = 7.9, 1.4 Hz, 1H), 7.99 (dd, *J* = 7.7, 1.4 Hz, 1H), 7.67 (td, *J* = 7.8, 0.8 Hz, 1H), 7.40 – 7.32 (m, 2H), 7.16 (d, *J* = 8.1 Hz, 1H), 2.09 (s,

3H).

^{13}C NMR (101 MHz, $CDCl_3$) δ 189.8, 147.4, 138.3, 137.8, 135.8, 134.7, 131.6, 131.6, 131.1, 130.7, 129.0, 126.6, 116.5, 115.0, 19.9.

IR (thin film): ν_{max} (cm^{-1}) = 3068, 2919, 2873, 2752, 2230, 1698, 1575, 1450, 1437, 1236, 1104, 916, 874, 811, 660.

HRMS (ESI, m/z) calcd. for $C_{15}H_{10}ClNO^+$ $[M+H]^+$: 256.0523, found: 254.0531.

HPLC analysis (Shimadzu): 98:2 er (AS-H column, 40 ºC, *n*-hexane / *i*-PrOH = 90 / 10, 0.5 mL / min, λ = 254 nm), Rt (major) = 22.2 min, Rt (minor) = 24.9 min.

(*S*)-5'-chloro-6-formyl-2'-methyl-[1,1'-biphenyl]-2-carbonitrile (3k)

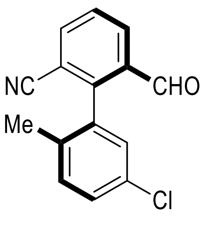

Brown oil, 52% yield, 13.2 mg.

$[\alpha]^{20}_D$ = -5.38 (*c* = 0.3 in MeOH).

^{1}H NMR (400 MHz, $CDCl_3$) δ 9.69 (s, 1H), 8.25 (dd, *J* = 7.8, 1.5 Hz, 1H), 8.00 (dd, *J* = 7.8, 1.4 Hz, 1H), 7.68 (t, *J* = 7.8 Hz, 1H), 7.41 (dd, *J* = 8.3, 2.3 Hz, 1H), 7.32 (d, *J* = 8.3 Hz, 1H), 7.22 (d, *J* = 2.2 Hz, 1H), 2.07 (s, 3H).

^{13}C NMR (101 MHz, $CDCl_3$) δ 189.6, 147.0, 137.8, 134.9, 134.7, 134.6, 132.1, 132.0, 131.6, 130.0, 129.6, 129.1, 116.3, 114.9, 19.4.

IR (thin film): ν_{max} (cm^{-1}) = 3067, 2855, 2758, 2235, 1679, 1571, 1497, 1449, 1387, 916, 803, 704, 633.

HRMS (ESI, m/z) calcd. for $C_{15}H_{11}ClNO^+$ $[M+H]^+$: 256.0524, found: 256.0519.

HPLC analysis (Shimadzu): 91:9 er (AS-H column, 40 ºC, *n*-hexane / *i*-PrOH = 90 / 10, 0.5 mL / min, λ = 254 nm), Rt (major) = 25.5 min, Rt (minor) = 21.9 min.

(*S*)-2'-ethyl-6-formyl-[1,1'-biphenyl]-2-carbonitrile (3l)

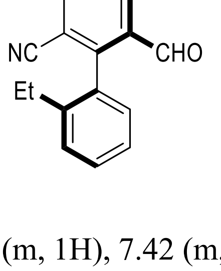

Green oil, 54% yield, 12.6 mg.

$[\alpha]^{20}_D$ = +6.83 (*c* = 0.1 in MeOH).

^{1}H NMR (400 MHz, $CDCl_3$) δ 9.68 (d, *J* = 0.9 Hz, 1H), 8.23 (dd, *J* = 7.9, 1.4 Hz, 1H), 7.98 (dd, *J* = 7.7, 1.4 Hz, 1H), 7.64 (m, 1H), 7.48 (m, 1H), 7.42 (m, 1H), 7.34 (m, 1H), 7.19 (dd, *J* = 7.5, 1.4 Hz, 1H), 2.39 (m, 2H), 1.06 (t, *J* = 7.6 Hz, 3H).

^{13}C NMR (101 MHz, $CDCl_3$) δ 190.4, 148.7, 142.3, 137.6, 134.8, 132.5, 131.1, 130.1, 129.9, 128.9, 128.6, 126.2, 116.7, 115.3, 26.4, 14.8.

IR (thin film): ν_{max} (cm^{-1}) = 3076, 2975, 2932, 2870, 2230, 1691, 1460, 1385, 1236, 1064, 954, 916, 809, 763, 717, 549.

HRMS (ESI, m/z) calcd. for $C_{16}H_{11}NO^-$ $[M-H]^-$: 234.0924, found: 234.0931.

HPLC analysis (Shimadzu): 99:1 er (AS-H column, 40 °C, *n*-hexane / *i*-PrOH = 90 / 10, 0.5 mL / min, λ = 254 nm), Rt (major) = 20.8 min, Rt (minor) = 26.8 min.

(*S*)-6-formyl-2'-(methylthio)-[1,1'-biphenyl]-2-carbonitrile (3m)

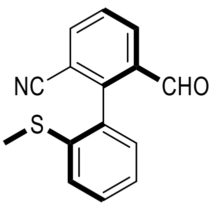

White solid, 71% yield, 18 mg; m.p. 87-89 °C.

$[\alpha]^{20}_D$ = +50.49 (*c* = 1.0 in $CHCl_3$)

^{1}H NMR (400 MHz, $CDCl_3$) δ 9.64 (d, *J* = 0.8 Hz, 1H), 8.23 (dt, *J* = 7.9, 1.1 Hz, 1H), 7.99 (dd, *J* = 7.7, 1.4 Hz, 1H), 7.65 (m, 1H), 7.51 (m, 1H), 7.38 – 7.30 (m, 2H), 7.25 (dd, *J* = 7.5, 1.6 Hz, 1H), 2.40 (d, *J* = 0.9 Hz, 3H).

^{13}C NMR (101 MHz, $CDCl_3$) δ 190.1, 146.9, 138.5, 137.9, 134.9, 131.9, 131.0, 130.4, 130.3, 129.1, 125.5, 125.3, 116.7, 115.2, 77.4, 77.1, 76.8, 15.7.

IR (thin film): ν_{max} (cm^{-1}) = 3083, 2924, 2860, 2752, 228, 1698, 1567, 1427, 1389, 1242, 1073, 1041, 914, 806, 753, 680, 552.

HRMS (ESI, m/z) calcd. for $C_{15}H_{11}NOSNa^+$ $[M+Na]^+$: 276.0453, found: 276.0453.

HPLC analysis (Shimadzu): 97:3 er (ID column, 40 °C, *n*-hexane / *i*-PrOH = 95 / 5, 0.5 mL / min, λ = 254 nm), Rt (major) = 25.1 min, Rt (minor) = 23.9 min.

(*S*)-6-formyl-2'-vinyl-[1,1'-biphenyl]-2-carbonitrile (3n)

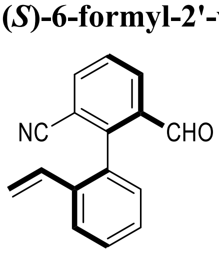

White oil, 49% yield, 11.5 mg.

$[\alpha]^{20}_D$ = 19.05 (*c* = 0.3 in MeOH).

^{1}H NMR (400 MHz, $CDCl_3$) δ 9.63 (s, 1H), 8.23 (dd, *J* = 7.9, 1.4 Hz, 1H), 7.99 (dd, *J* = 7.6, 1.3 Hz, 1H), 7.72 (d, *J* = 7.9 Hz, 1H), 7.65 (t, *J* = 7.8 Hz, 1H), 7.52 (m, 1H), 7.43 (m, 1H), 7.28 (dd, *J* = 7.6, 1.3 Hz, 1H), 6.25 (dd, *J* = 17.4, 11.0 Hz, 1H), 5.69 (d, *J* = 17.4 Hz, 1H), 5.22 (d, *J* = 11.0 Hz, 1H).

^{13}C NMR (101 MHz, $CDCl_3$) δ 190.1, 147.7, 137.7, 137.3, 135.0, 133.5, 131.8, 131.2,

130.3, 130.1, 128.8, 128.1, 126.0, 117.9, 116.7, 115.2.

IR (thin film): ν_{max} (cm^{-1}) = 3062, 2874, 2225, 1698, 1574, 152, 1386, 1242, 922, 805, 759, 741, 719.

HRMS (ESI, m/z) calcd. for $C_{16}H_{11}NONa^+$ [M+Na] $^+$: 256.0732, found: 256.0740.

UPLC analysis: 98:2 er (OD-3 column, 25 °C, *n*-hexane / *i*-PrOH = 90 / 10, 0.5 mL / min, λ = 254 nm), Rt (major) = 7.1 min, Rt (minor) = 7.6 min.

(*S*)-2'-chloro-6-formyl-[1,1'-biphenyl]-2-carbonitrile (3o)

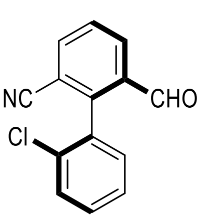

White solid, 46% yield, 11.0 mg; m.p. 72-74 °C.

$[\alpha]^{20}_{D}$ = +81.66 (*c* = 0.1 in MeOH).

^{1}H NMR (400 MHz, $CDCl_3$) δ 9.71 (d, *J* = 0.9 Hz, 1H), 8.25 (dd, *J* = 7.9, 1.4 Hz, 1H), 8.00 (dd, *J* = 7.7, 1.4 Hz, 1H), 7.69 (m, 1H), 7.63 – 7.56 (m, 1H), 7.53 – 7.43 (m, 2H), 7.41 – 7.35 (m, 1H).

^{13}C NMR (101 MHz, $CDCl_3$) δ 189.5, 145.7, 137.7, 134.7, 133.6, 132.6, 131.5, 131.4, 131.3, 130.1, 129.3, 127.4, 116.5, 115.1.

IR (thin film): ν_{max} (cm^{-1}) = 2966, 2923, 2844, 2277, 1694, 1574, 1388, 1258, 1242, 1093, 1038, 809, 752, 685.

HRMS (ESI, m/z) calcd. for $C_{14}H_7ClNO^-$ [M-H] $^-$: 240.0221, found: 240.0212.

HPLC analysis (Shimadzu): 92:8 er (OJ-H column, 40 °C, *n*-hexane / *i*-PrOH = 90 / 10, 0.5 mL / min, λ = 254 nm), Rt (major) = 33.8 min, Rt (minor) = 31.1 min.

(*S*)-2'-bromo-6-formyl-[1,1'-biphenyl]-2-carbonitrile (3p)

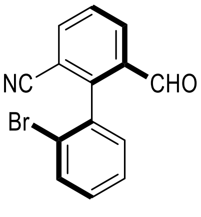

White solid, 47% yield, 13.5 mg; m.p. 114-115 °C.

$[\alpha]^{20}_{D}$ = -6.49 (c = 0.1 in MeOH).

^{1}H NMR (400 MHz, $CDCl_3$) δ 9.71 (d, *J* = 0.8 Hz, 1H), 8.25 (dd, *J* = 7.9, 1.4 Hz, 1H), 8.00 (dd, *J* = 7.7, 1.4 Hz, 1H), 7.77 (dd, *J* = 8.0, 1.2 Hz, 1H), 7.69 (m, 1H), 7.51 (m, 1H), 7.45 – 7.41 (m, 1H), 7.41 – 7.36 (m, 1H).

^{13}C NMR (101 MHz, $CDCl_3$) δ 189.5, 147.4, 137.6, 134.7, 134.5, 133.2, 131.4, 131.4, 131.3, 129.3, 127.9, 123.5, 116.4, 115.0.

IR (thin film): ν_{max} (cm^{-1}) = 3131, 2792, 2483, 2135, 1822, 1482, 1157, 810, 535.

HRMS (ESI, m/z) calcd. for $C_{14}H_9ClNO^+$ [M+H] $^+$: 285.9862, found: 285.9861.

HPLC analysis (Shimadzu): 94:6 er (OJ-H column, 40 ºC, *n*-hexane / *i*-PrOH = 90 / 10, 0.5 mL / min, λ = 254 nm), Rt (major) = 36.1 min, Rt (minor) = 33.8 min.

(*S*)-6-formyl-[1,1':2',1''-terphenyl]-2-carbonitrile (3q)

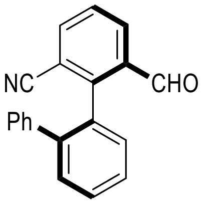

White solid, 35% yield, 10 mg; m.p. 135-137 ºC.

[α]$^{20}_{D}$ = -6.49 (*c* = 1.0 in $CHCl_3$).

^{1}H NMR (400 MHz, $CDCl_3$) δ 9.72 (d, *J* = 0.9 Hz, 1H), 8.01 (dd, *J* = 7.9, 1.4 Hz, 1H), 7.85 (dd, *J* = 7.7, 1.4 Hz, 1H), 7.64 – 7.59 (m, 1H), 7.57 – 7.52 (m, 2H), 7.49 (m, 1H), 7.45 – 7.41 (m, 1H), 7.20 – 7.14 (m, 3H), 7.08 – 7.02 (m, 2H).

^{13}C NMR (101 MHz, $CDCl_3$) δ 189.8, 148.5, 142.4, 139.5, 137.5, 134.5, 132.0, 131.1, 130.8, 130.5, 130.1, 129.5, 128.3, 128.3, 127.8, 127.3, 117.1, 115.4.

IR (thin film): ν_{max} (cm^{-1}) = 3056, 2919, 2848, 2746, 2233, 1685, 1568, 1478, 1449, 1393, 1233, 1070, 959, 912, 752, 700, 559.

HRMS (ESI, m/z) calcd. for $C_{20}H_{13}NONa^+$ [M+Na]$^+$: 306.0889, found: 306.0893.

HPLC analysis (Shimadzu): 94:6 er (OD-H column, 40 ºC, *n*-hexane / *i*-PrOH = 95 / 5, 0.5 mL / min, λ = 254 nm), Rt (major) = 25.1 min, Rt (minor) = 23.8 min.

(*S*)-3-formyl-2-(naphthalen-1-yl)benzonitrile (3r)

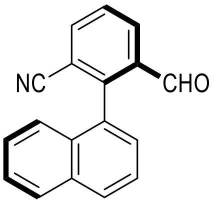

Yellow solid, 48% yield, 12.4. mg; m.p. 118-119 ºC.

[α]$^{20}_{D}$ = -31.38 (*c* = 0.3 in MeOH).

^{1}H NMR (400 MHz, $CDCl_3$) δ 9.50 (d, *J* = 0.9 Hz, 1H), 8.32 (dd, *J* = 7.9, 1.4 Hz, 1H), 8.05 (m, 2H), 7.98 (dt, *J* = 8.2, 0.9 Hz, 1H), 7.73 (m, 1H), 7.63 (dd, *J* = 8.3, 7.0 Hz, 1H), 7.55 (m, 1H), 7.52 – 7.44 (m, 2H), 7.28 – 7.24 (m, 1H).

^{13}C NMR (101 MHz, $CDCl_3$) δ 190.1, 147.4, 137.8, 135.7, 133.5, 132.3, 131.1, 130.9, 130.3, 129.0, 128.8, 128.6, 127.6, 126.7, 125.2, 124.7, 116.7, 115.8.

IR (thin film): ν_{max} (cm^{-1}) = 3051, 2849, 2747, 2237, 1693, 1574, 1508, 1389, 1333, 1236, 1217, 962, 800, 770,679.

HRMS (ESI, m/z) calcd. for $C_{18}H_{11}NONa^+$ [M+Na]$^+$: 280.0732, found: 280.0731.

HPLC analysis (Shimadzu): 99:1 er (AS-H column, 40 ºC, *n*-hexane / *i*-PrOH = 80 / 20, 0.5 mL / min, λ = 254 nm), Rt (major) = 18.5 min, Rt (minor) = 25.6 min.

(*S*)-3-formyl-2-(phenanthren-9-yl)benzonitrile (3s)

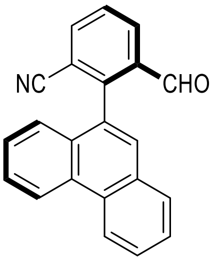

Yellow oil, 62% yield, 19.0 mg; m.p. 78-79 ºC.

$[\alpha]^{20}_D$ = 36.83 (*c* = 0.6 in MeOH).

^{1}H NMR (400 MHz, $CDCl_3$) δ 9.61 (d, *J* = 0.8 Hz, 1H), 8.80 – 8.76 (m, 1H), 8.35 (dd, *J* = 7.9, 1.4 Hz, 1H), 8.08 (dd, *J* = 7.7, 1.4 Hz, 1H), 7.94 (dd, *J* = 7.9, 1.4 Hz, 1H), 7.82 – 7.65 (m, 5H), 7.55 (m, 1H), 7.30 (dd, *J* = 8.3, 1.3 Hz, 1H).

^{13}C NMR (101 MHz, $CDCl_3$) δ 190.1, 147.3, 137.9, 135.9, 131.2, 131.1, 130.9, 130.6, 130.5, 130.1, 129.8, 129.2, 129.1, 128.0, 127.6, 127.5, 127.4, 125.9, 123.5, 122.8, 116.7, 115.9.

IR (thin film): ν_{max} (cm^{-1}) = 3073, 2858, 2232, 1698, 1565, 1448, 1381, 1241, 919, 804, 771, 742,612.

HRMS (ESI, m/z) calcd. for $C_{22}H_{13}NONa^+$ [M+Na] $^+$: 330.0889, found: 330.0884.

HPLC analysis (Shimadzu): 97:3 er (IB column, 40 ºC, *n*-hexane / *i*-PrOH = 90 / 10, 0.5 mL / min, λ = 254 nm), Rt (major) = 30.5 min, Rt (minor) = 42.0 min.

(*S*)-3-formyl-2-(pyren-1-yl)benzonitrile (3t)

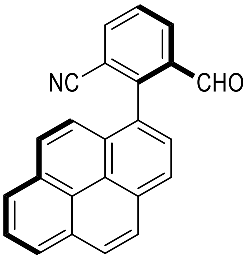

Brown solid, 42% yield,14.0 mg; m.p. 185-186 ºC.

$[\alpha]^{20}_D$ = -16.55 (*c* = 0.4 in MeOH).

^{1}H NMR (400 MHz, $CDCl_3$) δ 9.51 (s, 1H), 8.37 (m, 1H), 8.34 (d, *J* = 7.8 Hz, 1H), 8.31 – 8.27 (m, 1H), 8.25 – 8.17 (m, 3H), 8.12 – 8.07 (m, 3H), 8.00 (d, *J* = 7.8 Hz, 1H), 7.81 – 7.72 (m, 1H), 7.54 (dt, *J* = 9.2, 1.2 Hz, 1H).

^{13}C NMR (101 MHz, $CDCl_3$) δ 190.1, 147.9, 137.8, 135.9, 132.4, 131.4, 131.2, 130.7, 130.1, 129.4, 129.0, 128.8, 127.8, 127.6, 127.3, 126.6, 126.3, 125.9, 124.7, 124.7, 124.5, 123.5, 116.8, 116.1.

IR (thin film): ν_{max} (cm^{-1}) = 3052, 2847, 2739, 2236, 1696, 1581, 1387, 1241, 854, 802,

724, 701, 538.

HRMS (ESI, m/z) calcd. for $C_{24}H_{13}NONa^+$ [M+Na] $^+$: 330.0889, found: 330.0883.

HPLC analysis (Shimadzu): 97:3 er (IB column, 40 ºC, *n*-hexane / *i*-PrOH = 90 / 10, 0.5 mL / min, λ = 254 nm), Rt (major) = 27.1 min, Rt (minor) = 23.9 min.

(*S*)-3-formyl-2-(2-methoxynaphthalen-1-yl)benzonitrile (3u)

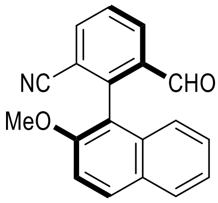

Yellow solid, 46% yield, 13.2 mg; m.p. 158-160 ºC.

$[\alpha]^{20}_D$ = 8.07 (*c* = 0.3 in MeOH).

^{1}H NMR (400 MHz, $CDCl_3$) δ 9.50 (d, *J* = 1.0 Hz, 1H), 8.32 (dd, *J* = 7.9, 1.4 Hz, 1H), 8.08 – 8.02 (m, 2H), 7.89 (m, 1H), 7.70 (td, *J* = 7.8, 0.9 Hz, 1H), 7.43 (d, *J* = 9.1 Hz, 1H), 7.41 – 7.36 (m, 2H), 7.09 – 7.04 (m, 1H), 3.90 (s, 3H).

^{13}C NMR (101 MHz, $CDCl_3$) δ 190.7, 154.7, 144.4, 138.0, 135.9, 133.5, 132.1, 131.1, 128.7, 128.6, 128.5, 128.0, 124.2, 123.4, 117.0, 116.6, 115.2, 112.7, 56.4.

IR (thin film): ν_{max} (cm^{-1}) = 3080, 2936, 2842, 2227, 1696, 1594, 1509, 1461, 1372, 1257, 1143, 1062, 1018, 901, 808, 755, 699, 581.

HRMS (ESI, m/z) calcd. for $C_{19}H_{13}NO_2Na^+$ [M+Na]$^+$: 310.0838, found 310.0840.

HPLC analysis (Waters): 93:7 er (AS-H column, 25 ºC, *n*-hexane / *i*-PrOH = 80 / 20, 0.5 mL / min, λ = 254 nm), Rt (major) = 31.0 min, Rt (minor) = 35.8 min.

(*S*)-6-formyl-4-methoxy-2'-methyl-[1,1'-biphenyl]-2-carbonitrile (3v)

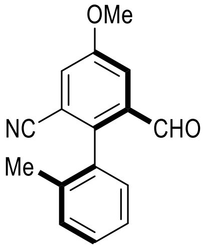

White solid, 75% yield, 19.0 mg; m.p. 118-119 ºC.

$[\alpha]^{20}_D$ = -24.07 (*c* = 0.5 in MeOH).

^{1}H NMR (400 MHz, $CDCl_3$) δ 9.61 (s, 1H), 7.71 (d, *J* = 2.9 Hz, 1H), 7.49 (d, *J* = 2.9 Hz, 1H), 7.41 (td, *J* = 7.3, 1.4 Hz, 1H), 7.37 – 7.28 (m, 2H), 7.19 (dd, *J* = 7.5, 1.6 Hz, 1H), 3.94 (s, 3H), 2.10 (s, 3H).

^{13}C NMR (101 MHz, $CDCl_3$) δ 190.3, 159.1, 141.1, 136.8, 135.7, 132.9, 130.6, 130.4, 129.6, 126.2, 124.4, 116.5, 115.8, 115.0, 56.1, 20.0.

IR (thin film): ν_{max} (cm^{-1}) = 2928, 2858, 2590, 2229, 1699, 1596, 1471, 1327, 1277, 1221, 1150, 1059, 953, 879, 739, 625.

HRMS (ESI, m/z) calcd. for $C_{16}H_{13}NO_2Na^+$ [M+Na]$^+$: 274.0838, found 274.0844.

HPLC analysis (Waters): >99:1 er (AS-H column, 25 °C, *n*-hexane / *i*-PrOH = 90 / 10, 0.5 mL / min, λ = 254 nm), Rt (major) = 20.9 min, Rt (minor) = 25.2 min.

(*S*)-4-fluoro-6-formyl-2'-methyl-[1,1'-biphenyl]-2-carbonitrile (3w)

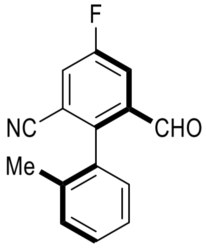

Yellow oil, 60% yield, 14.4 mg.

[α]$^{20}_{D}$ = 39.20 (c = 0.3 in MeOH).

^{1}H NMR (400 MHz, $CDCl_3$) δ 9.60 (d, J = 3.1 Hz, 1H), 7.92 (dd, J = 8.2, 2.8 Hz, 1H), 7.69 (dd, J = 7.3, 2.8 Hz, 1H), 7.45 (td, J = 7.5, 1.5 Hz, 1H), 7.40 – 7.32 (m, 2H), 7.20 (dd, J = 7.6, 1.6 Hz, 1H), 2.11 (s, 3H).

^{13}C NMR (101 MHz, $CDCl_3$) δ 189.0, 161.5 (d, J = 253.6 Hz), 144.8 (d, J = 4.4 Hz), 136.7 (d, J = 6.5 Hz), 136.5, 132.1, 130.8, 130.1, 130.1, 126.4, 124.7 (d, J = 25.4 Hz), 118.3 (d, J = 21.8 Hz), 116.6 (d, J = 8.7 Hz), 115.5, 115.5, 20.0.

IR (thin film): ν_{max} (cm^{-1}) = 3067, 2926, 2861, 2740, 2242, 1694, 1593, 1453, 1385, 1296, 1198, 1120, 998, 890, 734, 612, 575.

^{19}F NMR (377 MHz, $CDCl_3$) δ -109.65.

HRMS (ESI, m/z) calcd. for $C_{15}H_9NO^-$ [M-H]$^-$: 238.0673, found 238.0678.

HPLC analysis (Waters): >99:1 er (AS-H column, 25 °C, *n*-hexane / *i*-PrOH = 90 / 10, 0.5 mL / min, λ = 254 nm), Rt (major) = 16.1 min, Rt (minor) = 17.7 min.

(*R*)-2'-methyl-6-vinyl-[1,1'-biphenyl]-2-carbonitrile (5)

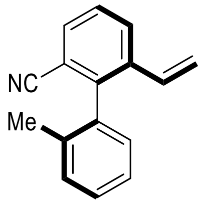

White oil, 80% yield, 17.5 mg.

[α]$^{25}_{D}$ = +36.24 (c = 0.2 in MeOH).

^{1}H NMR (400 MHz, $CDCl_3$) δ 7.87 (dd, J = 8.0, 1.3 Hz, 1H), 7.66 (dd, J = 7.7, 1.3 Hz, 1H), 7.45 (t, J = 7.8 Hz, 1H), 7.40 – 7.27 (m, 3H), 7.12 (dd, J = 7.5, 1.4 Hz, 1H), 6.33 (dd, J = 17.5, 11.0 Hz, 1H), 5.71 (d, J = 17.5 Hz, 1H), 5.21 (d, J = 11.0 Hz, 1H), 2.04 (s, 3H).

^{13}C NMR (101 MHz, $CDCl_3$) δ 143.9, 137.5, 136.3, 136.2, 133.5, 131.9, 130.3, 129.4, 129.1, 128.9, 128.0, 126.1, 117.9, 116.9, 113.8, 19.6.

IR (thin film): ν_{max} (cm^{-1}) = 3073, 3023, 2925, 2849, 2230, 1730, 1632, 1458, 1408,

1268, 1123, 994, 916, 817, 753, 727.

HRMS (ESI, m/z) calcd. for $C_{16}H_{13}NNa^+$ [M+Na] $^+$: 242.0940, found: 242.0942.

HPLC analysis (Waters): >99:1 er (OD-H column, 25 °C, *n*-hexane / *i*-PrOH = 98 / 2, 0.5 mL / min, λ = 254 nm), Rt (major) = 11.9 min, Rt (minor) = 12.6 min.

(*R*)-2'-methyl-6-vinyl-[1,1'-biphenyl]-2-carbaldehyde (6)

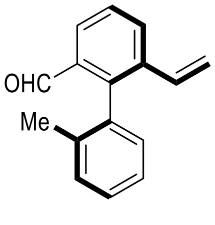

Yellow oil, 86% yield, 19.2 mg.

$[\alpha]^{20}_D$ = +62.16 (*c* = 0.2 in MeOH).

^{1}H NMR (400 MHz, $CDCl_3$) δ 9.61 (d, *J* = 0.8 Hz, 1H), 7.92 (m, 2H), 7.50 (m, 1H), 7.39 – 7.27 (m, 3H), 7.12 (dd, *J* = 7.4, 1.4 Hz, 1H), 6.33 (dd, *J* = 17.5, 11.0 Hz, 1H), 5.70 (dd, *J* = 17.5, 1.0 Hz, 1H), 5.17 (dd, *J* = 11.0, 1.0 Hz, 1H), 1.99 (s, 3H).

^{13}C NMR (101 MHz, $CDCl_3$) δ 192.5, 143.8, 137.3, 136.8, 135.2, 134.2, 133.6, 130.5, 130.3, 130.1, 128.4, 128.0, 126.5, 125.8, 116.1, 20.2.

IR (thin film): ν_{max} (cm^{-1}) = 3065, 3013, 2929, 2858, 2747, 1689, 1627, 1588, 1457, 1388, 1250, 1224, 910, 814, 809, 769, 750, 735, 645.

HRMS (ESI, m/z) calcd. for $C_{16}H_{14}NNa^+$ [M+Na] $^+$: 245.0936, found: 245.0937.

HPLC analysis (Waters): >99:1 er (AS-H column, 25 °C, *n*-hexane / *i*-PrOH = 90 / 10, 0.5 mL / min, λ = 254 nm), Rt (major) = 15.8 min, Rt (minor) = 14.2 min.

(*S*)-6-(hydroxymethyl)-2'-methyl-[1,1'-biphenyl]-2-carbonitrile (7)

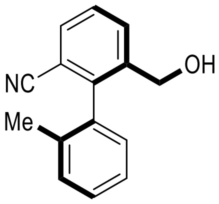

Green oil, 94% yield, 21.0 mg.

$[\alpha]^{20}_D$ = -22.66 (*c* = 0.5 in MeOH)

^{1}H NMR (400 MHz, $CDCl_3$) δ 7.83 (d, *J* = 7.8 Hz, 1H), 7.67 (dd, *J* = 7.8, 1.3 Hz, 1H), 7.49 (t, *J* = 7.7 Hz, 1H), 7.38 – 7.27 (m, 3H), 7.09 (dd, *J* = 7.7, 1.3 Hz, 1H), 4.42 – 4.30 (m, 2H), 2.05 (s, 3H), 1.91 (s, 1H).

^{13}C NMR (101 MHz, $CDCl_3$) δ 143.6, 140.3, 135.8, 135.7, 131.6, 131.6, 130.6, 129.1, 128.8, 128.2, 126.3, 117.8, 113.3, 62.2, 19.6.

IR (thin film): ν_{max} (cm^{-1}) = 3068, 3018, 2927, 2865, 2232, 1455, 1282, 1229, 1124, 1055, 908, 805, 745, 726, 686, 628.

HRMS (ESI, m/z) calcd. for $C_{15}H_{13}NONa^+$ [M+Na] $^+$: 246.0889, found: 246.0888.

HPLC analysis (Shimadzu): >99:1 er (OH-H column, 40 °C, *n*-hexane / *i*-PrOH = 90 / 10, 0.5 mL / min, λ = 254 nm), Rt (major) = 28.4 min, Rt (minor) = 24.3 min.

(*S*)-6-(chloromethyl)-2'-methyl-[1,1'-biphenyl]-2-carbonitrile (8)

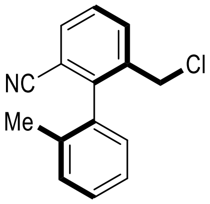

Green solid, 70% yield, 17.0 mg; m.p. 53-54 °C.

$[\alpha]^{20}_D$ = -23.49 (*c* = 0.1 in MeOH).

^{1}H NMR (400 MHz, $CDCl_3$) δ 7.81 (dd, *J* = 7.9, 1.3 Hz, 1H), 7.72 (dd, *J* = 7.8, 1.3 Hz, 1H), 7.51 (t, *J* = 7.8 Hz, 1H), 7.43 – 7.28 (m, 3H), 7.15 (dd, *J* = 7.5, 1.4 Hz, 1H), 4.38 – 4.23 (m, 2H), 2.09 (s, 3H).

^{13}C NMR (101 MHz, $CDCl_3$) δ 144.9, 137.3, 136.0, 135.2, 134.2, 132.8, 130.6, 129.3, 129.1, 128.5, 126.2, 117.4, 114.1, 43.0, 19.8.

IR (thin film): ν_{max} (cm^{-1}) = 2924, 2862, 2234, 1578, 1452, 1272, 1172, 1119, 817, 773, 691, 603, 600, 548.

HRMS (ESI, m/z) calcd. for $C_{15}H_{12}ClNNa^+$ [M+Na] $^+$: 264.0550, found: 264.0550.

HPLC analysis (Waters): >99:1 er (AS-H column, 25 °C, *n*-hexane / *i*-PrOH = 95 / 5, 0.5 mL / min, λ = 254 nm), Rt (major) = 17.4 min, Rt (minor) = 16.4 min.

(*S*)-6-cyano-2'-methyl-[1,1'-biphenyl]-2-carboxylic acid (9)

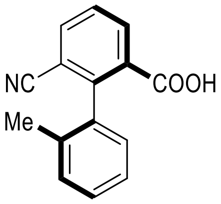

White solid, 84% yield, 20.0 mg; m.p. 192-193 °C.

$[\alpha]^{20}_D$ = -10.38 (*c* = 0.3 in MeOH).

^{1}H NMR (400 MHz, CD_3OD) δ 8.19 (dd, *J* = 7.9, 1.4 Hz, 1H), 7.97 (dd, *J* = 7.8, 1.4 Hz, 1H), 7.64 (t, *J* = 7.8 Hz, 1H), 7.34 – 7.27 (m, 2H), 7.26 – 7.21 (m, 1H), 7.06 (dd, *J* = 7.4, 1.3 Hz, 1H), 2.08 (s, 3H).

^{13}C NMR (101 MHz, CD_3OD) δ 171.6, 149.5, 141.5, 139.5, 139.3, 137.7, 137.2, 133.4, 132.2, 132.0, 129.2, 120.7, 118.5, 22.5.

IR (thin film): ν_{max} (cm^{-1}) = 2923, 2858, 2232, 1683, 1577, 1461, 1424, 1401, 1301, 1189, 1153, 1117, 924, 826, 752, 714, 645, 621, 515.

HRMS (ESI, m/z) calcd. for $C_{15}H_{11}NO_2Na^+$ [M+Na] $^+$: 260.0682, found: 260.0682.

HPLC analysis (Shimadzu): >99:1 er (AD-H column, 40 °C, *n*-hexane / *i*-PrOH = 80

/ 20, 0.5 mL / min, λ = 254 nm), Rt (major) = 13.4 min, Rt (minor) = 17.6 min.

XI. ^{1}H NMR, ^{13}C NMR, ^{19}F NMR, and HPLC/UPLC spectra

2'-methyl-[1,1'-biphenyl]-2,6-dicarbaldehyde (1a)

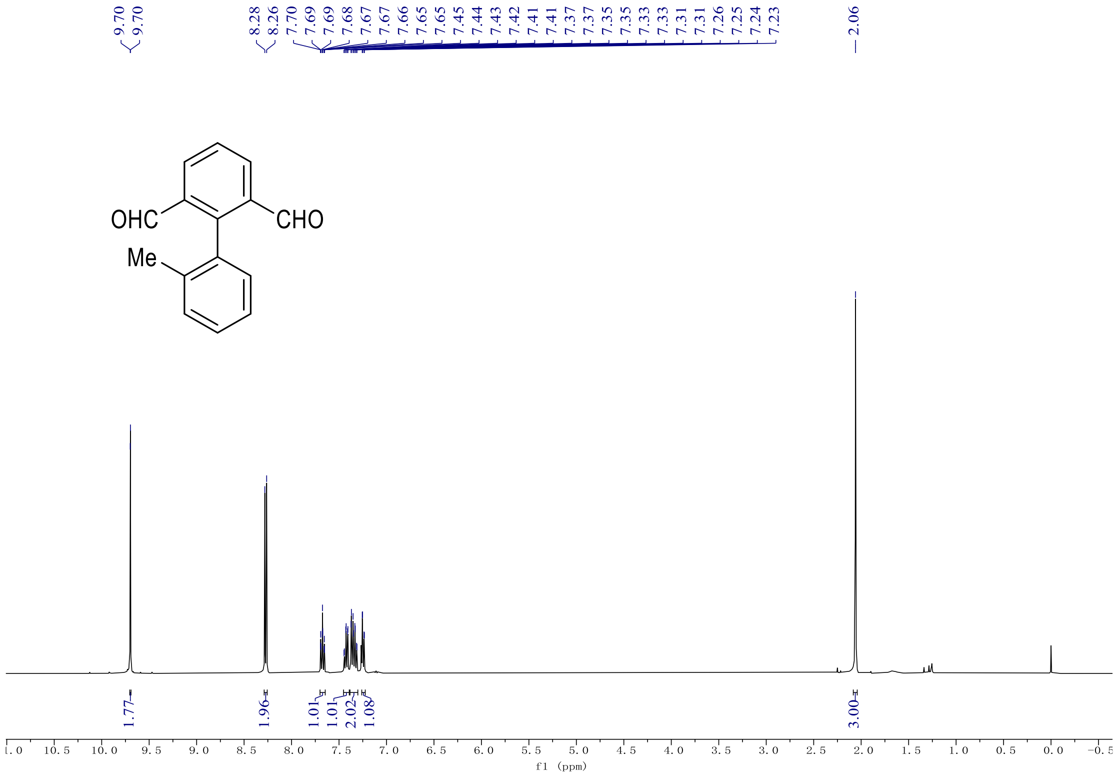

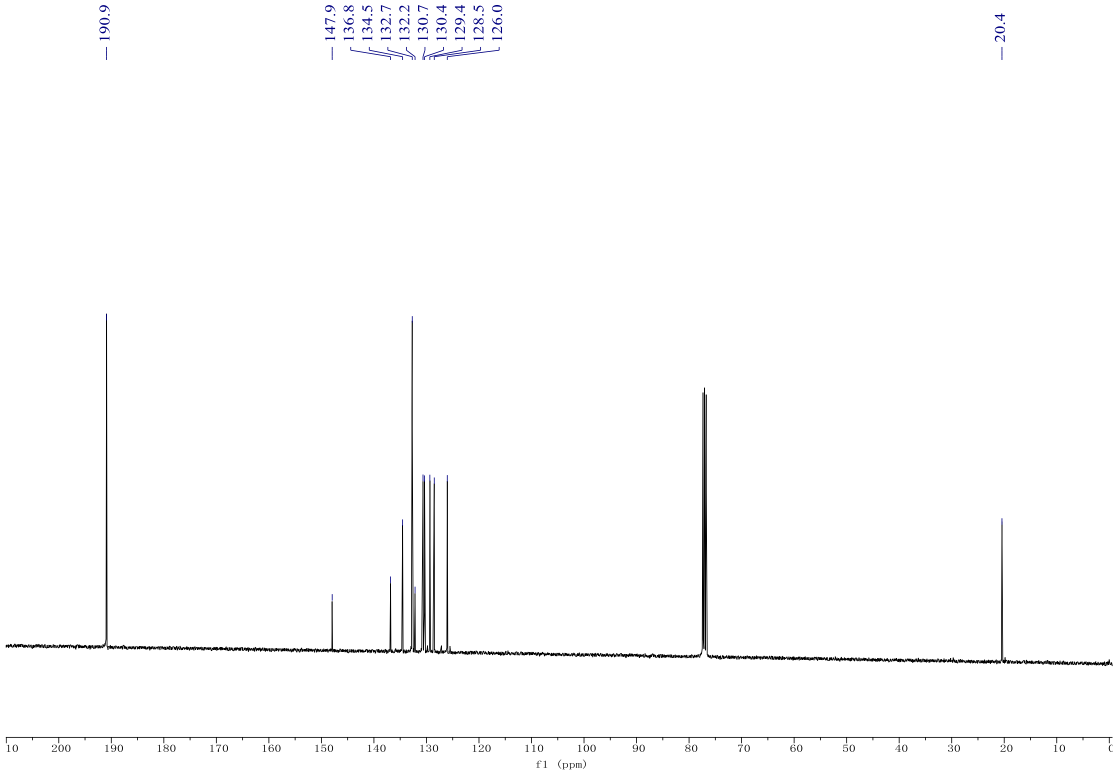

2',3'-dimethyl-[1,1'-biphenyl]-2,6-dicarbaldehyde (1b)

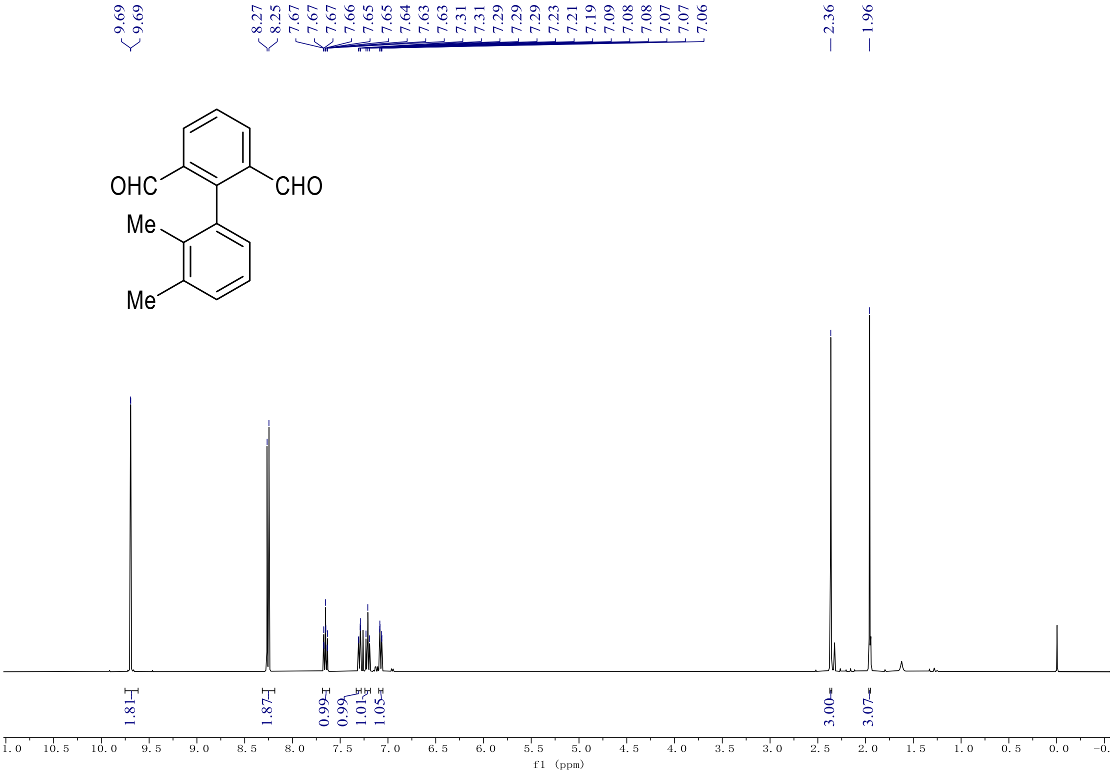

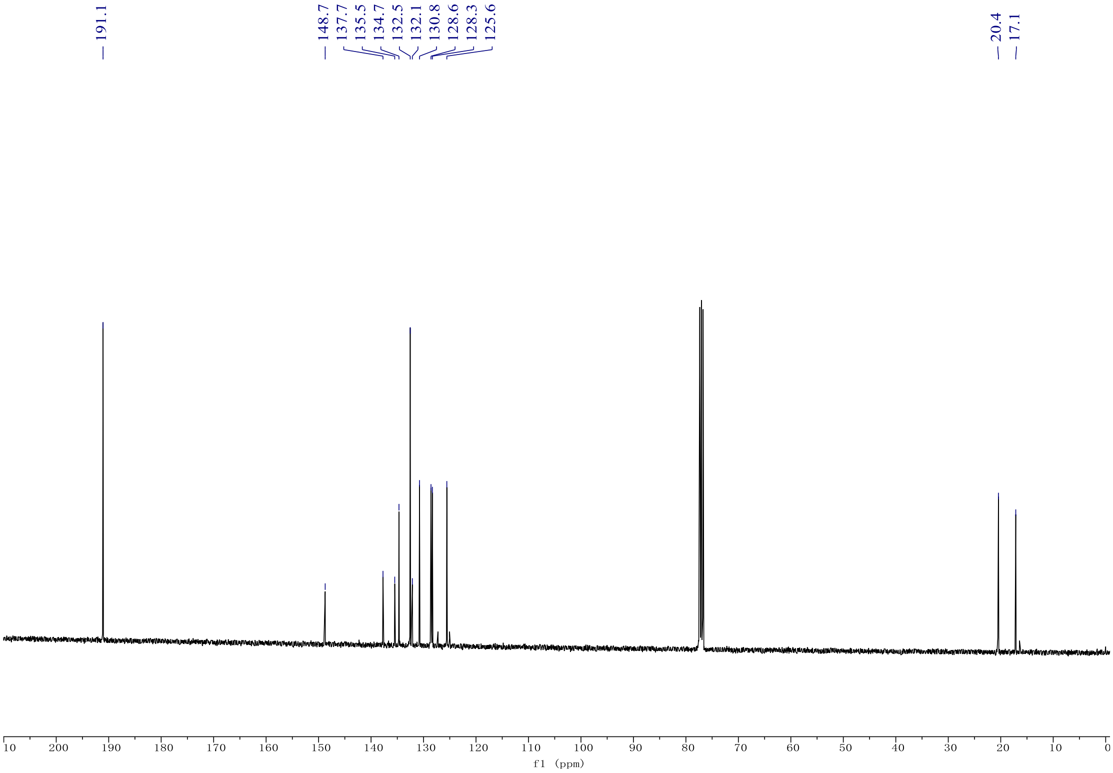

2',4'-dimethyl-[1,1'-biphenyl]-2,6-dicarbaldehyde (1c)

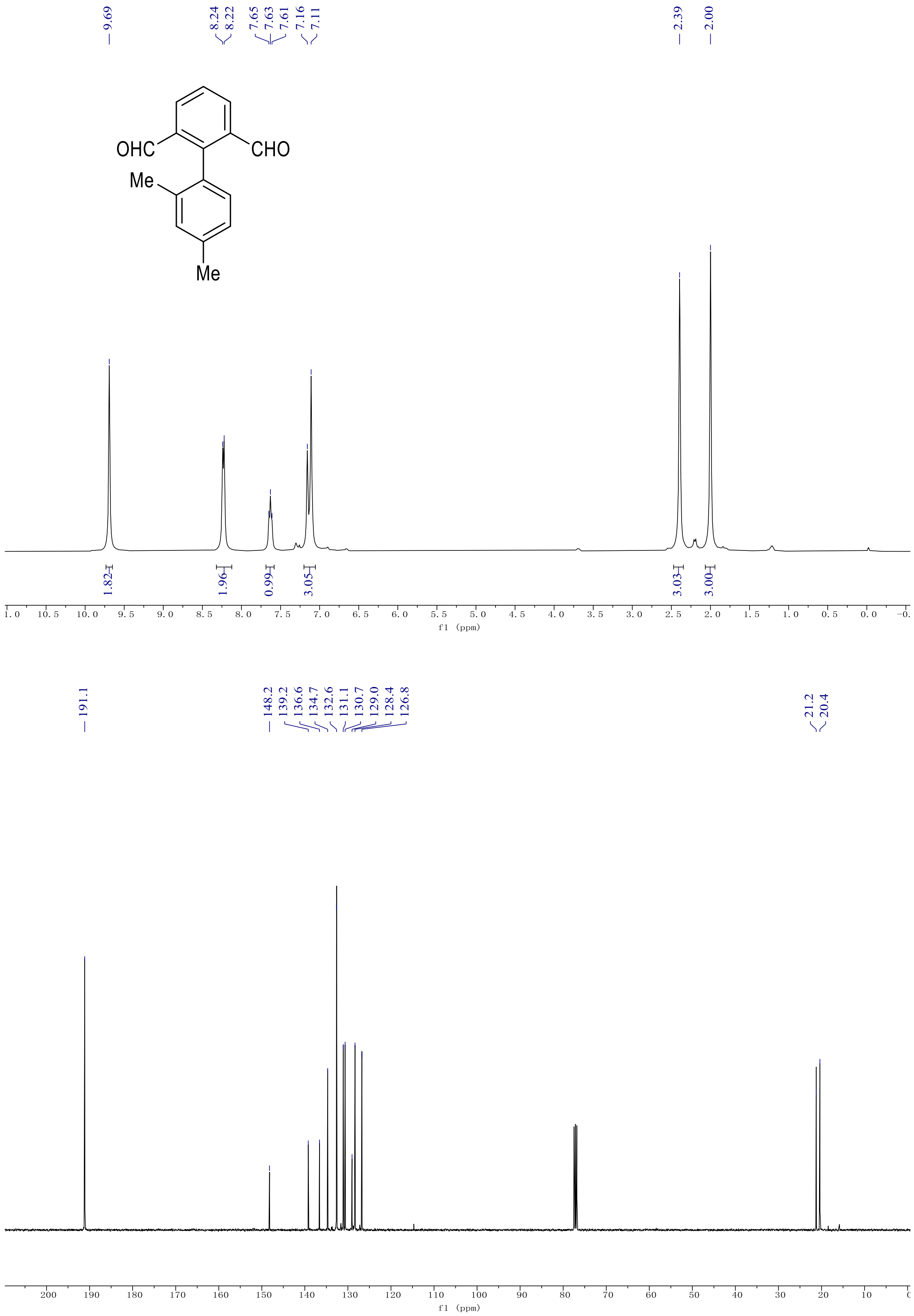

2',5'-dimethyl-[1,1'-biphenyl]-2,6-dicarbaldehyde (1d)

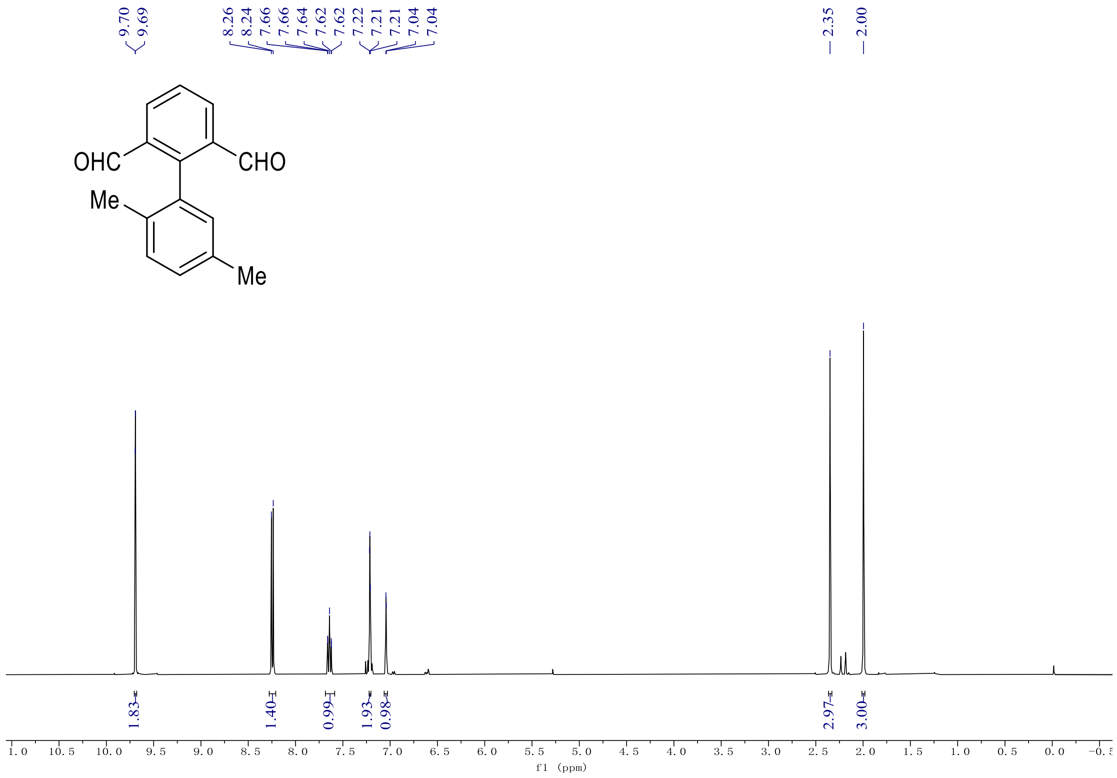

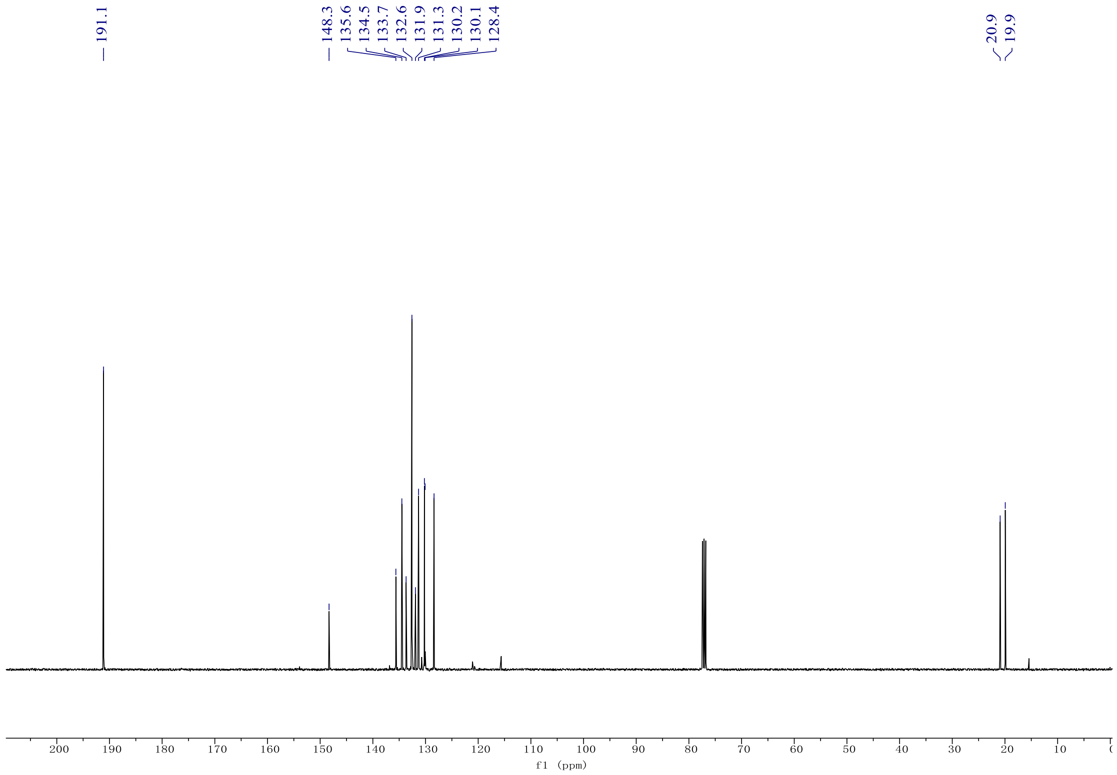

2',4',5'-trimethyl-[1,1'-biphenyl]-2,6-dicarbaldehyde (1e)

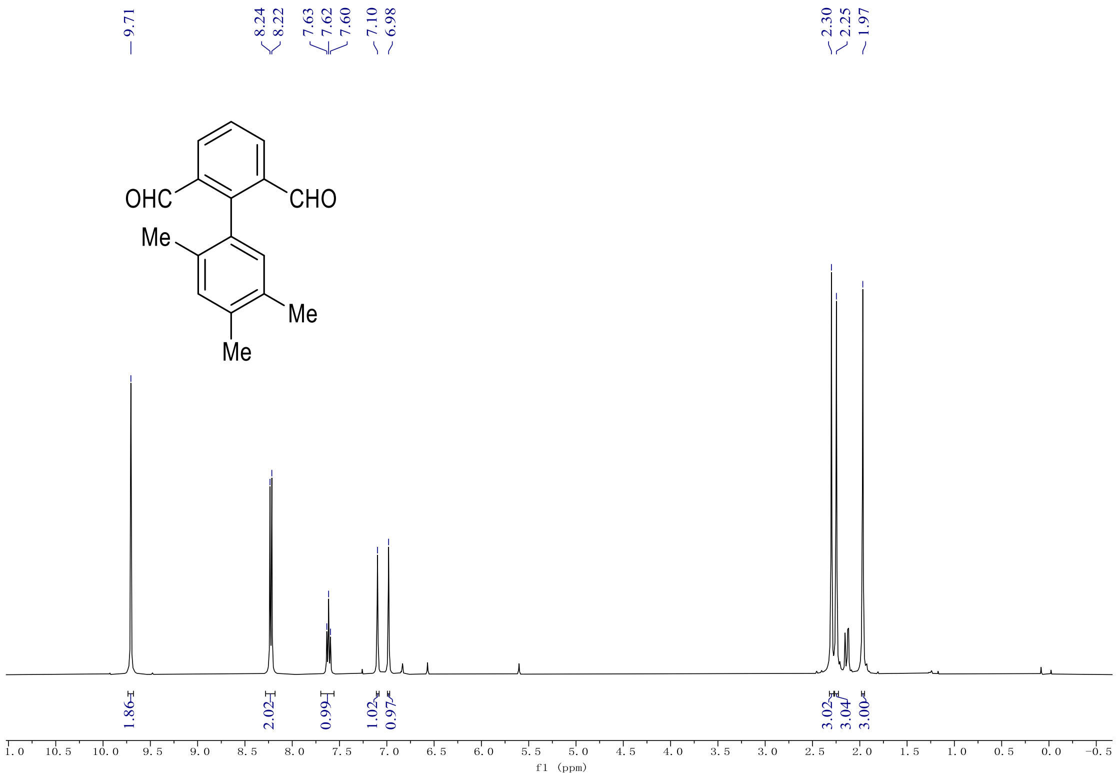

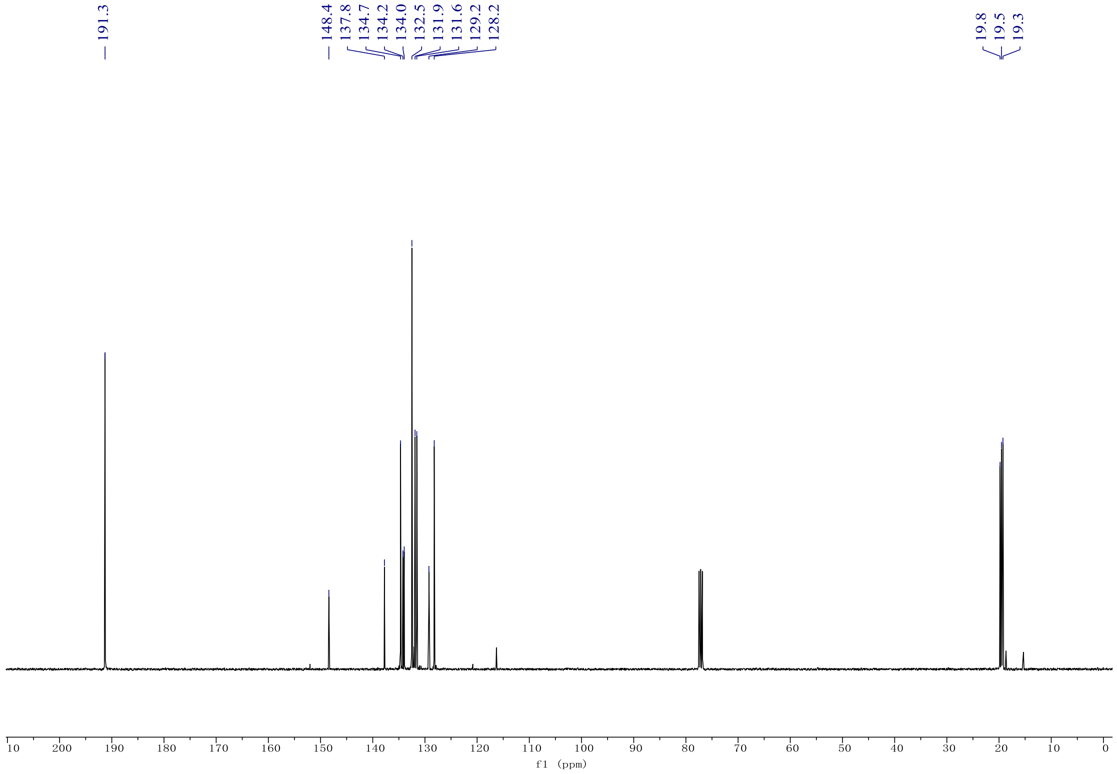

4'-methoxy-2'-methyl-[1,1'-biphenyl]-2,6-dicarbaldehyde (1f)

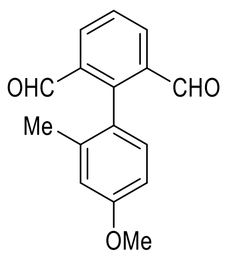

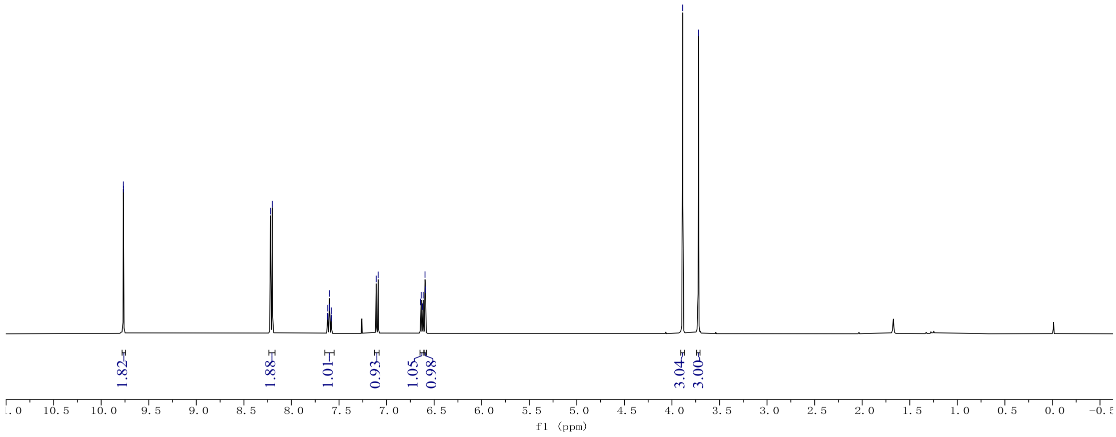

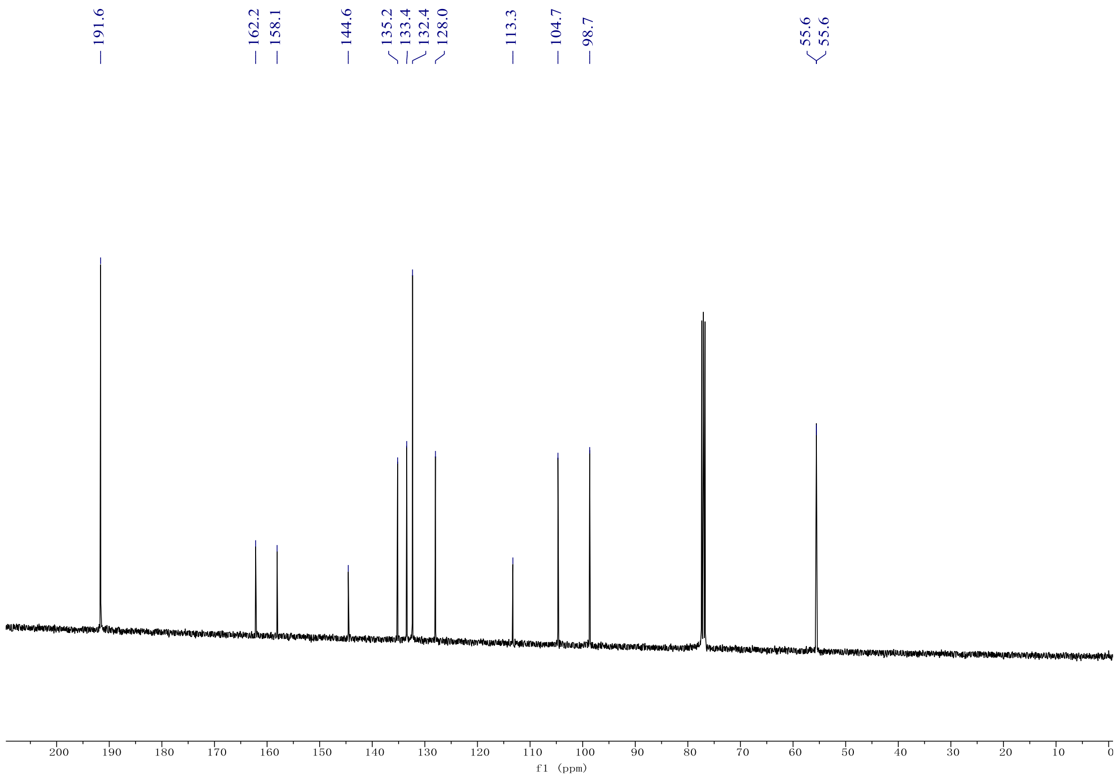

4'-fluoro-2'-methyl-[1,1'-biphenyl]-2,6-dicarbaldehyde (1g)

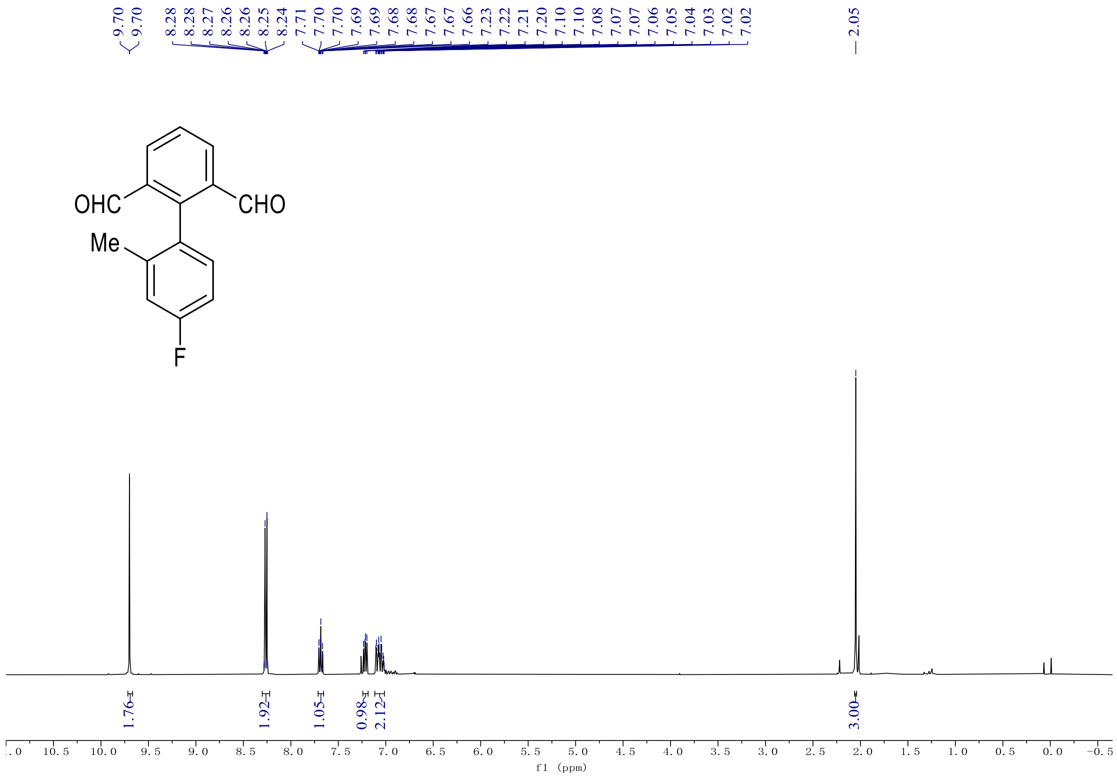

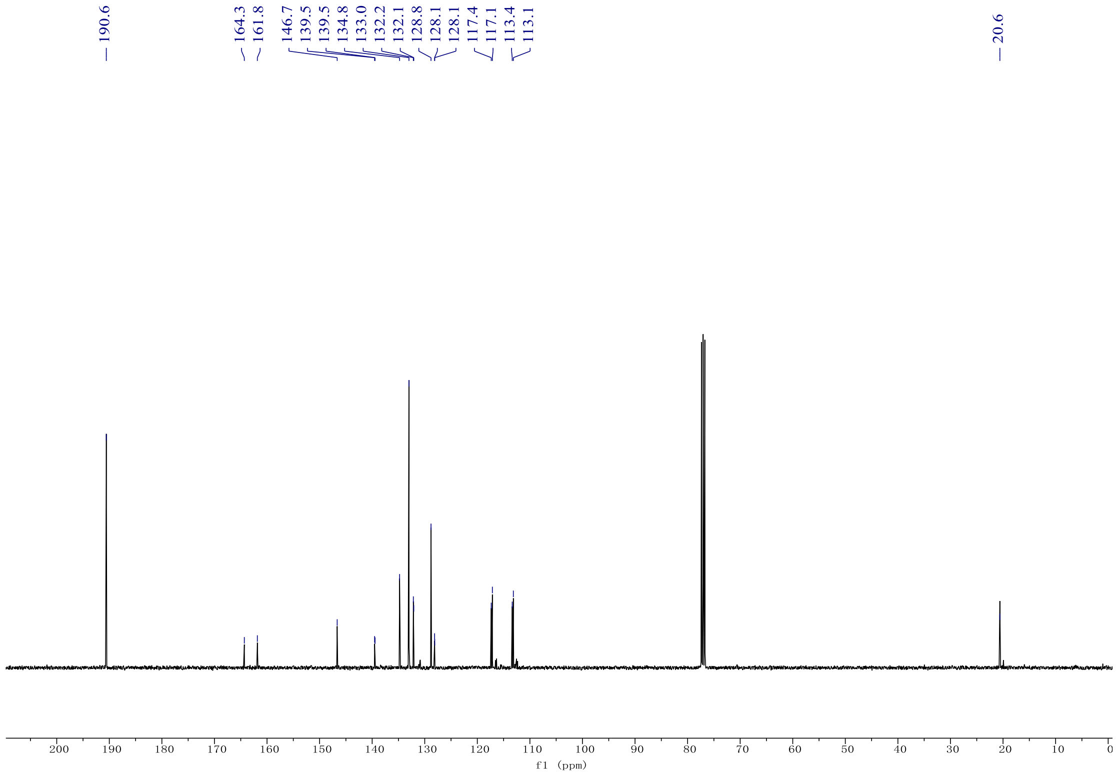

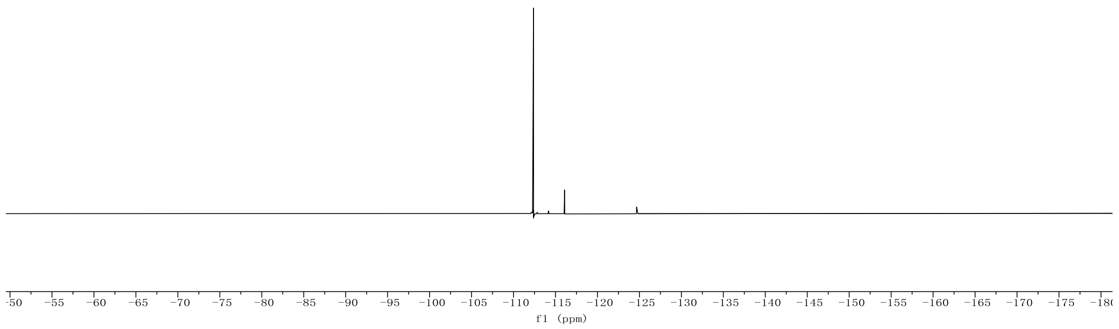

-50 -55 -60 -65 -70 -75 -80 -85 -90 -95 -100 -105 -110 -115 -120 -125 -130 -135 -140 -145 -150 -155 -160 -165 -170 -175
f1 (ppm)

5'-fluoro-2'-methyl-[1,1'-biphenyl]-2,6-dicarbaldehyde (1h)

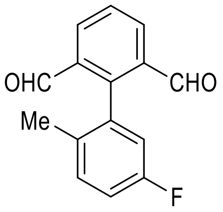

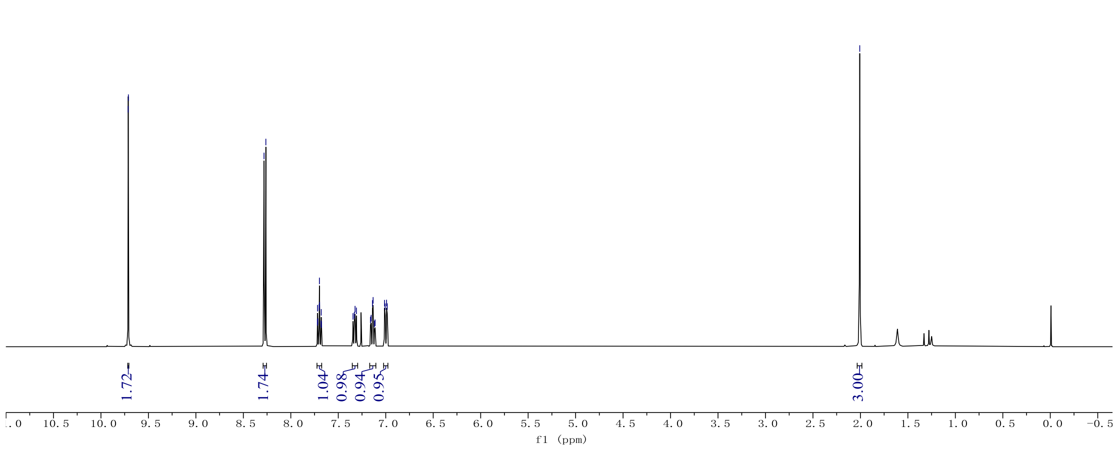

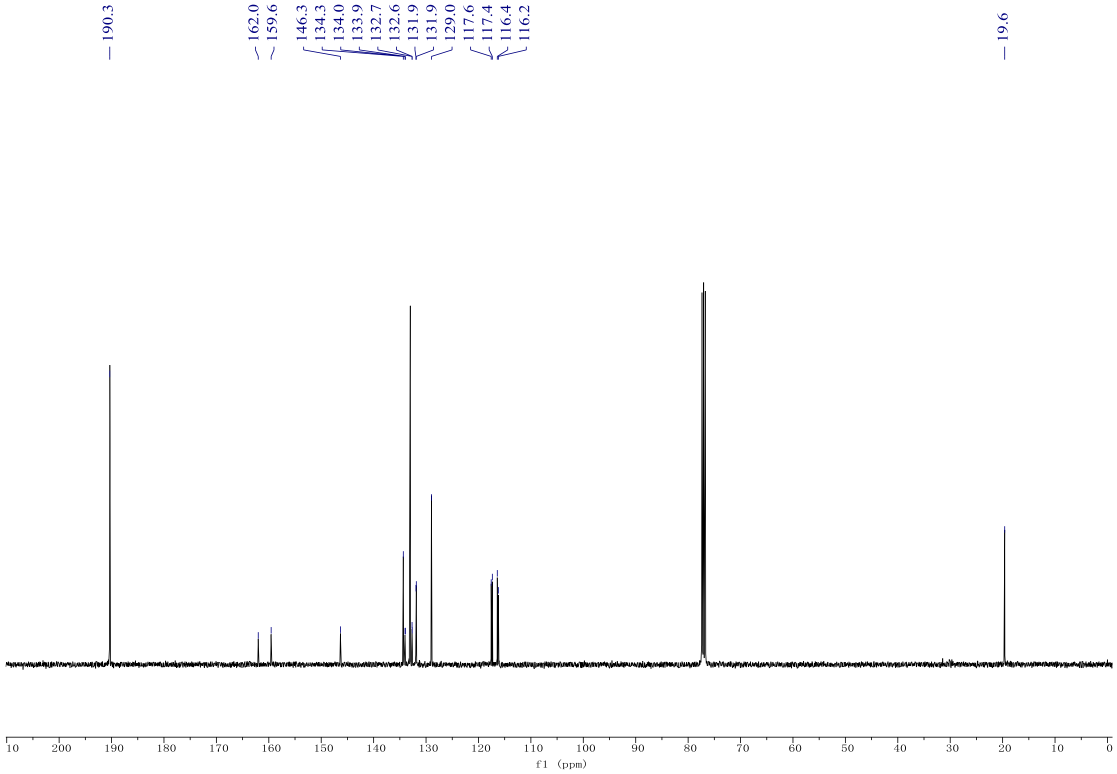

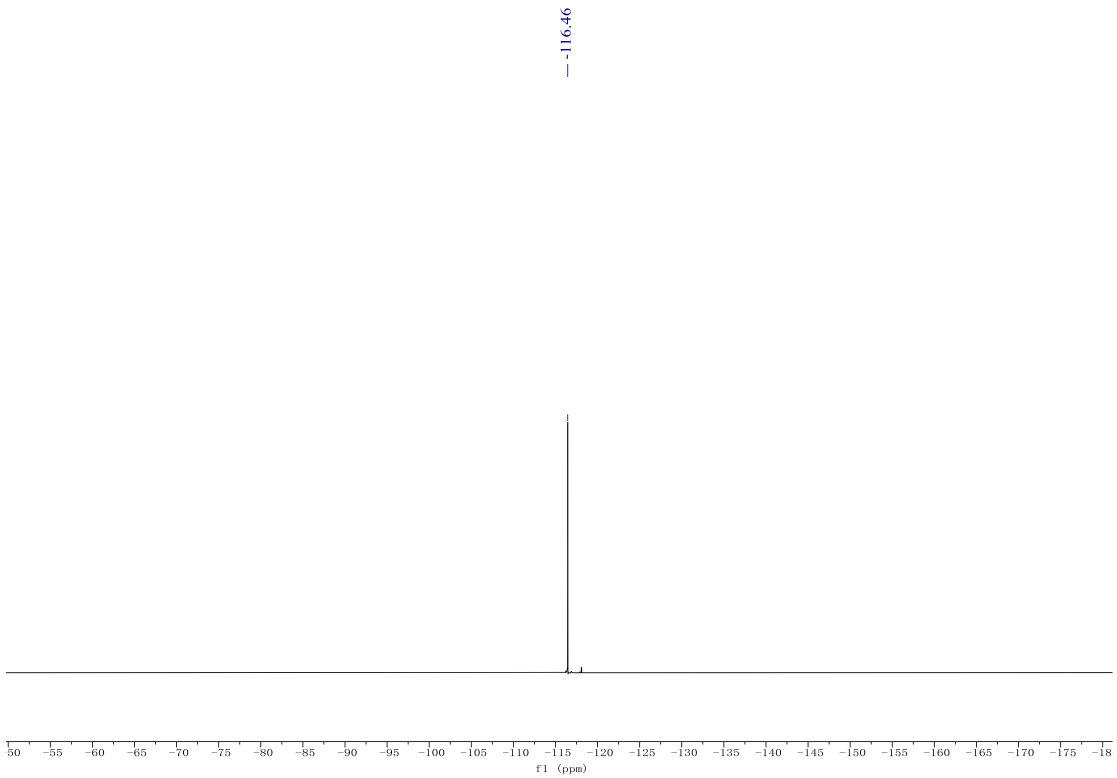
-116.46
f1 (ppm)

3'-chloro-2'-methyl-[1,1'-biphenyl]-2,6-dicarbaldehyde (1i)

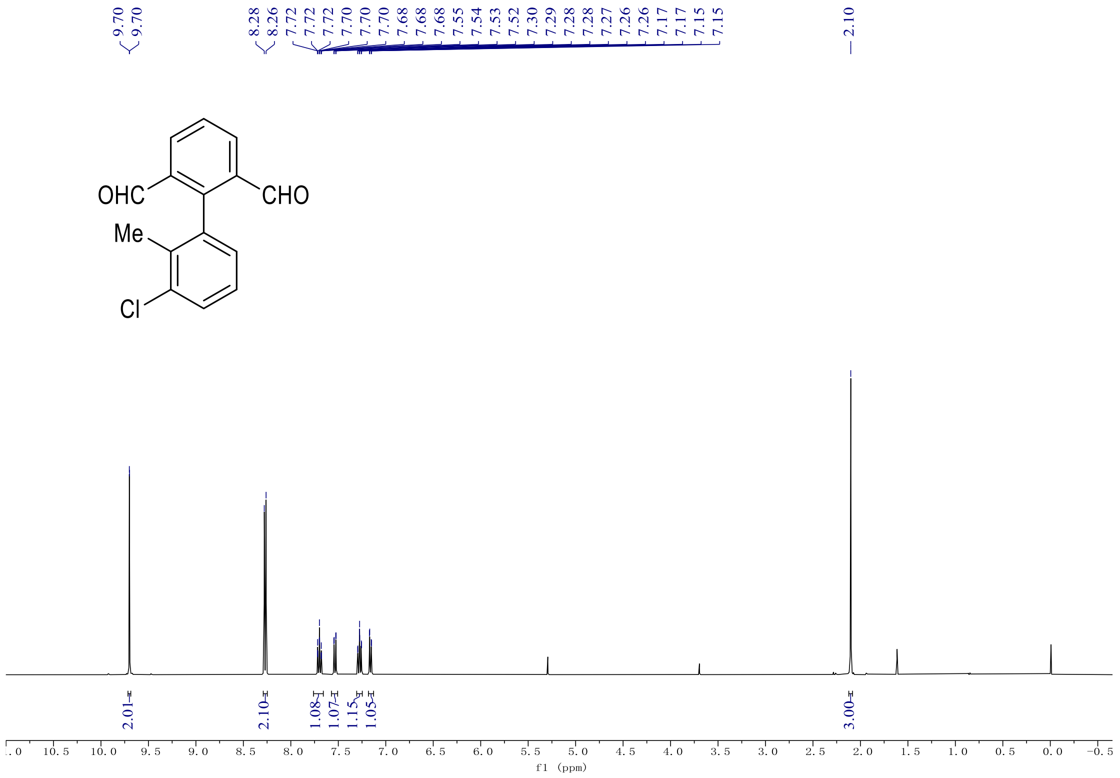

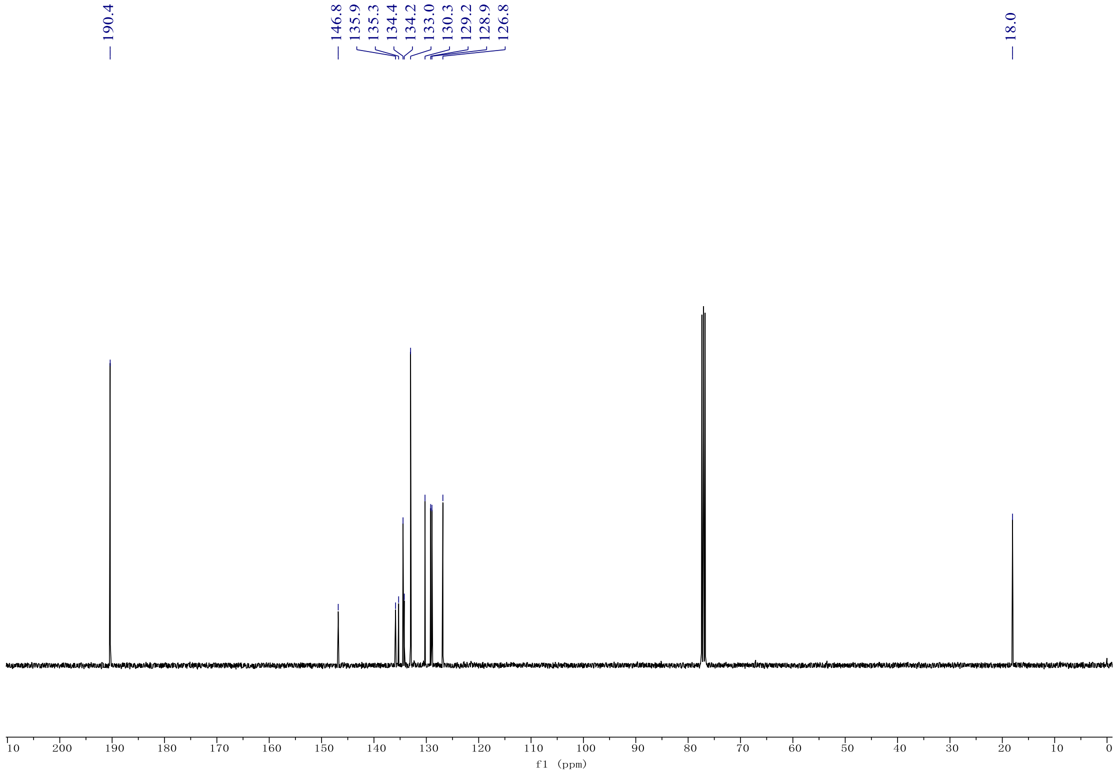

4'-chloro-2'-methyl-[1,1'-biphenyl]-2,6-dicarbaldehyde (1j)

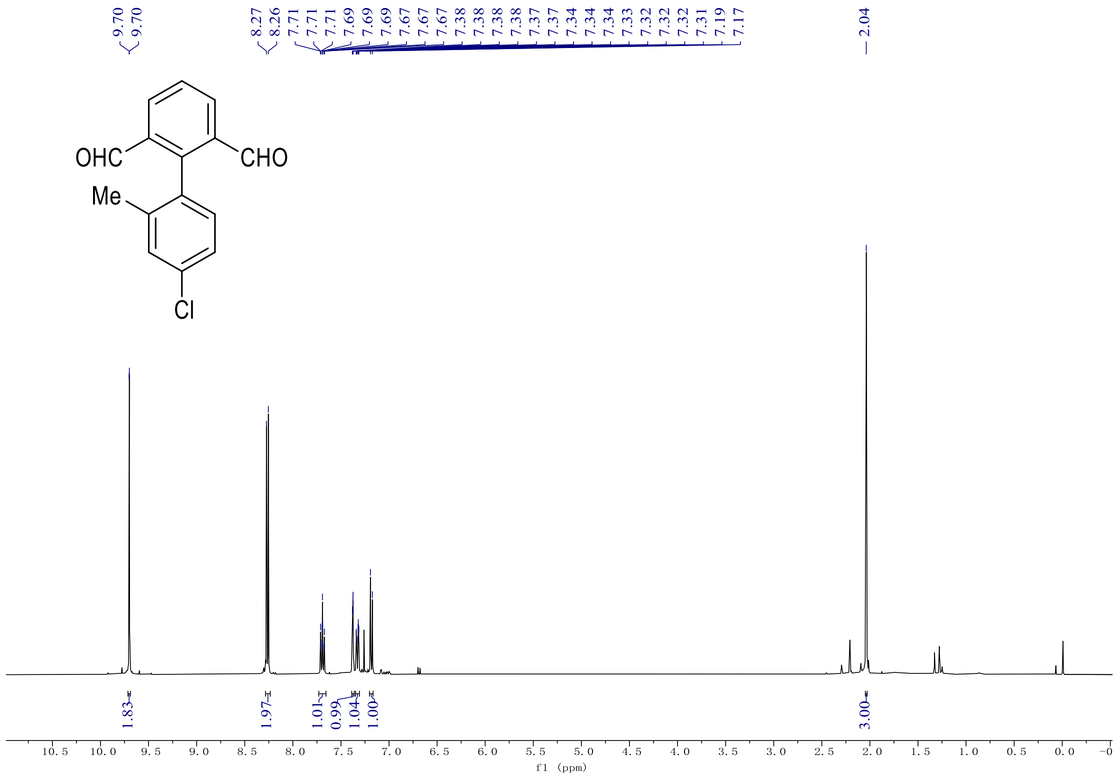

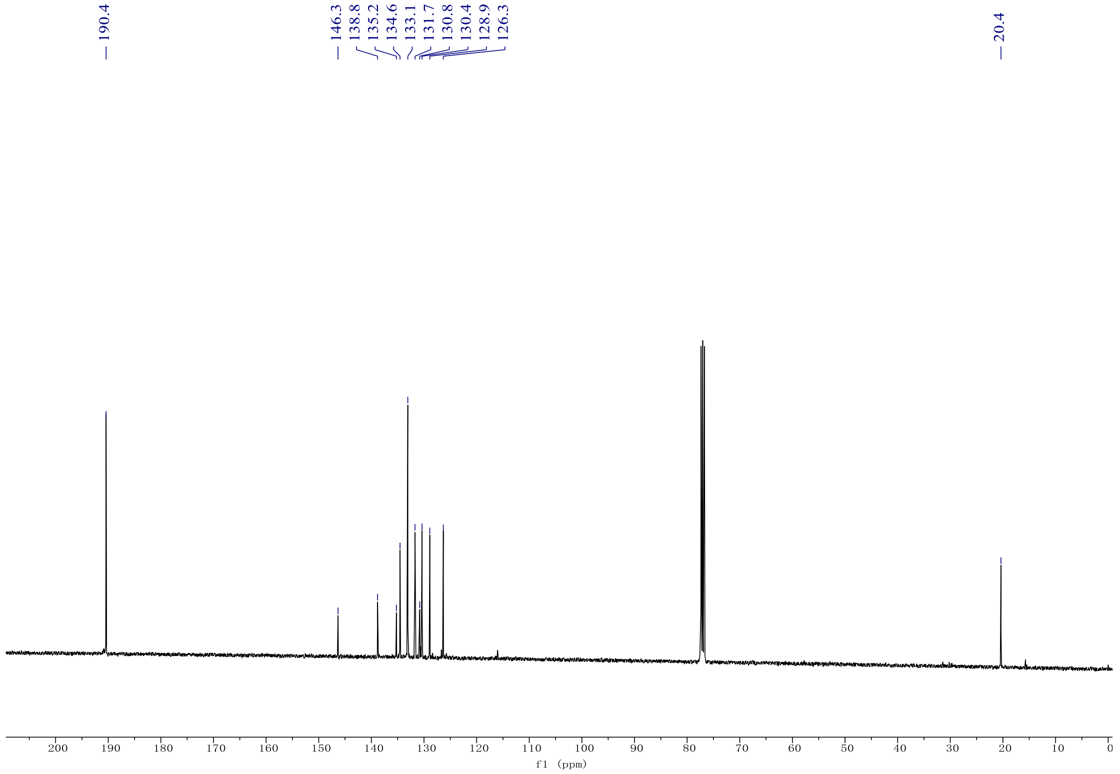

5'-chloro-2'-methyl-[1,1'-biphenyl]-2,6-dicarbaldehyde (1k)

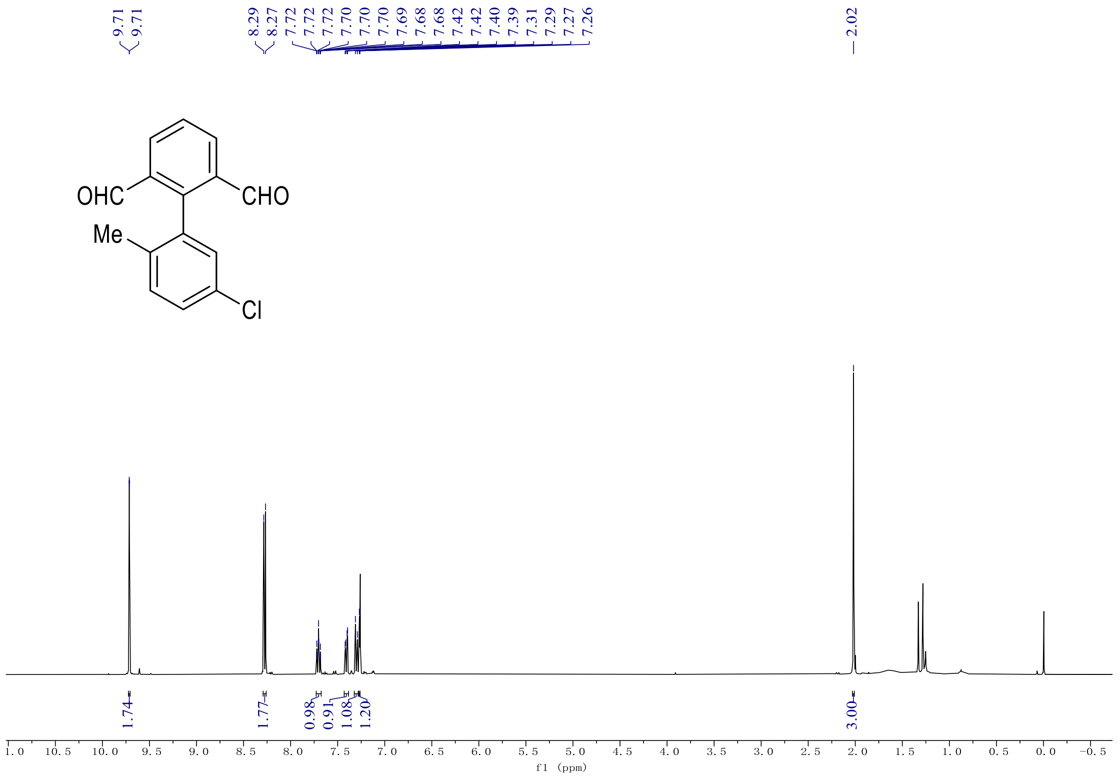

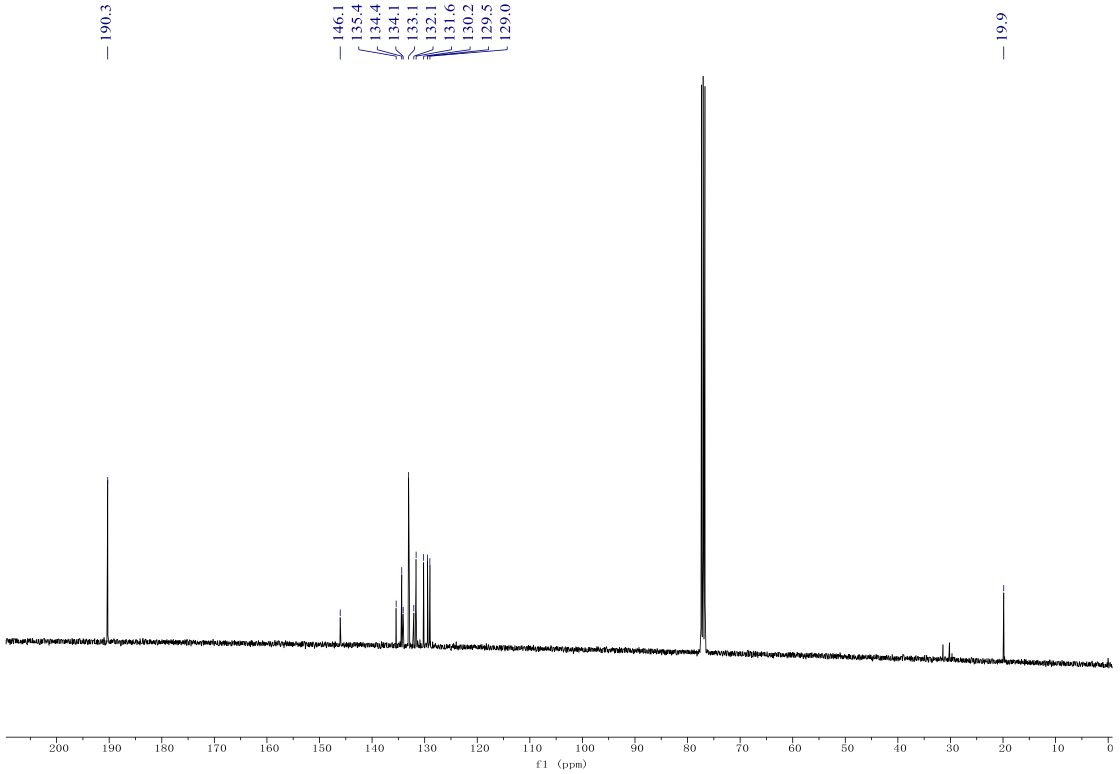

2'-ethyl-[1,1'-biphenyl]-2,6-dicarbaldehyde (1l)

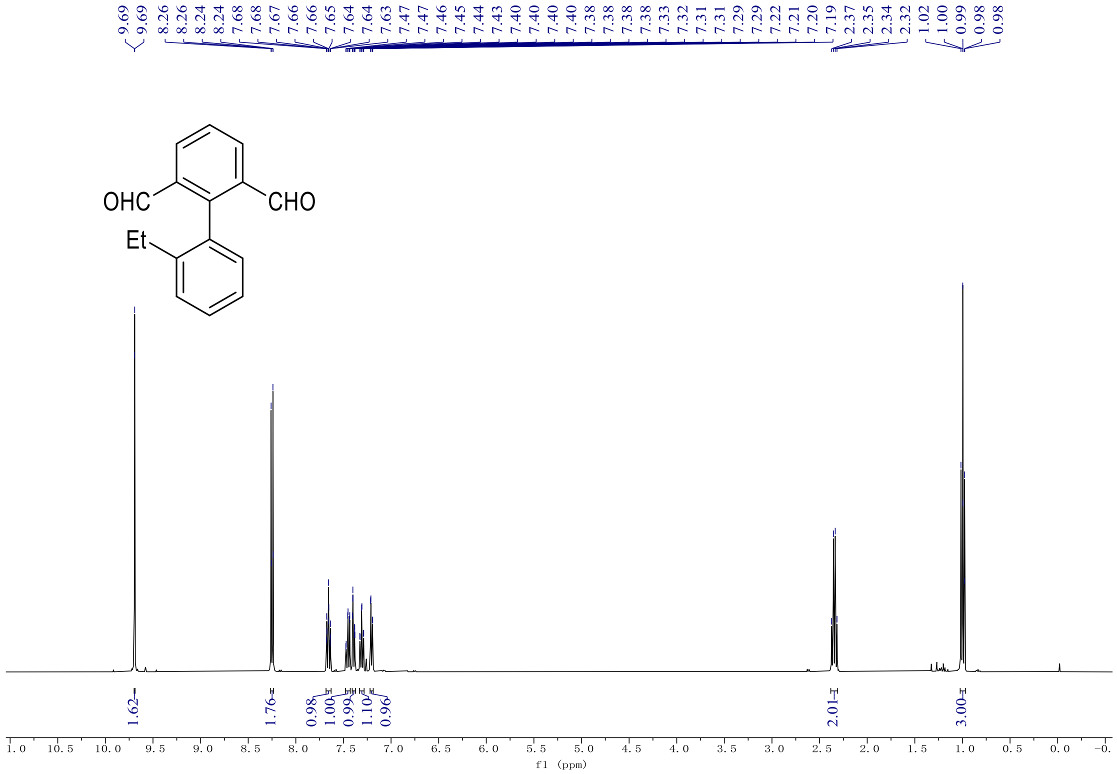

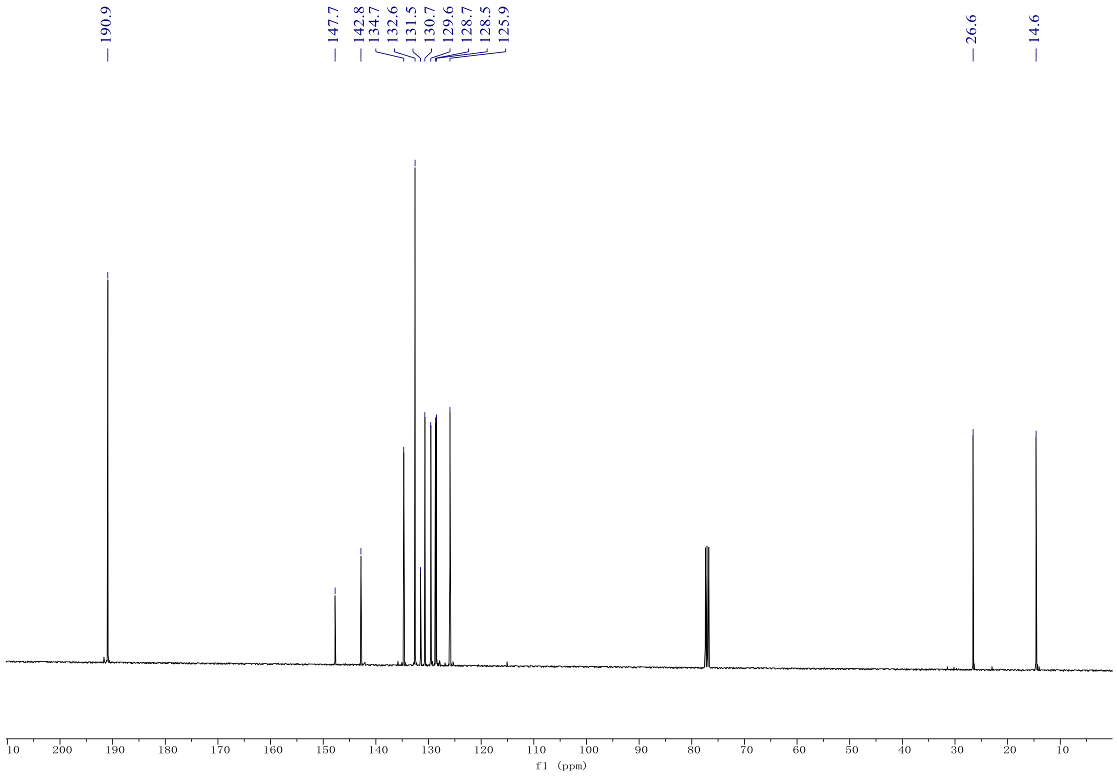

2'-(methylthio)-[1,1'-biphenyl]-2,6-dicarbaldehyde (1m)

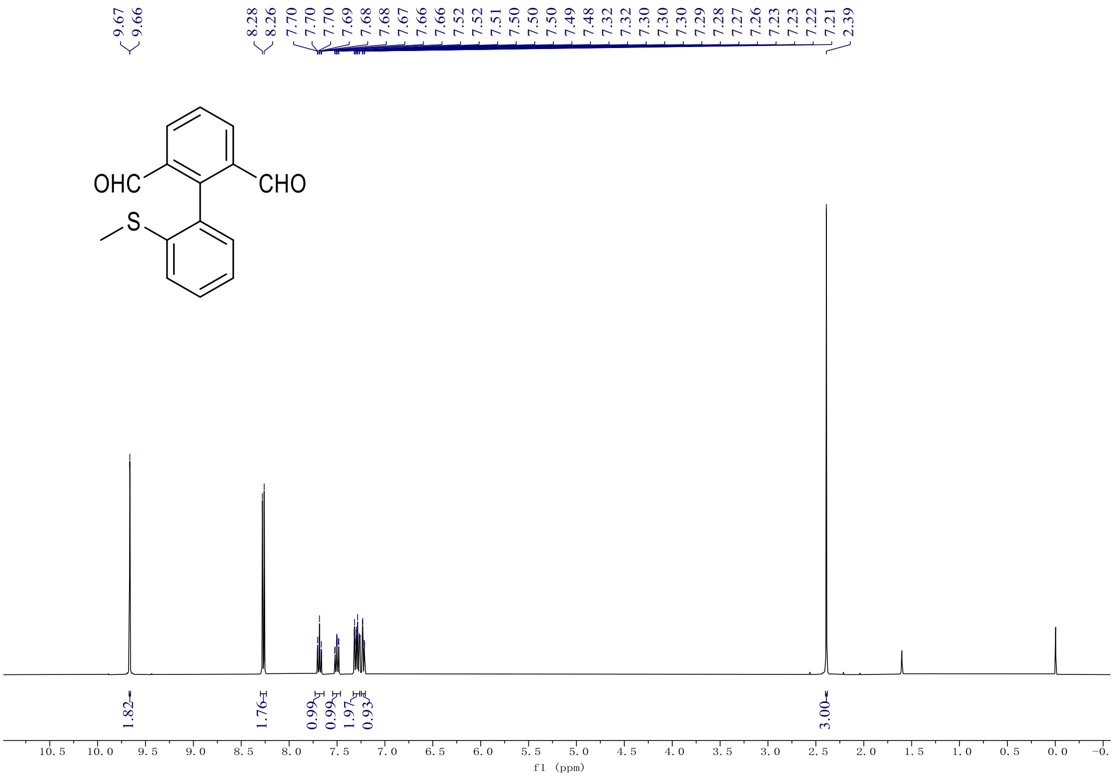

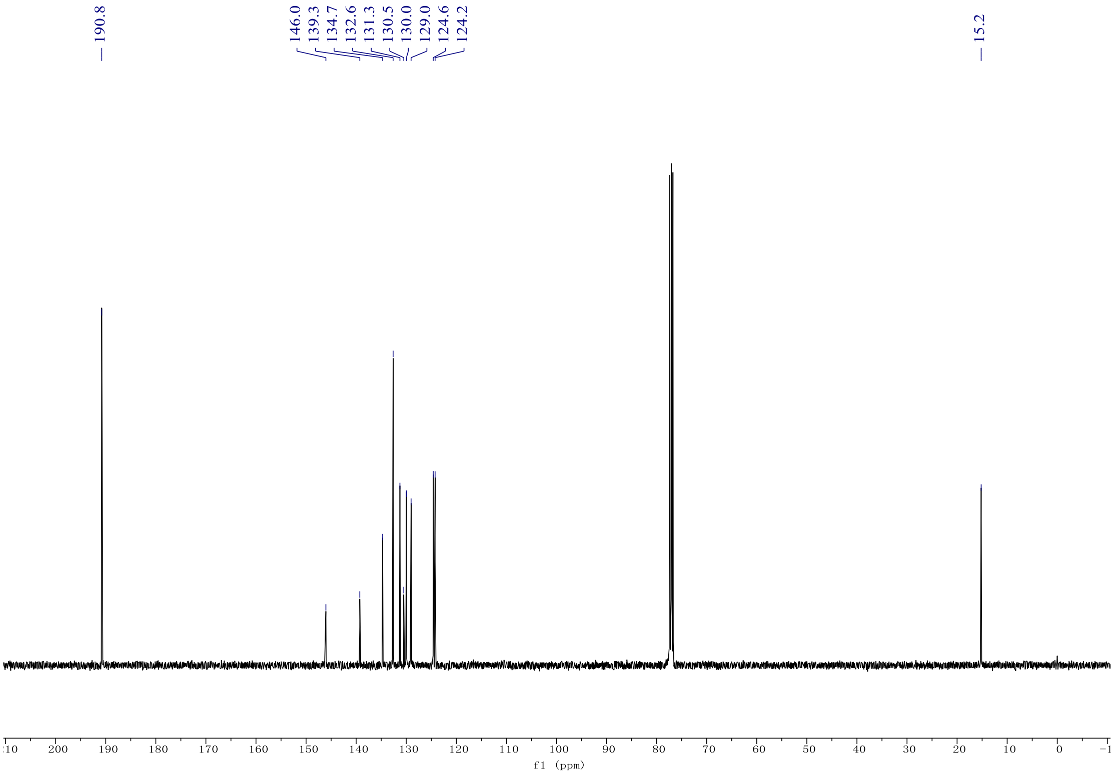

2'-vinyl-[1,1'-biphenyl]-2,6-dicarbaldehyde (1n)

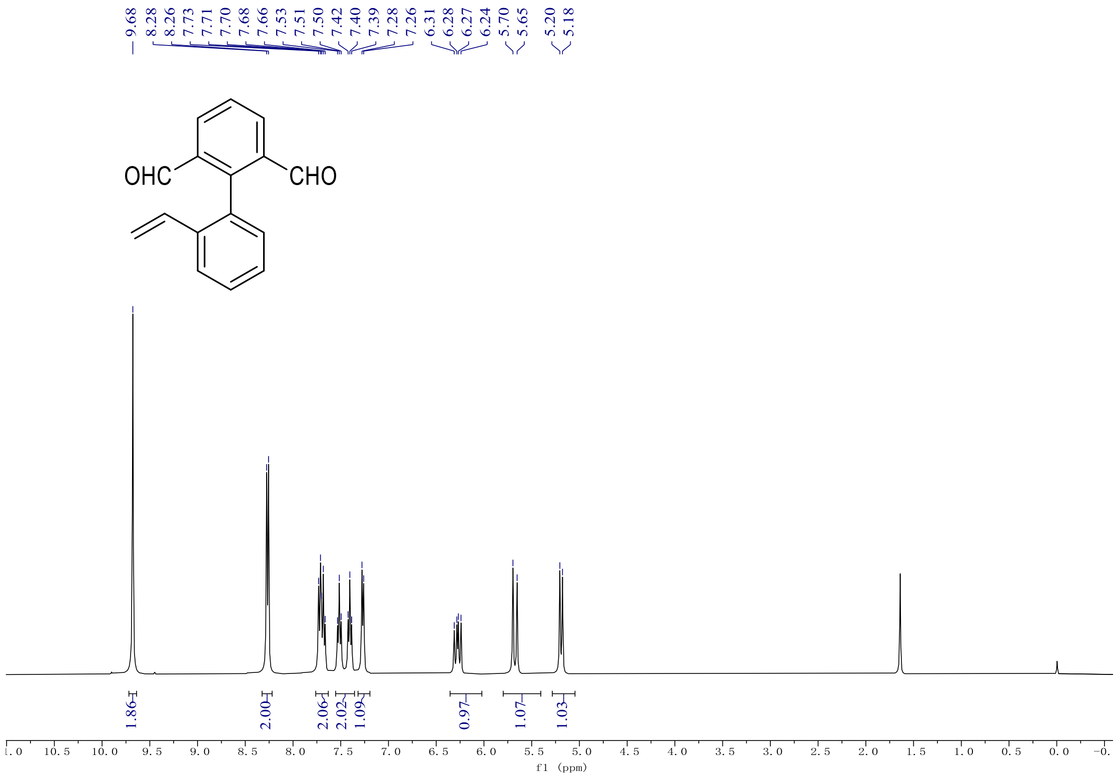

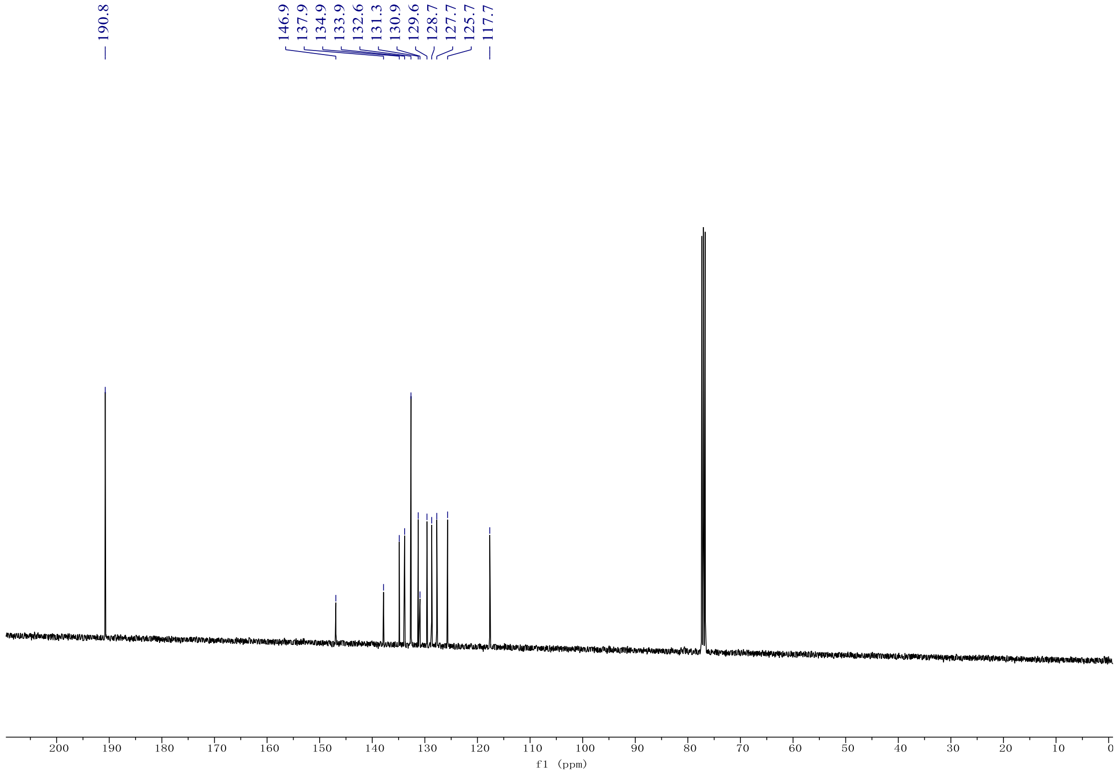

2'-chloro-[1,1'-biphenyl]-2,6-dicarbaldehyde (1o)

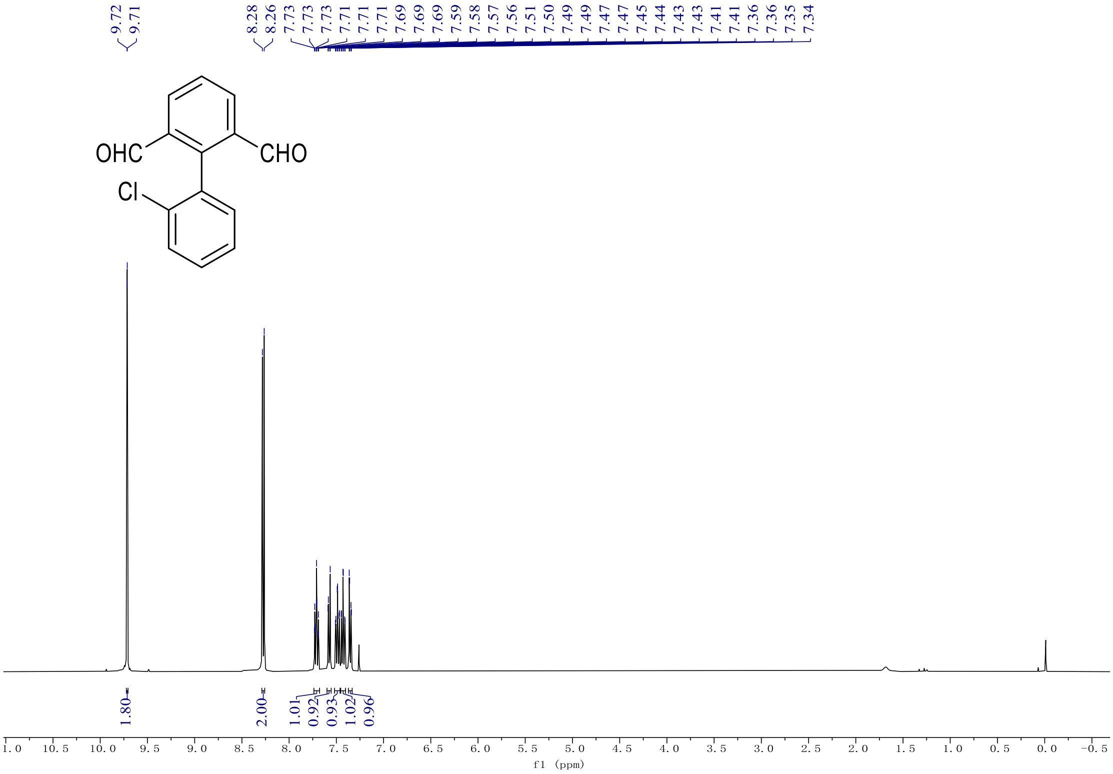

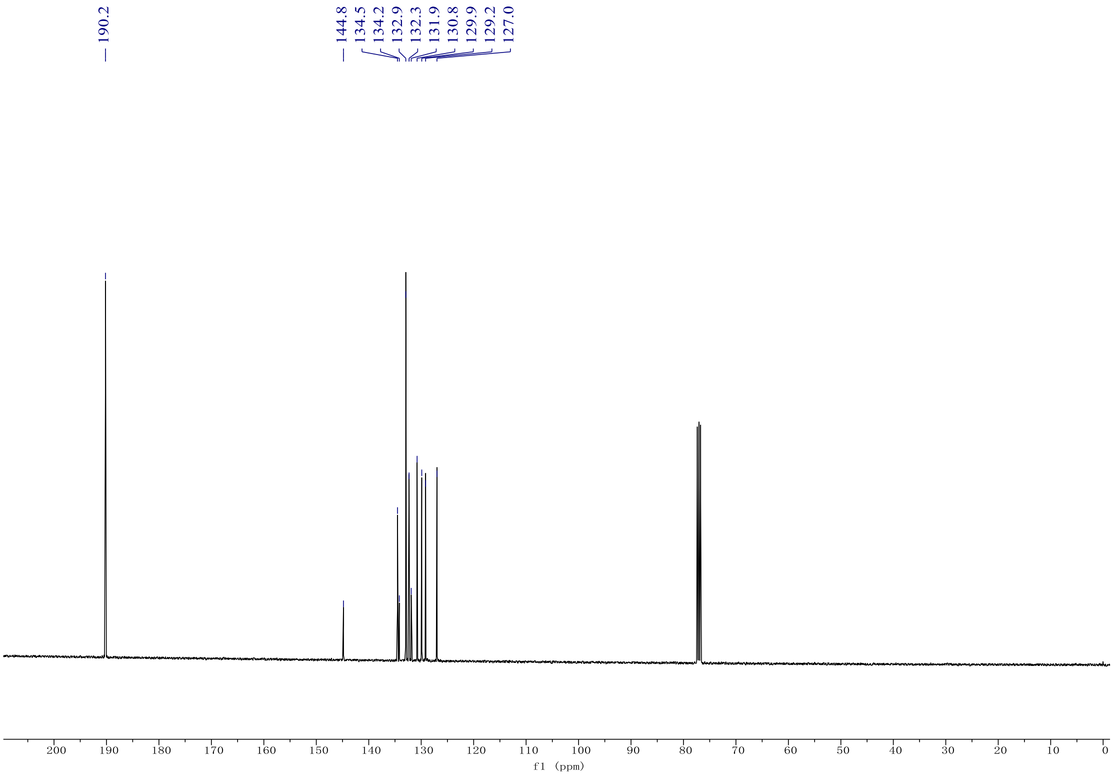

2'-bromo-[1,1'-biphenyl]-2,6-dicarbaldehyde (1p)

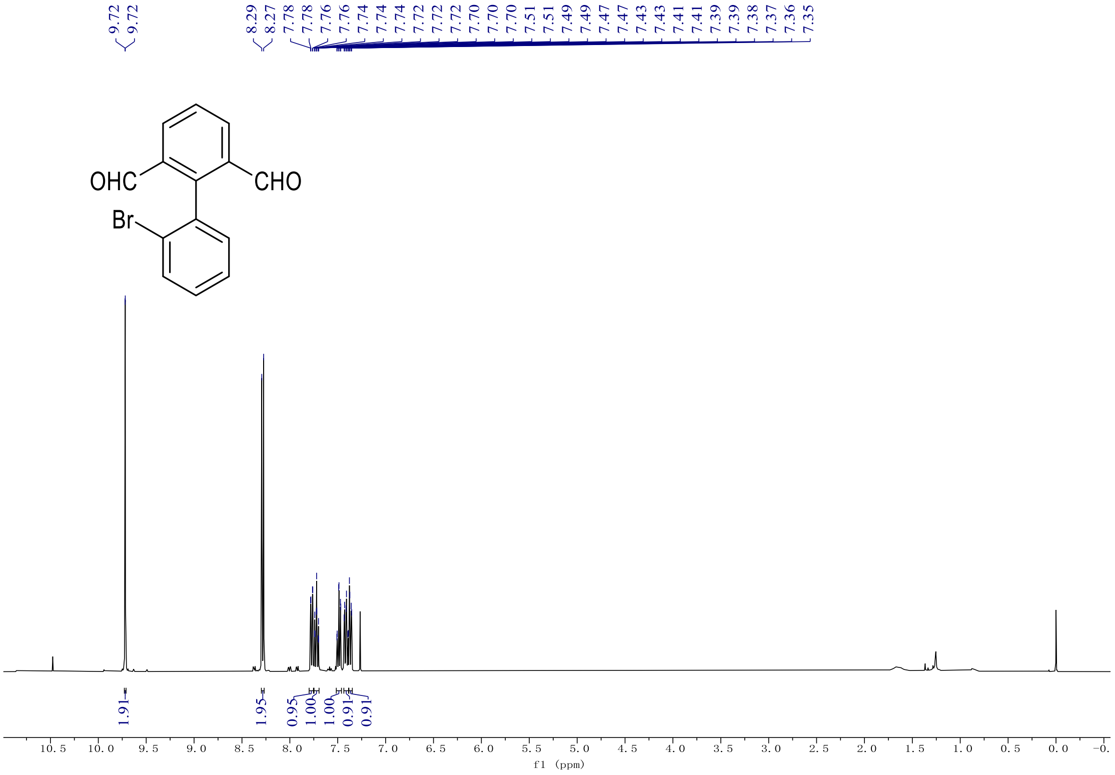

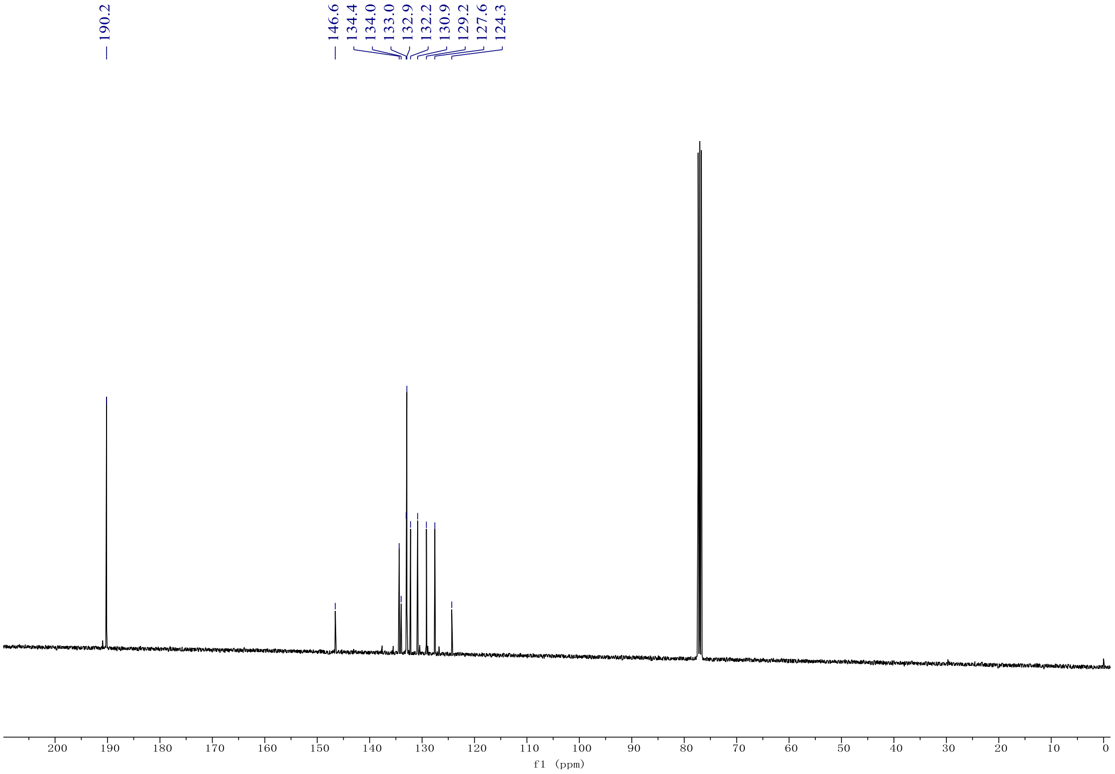

[1,1':2',1''-terphenyl]-2,6-dicarbaldehyde (1q)

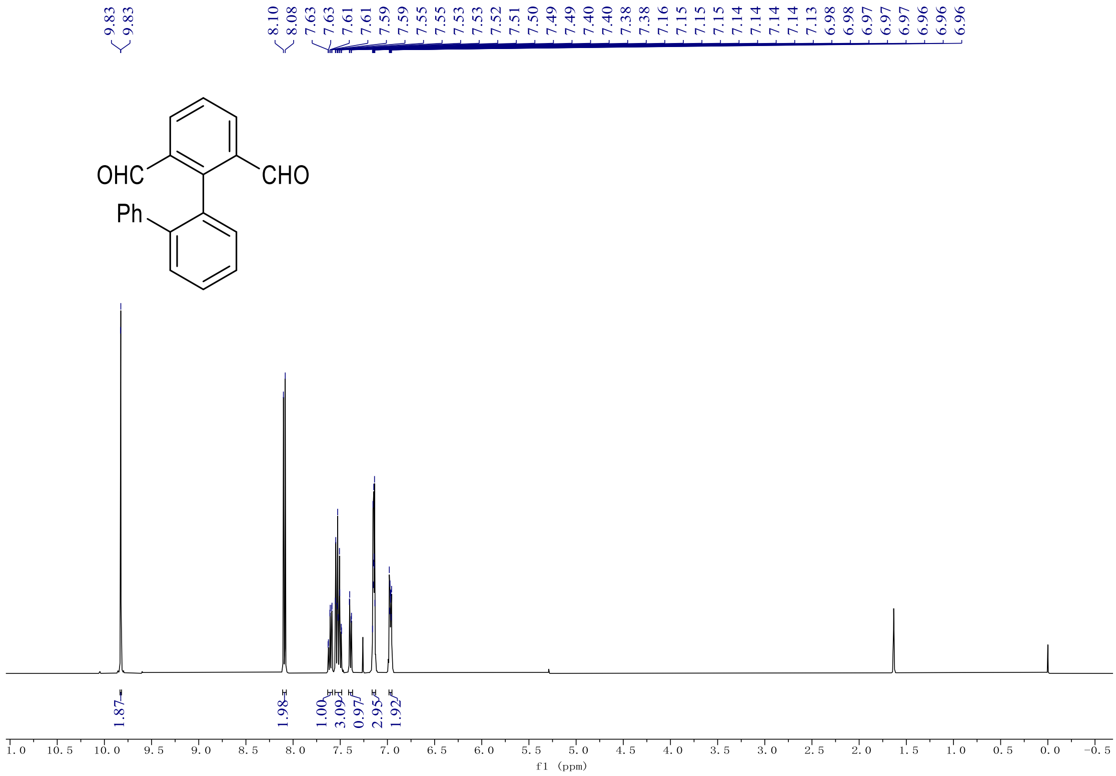

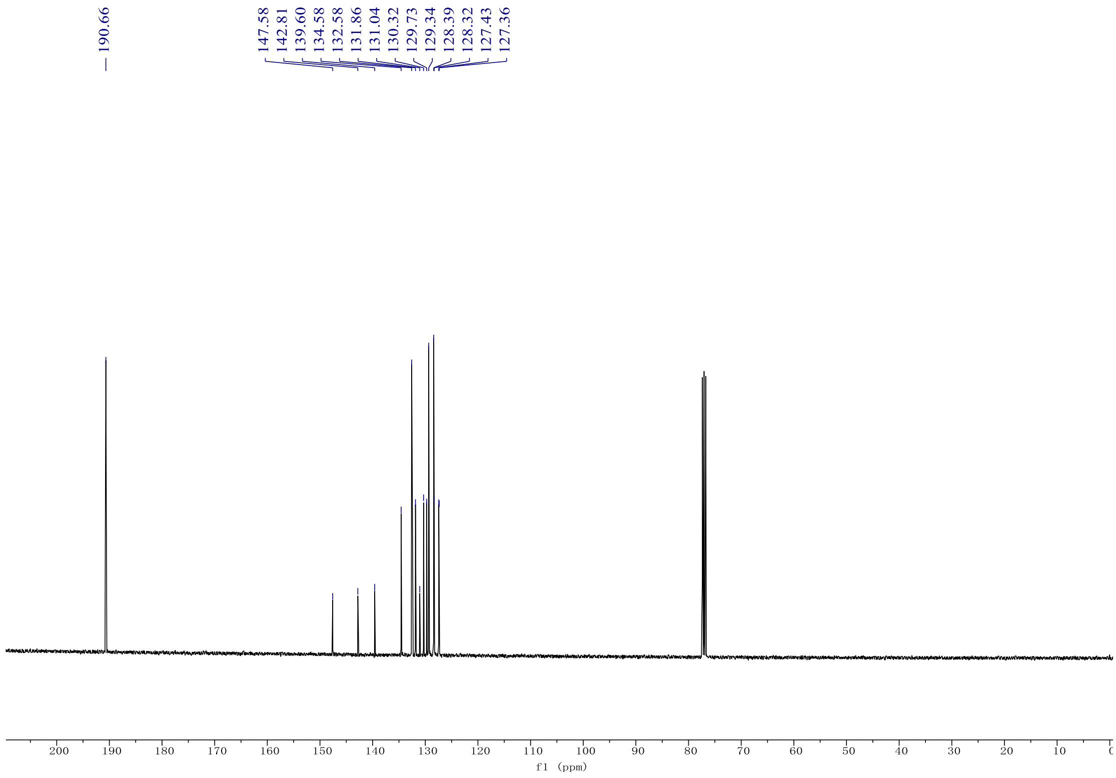

2-(naphthalen-1-yl)isophthalaldehyde (1r)

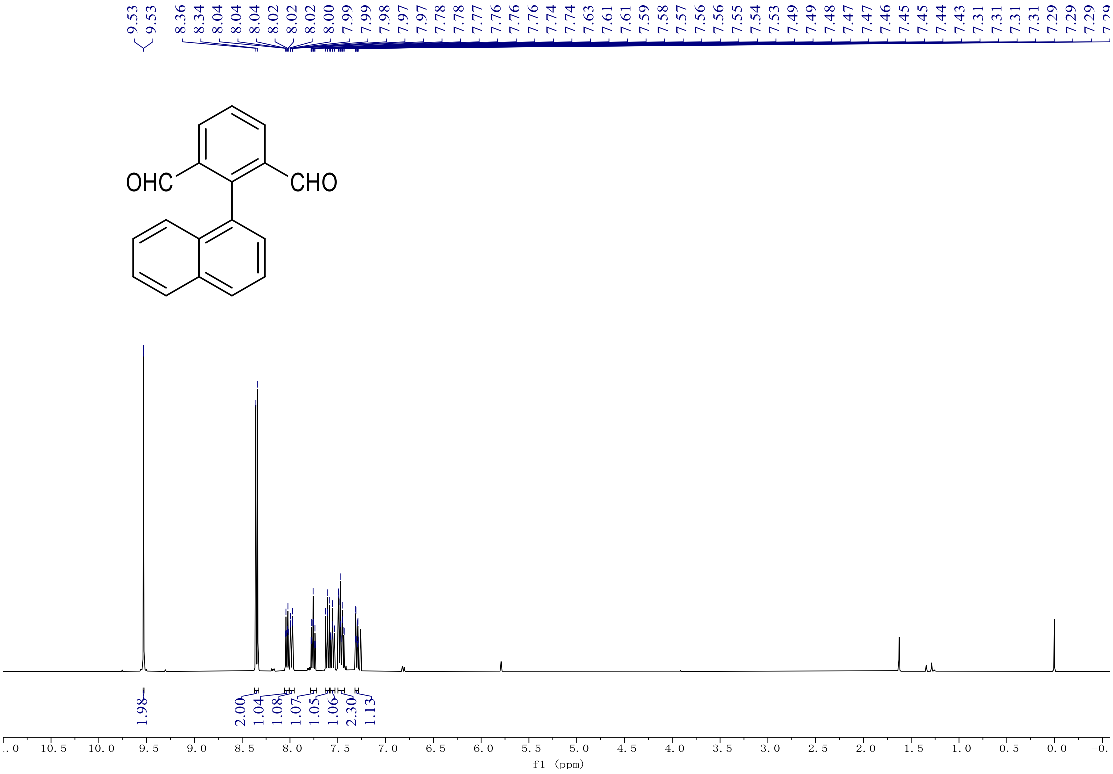

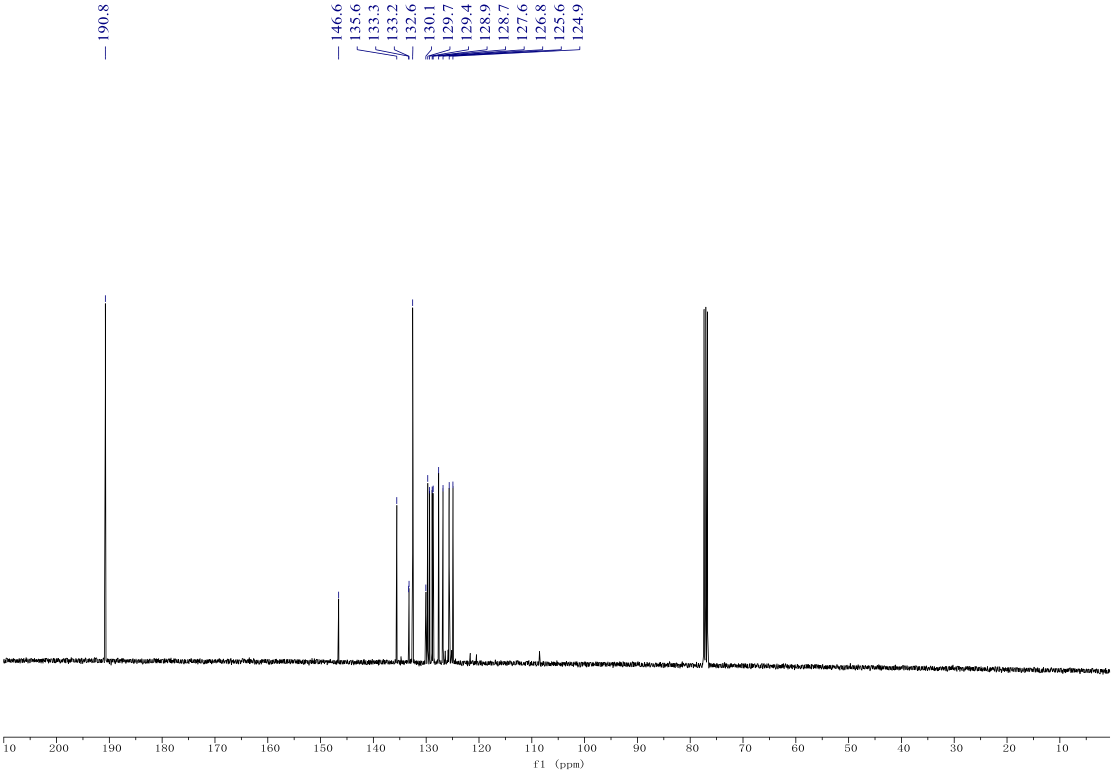

2-(phenanthren-9-yl)isophthalaldehyde (1s)

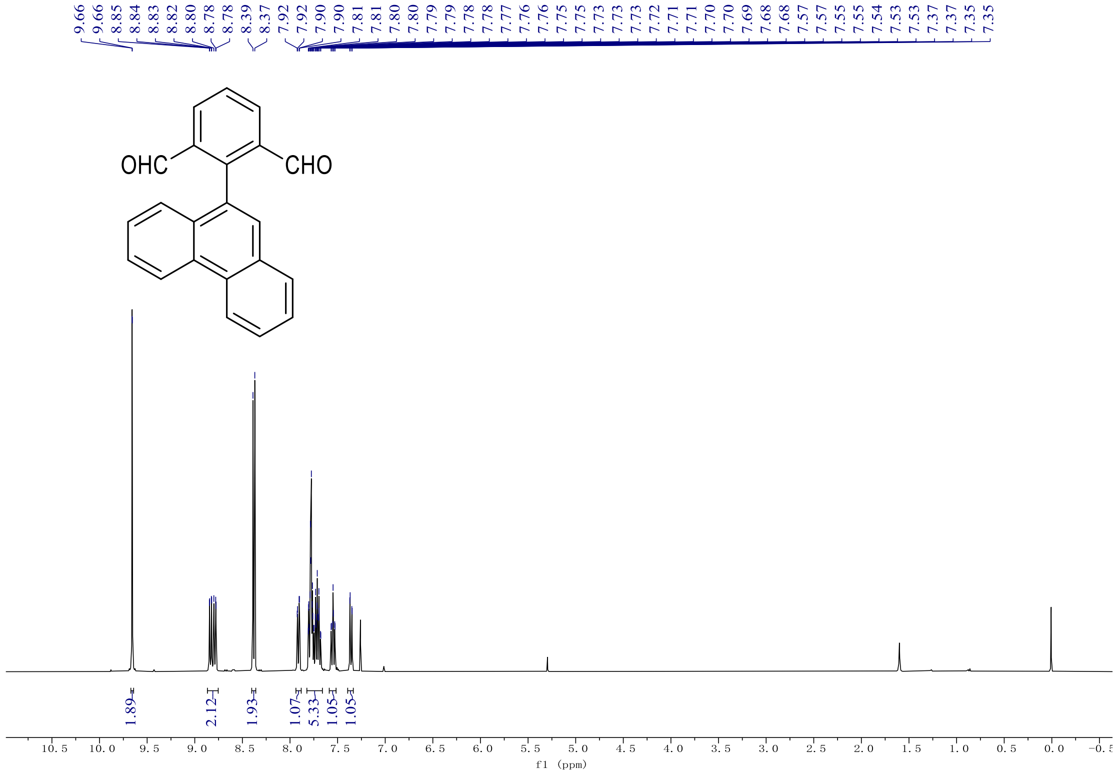

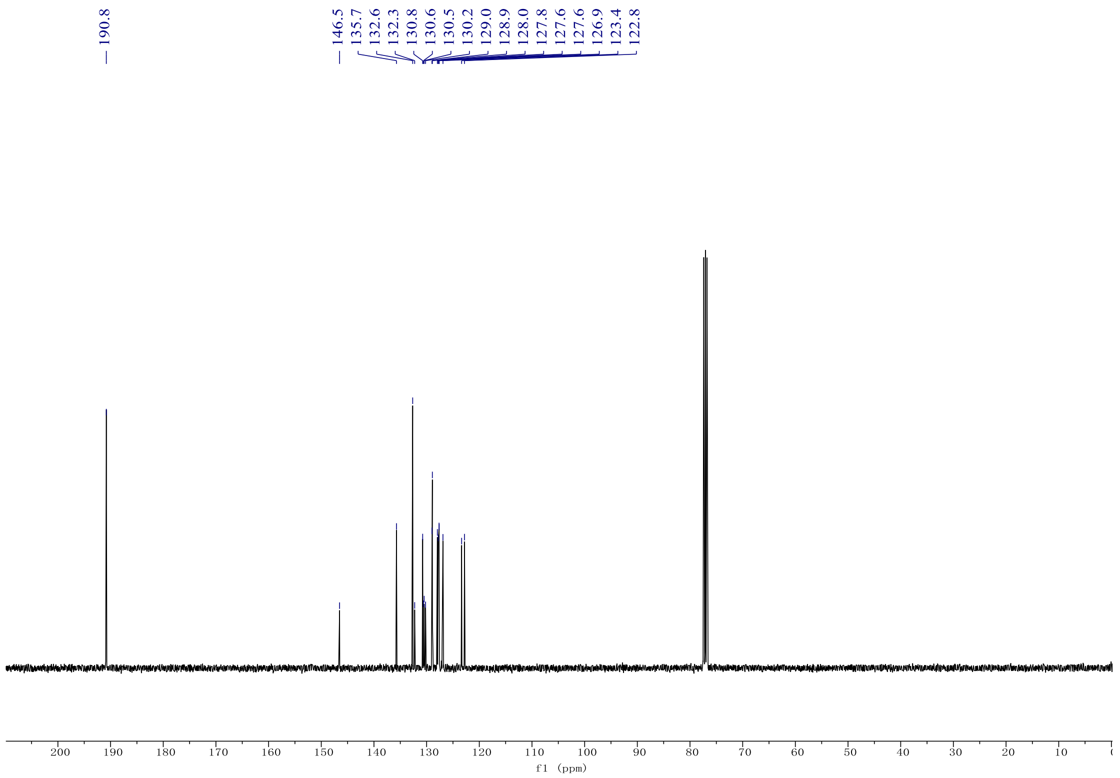

2-(pyren-1-yl)isophthalaldehyde (1t)

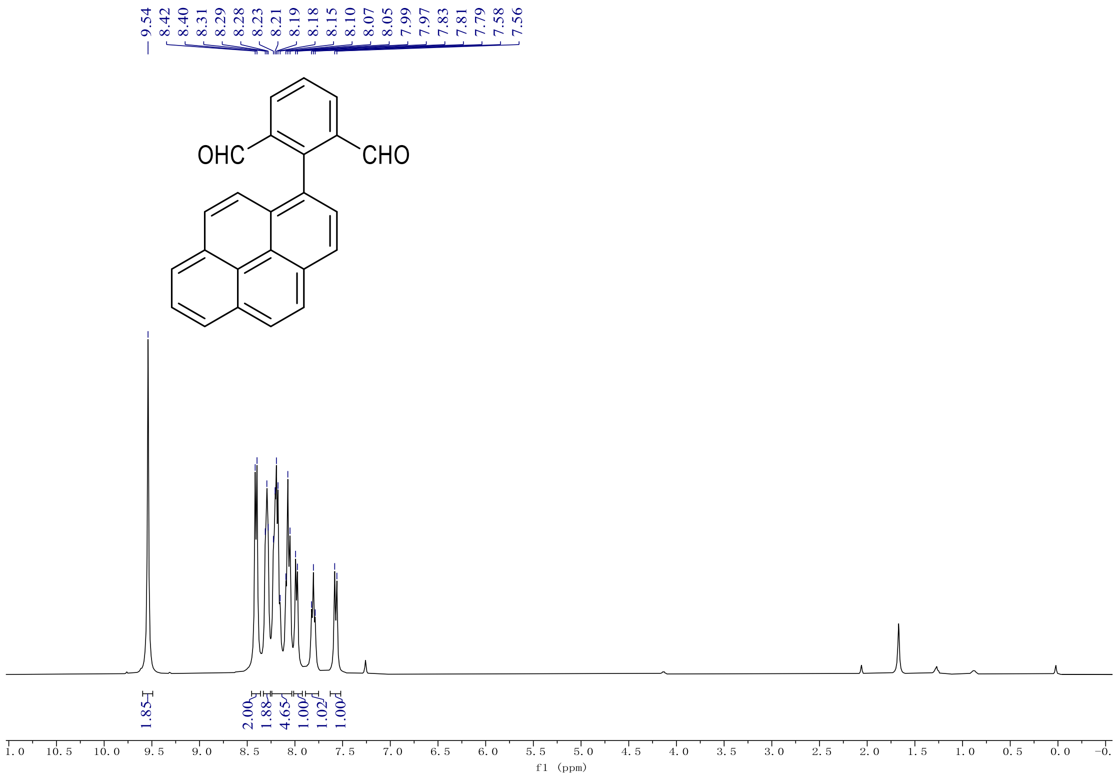

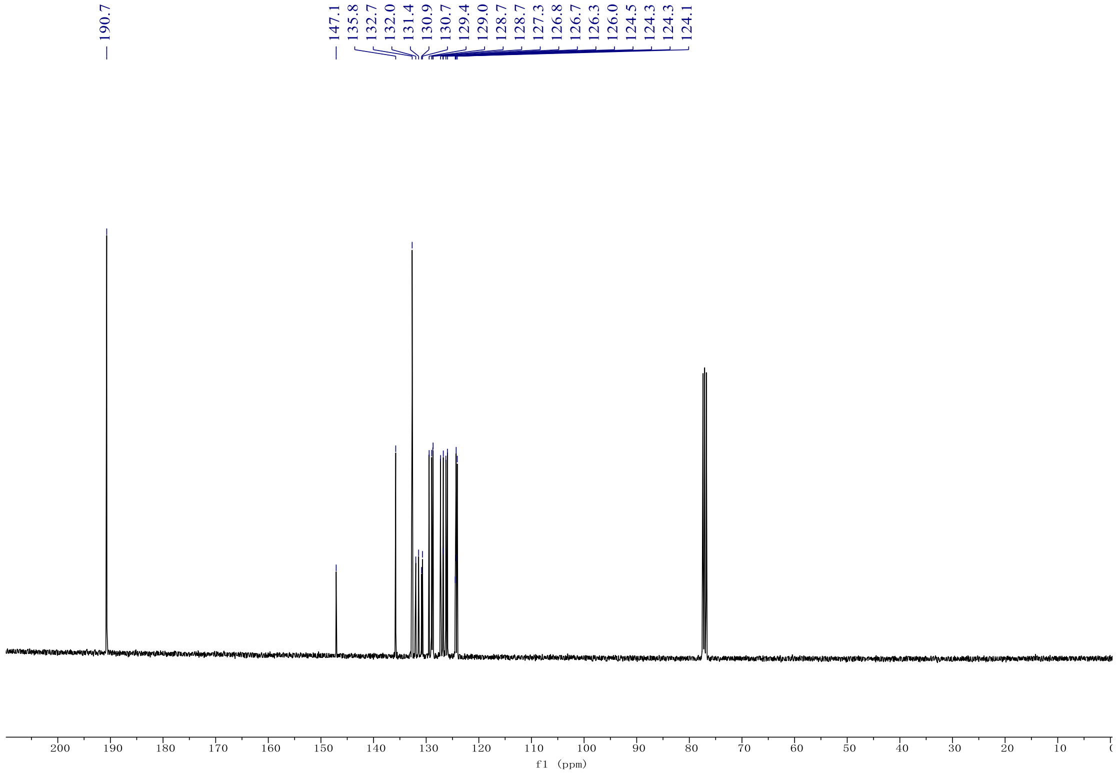

2-(2-methoxynaphthalen-1-yl)isophthalaldehyde (1u)

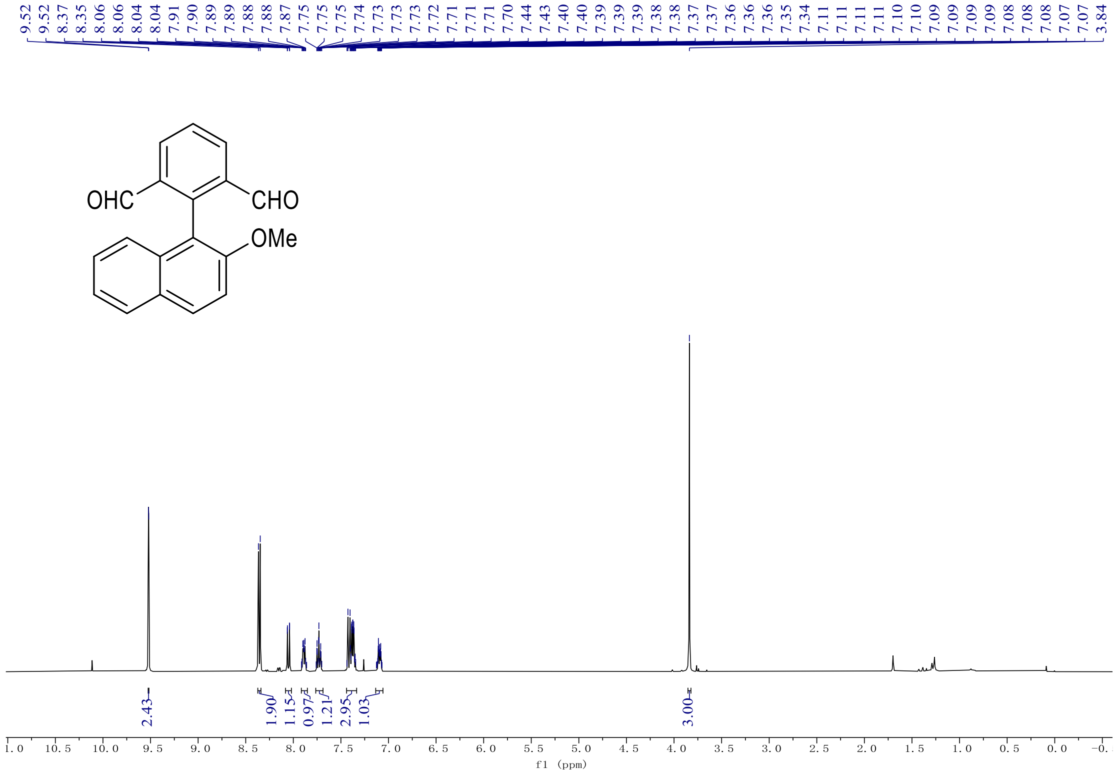

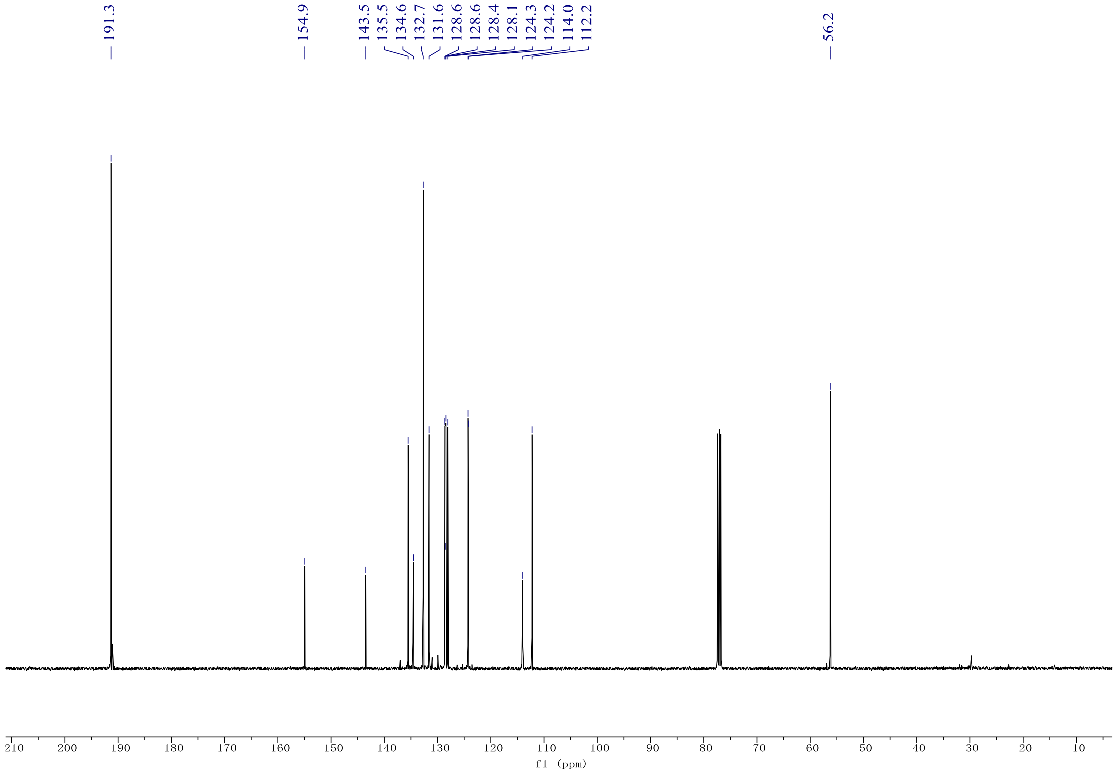

4-methoxy-2'-methyl-[1,1'-biphenyl]-2,6-dicarbaldehyde (1v)

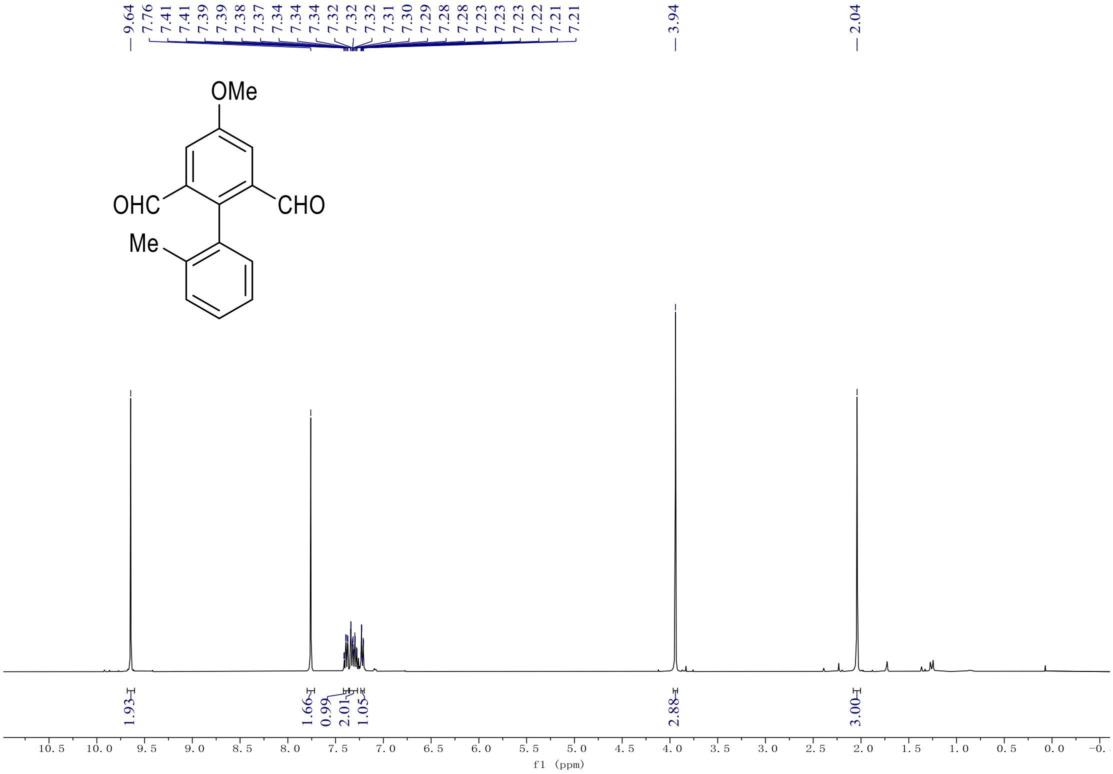

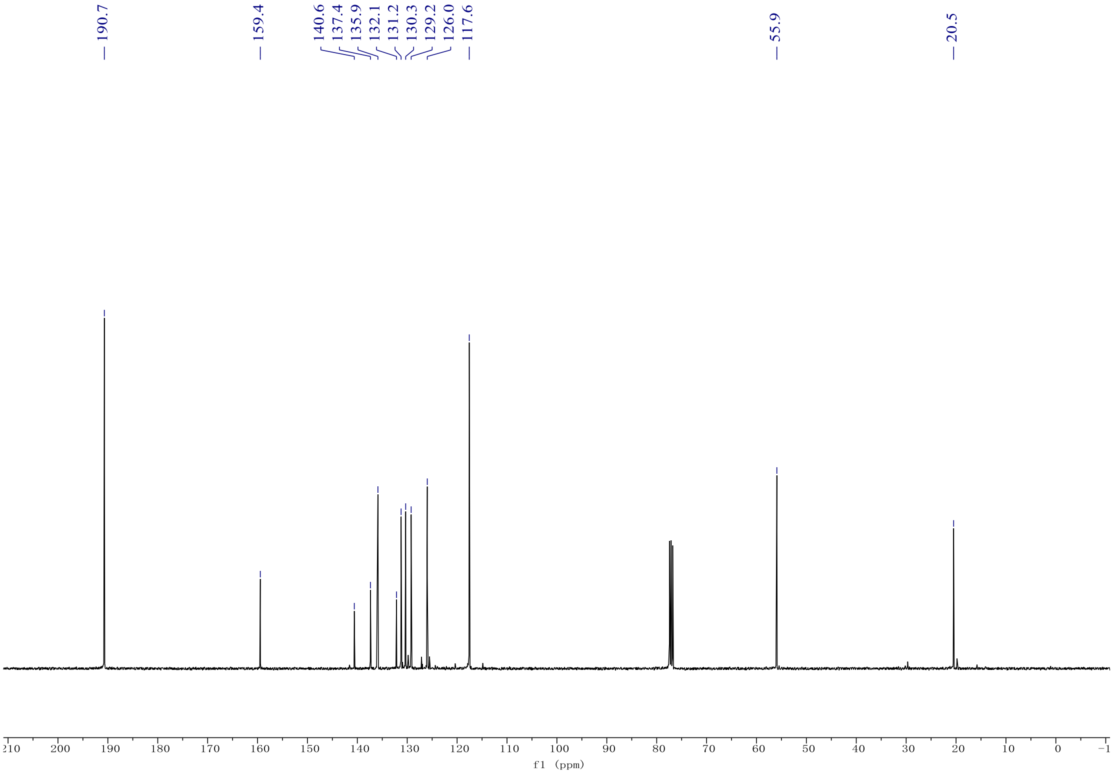

4-fluoro-2'-methyl-[1,1'-biphenyl]-2,6-dicarbaldehyde (1w)

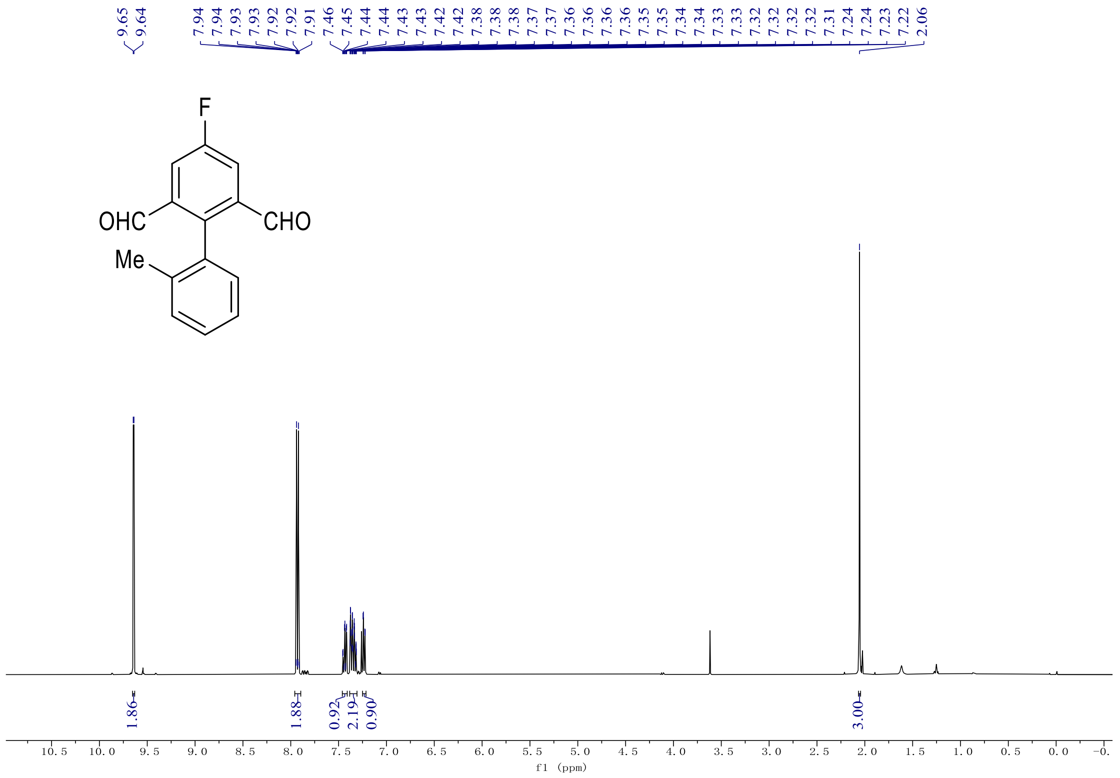

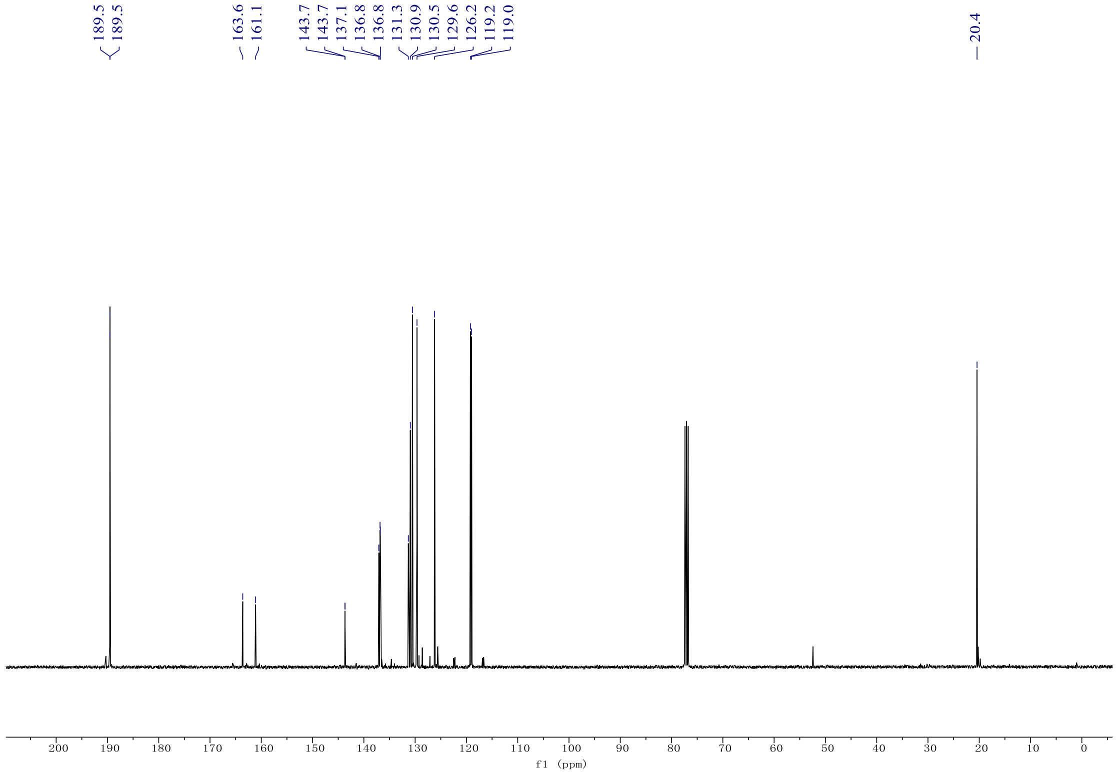

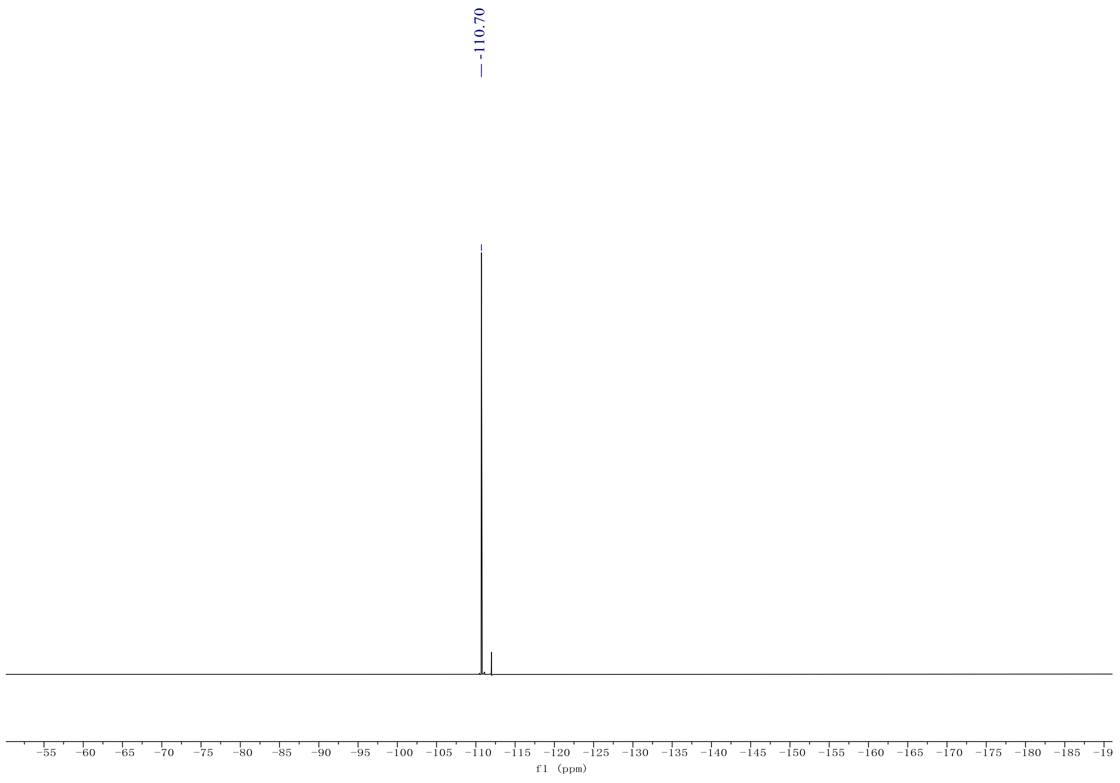
-110.70
-55 -60 -65 -70 -75 -80 -85 -90 -95 -100 -105 -110 -115 -120 -125 -130 -135 -140 -145 -150 -155 -160 -165 -170 -175 -180 -185 -19
f1 (ppm)

2'-methyl-[1,1'-biphenyl]-2,6-dicarbonitrile (4a)

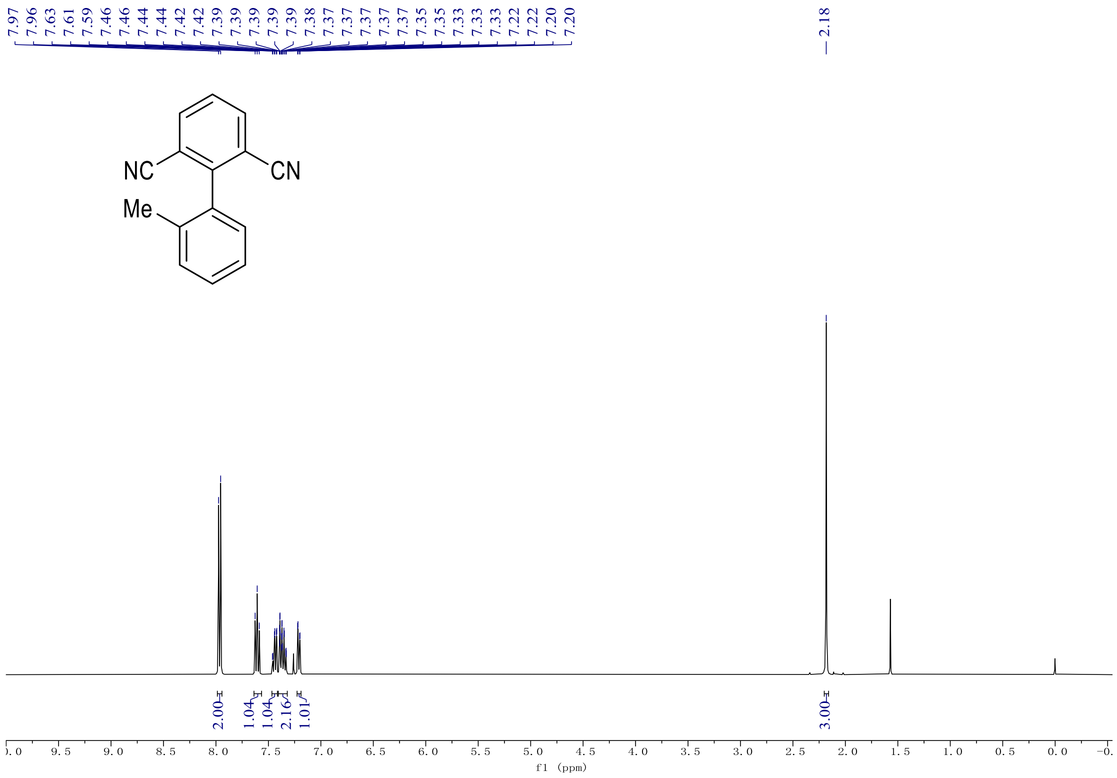

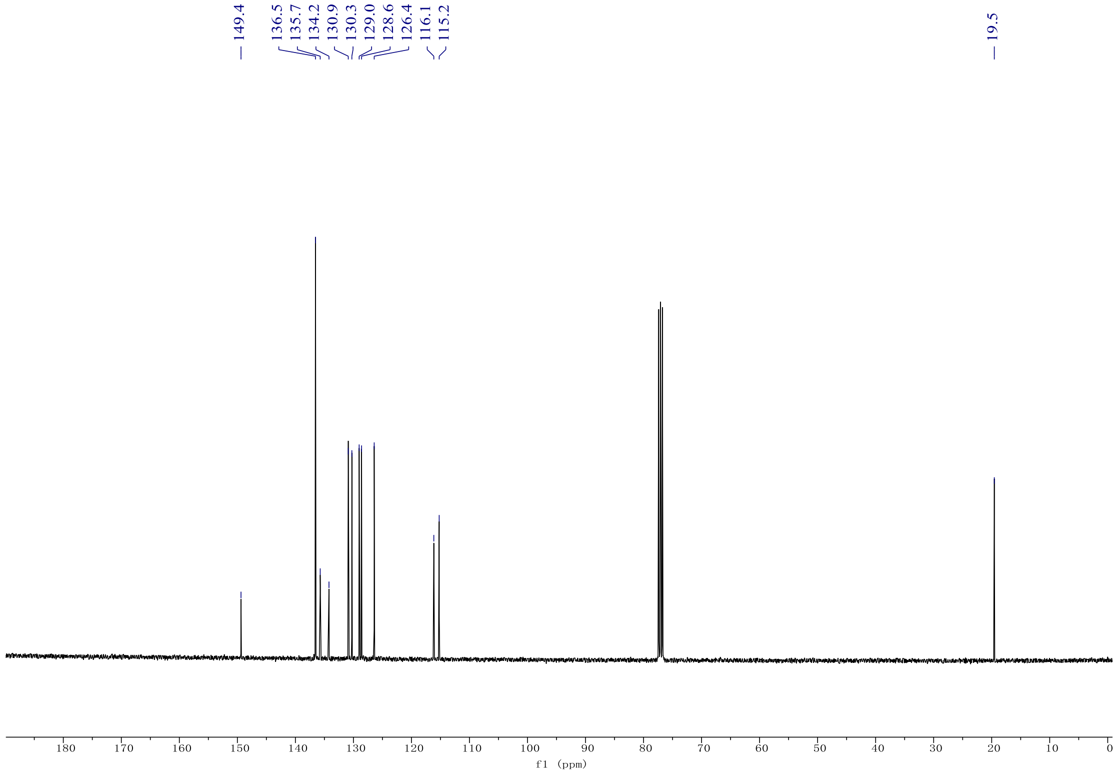

(*S*)-6-formyl-2'-methyl-[1,1'-biphenyl]-2-carbonitrile (3a)

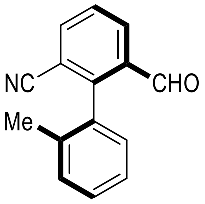

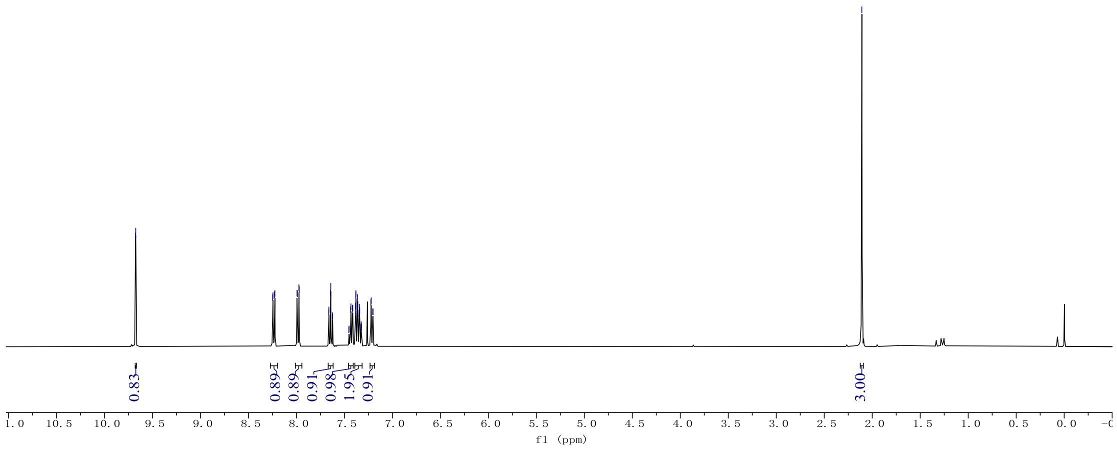

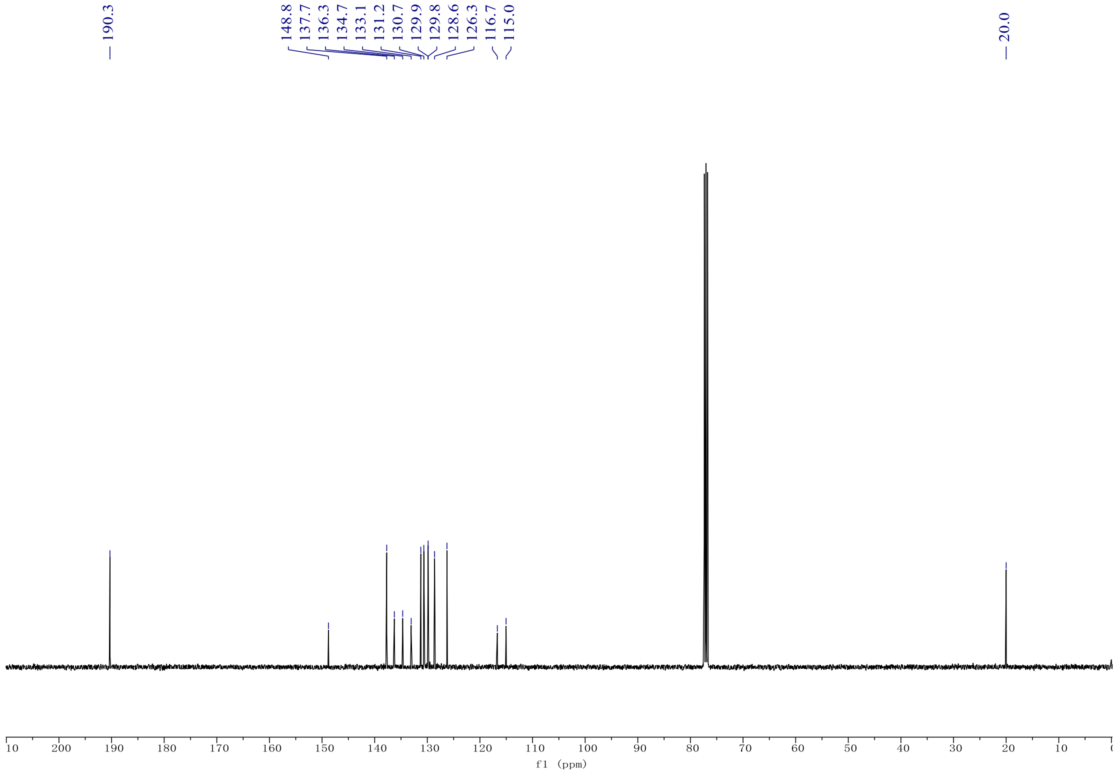

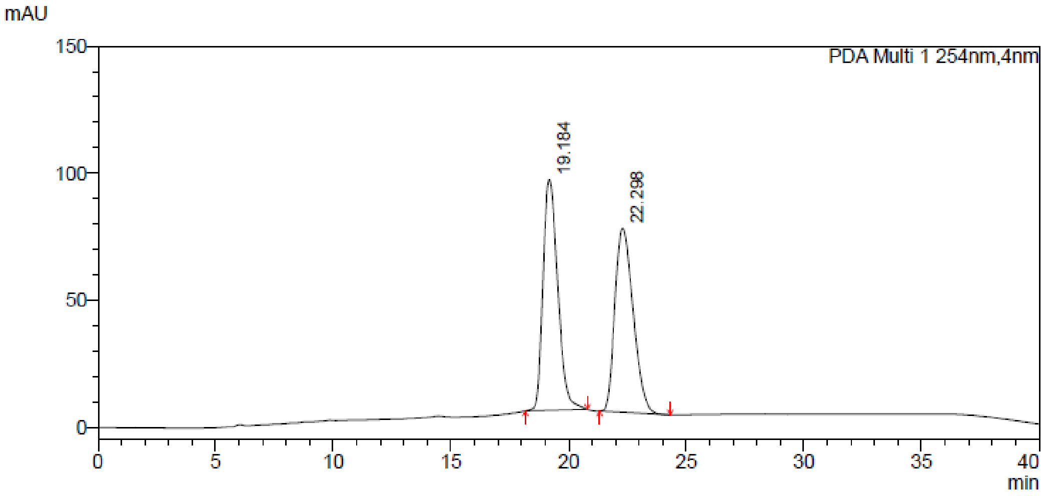

PDA Ch1 254nm

Ret. Time	Height	Area	Area%
19.184	90638	3957413	50.304
22.298	72339	3909631	49.696
	162977	7867044	100.000

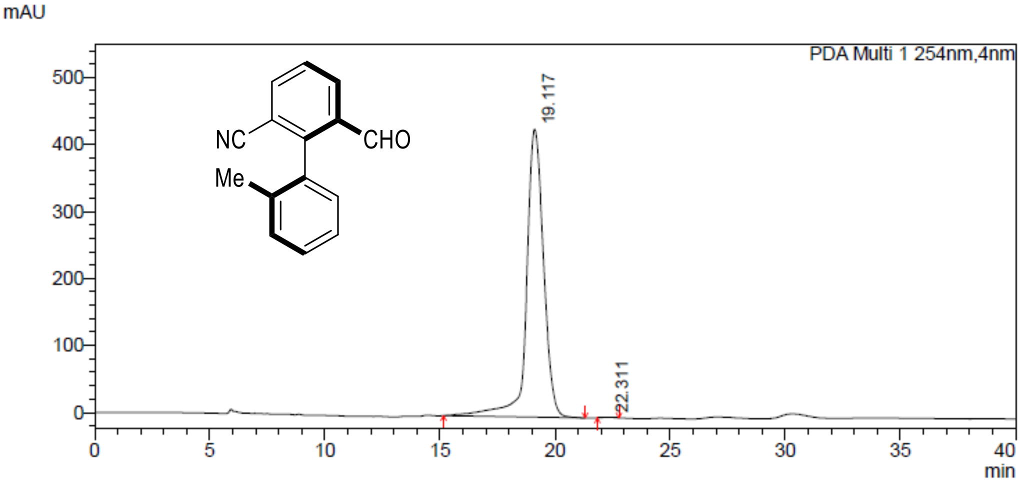

PDA Ch1 254nm

Ret. Time	Height	Area	Area%
19.117	429463	22275653	99.880
22.311	802	26817	0.120
	430265	22302470	100.000

(*S*)-6-formyl-2',3'-dimethyl-[1,1'-biphenyl]-2-carbonitrile (3b)

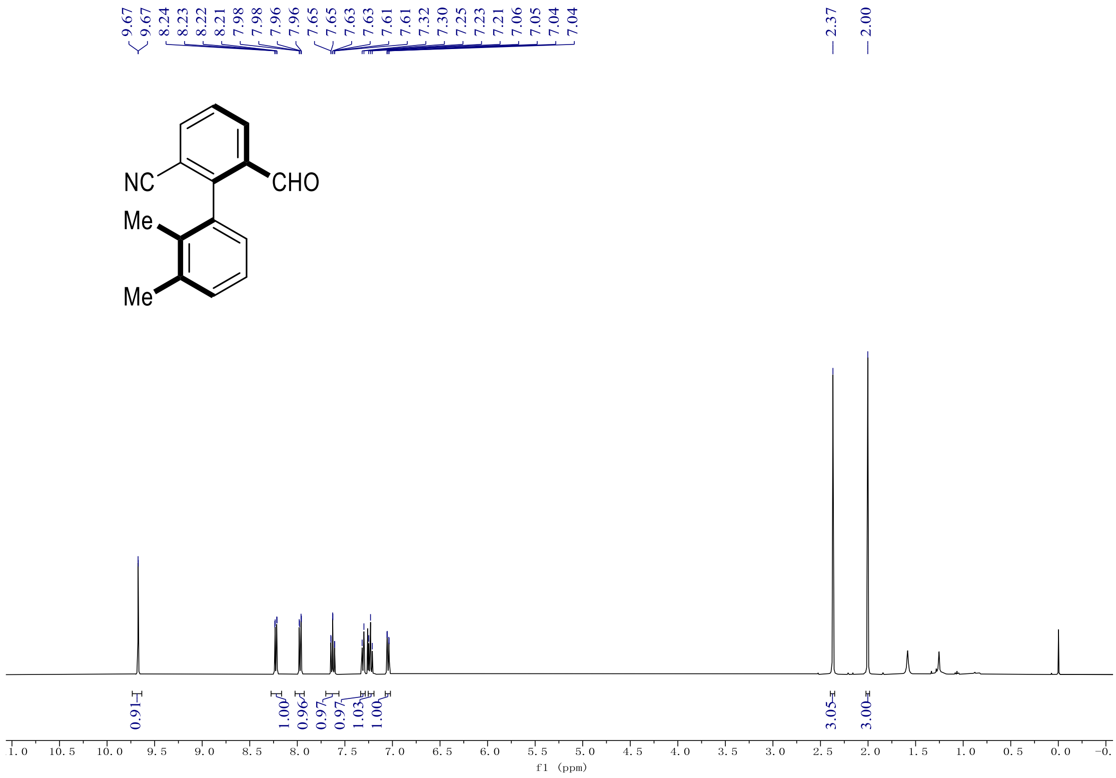

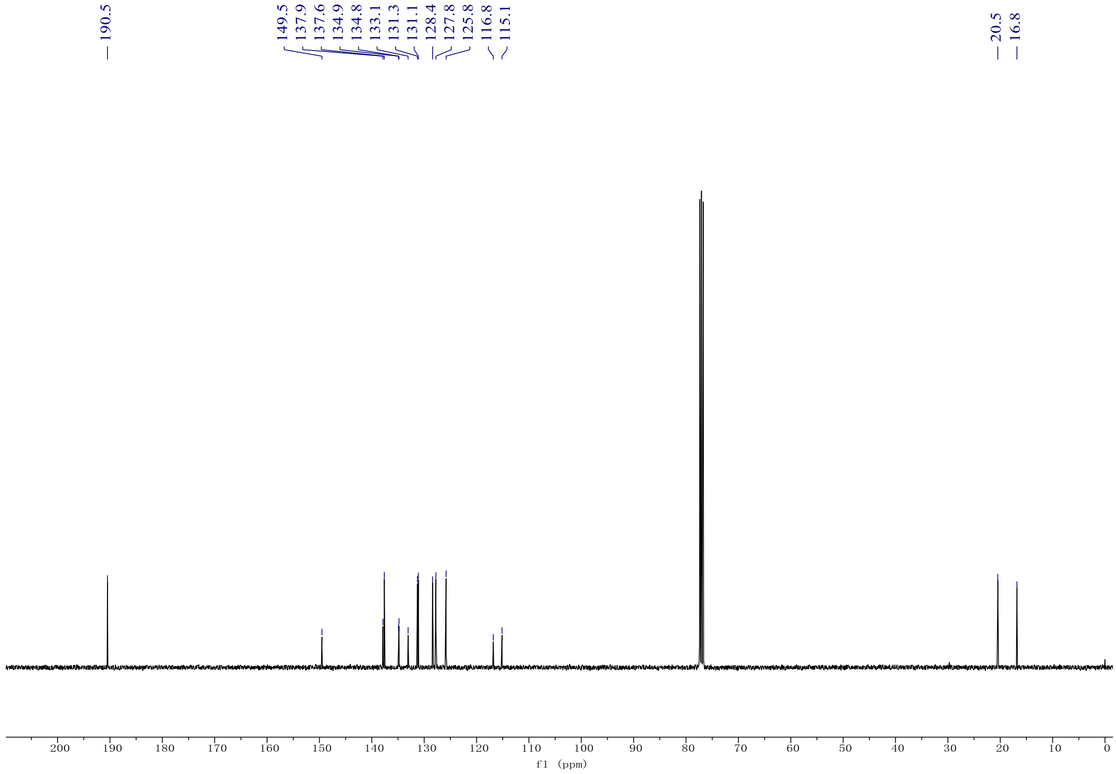

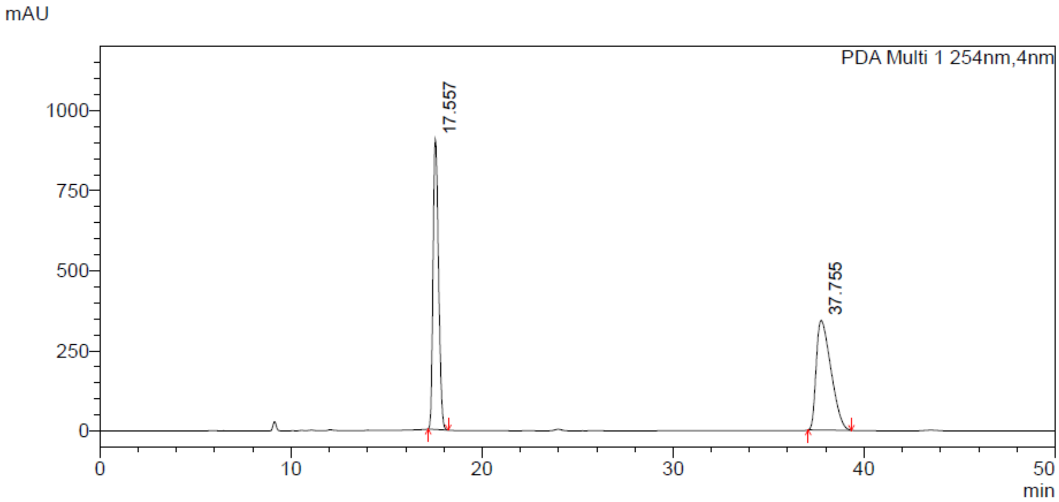

PDA Ch1 254nm

Ret. Time	Height	Area	Area%
17.557	905469	18097169	49.696
37.755	342350	18318510	50.304
	1247819	36415679	100.000

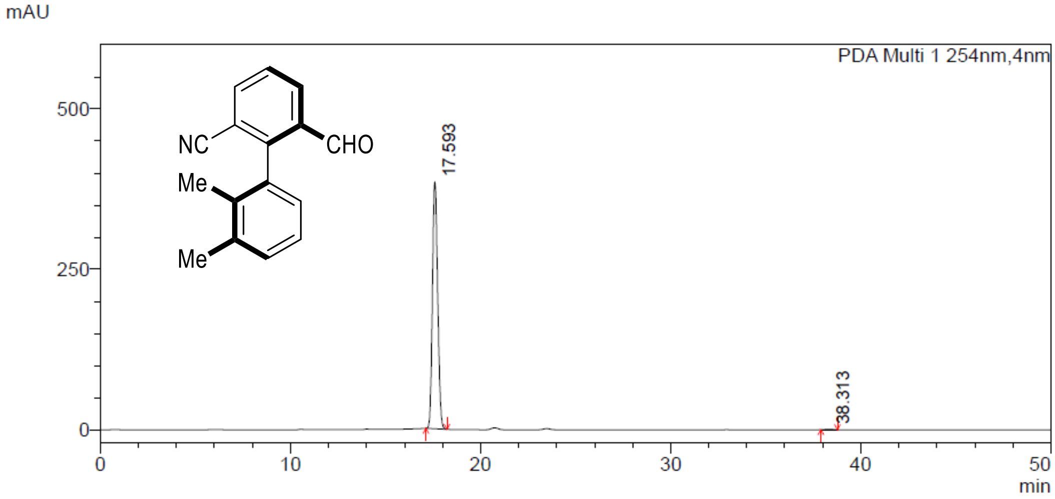

PDA Ch1 254nm

Ret. Time	Height	Area	Area%
17.593	384970	7403407	99.775
38.313	540	16695	0.225
	385510	7420102	100.000

(*S*)-6-formyl-2',4'-dimethyl-[1,1'-biphenyl]-2-carbonitrile (3c)

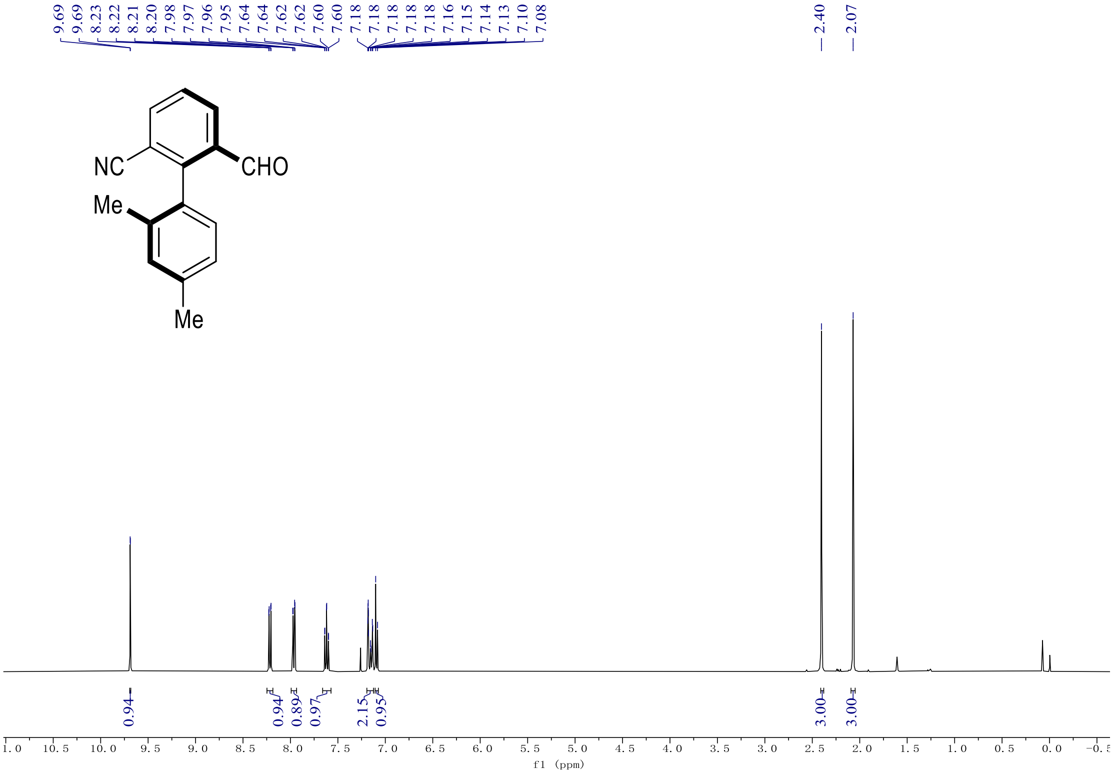

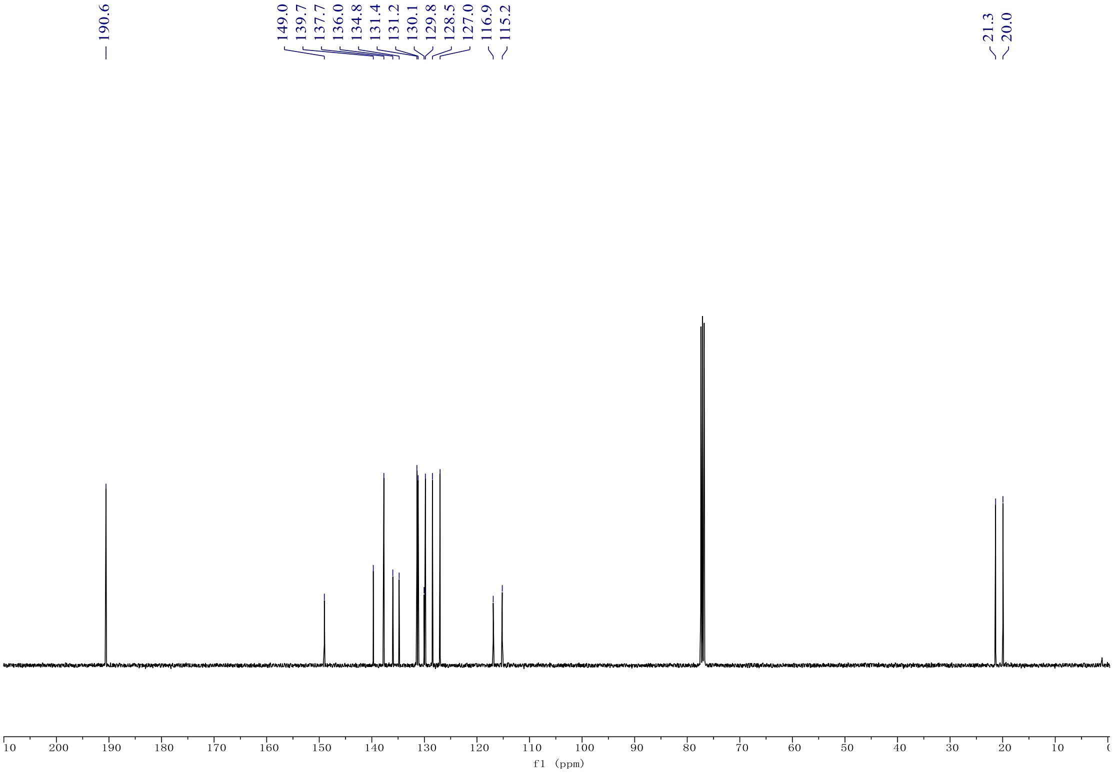

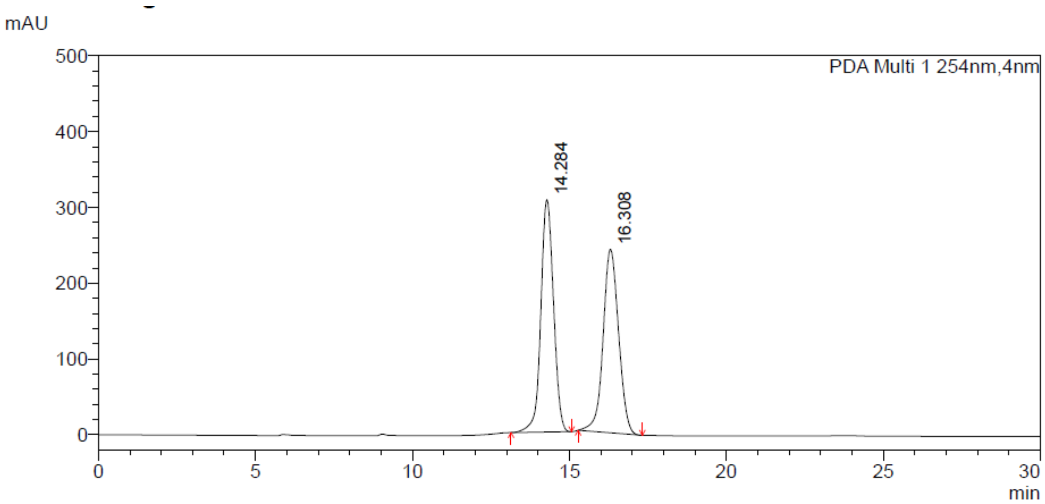

PDA Ch1 254nm

Ret. Time	Height	Area	Area%
14.284	306738	8645192	50.965
16.308	242257	8317644	49.035
	548995	16962836	100.000

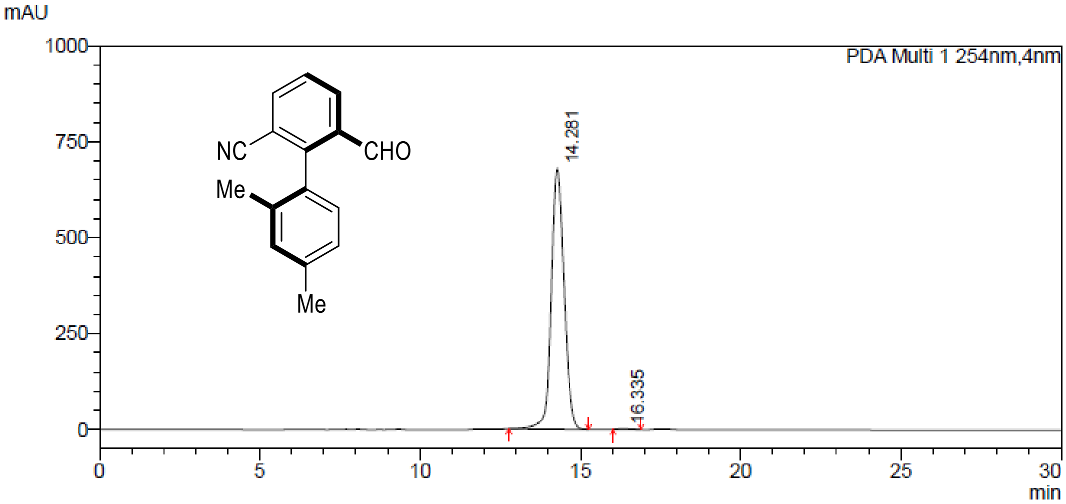

PDA Ch1 254nm

Ret. Time	Height	Area	Area%
14.281	678070	18954446	99.689
16.335	2198	59167	0.311
	680268	19013613	100.000

(*S*)-6-formyl-2',4'-dimethyl-[1,1'-biphenyl]-2-carbonitrile (3d)

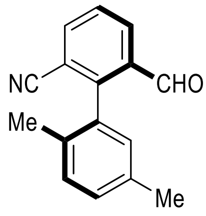

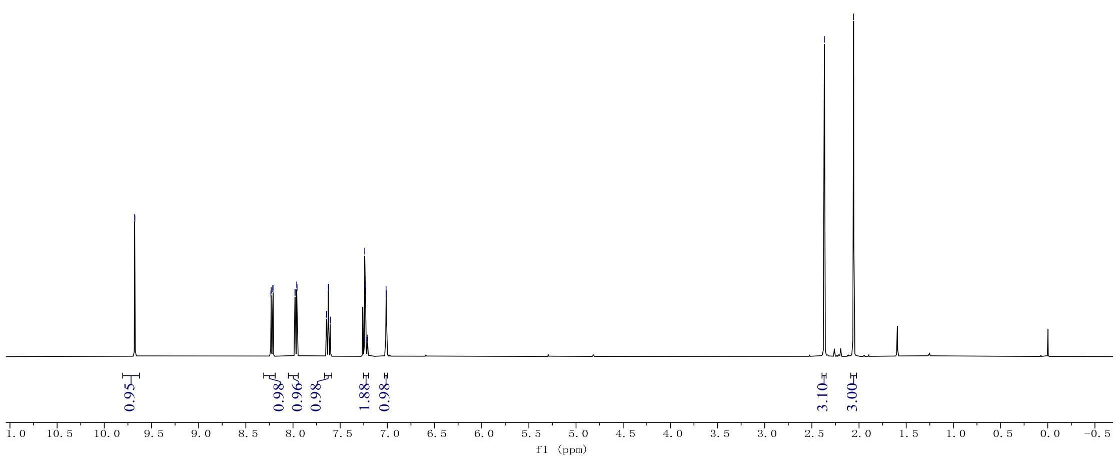

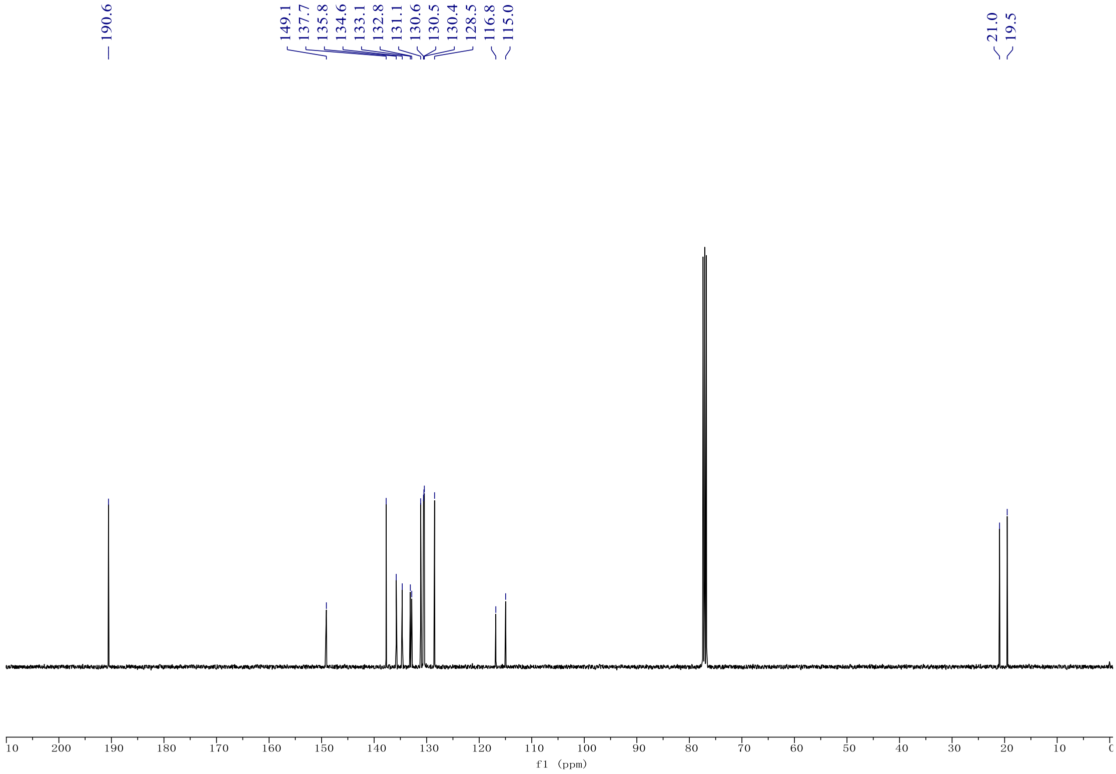

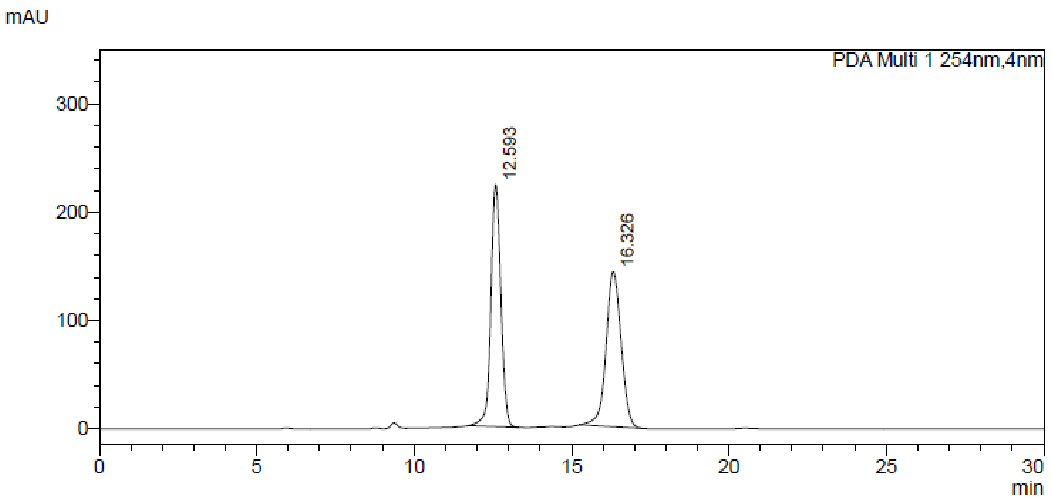

PDA Ch1 254nm

Ret. Time	Height	Area	Area%
12.593	223391	4915630	50.775
16.326	143171	4765488	49.225
	366562	9681119	100.000

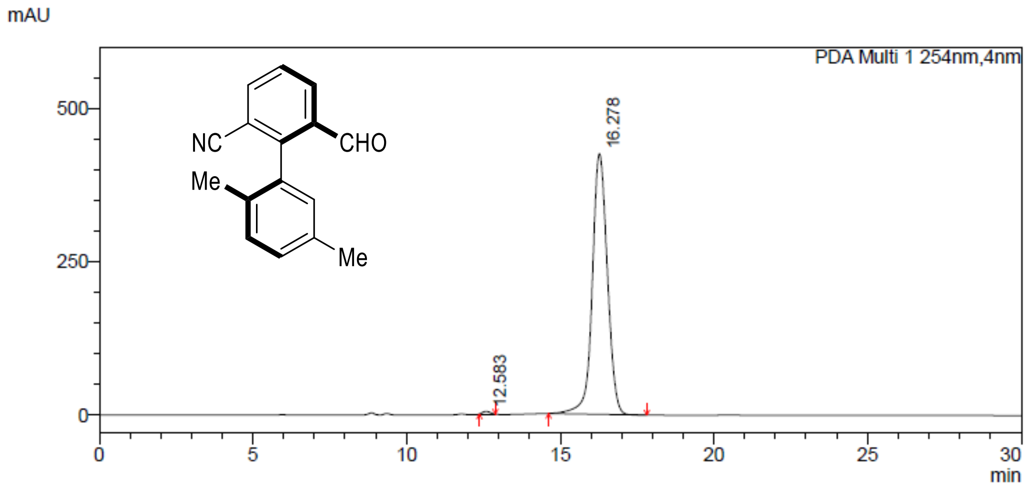

PDA Ch1 254nm

Ret. Time	Height	Area	Area%
12.583	4223	71981	0.495
16.278	424853	14482868	99.505
	429076	14554849	100.000

(*S*)-6-formyl-2',4',5'-trimethyl-[1,1'-biphenyl]-2-carbonitrile (3e)

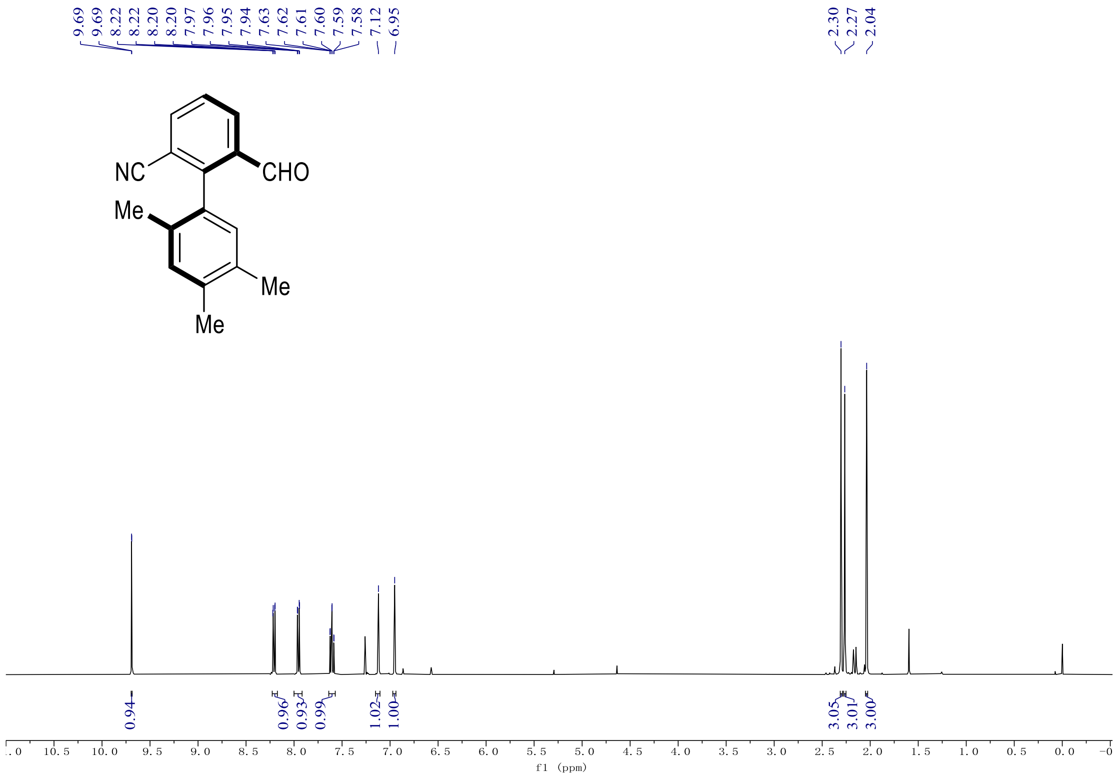

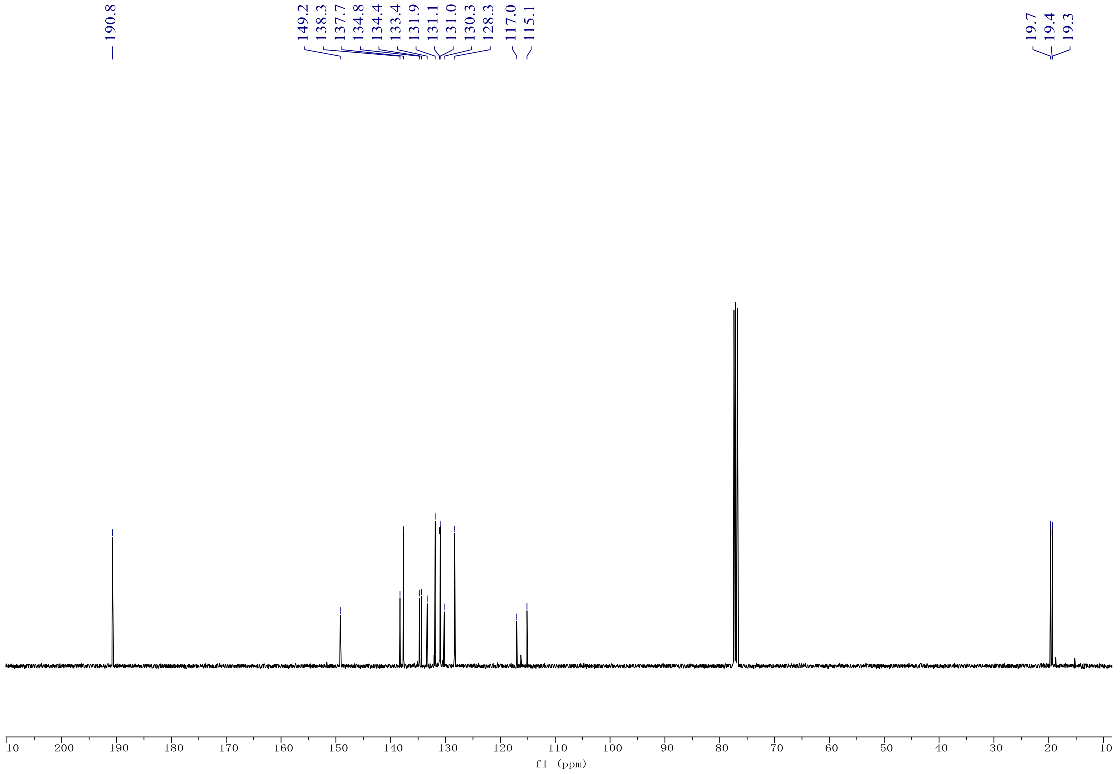

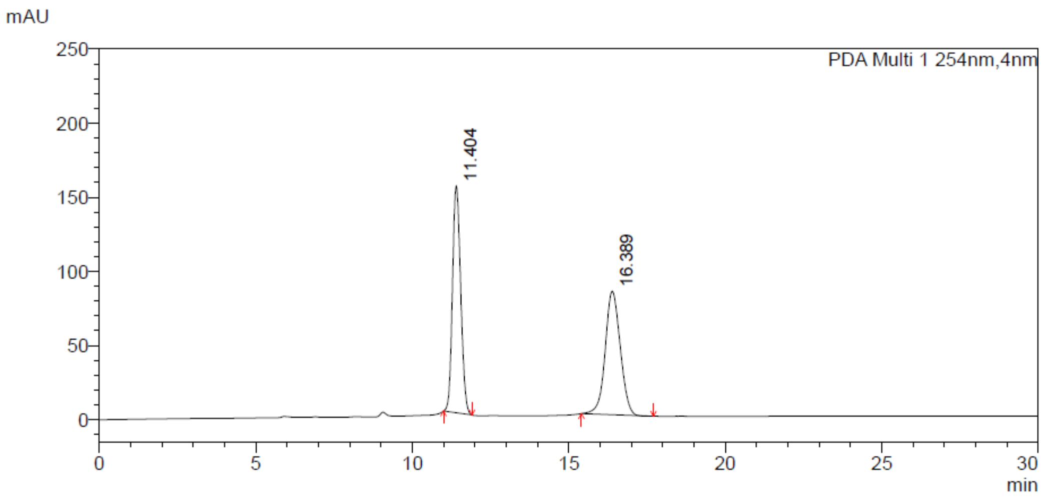

PDA Ch1 254nm

Ret. Time	Height	Area	Area%
11.404	153032	2793102	50.038
16.389	83251	2788910	49.962
	236283	5582012	100.000

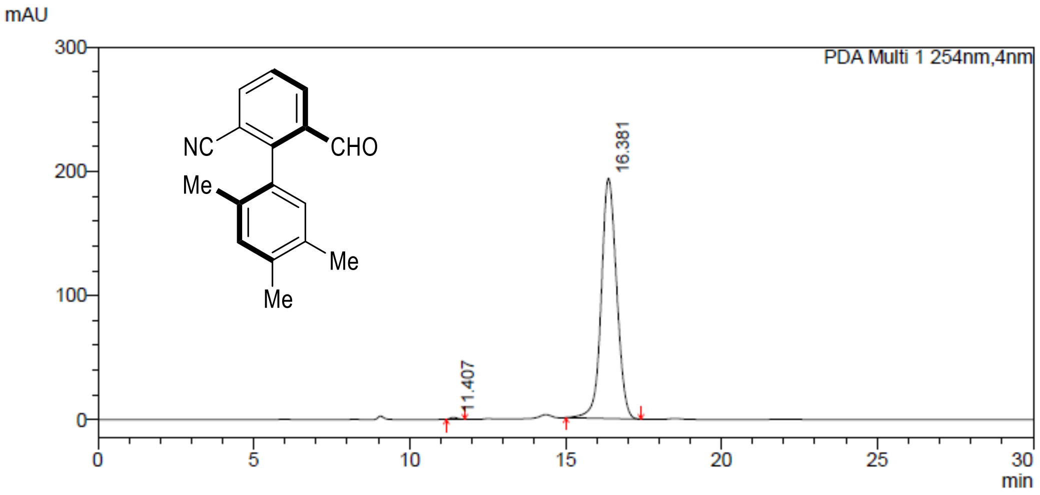

PDA Ch1 254nm

Ret. Time	Height	Area	Area%
11.407	1398	22673	0.341
16.381	193263	6624546	99.659
	194661	6647219	100.000

(*S*)-6-formyl-4'-methoxy-2'-methyl-[1,1'-biphenyl]-2-carbonitrile (3f)

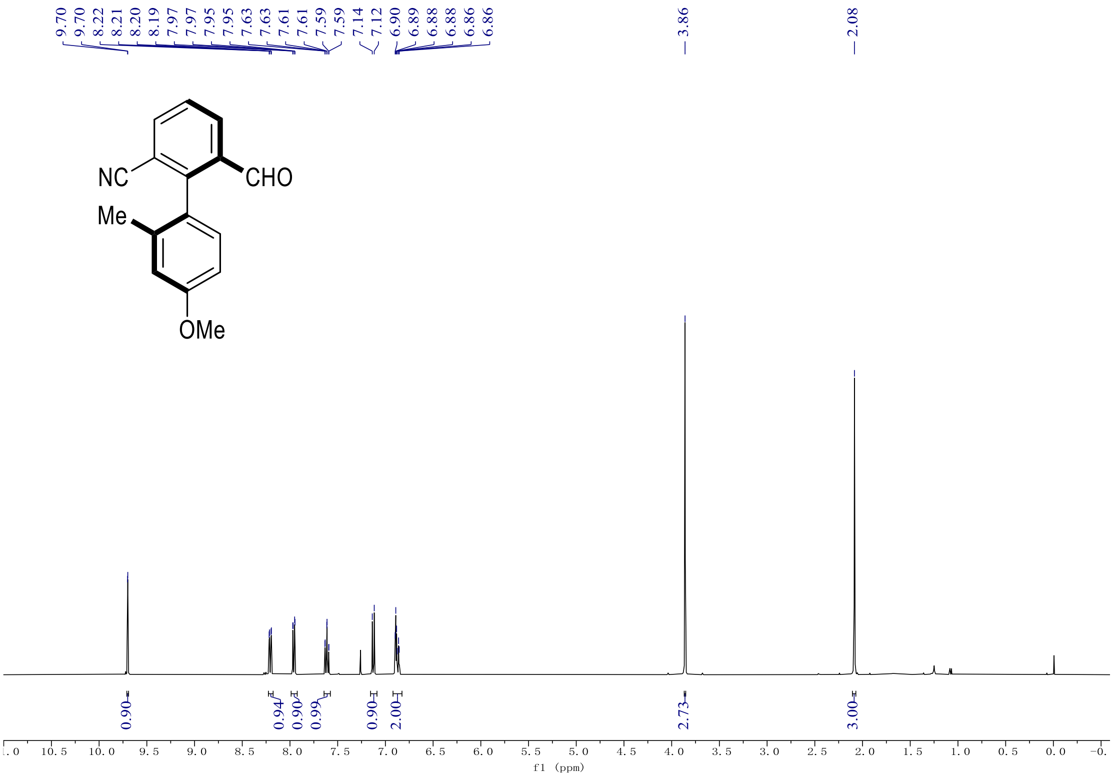

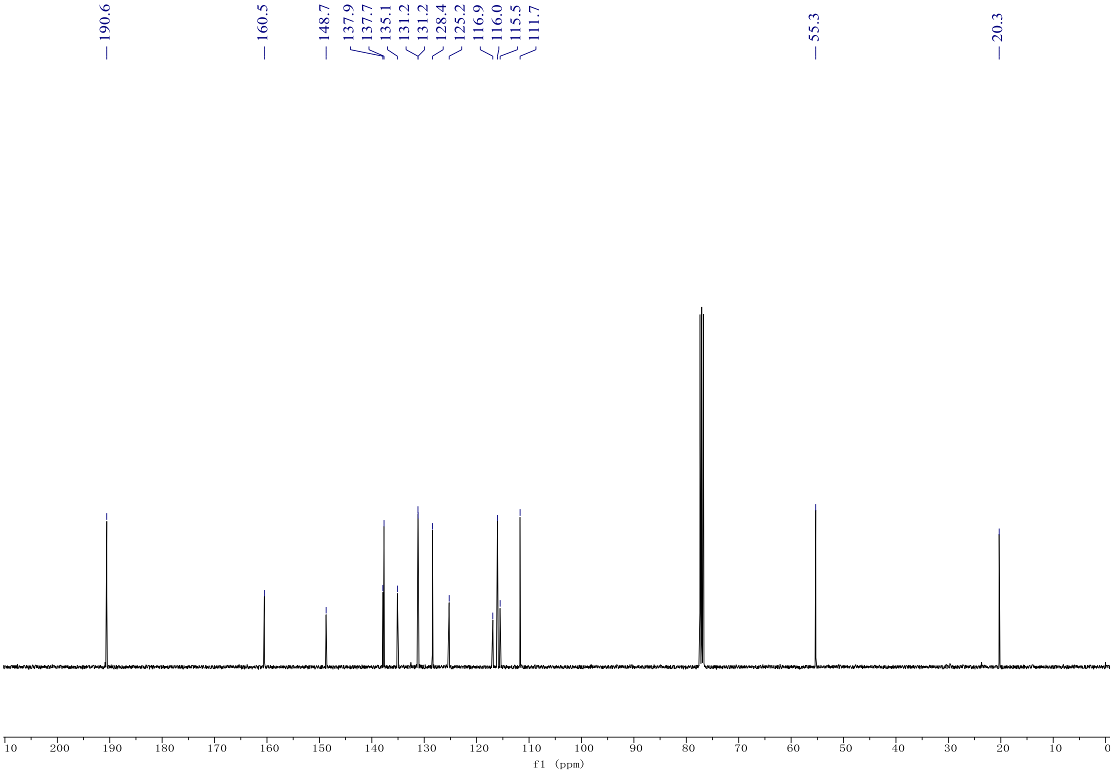

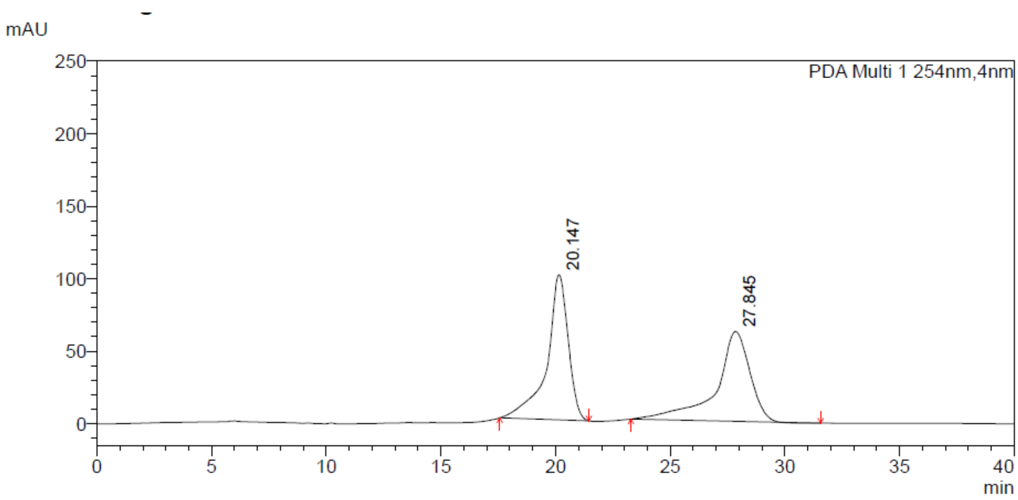

PDA Ch1 254nm

Ret. Time	Height	Area	Area%
20.147	99981	6485604	50.444
27.845	61878	6371325	49.556
	161859	12856930	100.000

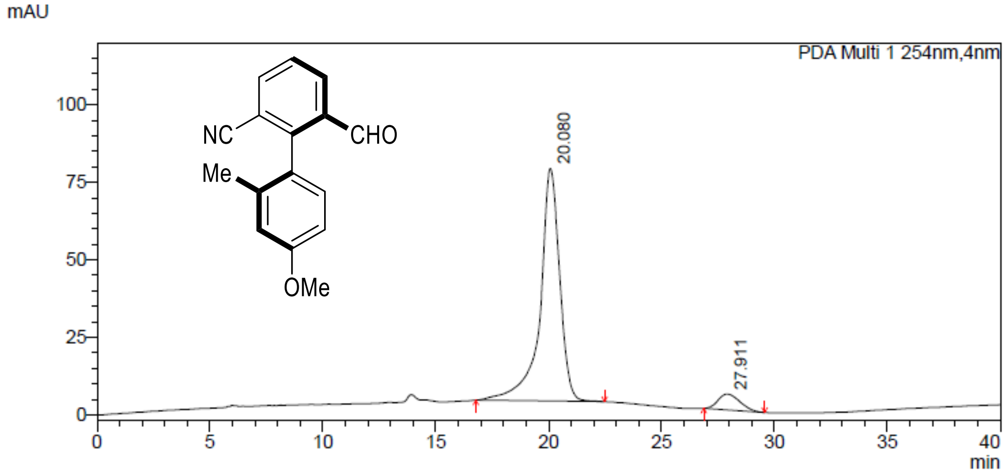

PDA Ch1 254nm

Ret. Time	Height	Area	Area%
20.080	74967	4545426	92.863
27.911	5168	349320	7.137
	80135	4894746	100.000

(*S*)-4'-fluoro-6-formyl-2'-methyl-[1,1'-biphenyl]-2-carbonitrile (3g)

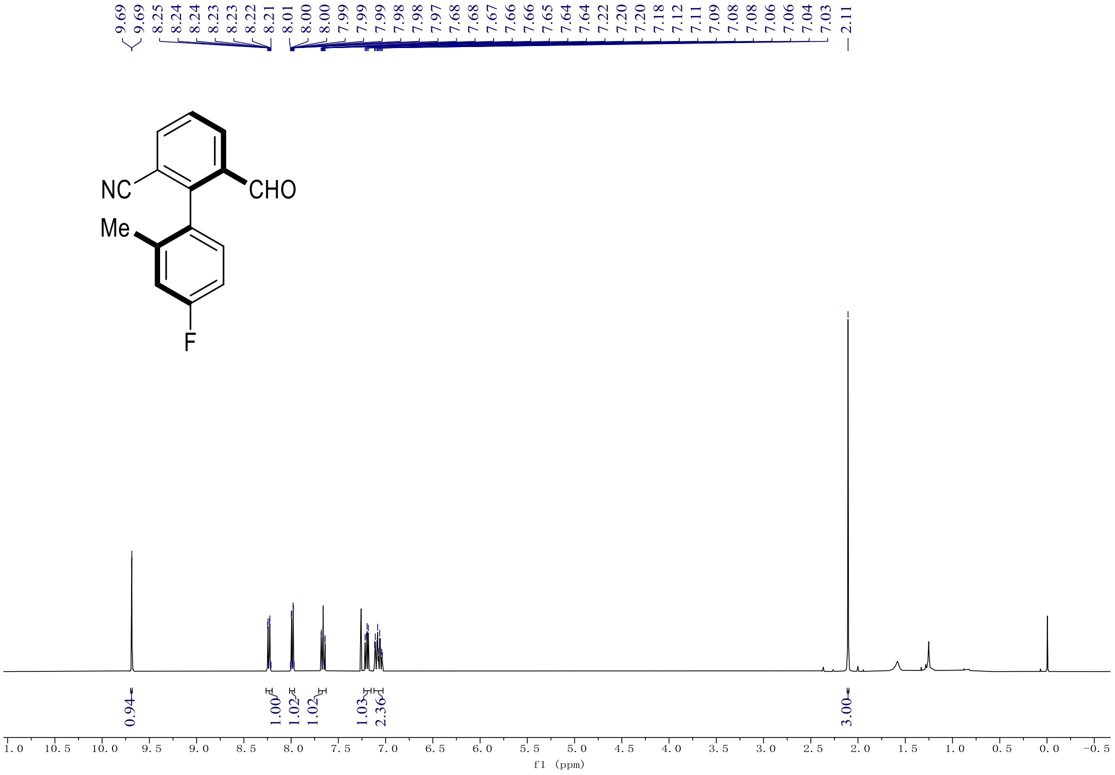

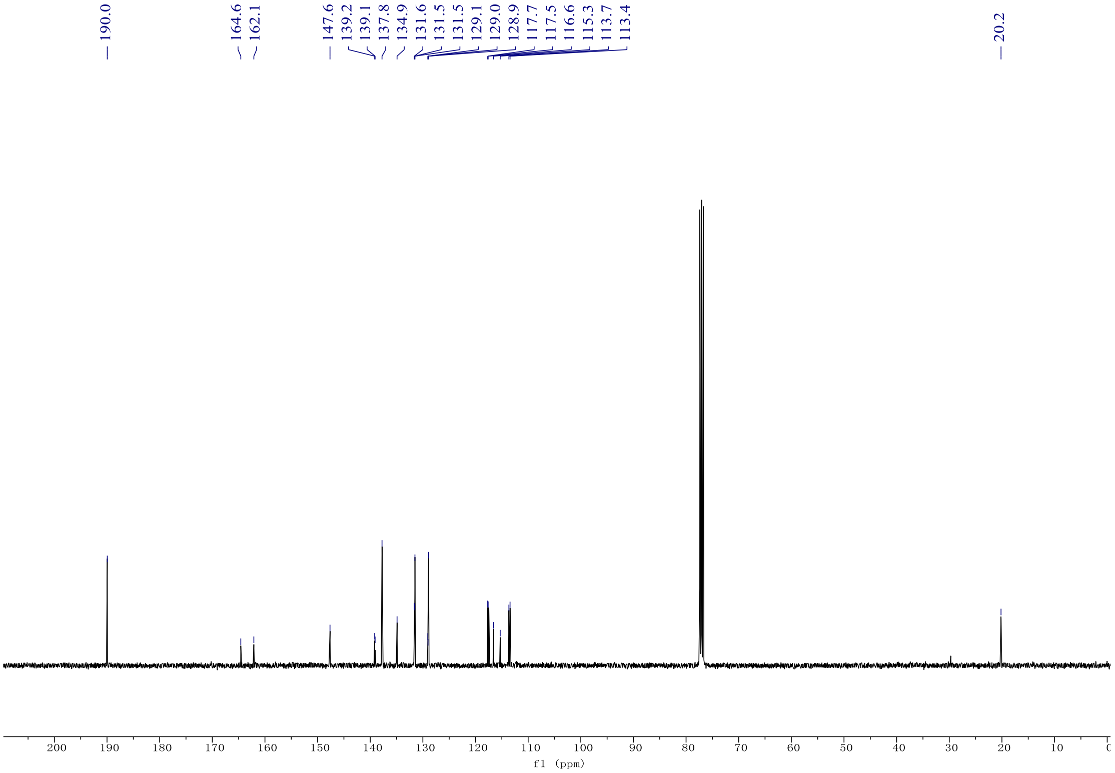

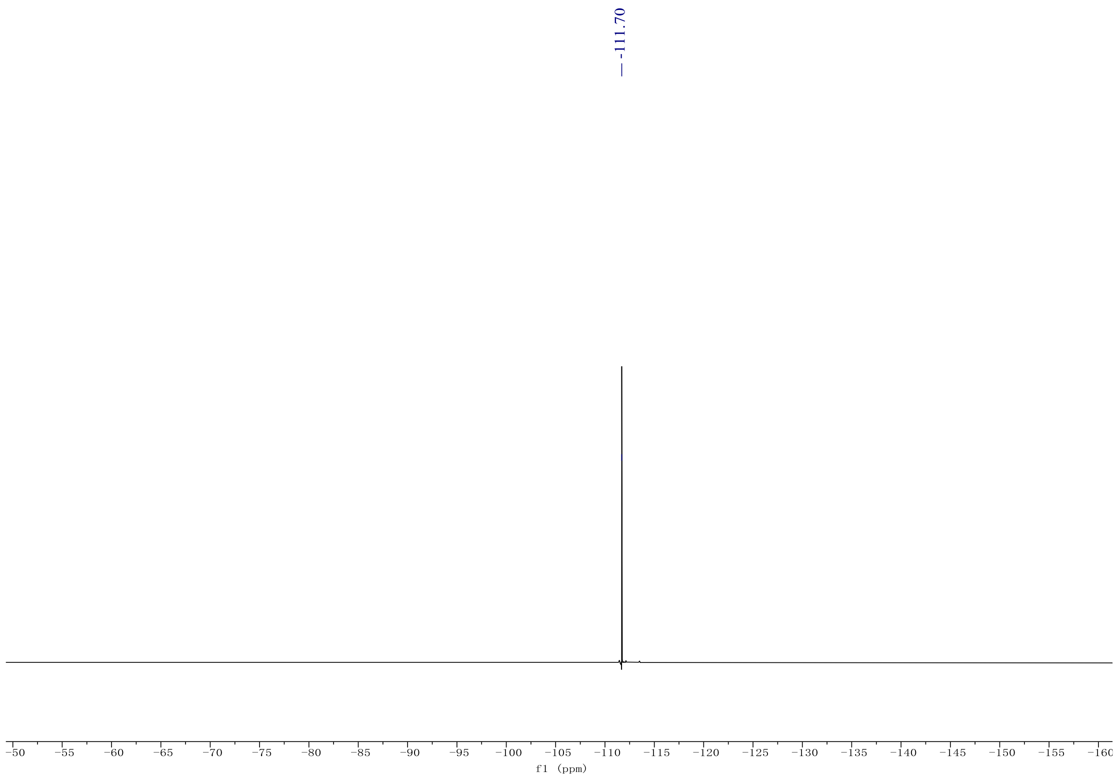
-111.70
-50
-55
-60
-65
-70
-75
-80
-85
-90
-95
-100
-105
-110
-115
-120
-125
-130
-135
-140
-145
-150
-155
-160
f1 (ppm)

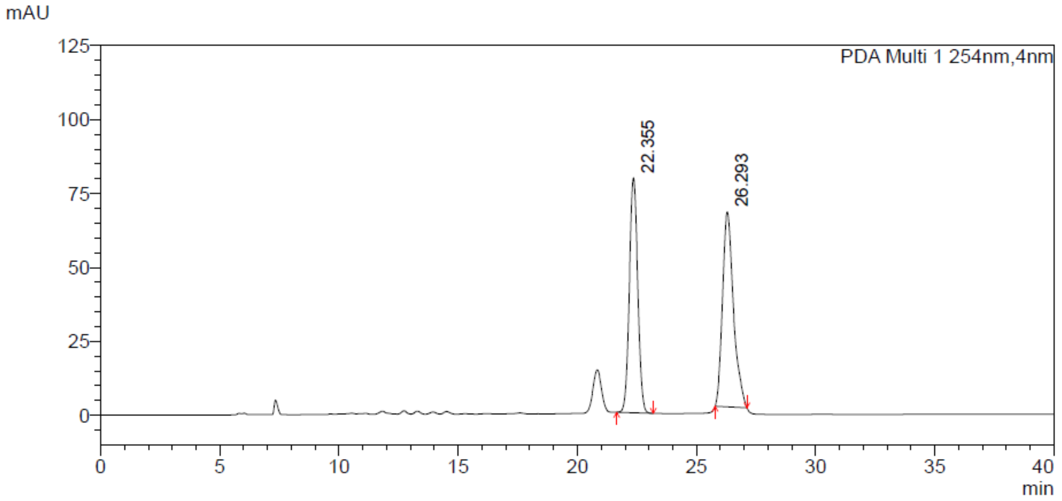

PDA Ch1 254nm

Ret. Time	Height	Area	Area%
22.355	79413	1938051	47.898
26.293	65916	2108177	52.102
	145329	4046228	100.000

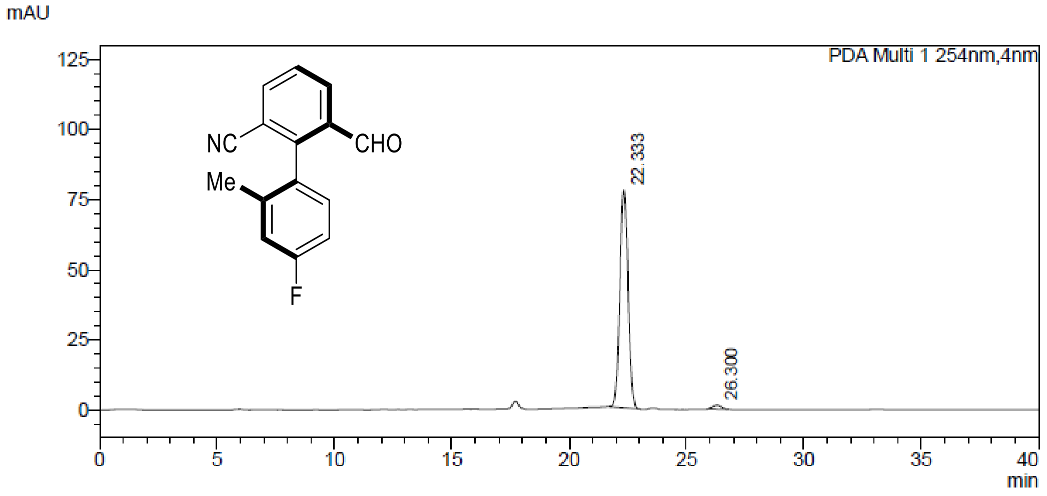

PDA Ch1 254nm

Ret. Time	Height	Area	Area%
22.333	77437	1863690	98.015
26.300	1442	37737	1.985
	78879	1901428	100.000

(*S*)-5'-fluoro-6-formyl-2'-methyl-[1,1'-biphenyl]-2-carbonitrile (3h)

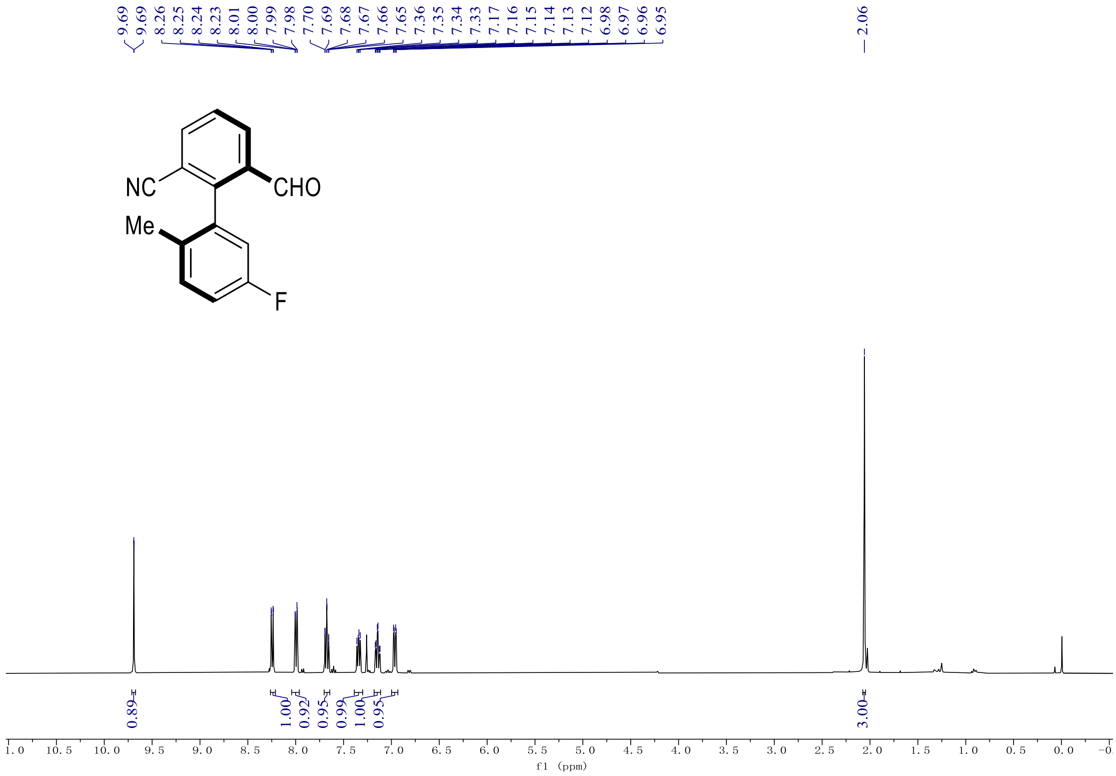

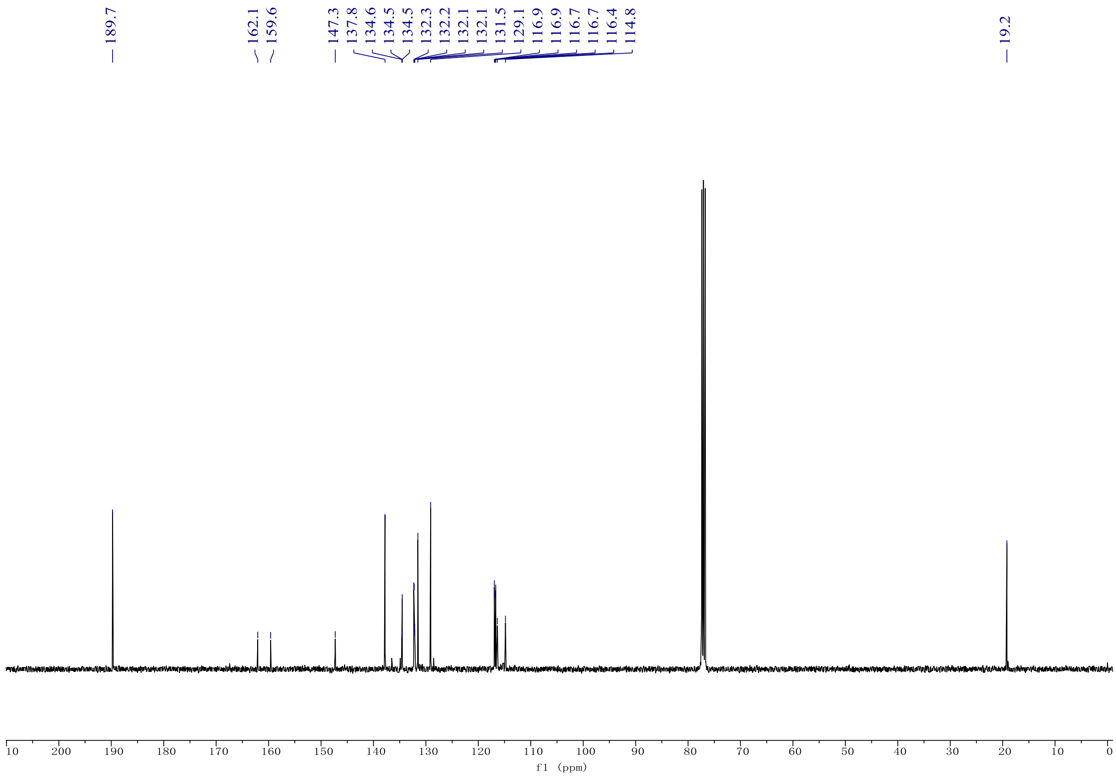

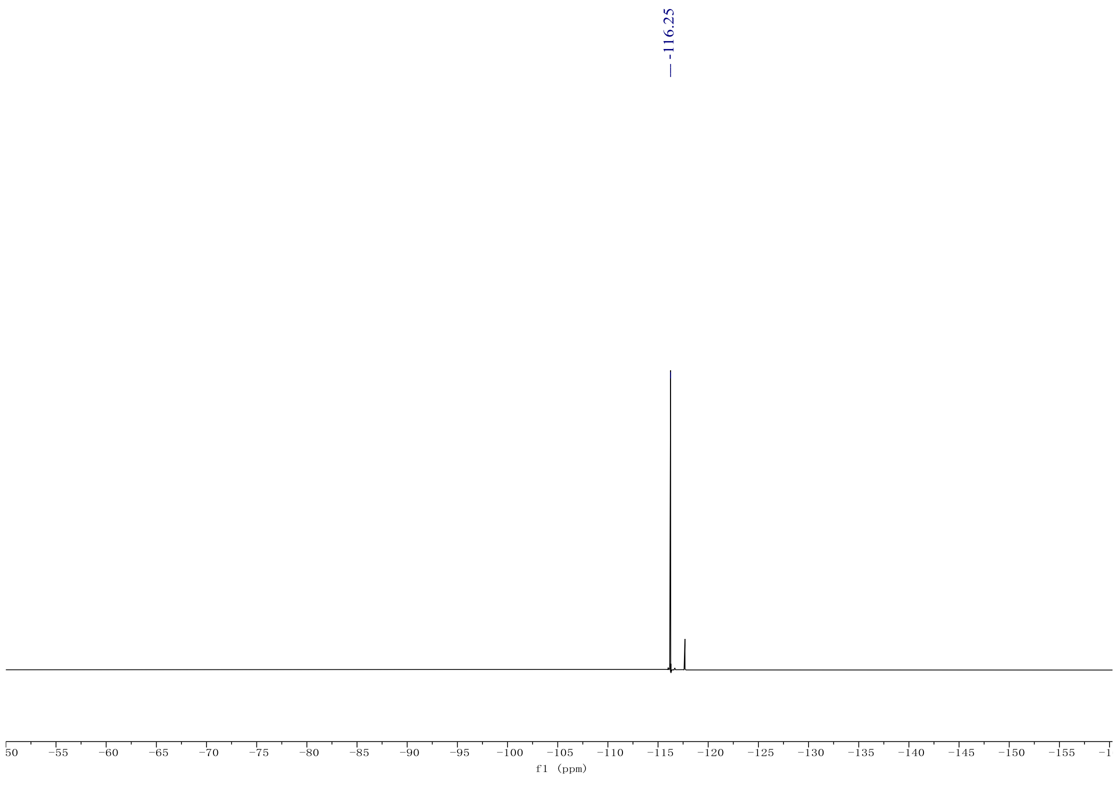

-116.25
50
−55
−60
−65
−70
−75
−80
−85
−90
−95
−100
−105
−110
−115
−120
−125
−130
−135
−140
−145
−150
−155
−1
f1 (ppm)

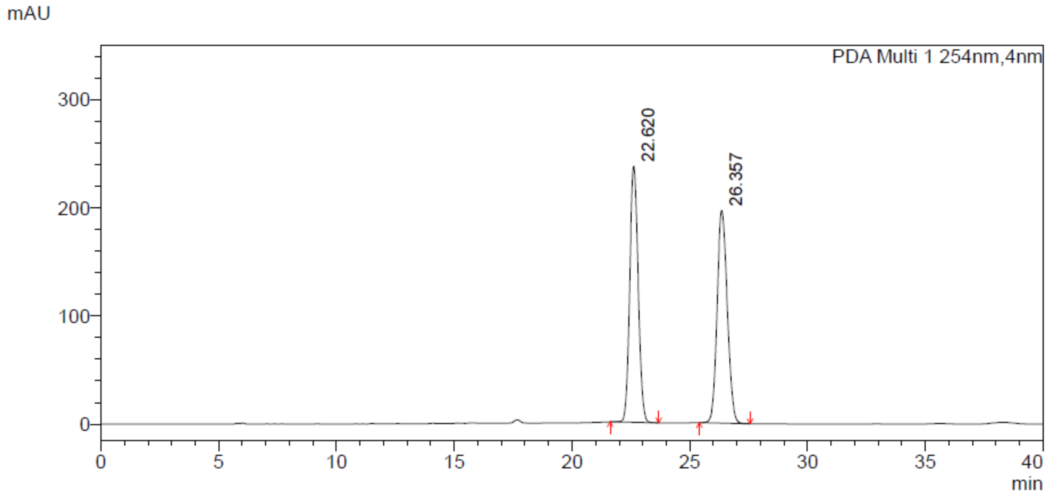

PDA Ch1 254nm

Ret. Time	Height	Area	Area%
22.620	236842	5880692	49.923
26.357	196788	5898927	50.077
	433629	11779619	100.000

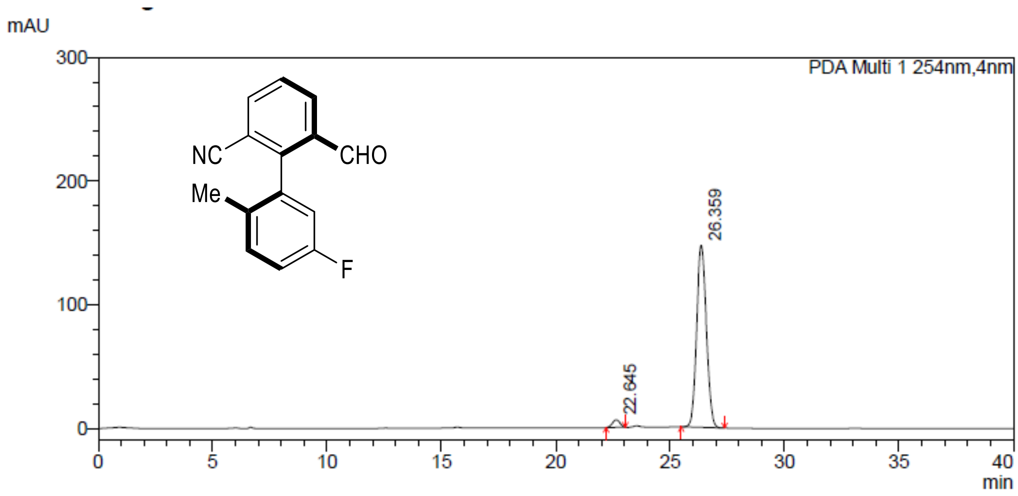

PDA Ch1 254nm

Ret. Time	Height	Area	Area%
22.645	6100	138785	3.092
26.359	147214	4349522	96.908
	153314	4488306	100.000

(*S*)-3'-chloro-6-formyl-2'-methyl-[1,1'-biphenyl]-2-carbonitrile (3i)

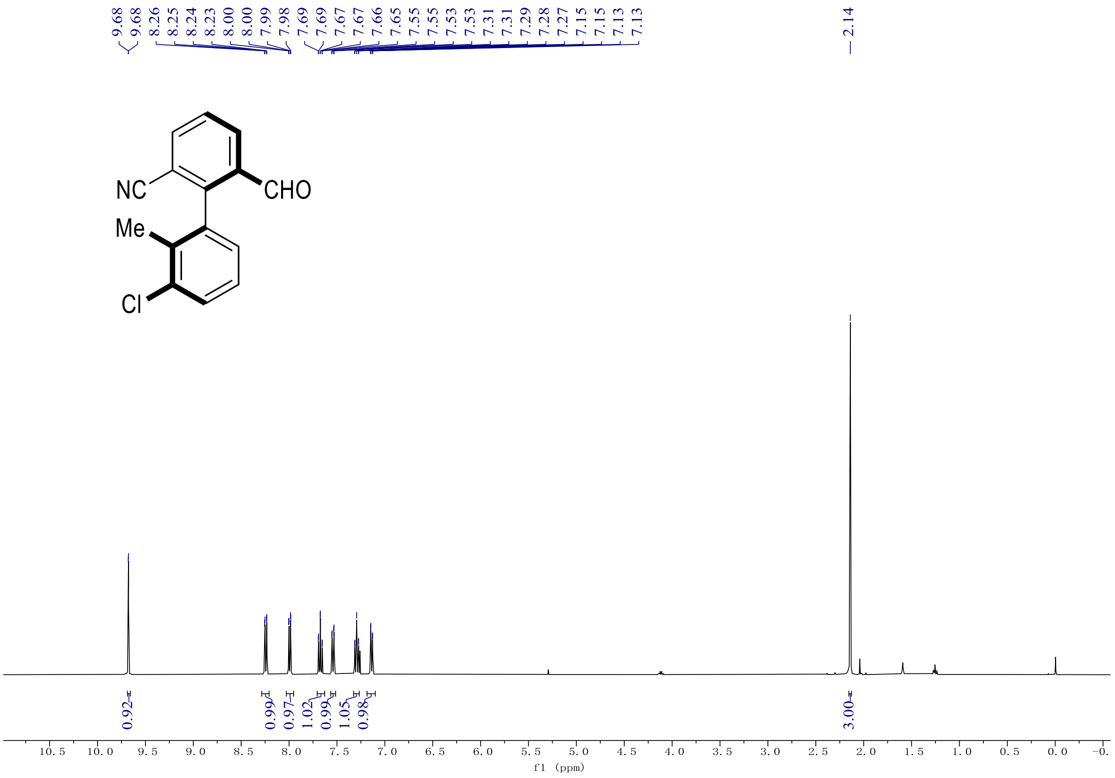

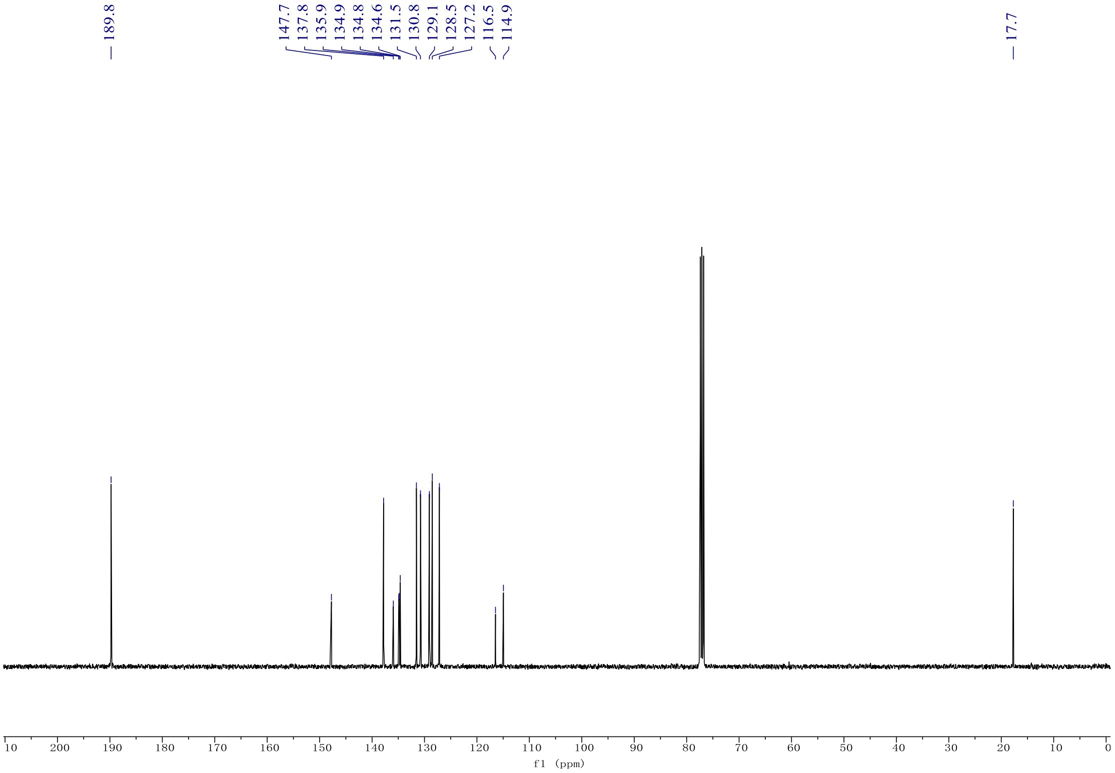

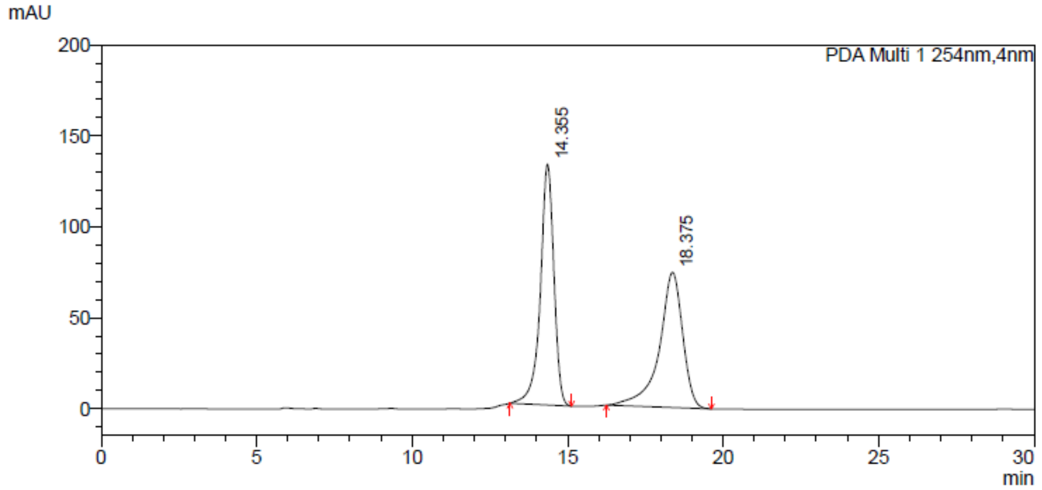

PDA Ch1 254nm

Ret. Time	Height	Area	Area%
14.355	132328	4015605	50.848
18.375	74302	3881717	49.152
	206629	7897322	100.000

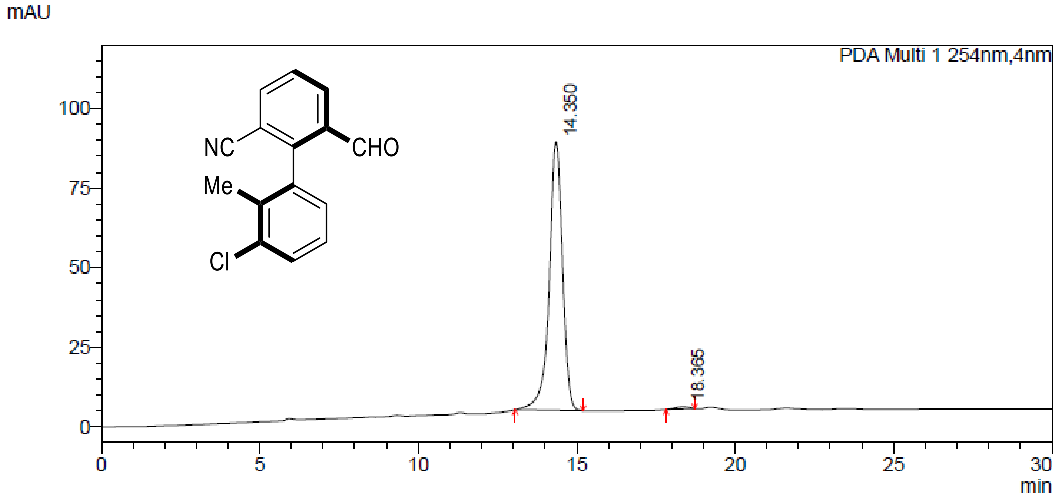

PDA Ch1 254nm

Ret. Time	Height	Area	Area%
14.350	84326	2467723	99.101
18.365	741	22395	0.899
	85067	2490118	100.000

(*S*)-4'-chloro-6-formyl-2'-methyl-[1,1'-biphenyl]-2-carbonitrile (3j)

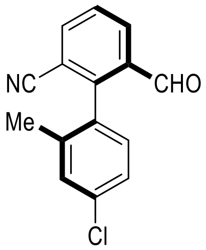

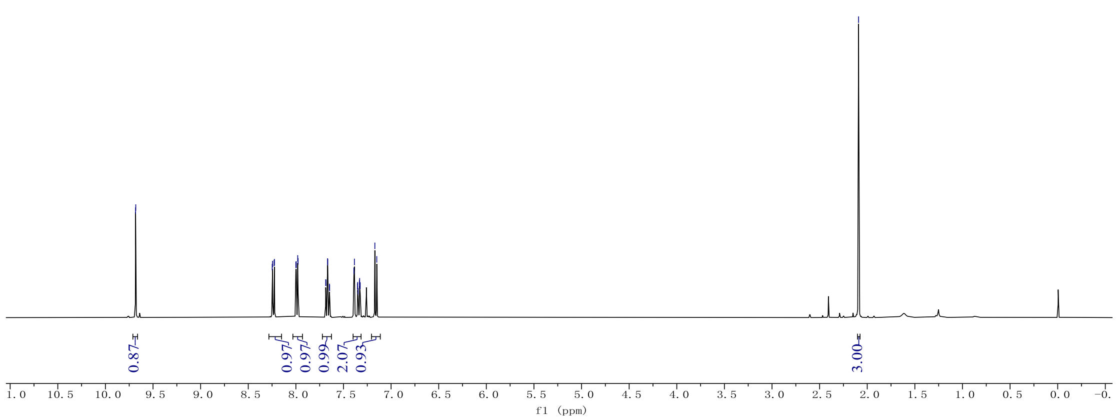

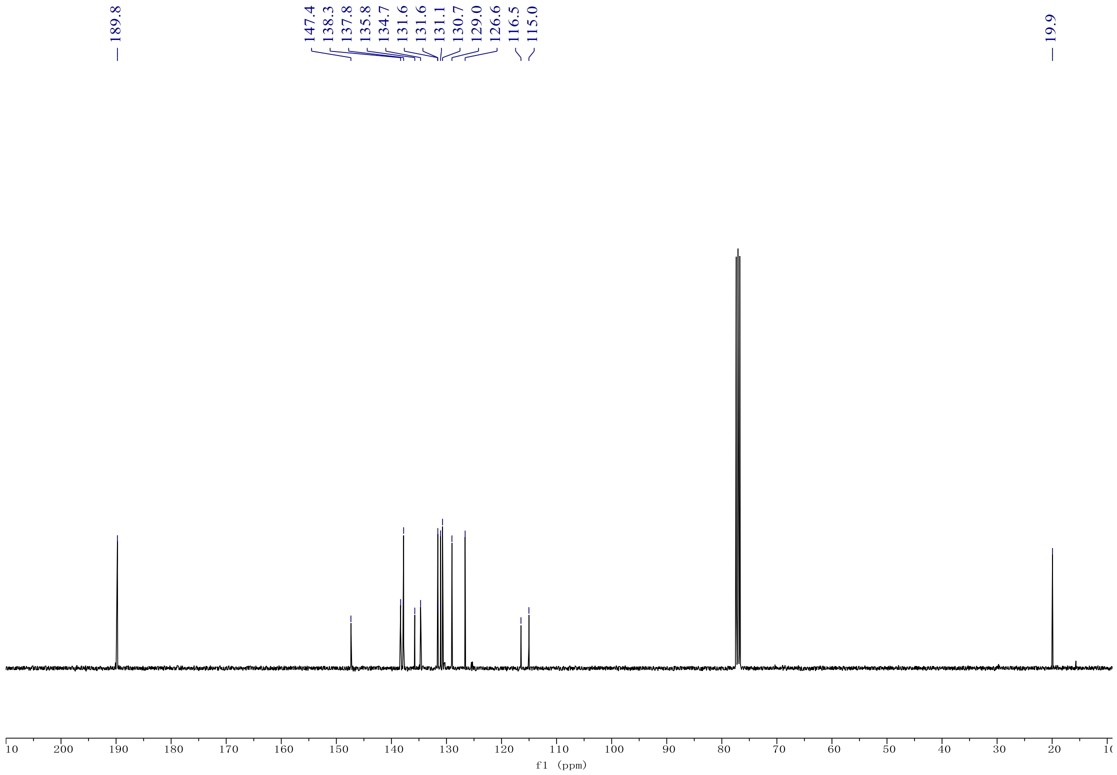

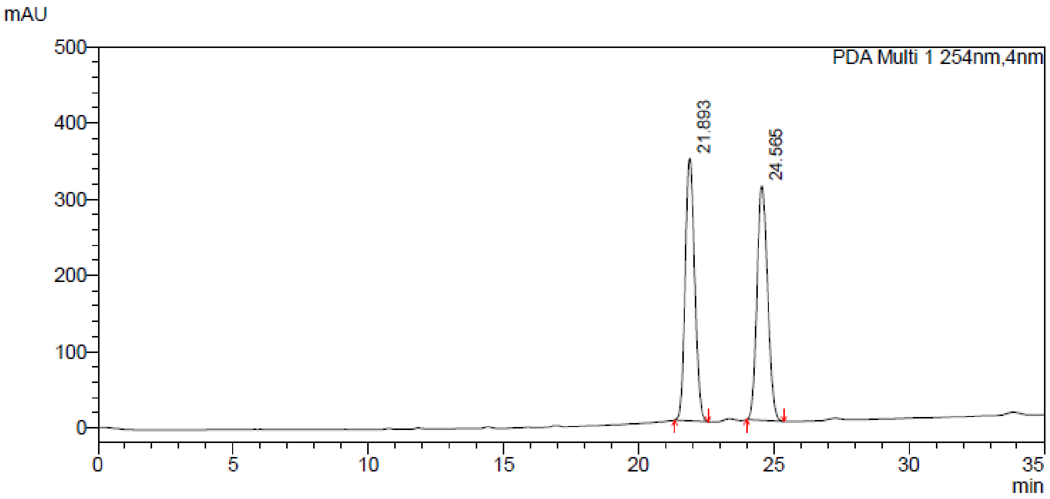

PDA Ch1 254nm

Ret. Time	Height	Area	Area%
21.893	344432	8429861	49.982
24.565	307576	8436095	50.018
	652008	16865956	100.000

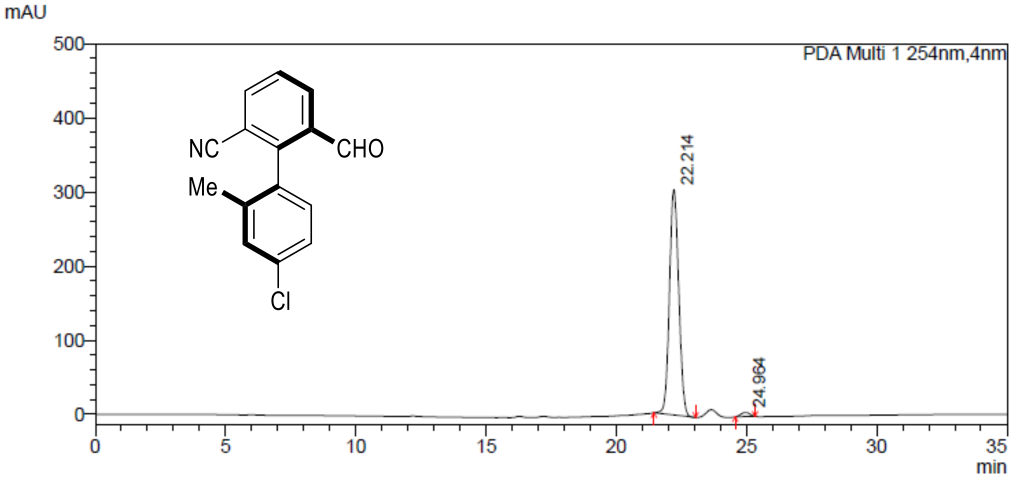

PDA Ch1 254nm

Ret. Time	Height	Area	Area%
22.214	303683	7537551	98.264
24.964	5601	133183	1.736
	309285	7670735	100.000

(*S*)-5'-chloro-6-formyl-2'-methyl-[1,1'-biphenyl]-2-carbonitrile (3k)

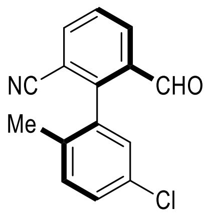

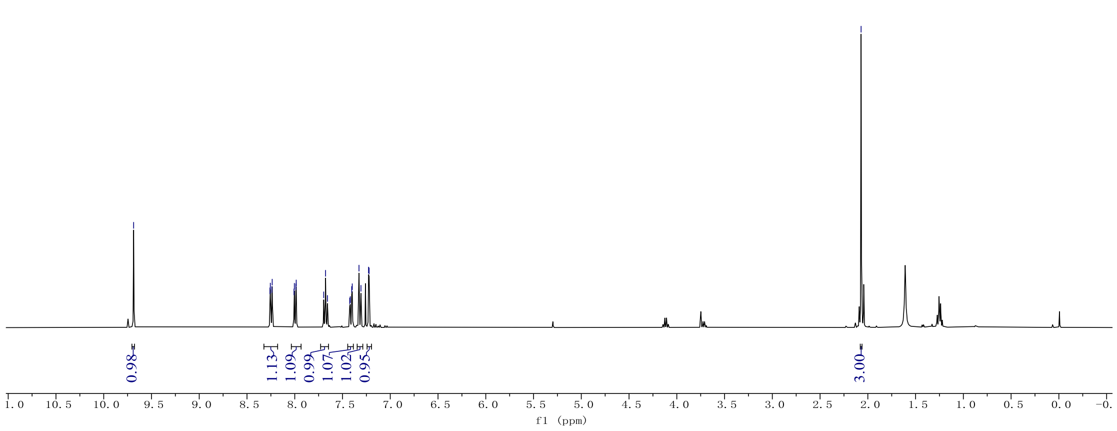

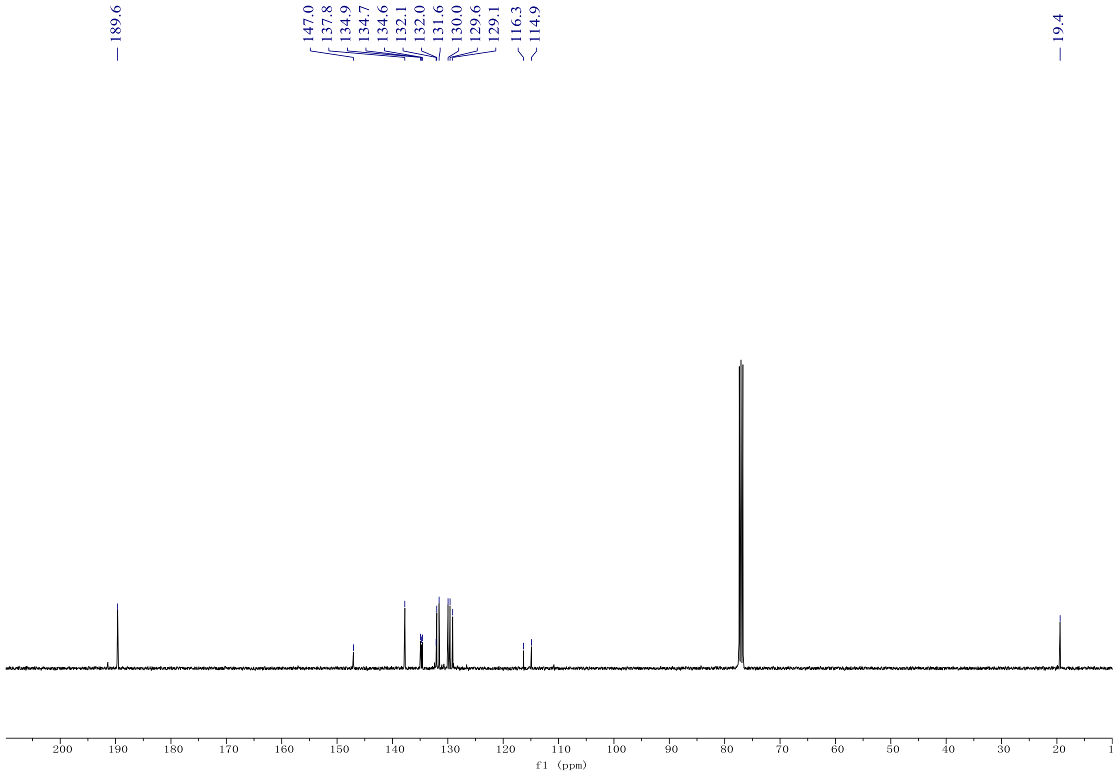

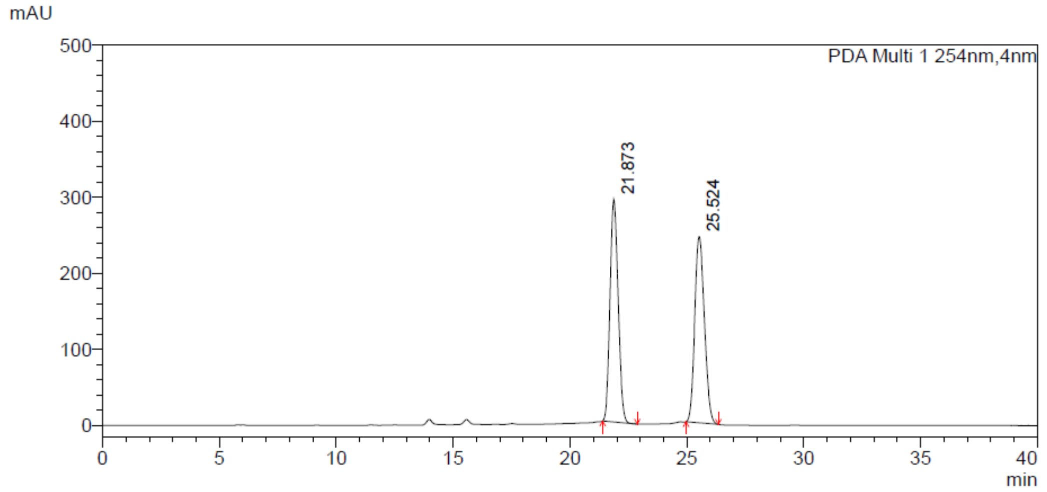

PDA Ch1 254nm

Ret. Time	Height	Area	Area%
21.873	292677	7076957	50.046
25.524	245195	7063907	49.954
	537872	14140864	100.000

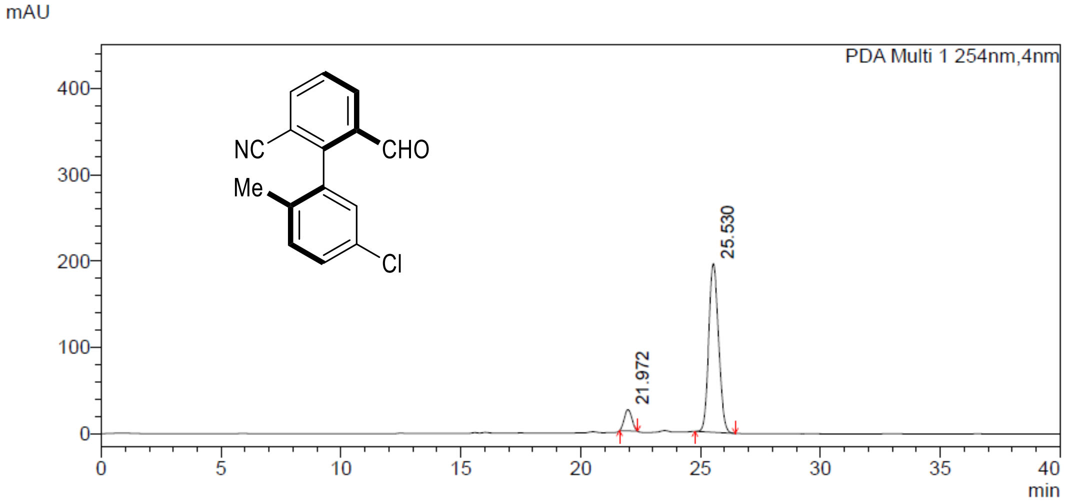

PDA Ch1 254nm

Ret. Time	Height	Area	Area%
21.972	24687	545823	8.889
25.530	195253	5594575	91.111
	219940	6140397	100.000

(*S*)-2'-ethyl-6-formyl-[1,1'-biphenyl]-2-carbonitrile (3l)

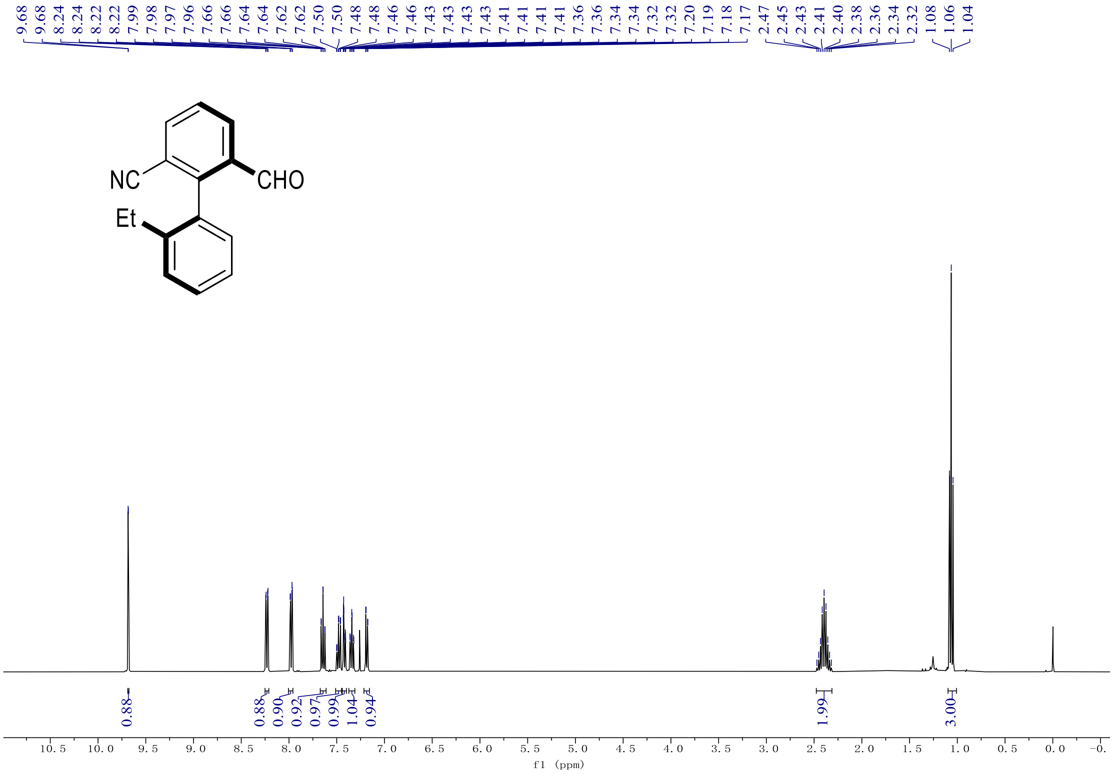

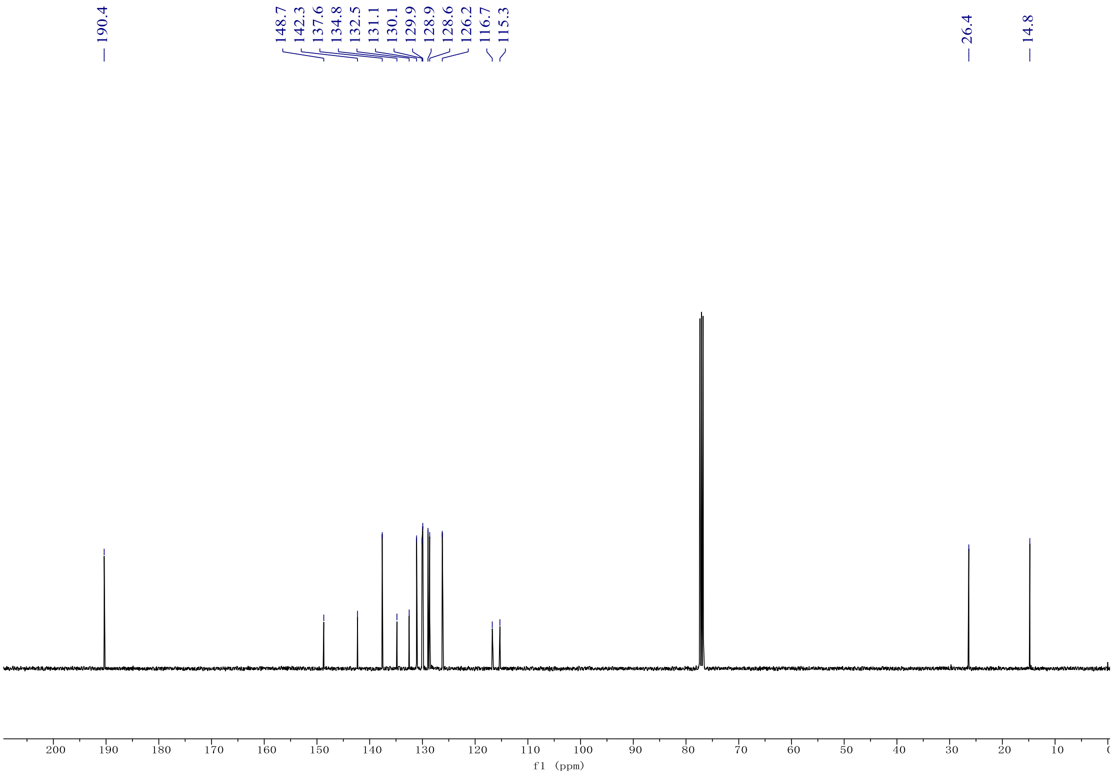

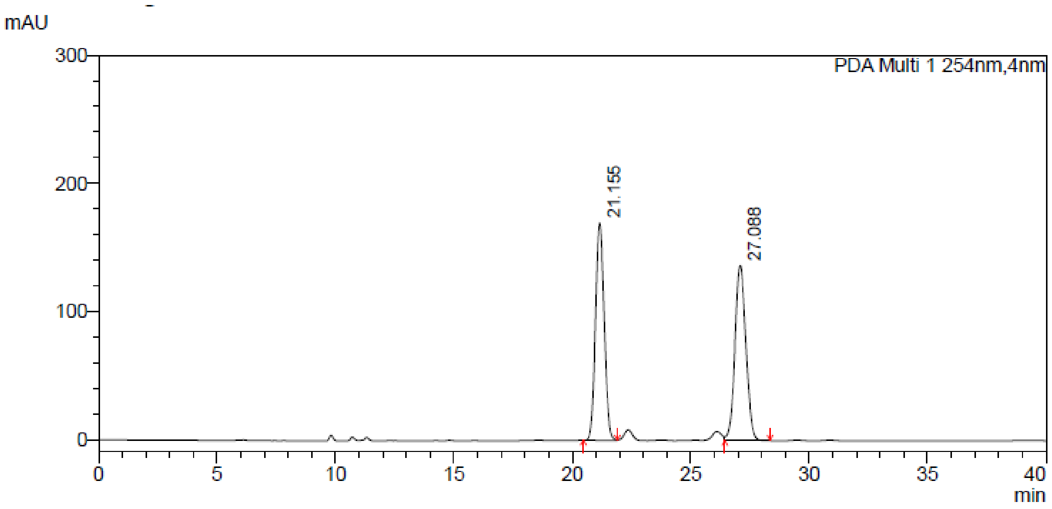

PDA Ch1 254nm

Ret. Time	Height	Area	Area%
21.155	169554	4373048	50.017
27.088	136551	4370065	49.983
	306104	8743113	100.000

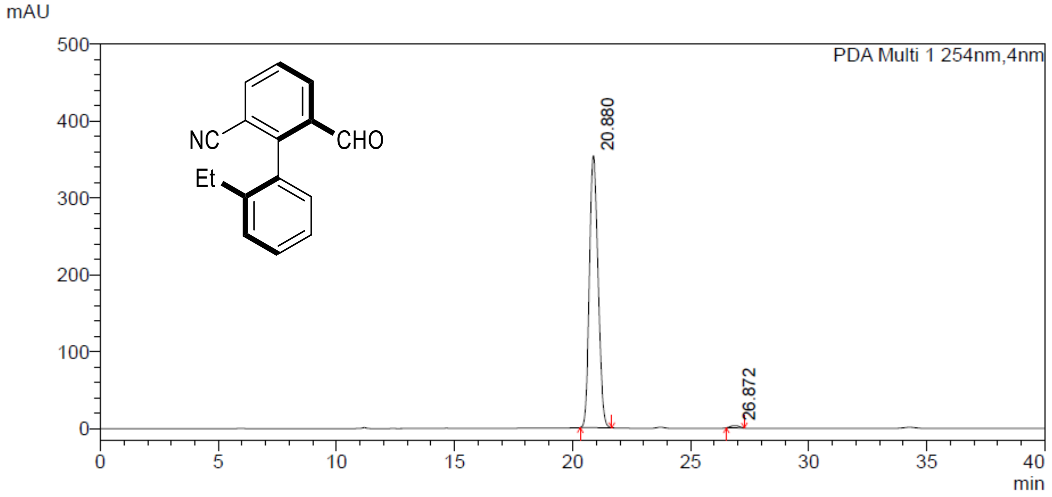

PDA Ch1 254nm

Ret. Time	Height	Area	Area%
20.880	353773	9039449	99.188
26.872	2982	74006	0.812
	356755	9113456	100.000

(*S*)-6-formyl-2'-(methylthio)-[1,1'-biphenyl]-2-carbonitrile (3m)

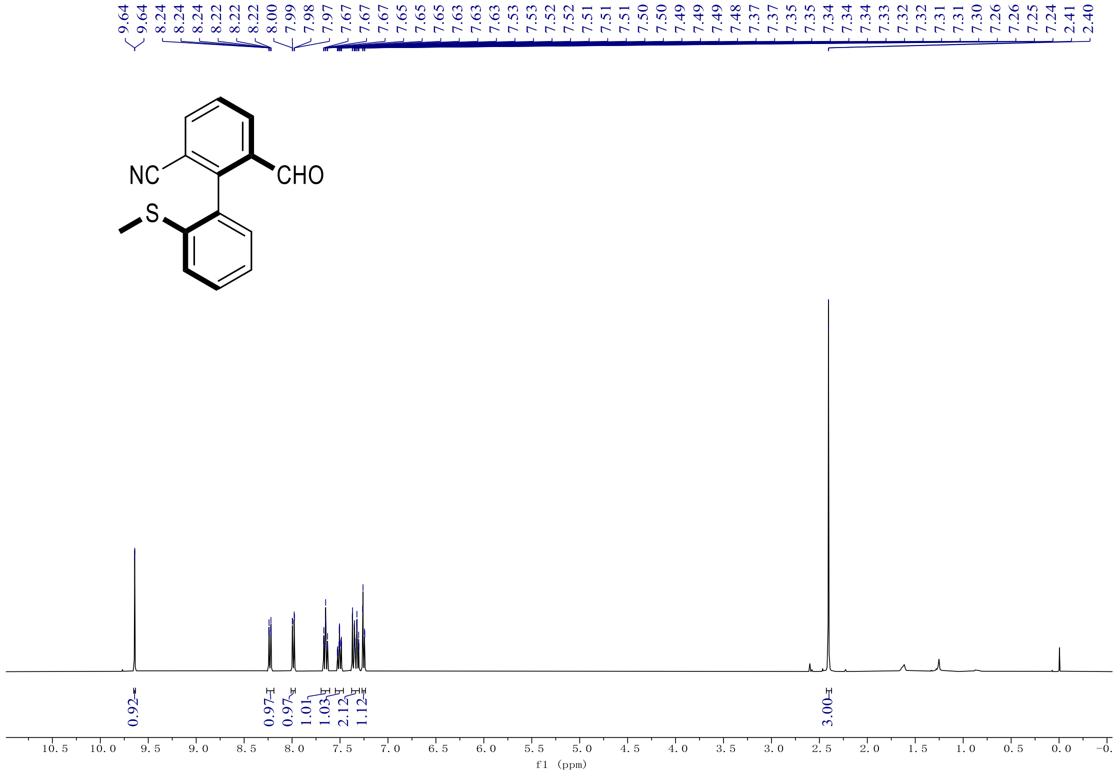

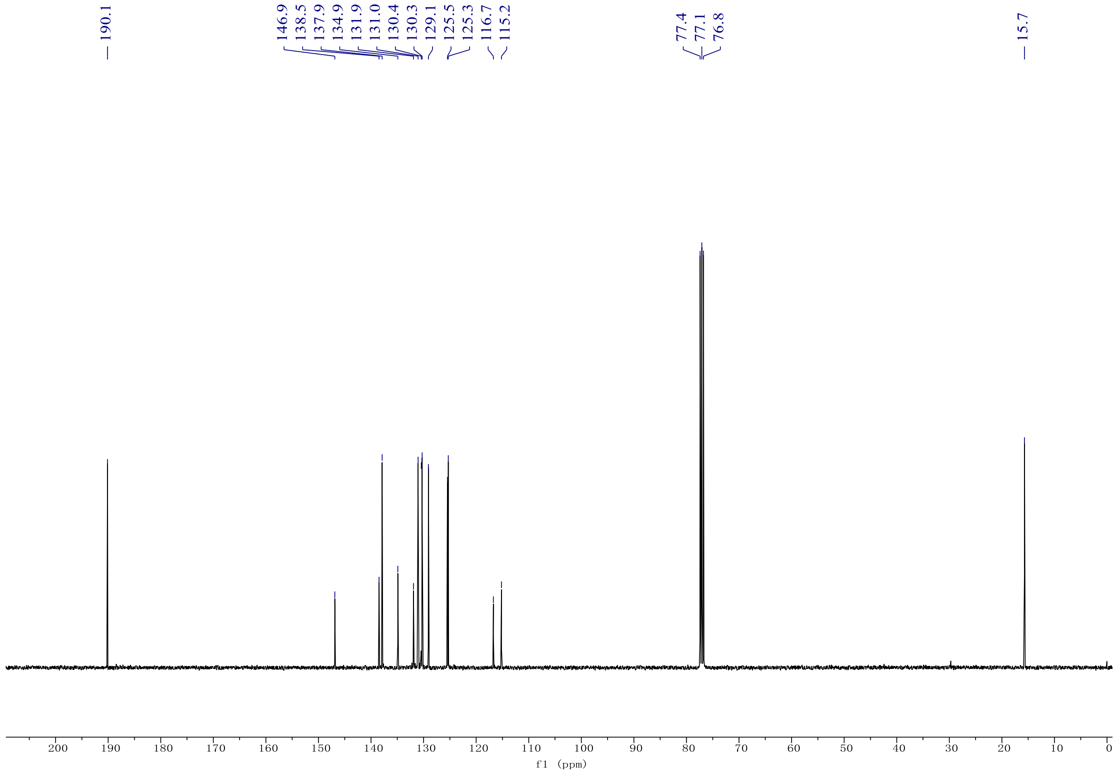

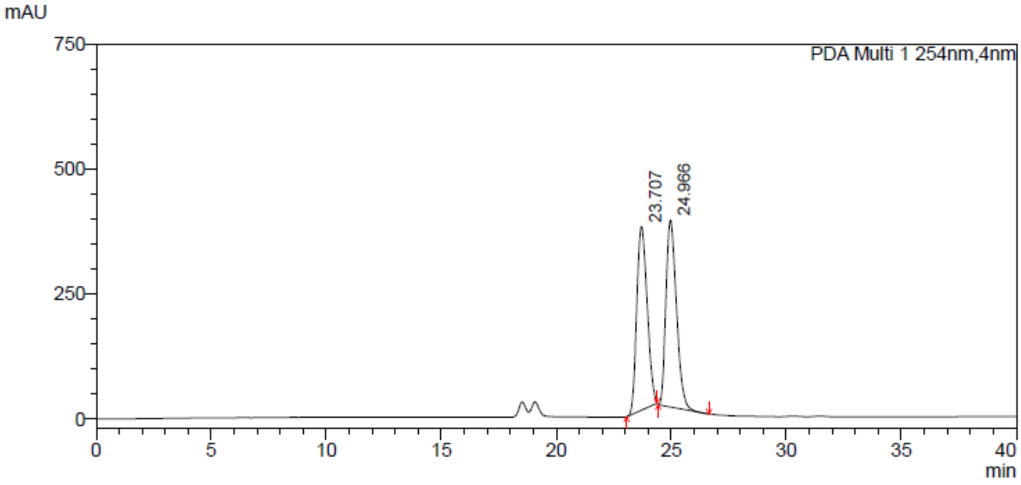

PDA Ch1 254nm

Ret. Time	Height	Area	Area%
23.707	366903	11575644	49.337
24.966	373485	11886974	50.663
	740388	23462618	100.000

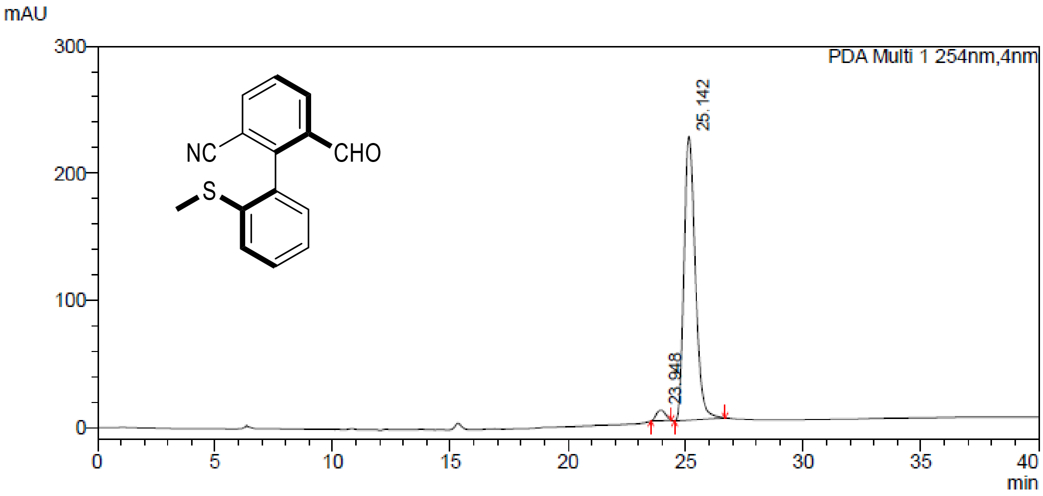

PDA Ch1 254nm

Ret. Time	Height	Area	Area%
23.948	8463	231291	3.015
25.142	223136	7441103	96.985
	231599	7672394	100.000

(*S*)-6-formyl-2'-vinyl-[1,1'-biphenyl]-2-carbonitrile (3n)

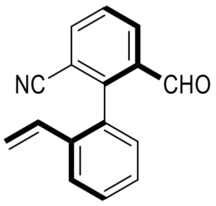

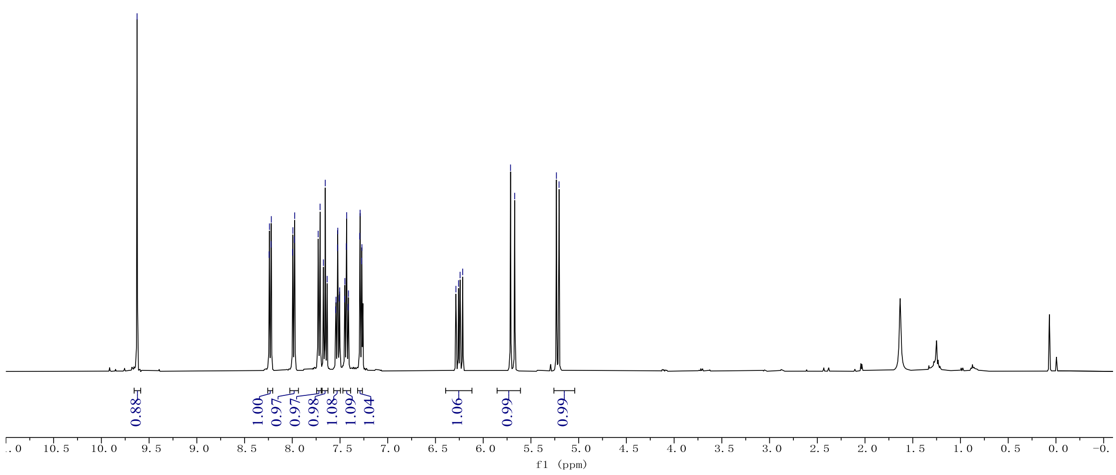

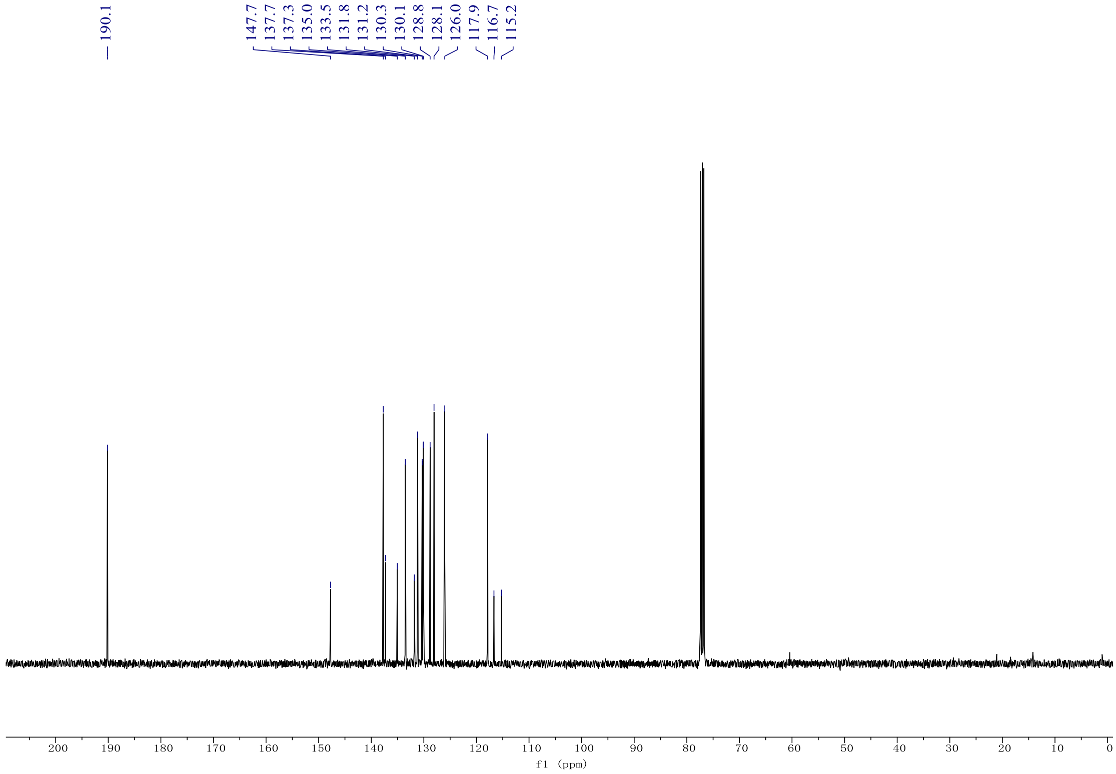

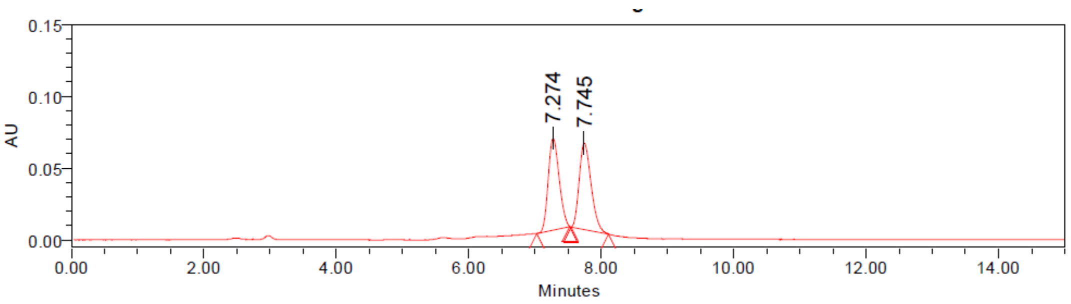

Peak Results

	RT	Height	Area (礦*sec)	% Area
1	7.274	63959	730824	49.46
2	7.745	60113	746840	50.54

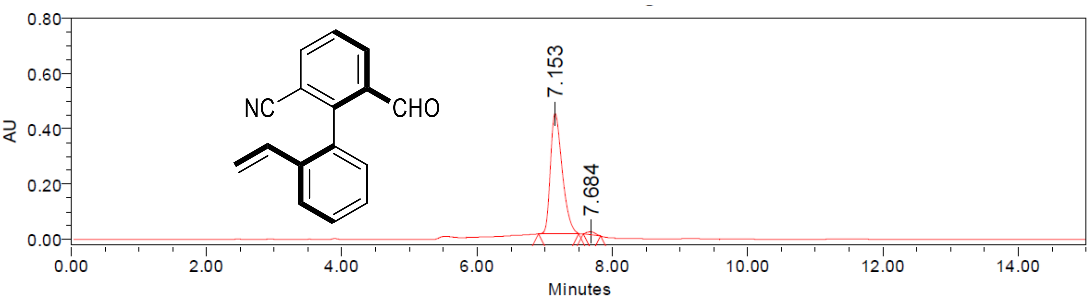

Peak Results

	RT	Height	Area (礦*sec)	% Area
1	7.153	435540	5247015	98.22
2	7.684	10931	95140	1.78

(*S*)-2'-chloro-6-formyl-[1,1'-biphenyl]-2-carbonitrile (3o)

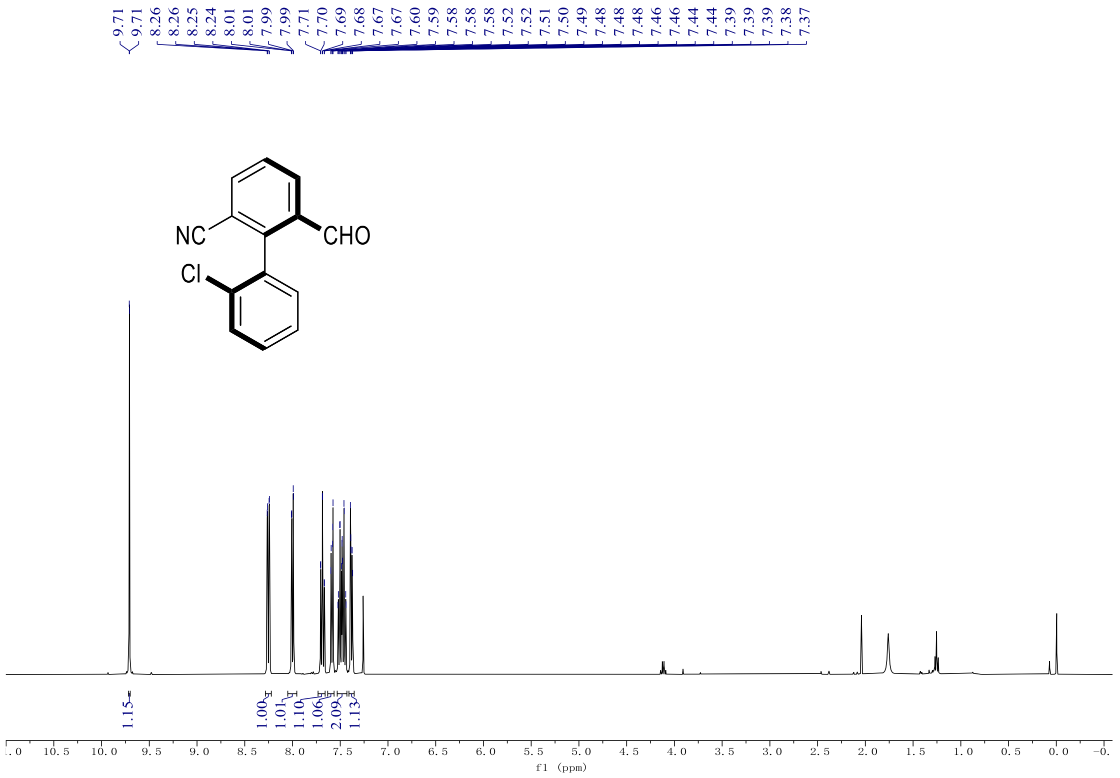

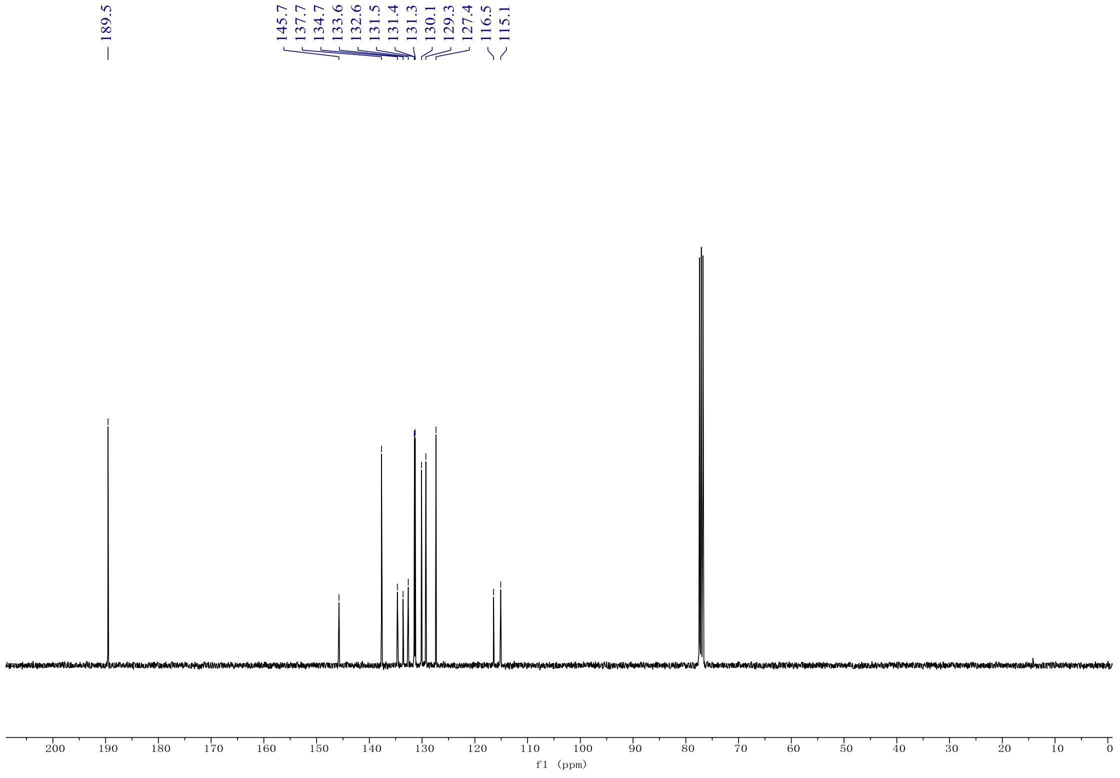

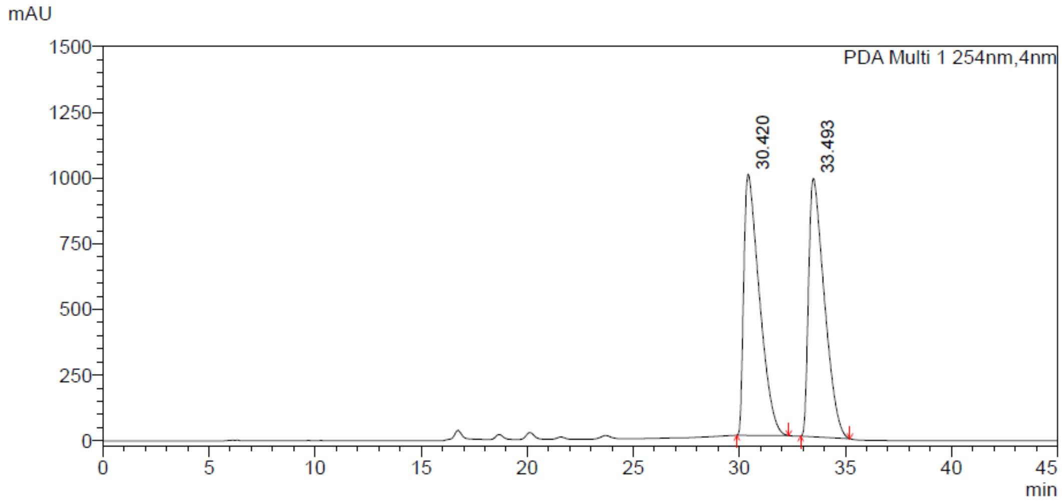

PDA Ch1 254nm

Ret. Time	Height	Area	Area%
30.420	993260	50946352	50.077
33.493	983866	50789391	49.923
	1977125	101735743	100.000

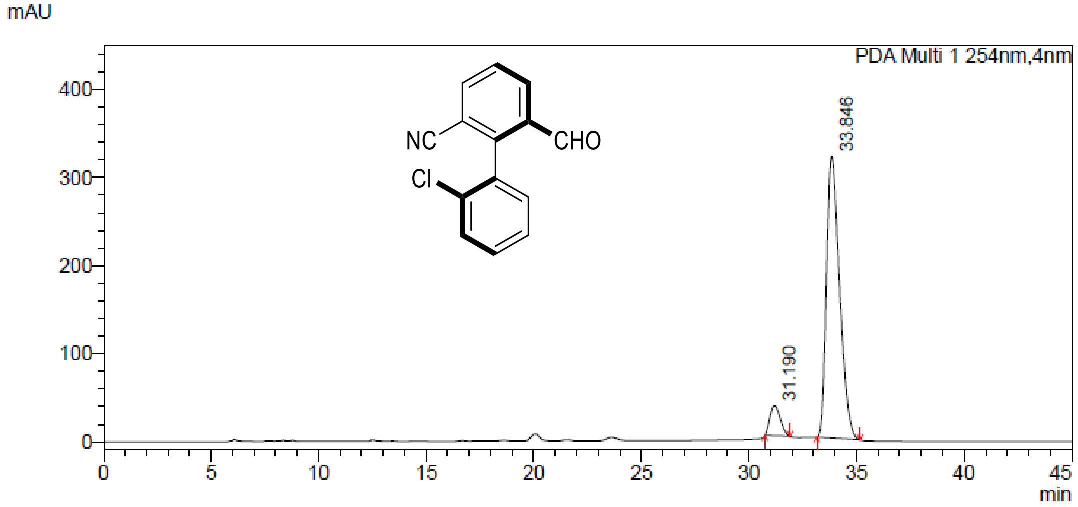

PDA Ch1 254nm

Ret. Time	Height	Area	Area%
31.190	33964	1143438	7.765
33.846	319653	13582961	92.235
	353616	14726399	100.000

(*S*)-2'-bromo-6-formyl-[1,1'-biphenyl]-2-carbonitrile (3p)

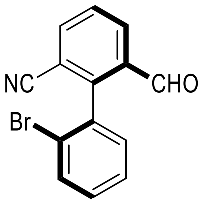

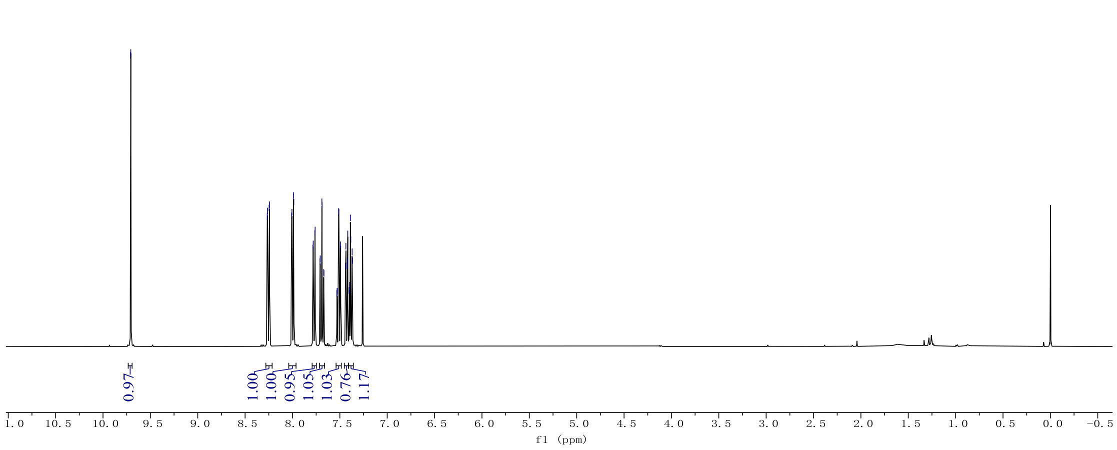

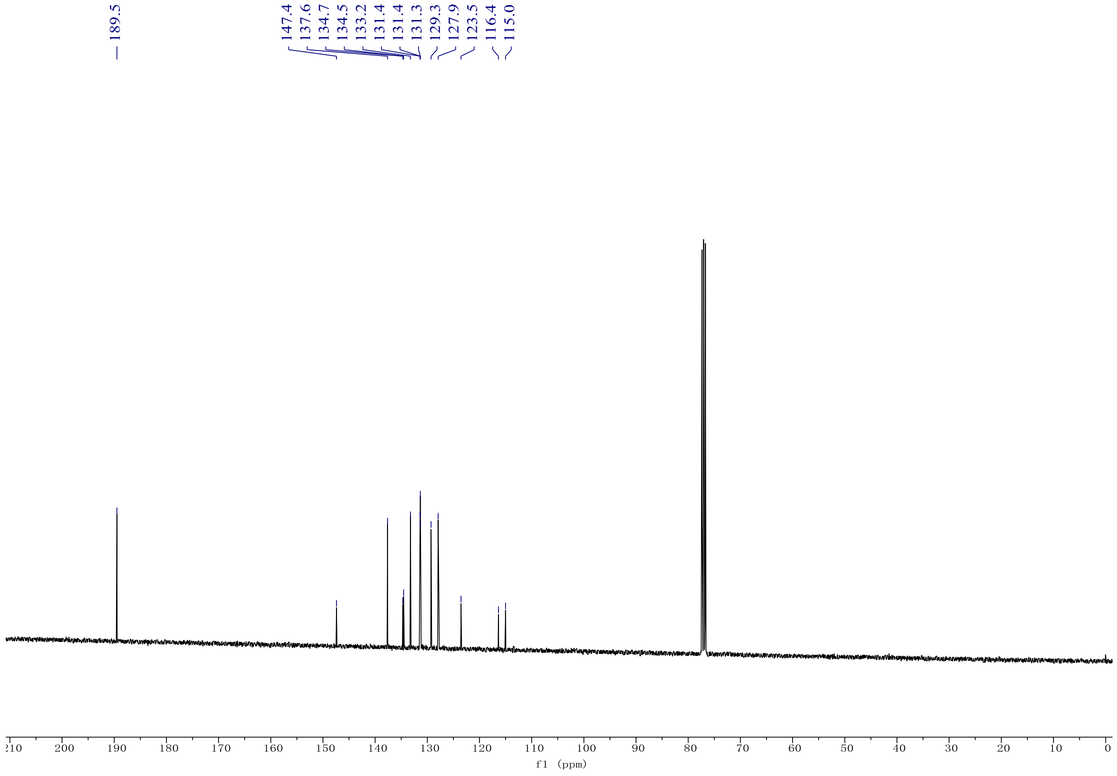

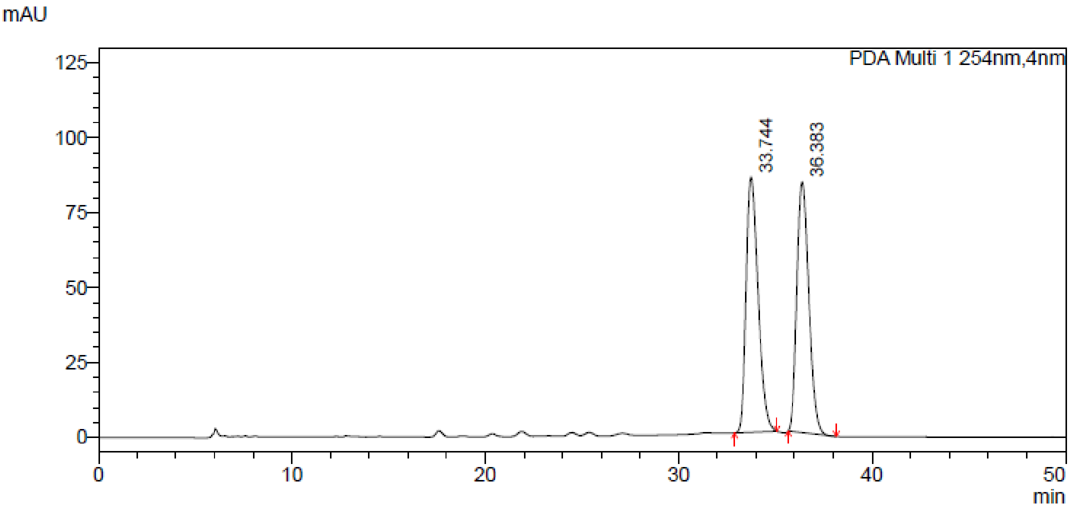

PDA Ch1 254nm

Ret. Time	Height	Area	Area%
33.744	84979	3642396	50.431
36.383	83570	3580087	49.569
	168549	7222483	100.000

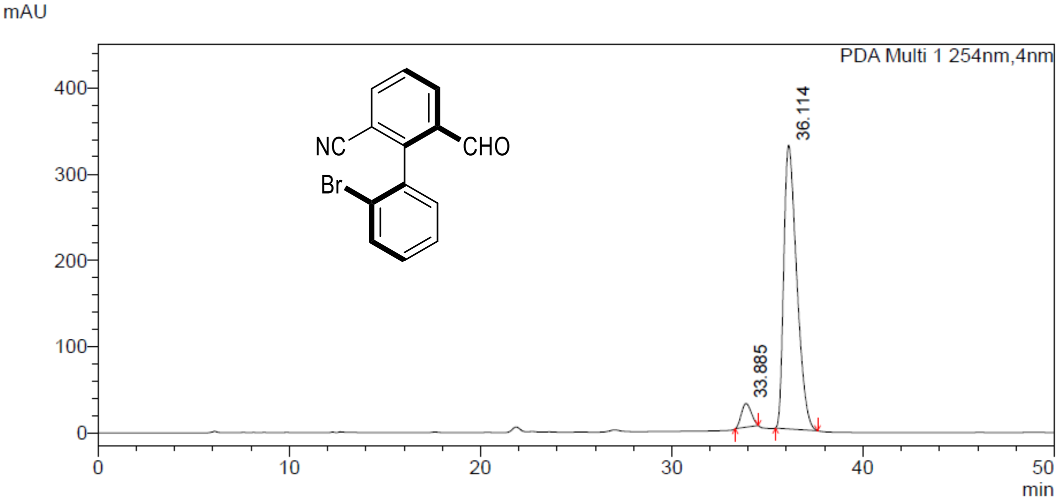

PDA Ch1 254nm

Ret. Time	Height	Area	Area%
33.885	27331	989519	5.996
36.114	329066	15513942	94.004
	356397	16503462	100.000

(*S*)-6-formyl-[1,1':2',1''-terphenyl]-2-carbonitrile (3q)

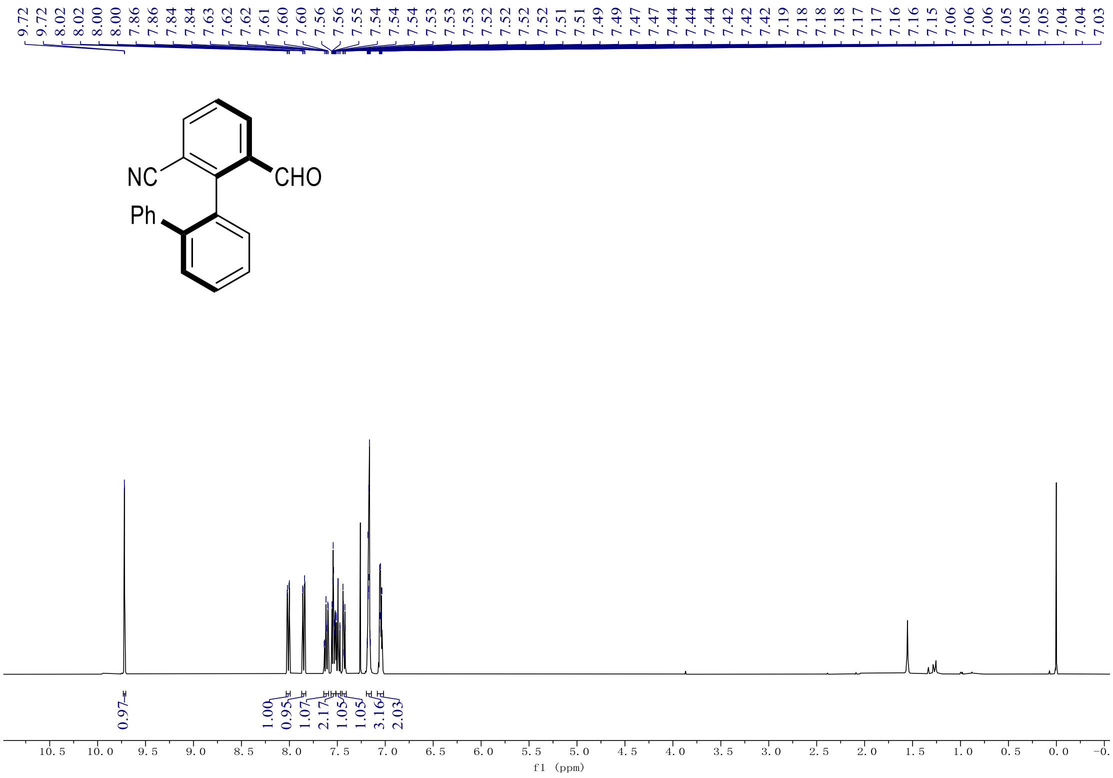

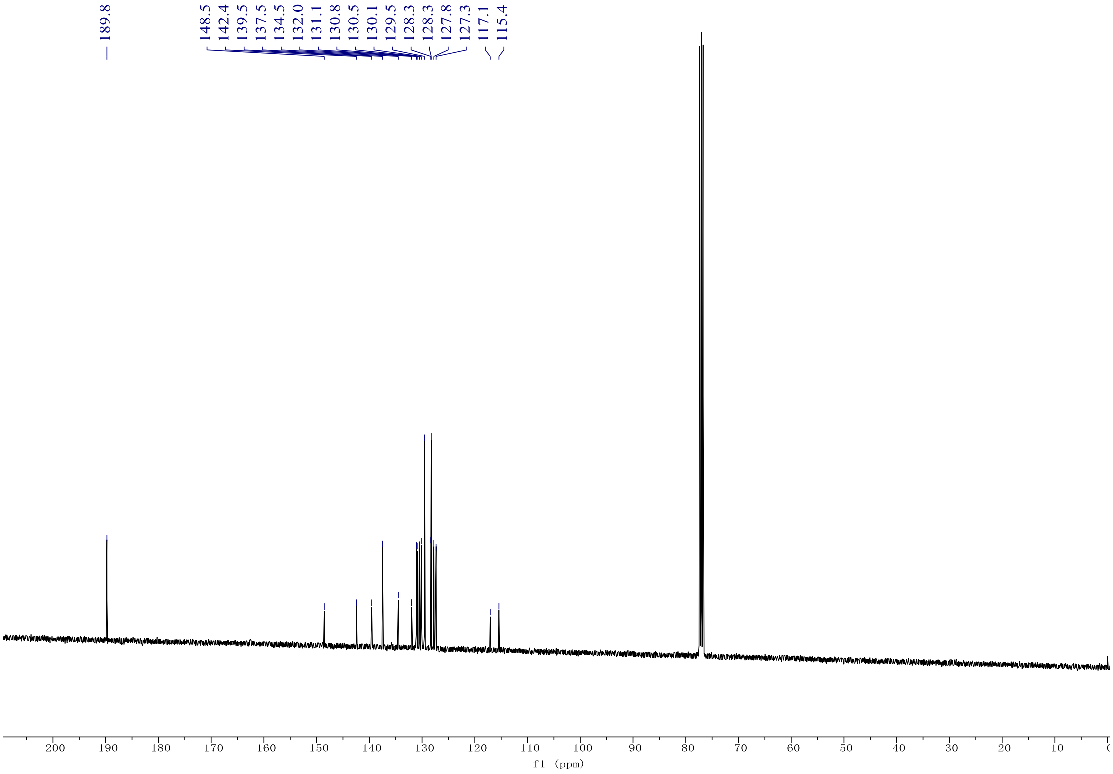

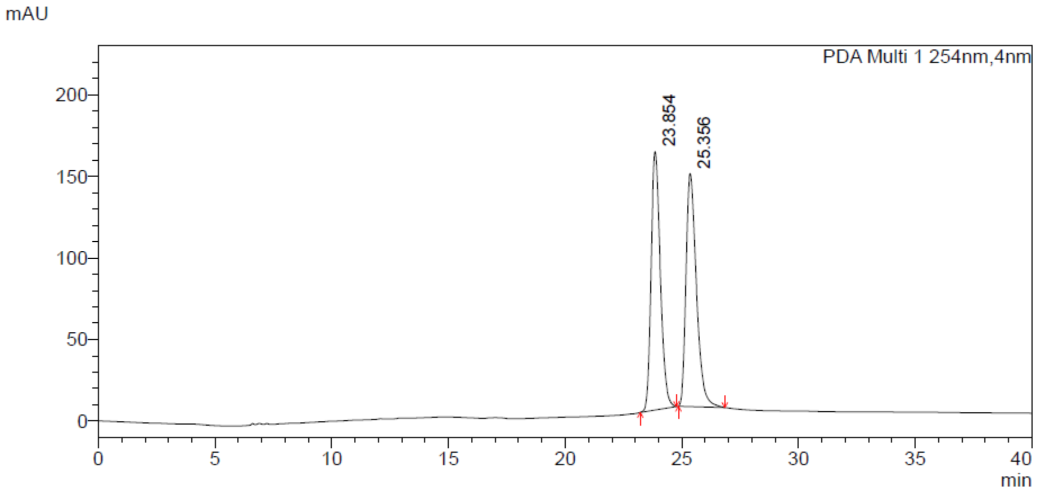

PDA Ch1 254nm

Ret. Time	Height	Area	Area%
23.854	158430	4343976	49.511
25.356	142859	4429712	50.489
	301289	8773688	100.000

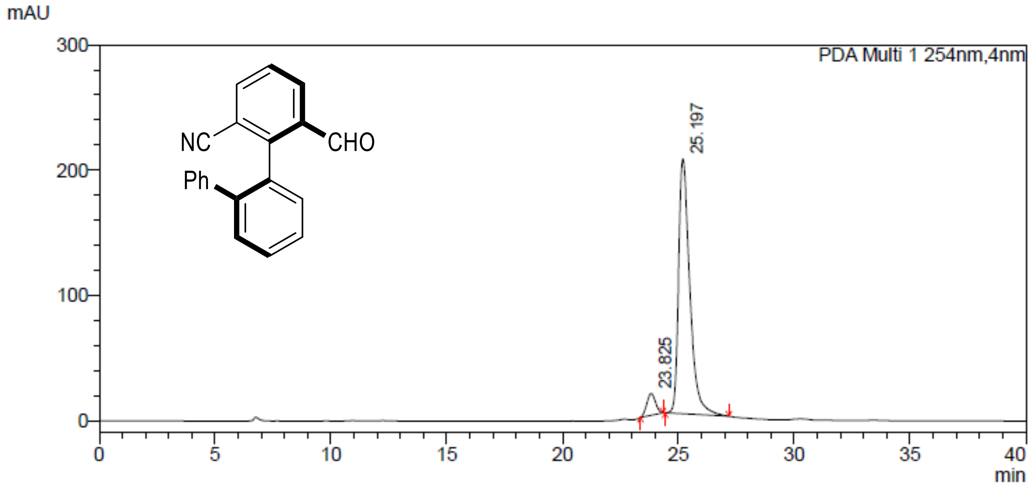

PDA Ch1 254nm

Ret. Time	Height	Area	Area%
23.825	17402	456460	6.238
25.197	203514	6860626	93.762
	220916	7317085	100.000

(*S*)-3-formyl-2-(naphthalen-1-yl)benzonitrile (3r)

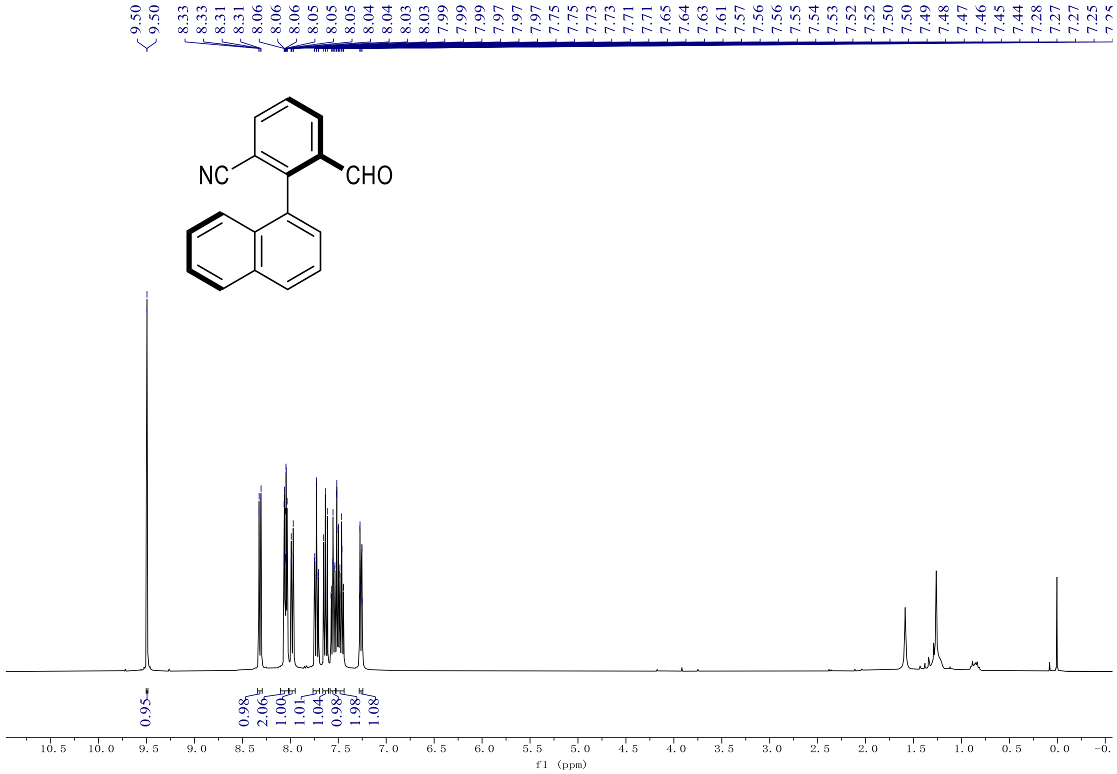

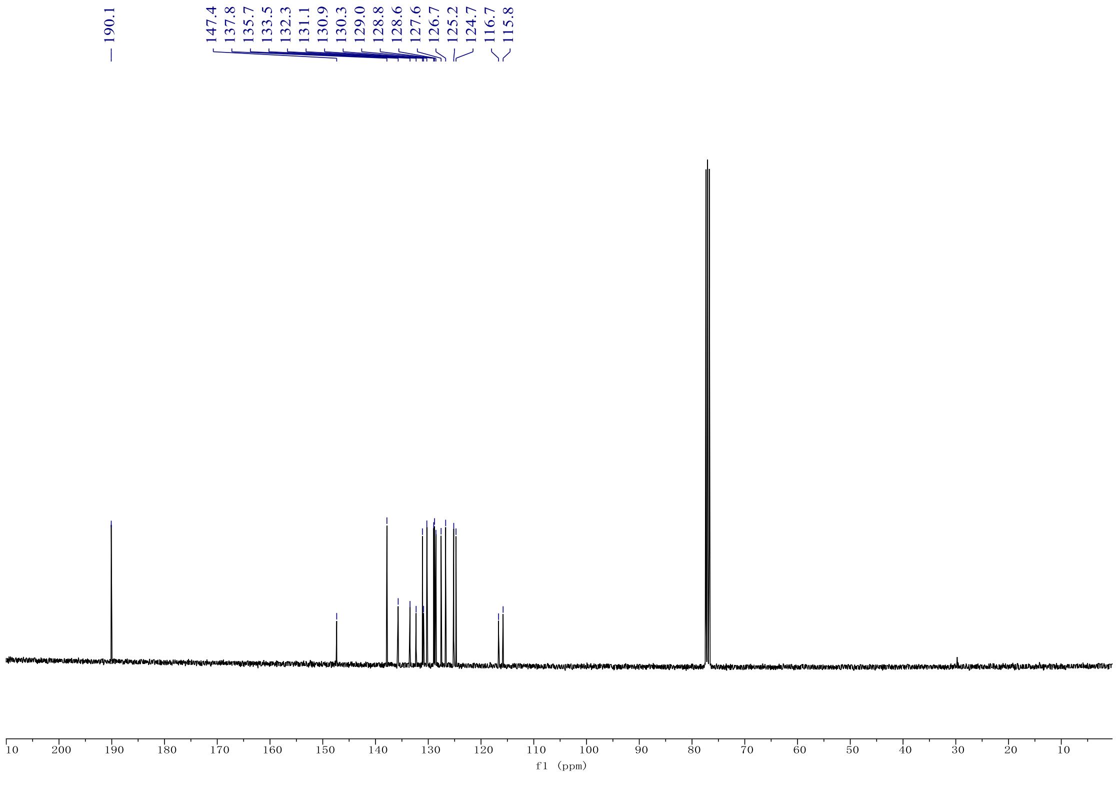

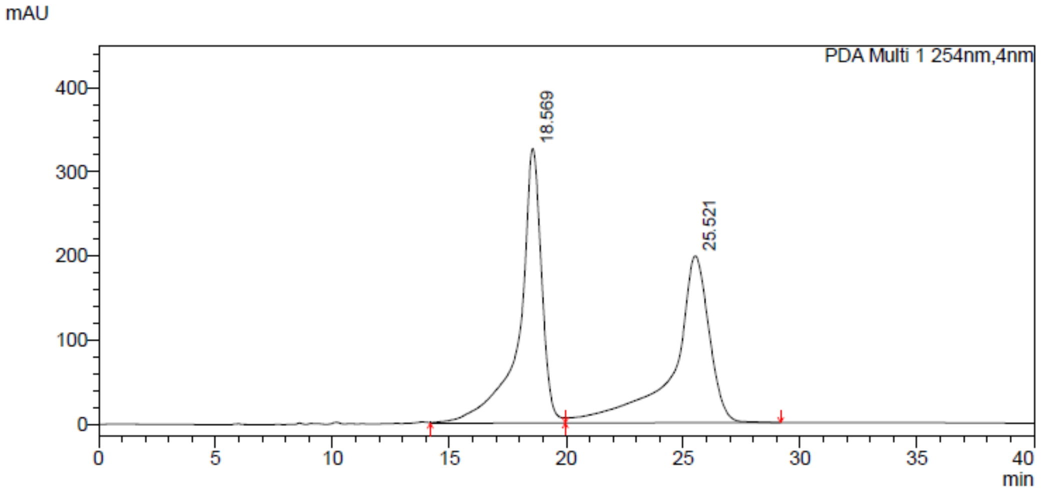

PDA Ch1 254nm

Ret. Time	Height	Area	Area%
18.569	326129	21794342	50.448
25.521	198021	21407491	49.552
	524150	43201833	100.000

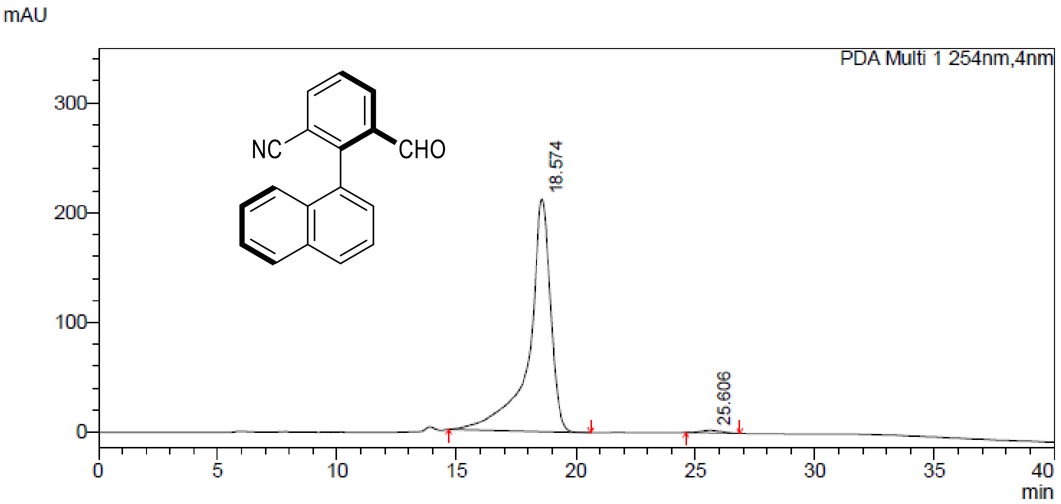

PDA Ch1 254nm

Ret. Time	Height	Area	Area%
18.574	211662	13253949	98.914
25.606	2290	145471	1.086
	213951	13399420	100.000

(*S*)-3-formyl-2-(phenanthren-9-yl)benzonitrile (3s)

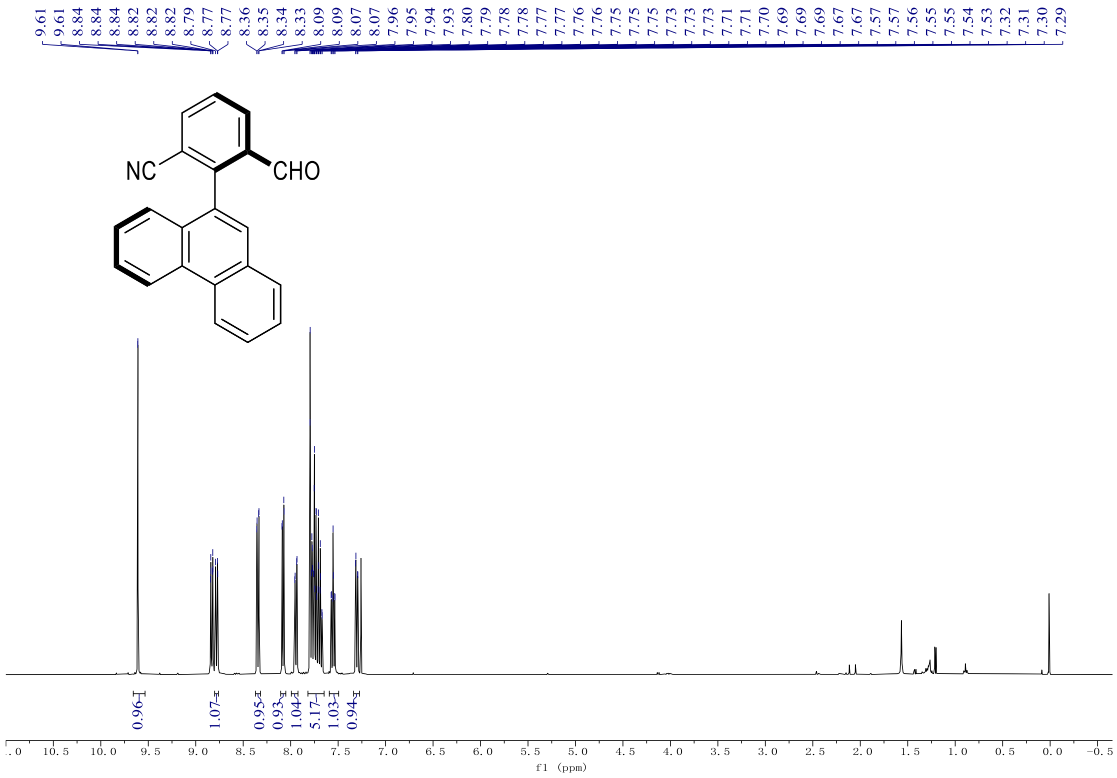

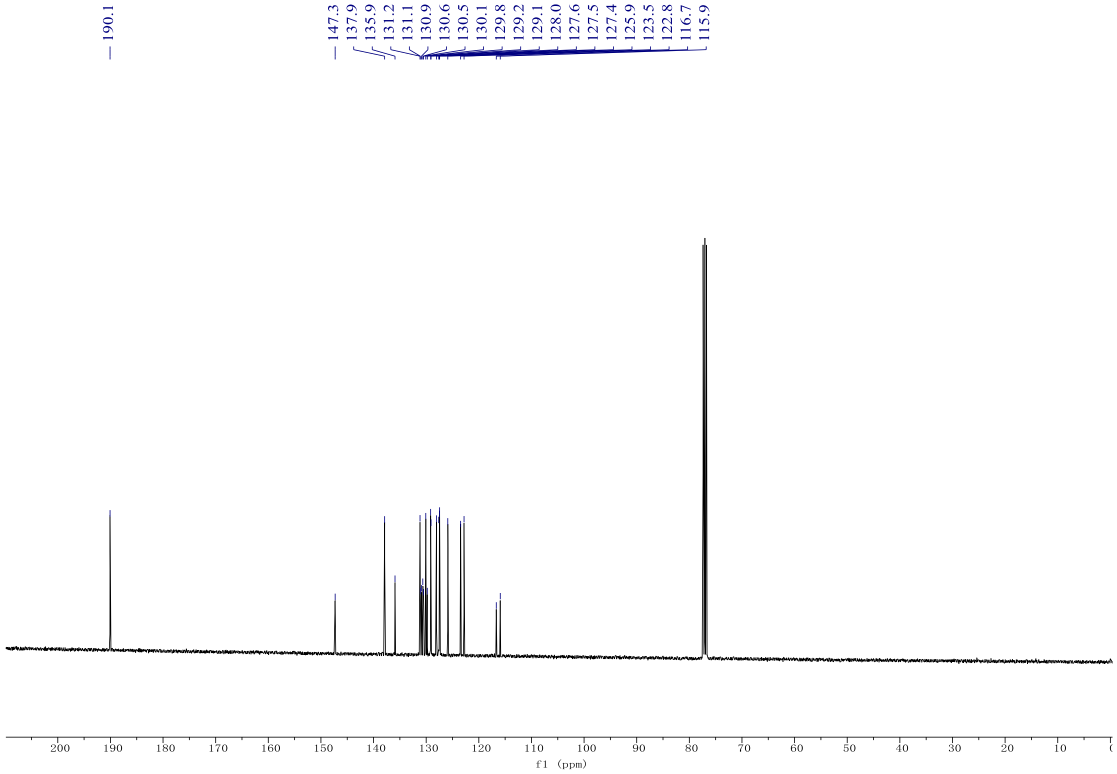

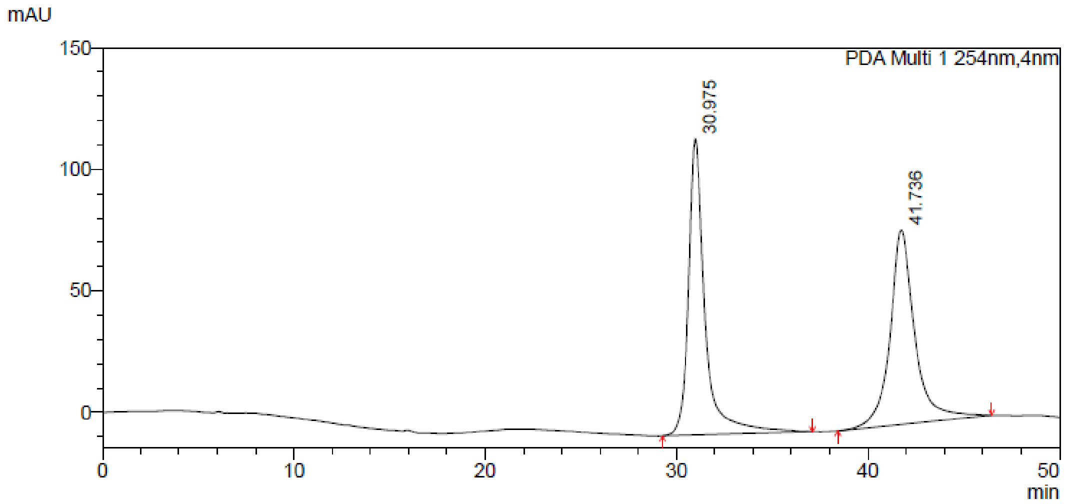

PDA Ch1 254nm

Ret. Time	Height	Area	Area%
30.975	121838	7341857	50.433
41.736	79978	7215736	49.567
	201816	14557593	100.000

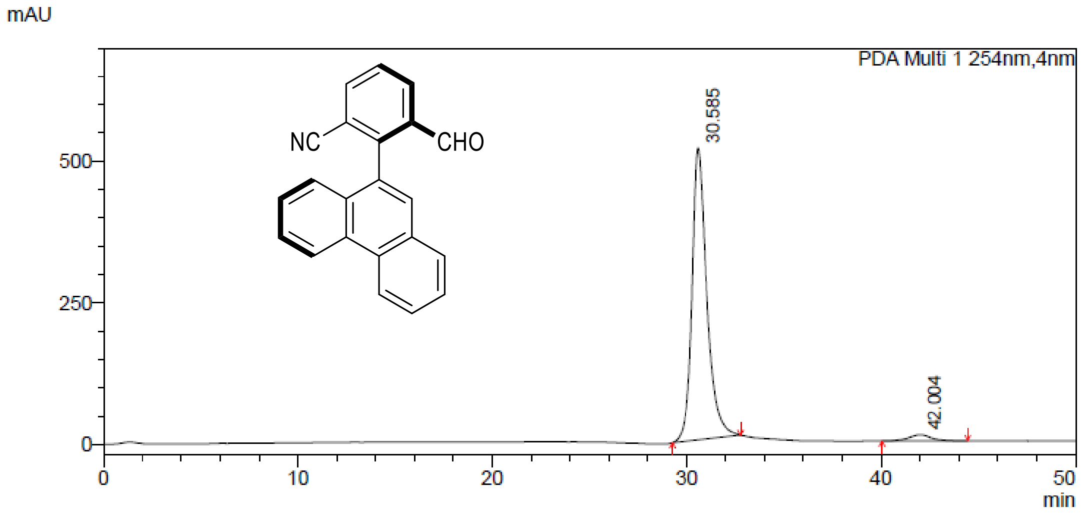

PDA Ch1 254nm

Ret. Time	Height	Area	Area%
30.585	515371	27309620	96.916
42.004	10711	869127	3.084
	526081	28178748	100.000

(*S*)-3-formyl-2-(pyren-1-yl)benzonitrile (3t)

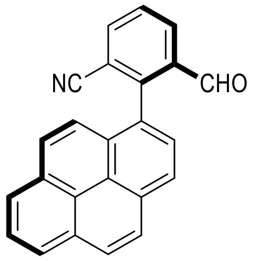

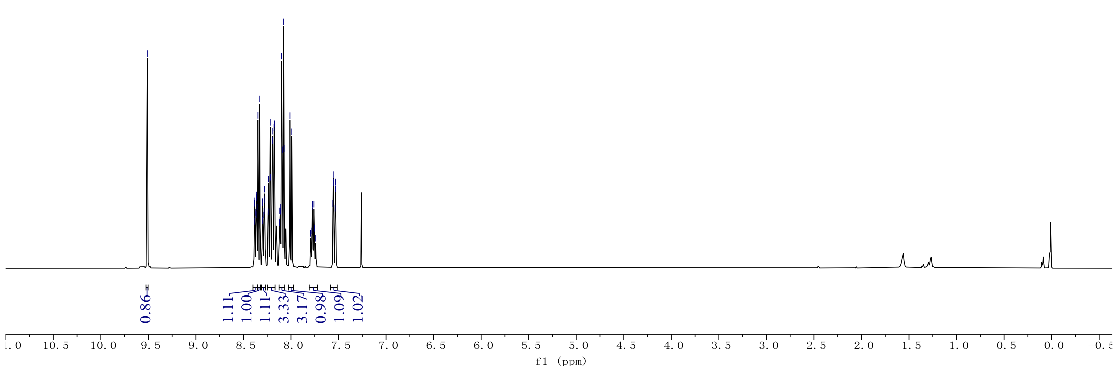

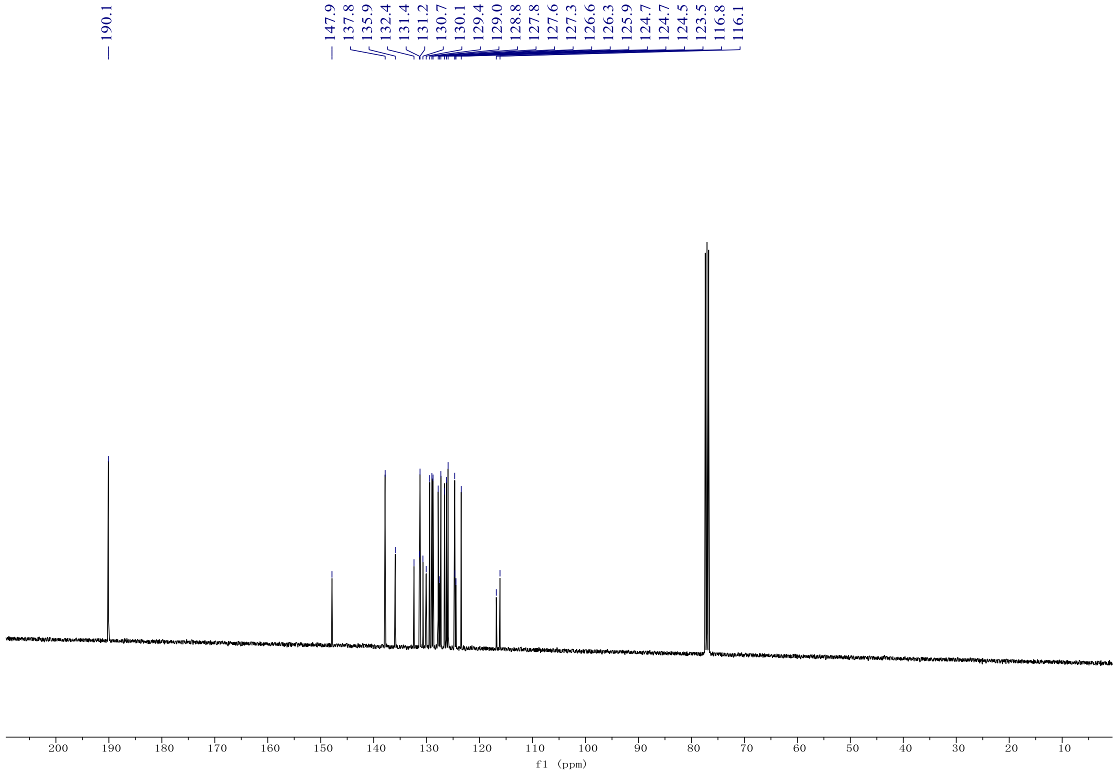

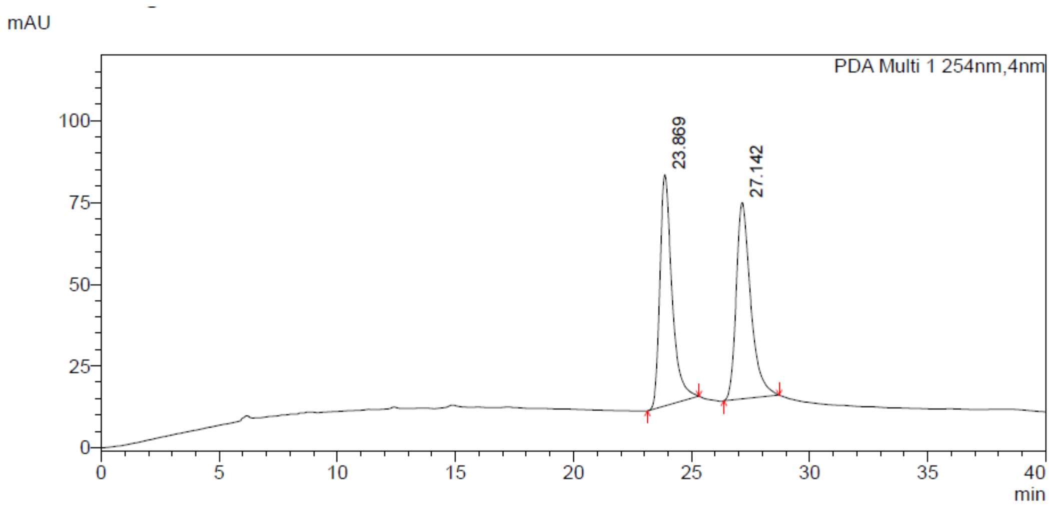

PDA Ch1 254nm

Ret. Time	Height	Area	Area%
23.869	70665	2535279	49.686
27.142	60019	2567347	50.314
	130683	5102626	100.000

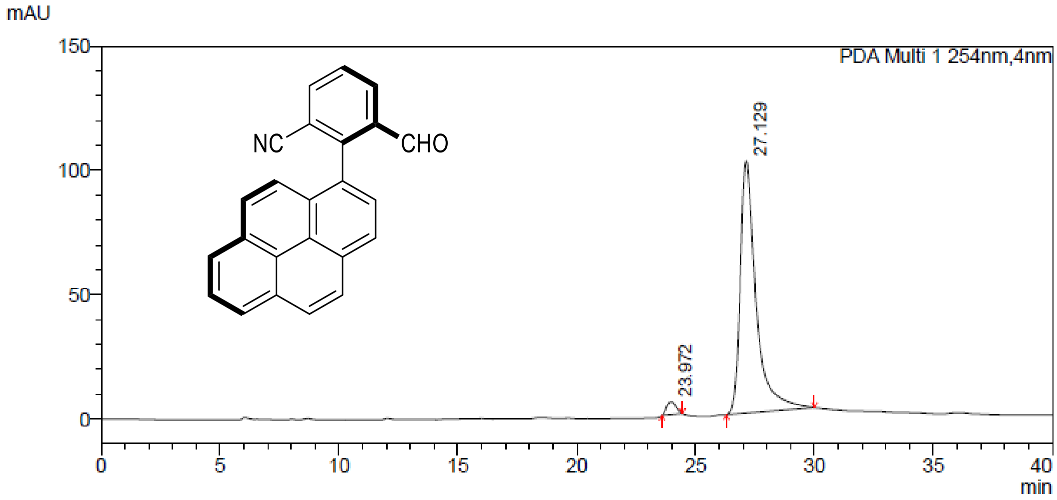

PDA Ch1 254nm

Ret. Time	Height	Area	Area%
23.972	5066	137796	2.850
27.129	101326	4697794	97.150
	106391	4835589	100.000

(*S*)-3-formyl-2-(2-methoxynaphthalen-1-yl)benzonitrile (3u)

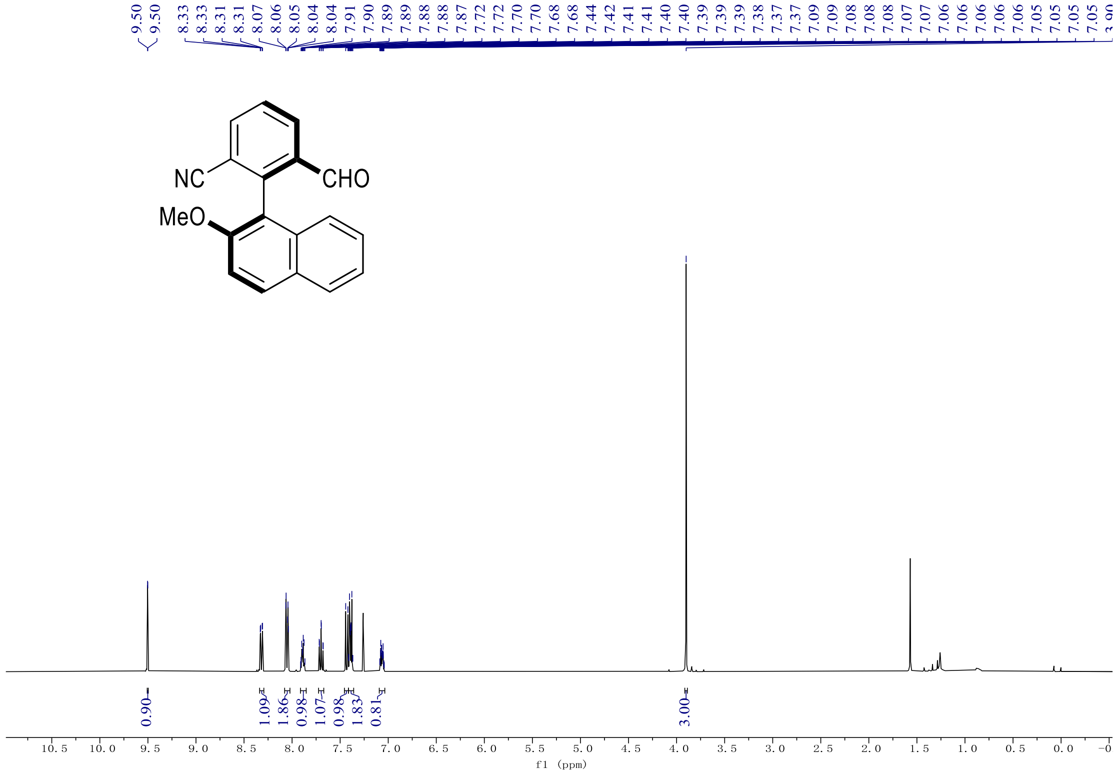

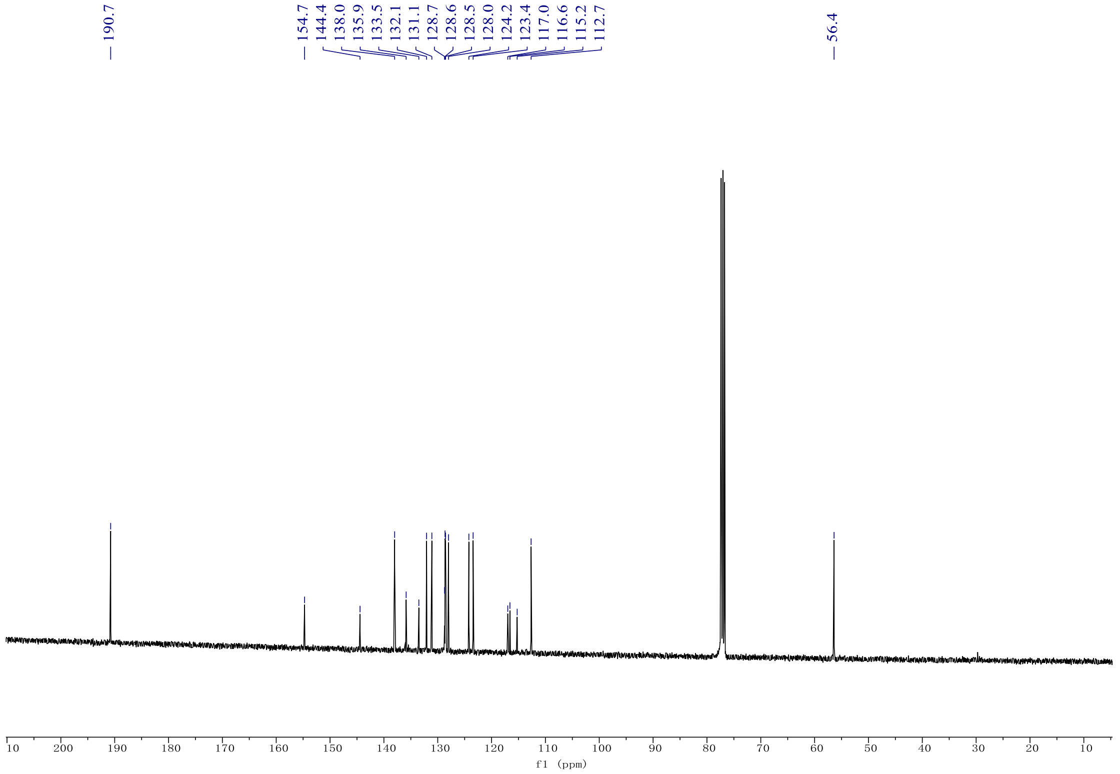

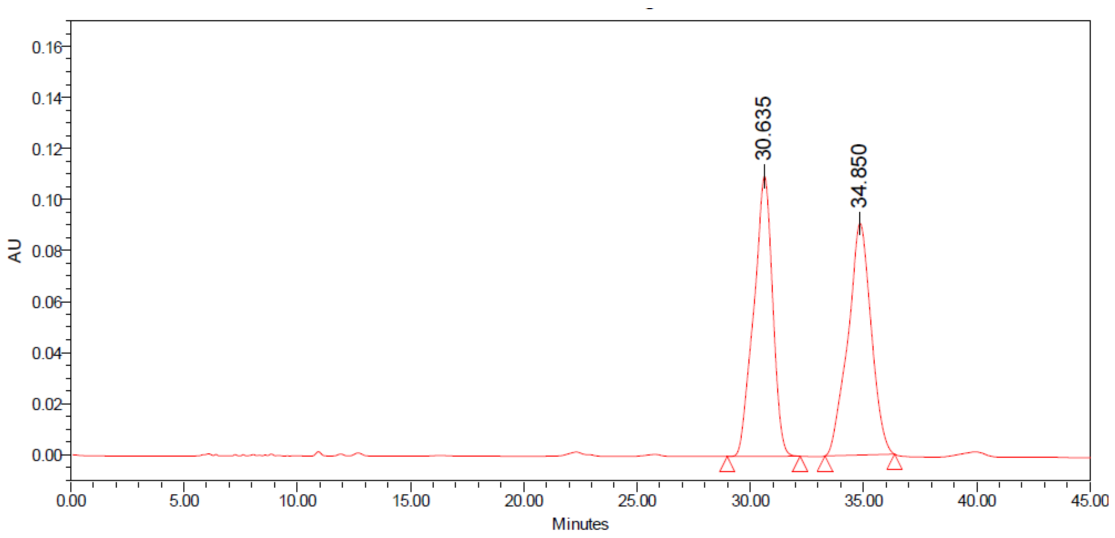

Peak Results

	RT	Height	Area	% Area
1	30.635	109739	6359716	49.47
2	34.850	90724	6497252	50.53

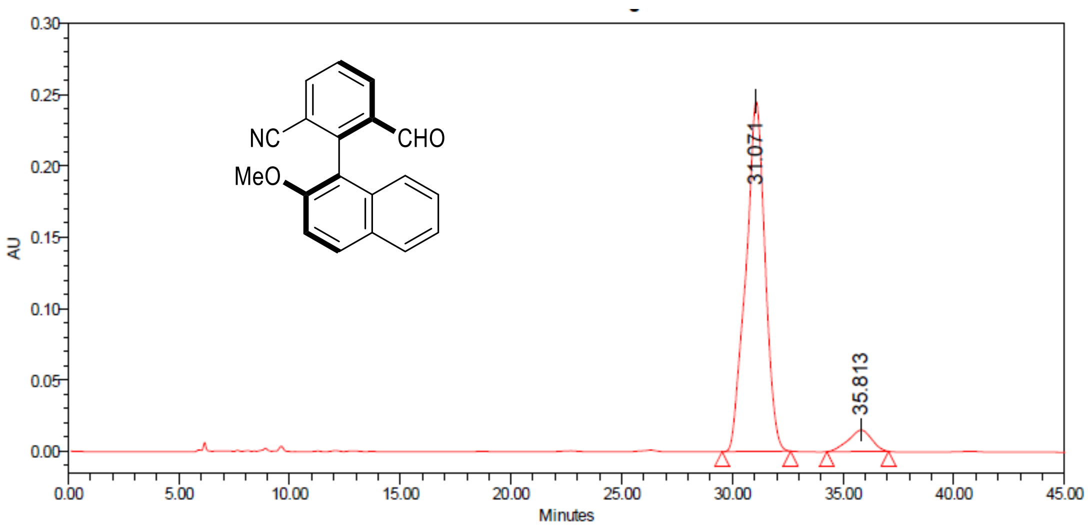

Peak Results

	RT	Height	Area	% Area
1	31.071	245415	14882573	93.12
2	35.813	14934	1099016	6.88

(*S*)-6-formyl-4-methoxy-2'-methyl-[1,1'-biphenyl]-2-carbonitrile (3v)

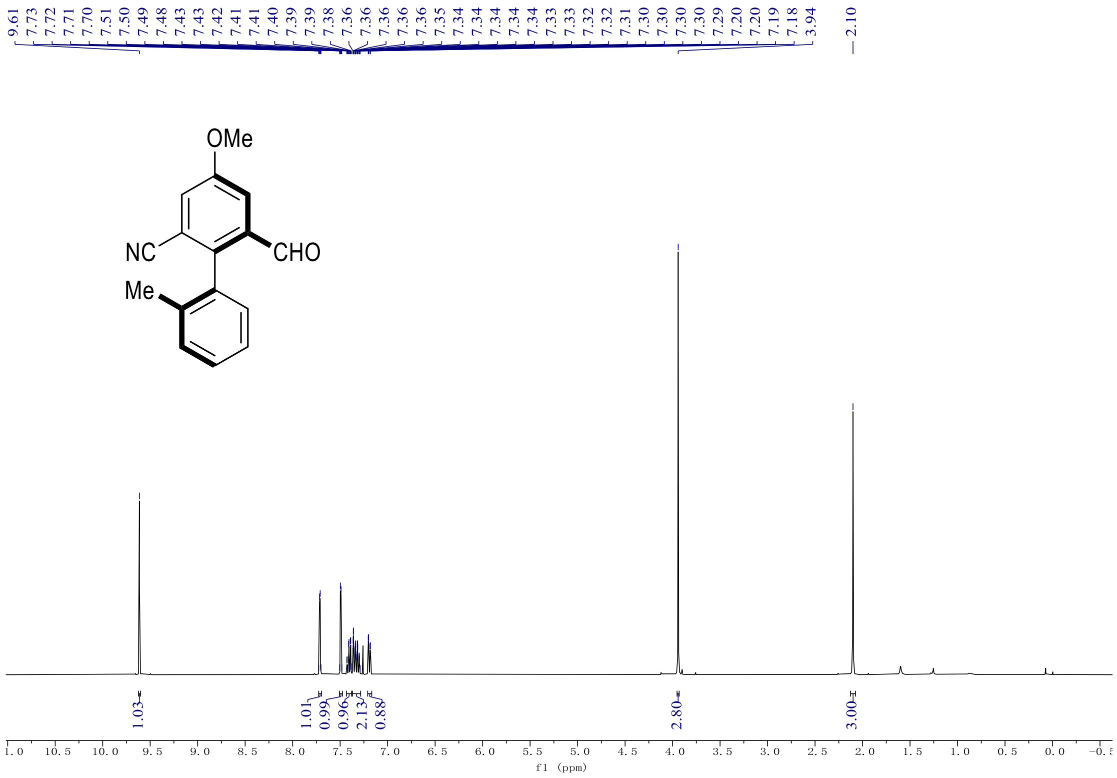

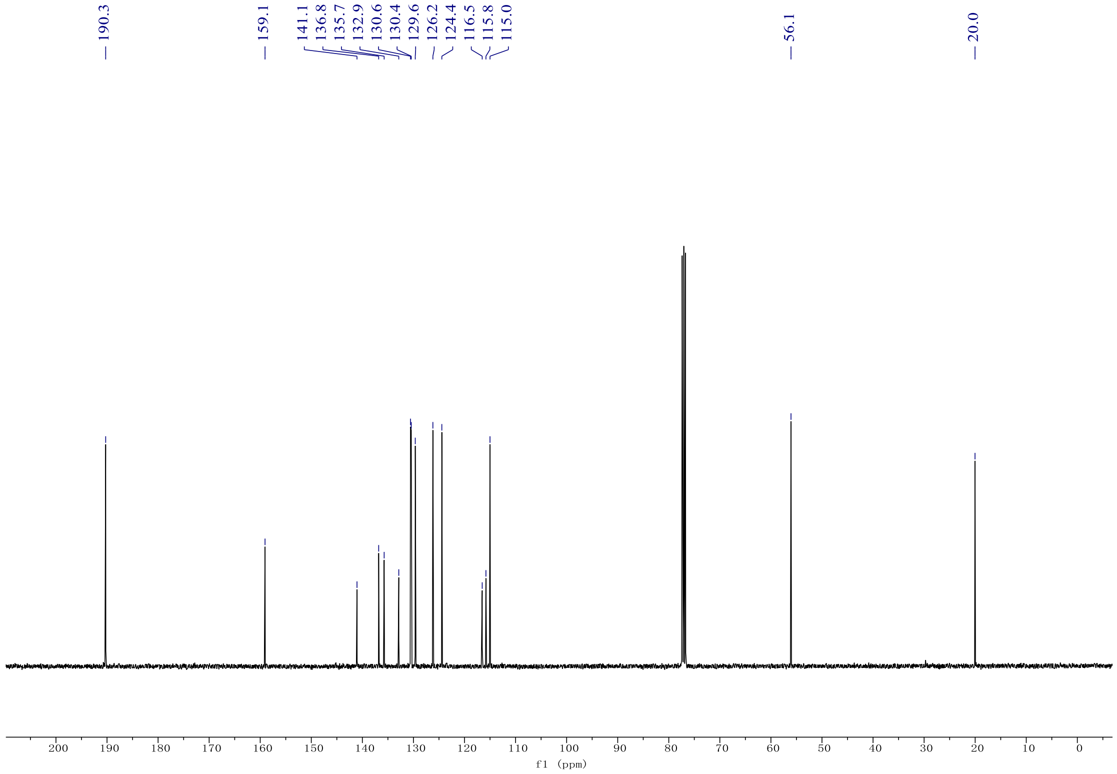

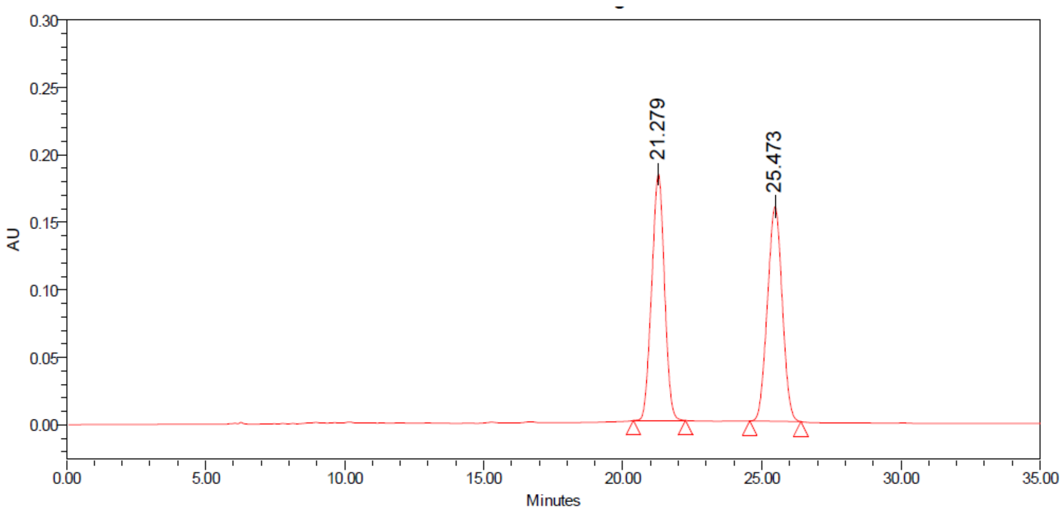

Peak Results

	RT	Height	Area	% Area
1	21.279	182557	5816917	49.75
2	25.473	159017	5875653	50.25

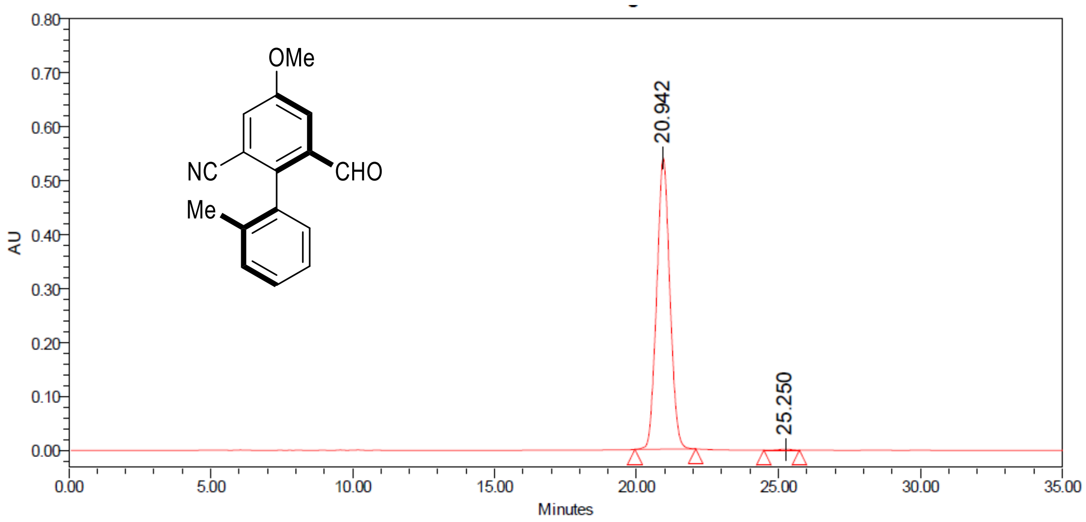

Peak Results

	RT	Height	Area	% Area
1	20.942	540539	17401005	99.71
2	25.250	1514	50364	0.29

(*S*)-4-fluoro-6-formyl-2'-methyl-[1,1'-biphenyl]-2-carbonitrile (3w)

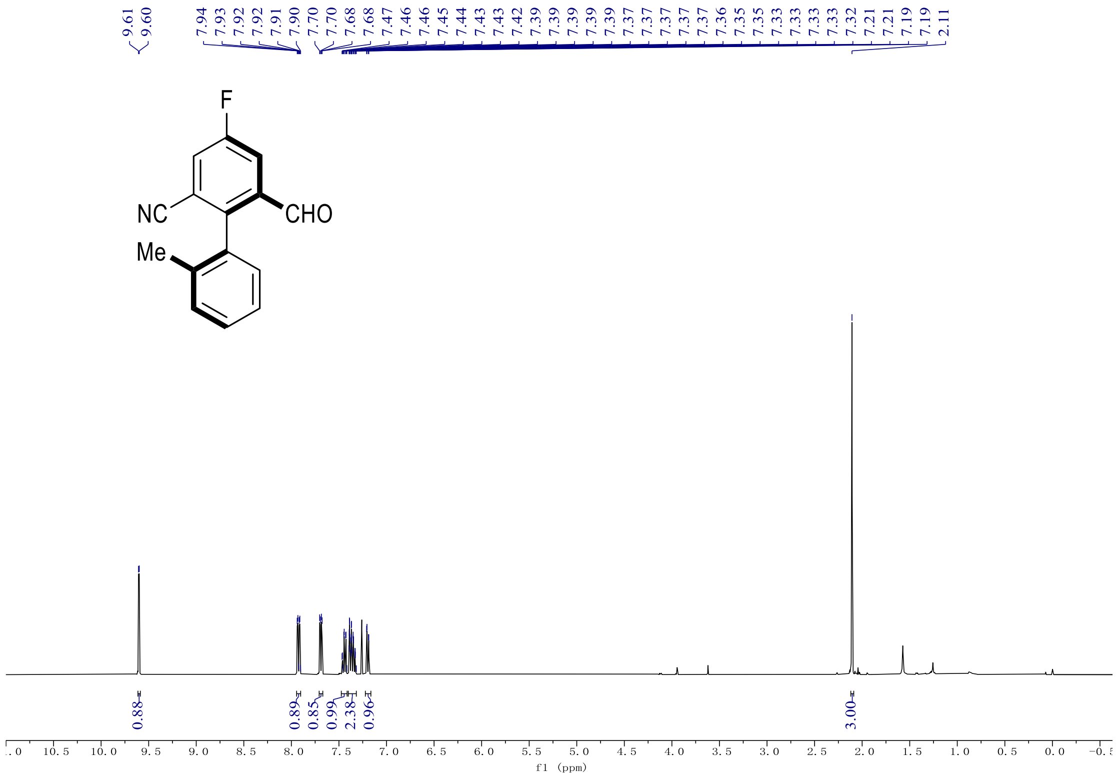

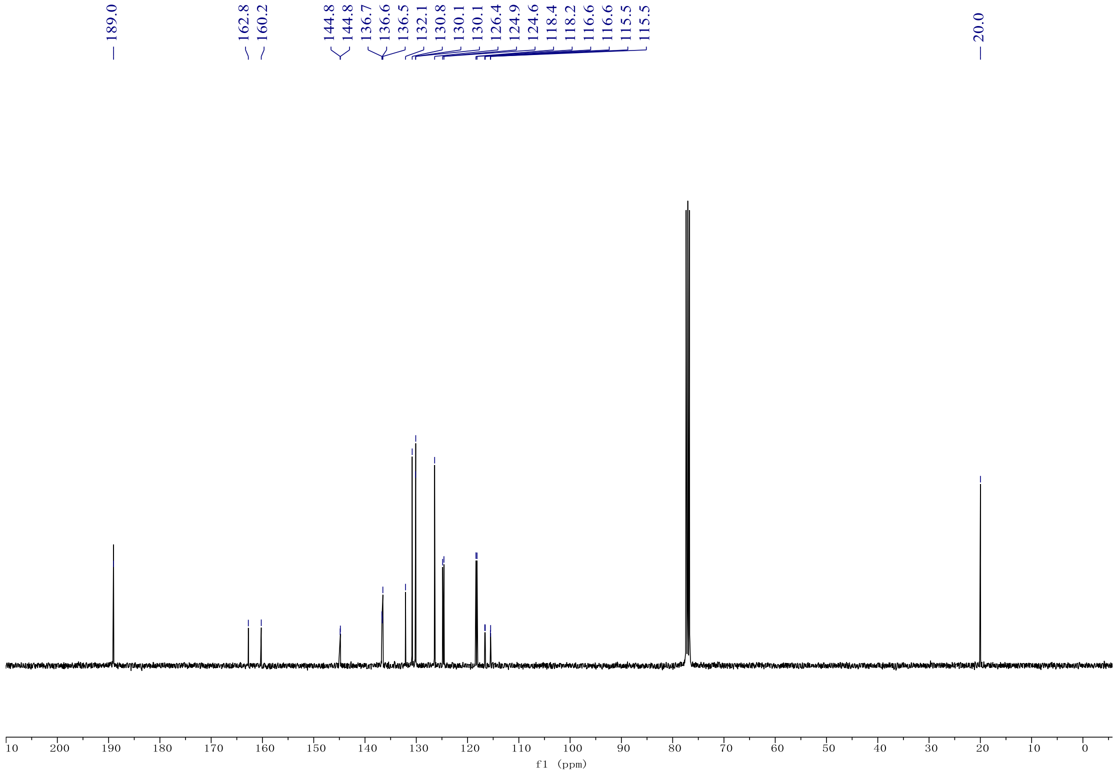

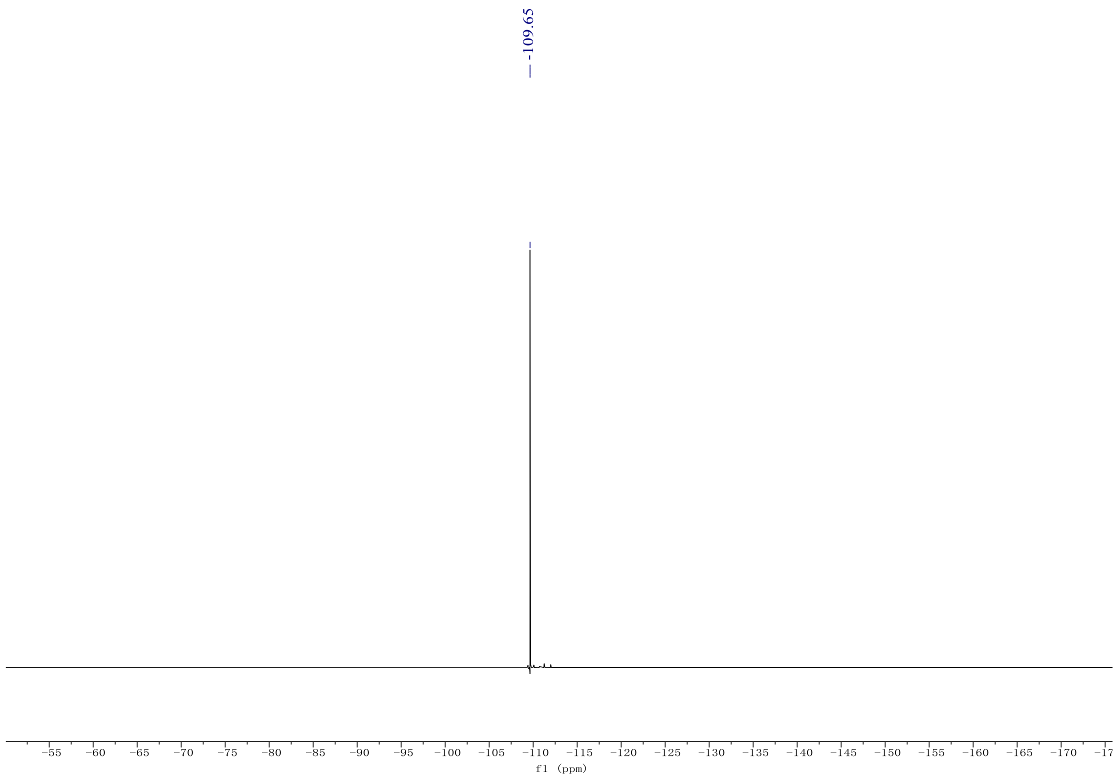
-109.65
f1 (ppm)

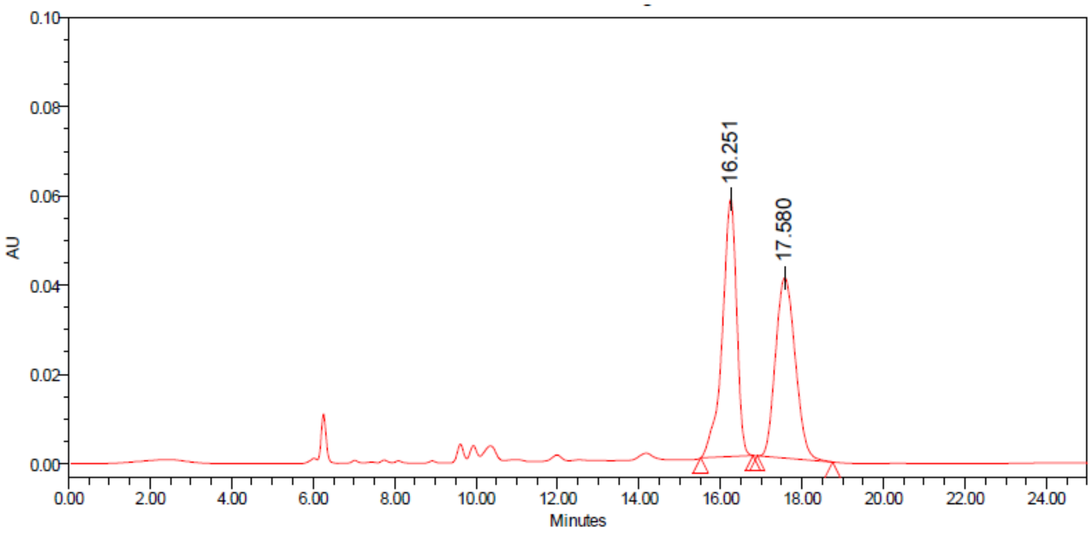

Peak Results

	RT	Height	Area	% Area
1	16.251	57677	1412973	50.86
2	17.580	40462	1365029	49.14

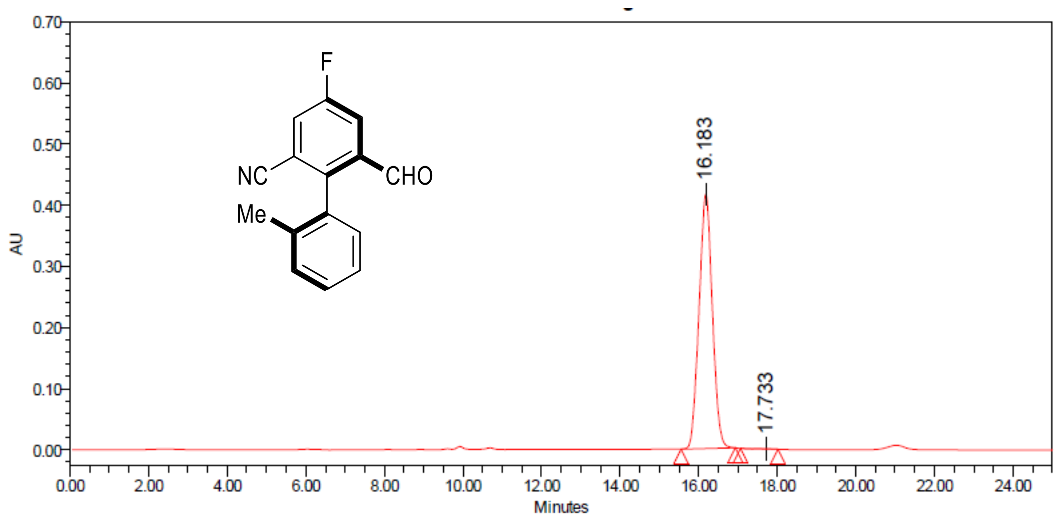

Peak Results

	RT	Height	Area	% Area
1	16.183	415878	9806033	99.81
2	17.733	609	18594	0.19

(*R*)-2'-methyl-6-vinyl-[1,1'-biphenyl]-2-carbonitrile (5)

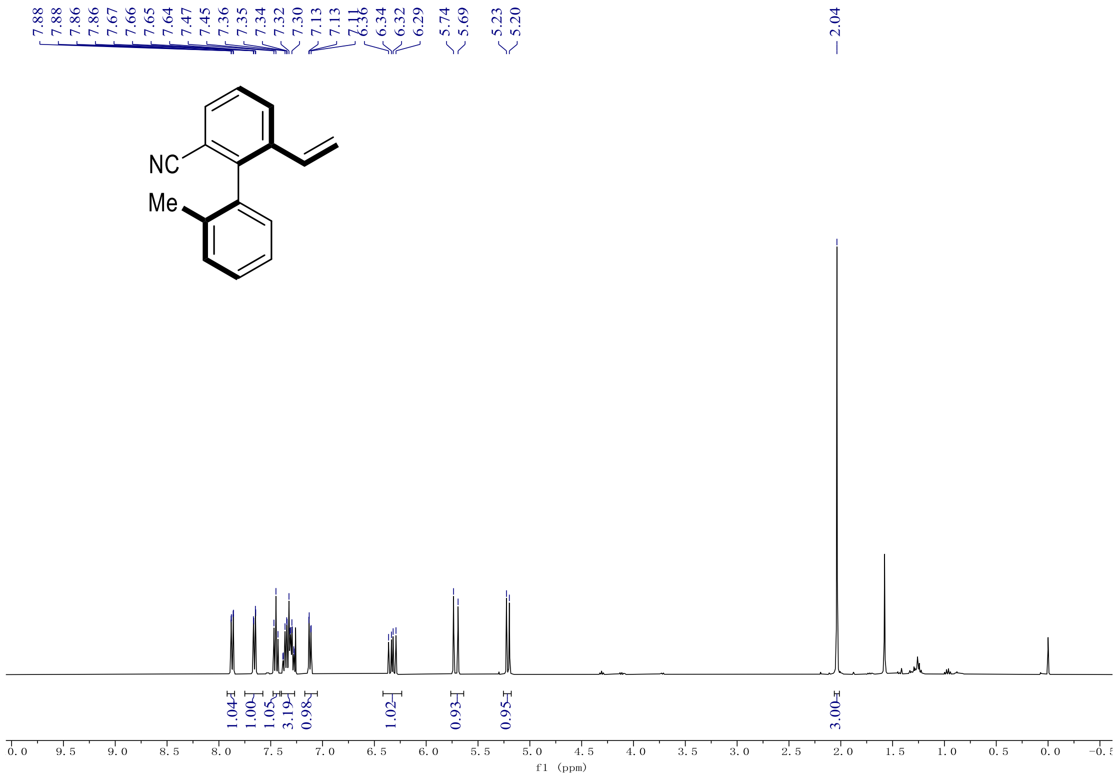

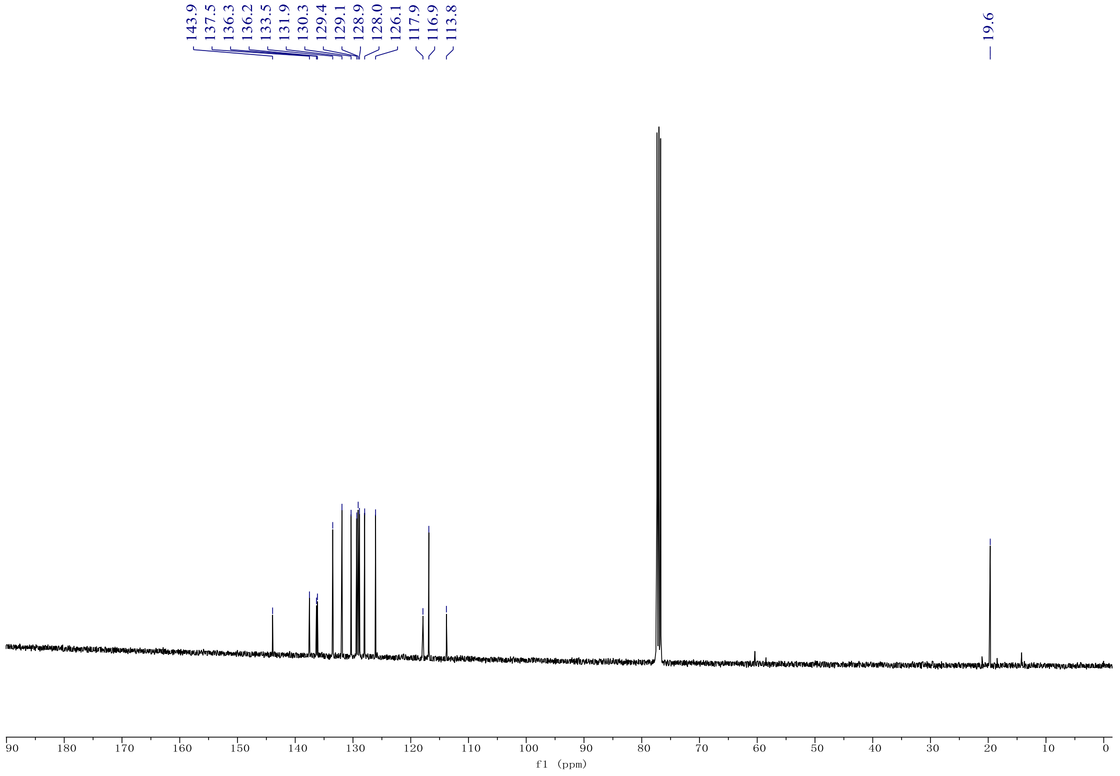

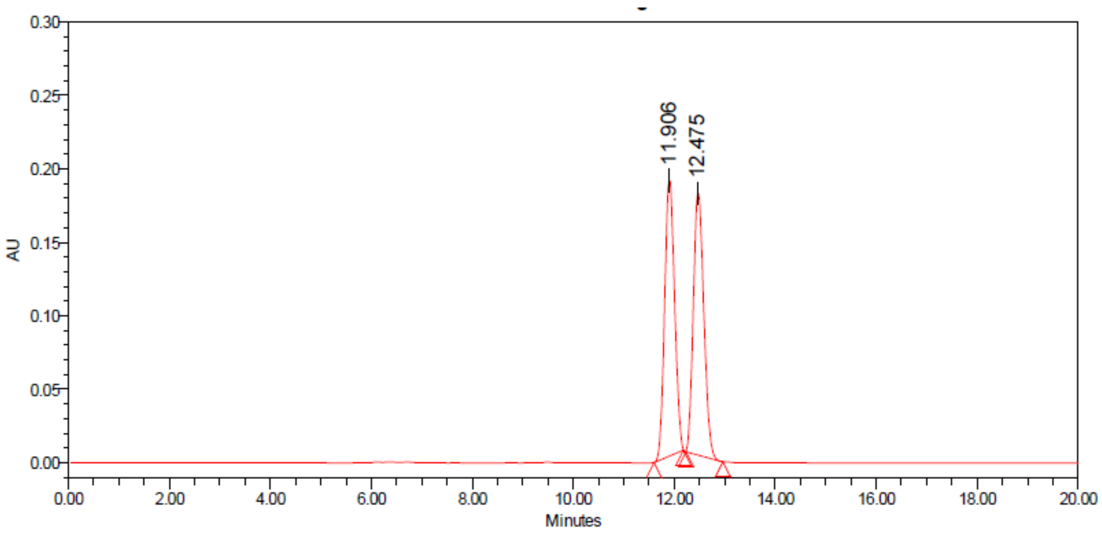

Peak Results

	RT	Height	Area	% Area
1	11.906	188300	2571029	49.90
2	12.475	178783	2581061	50.10

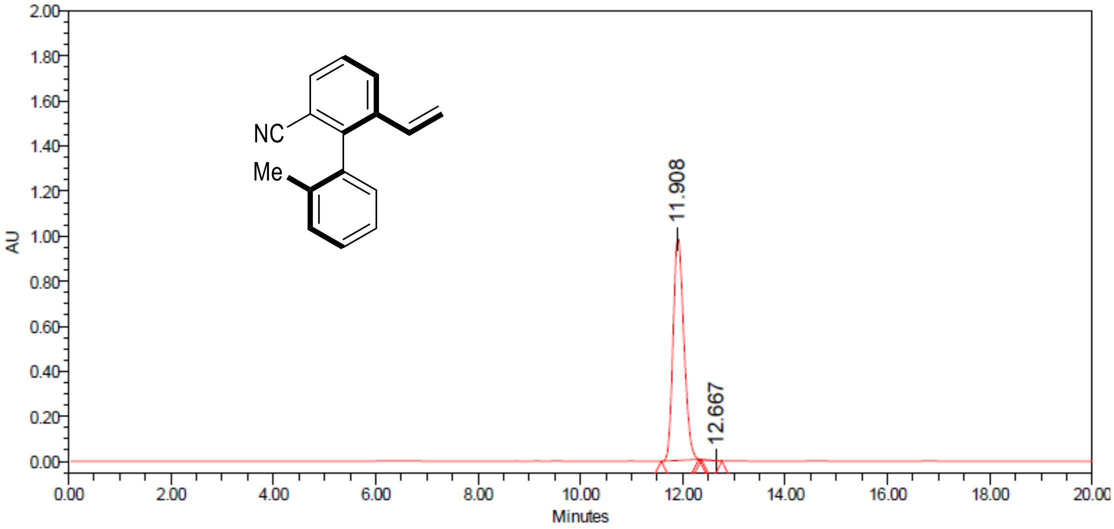

Peak Results

	RT	Height	Area	% Area
1	11.908	987552	14448487	99.96
2	12.667	-445	5684	0.04

(*R*)-2'-methyl-6-vinyl-[1,1'-biphenyl]-2-carbaldehyde (6)

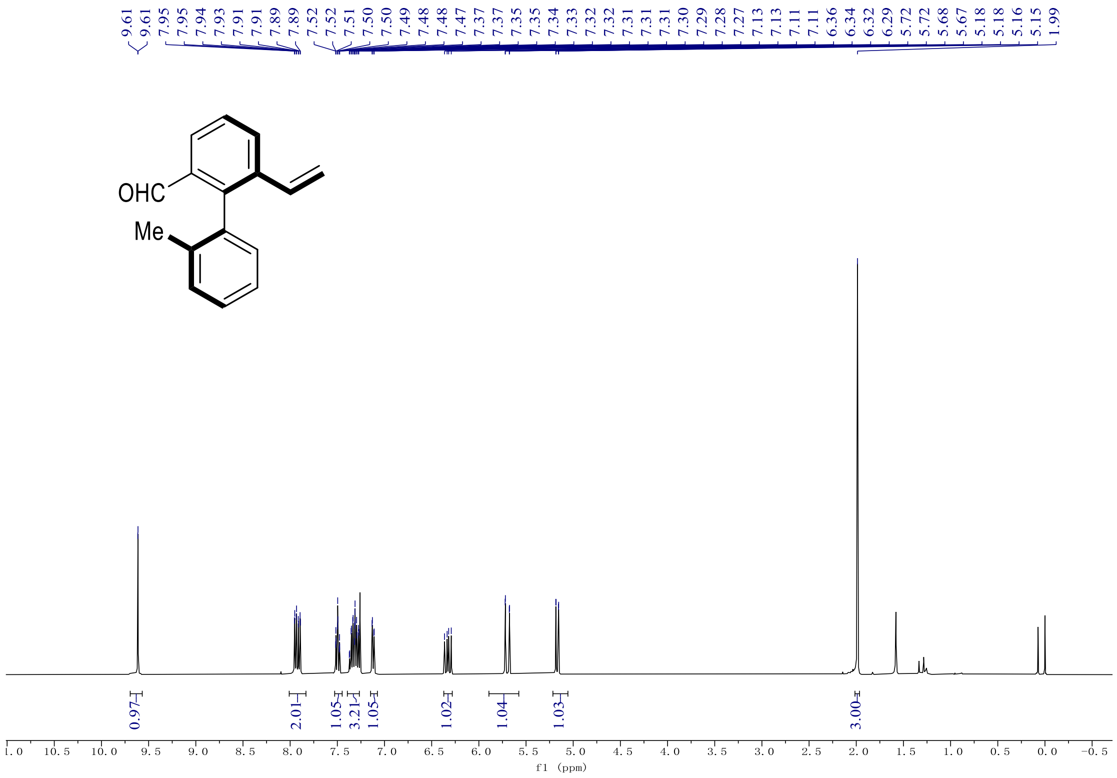

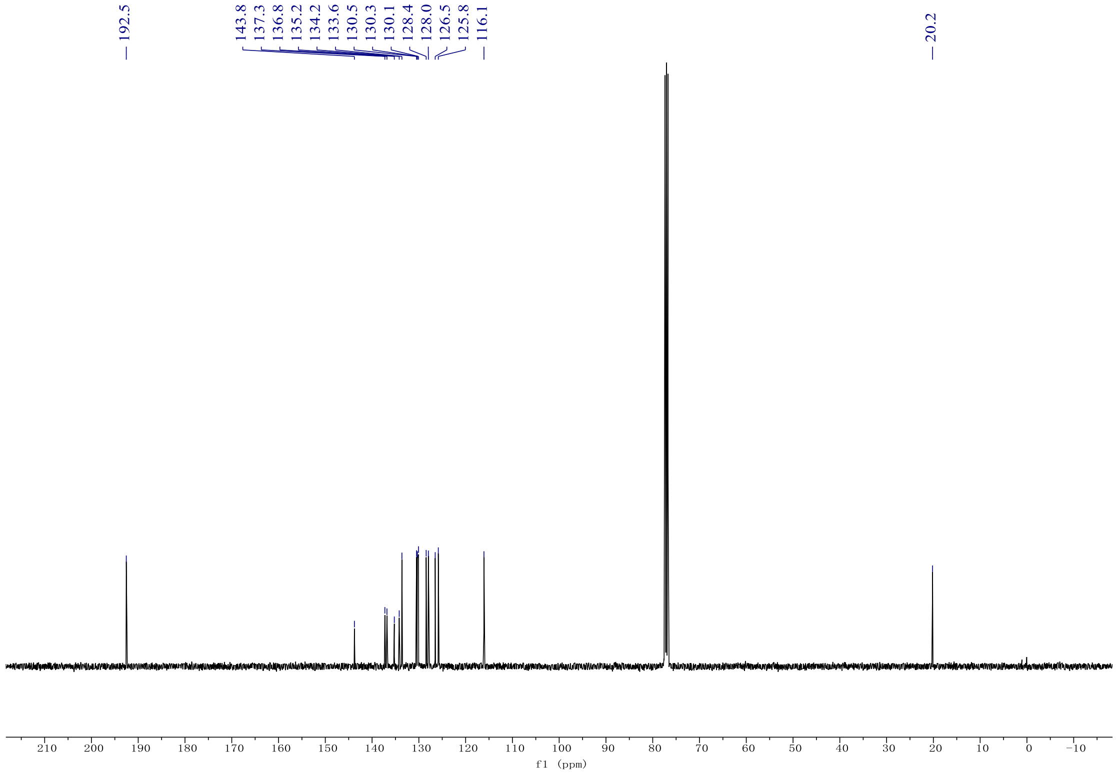

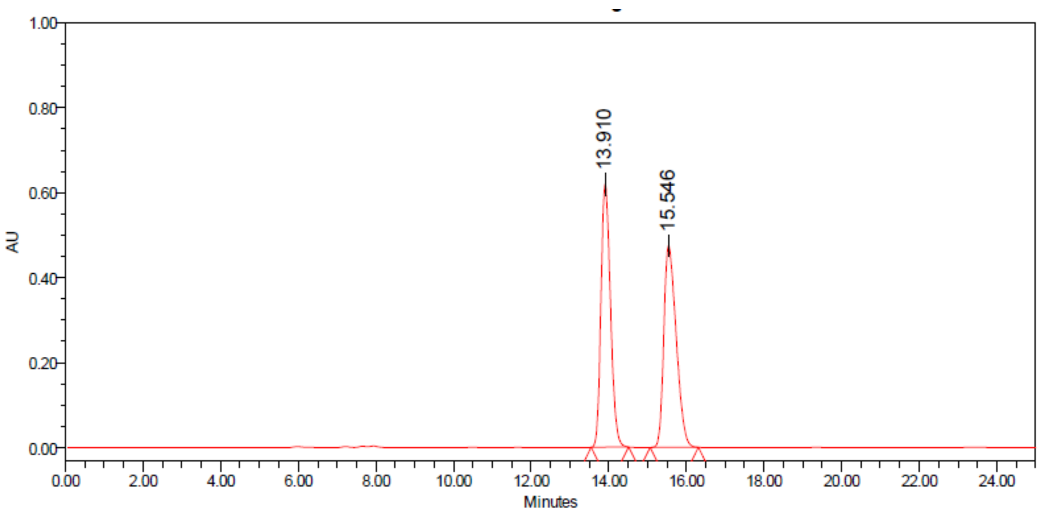

Peak Results

	RT	Height	Area	% Area
1	13.910	620246	10508197	49.90
2	15.546	475011	10548533	50.10

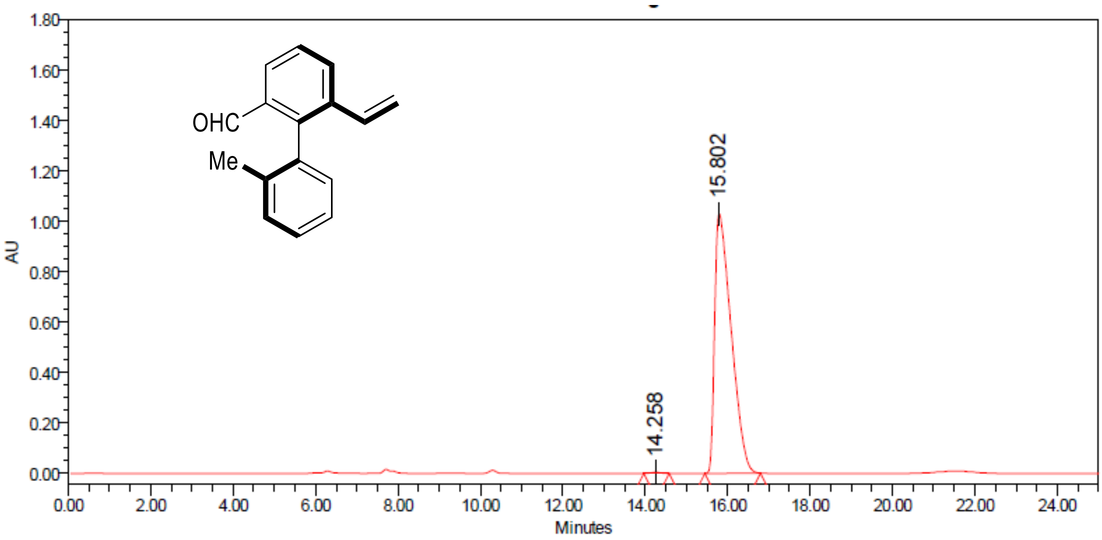

Peak Results

	RT	Height	Area	% Area
1	14.258	4693	74477	0.26
2	15.802	1029887	28758126	99.74

(*S*)-6-(hydroxymethyl)-2'-methyl-[1,1'-biphenyl]-2-carbonitrile (7)

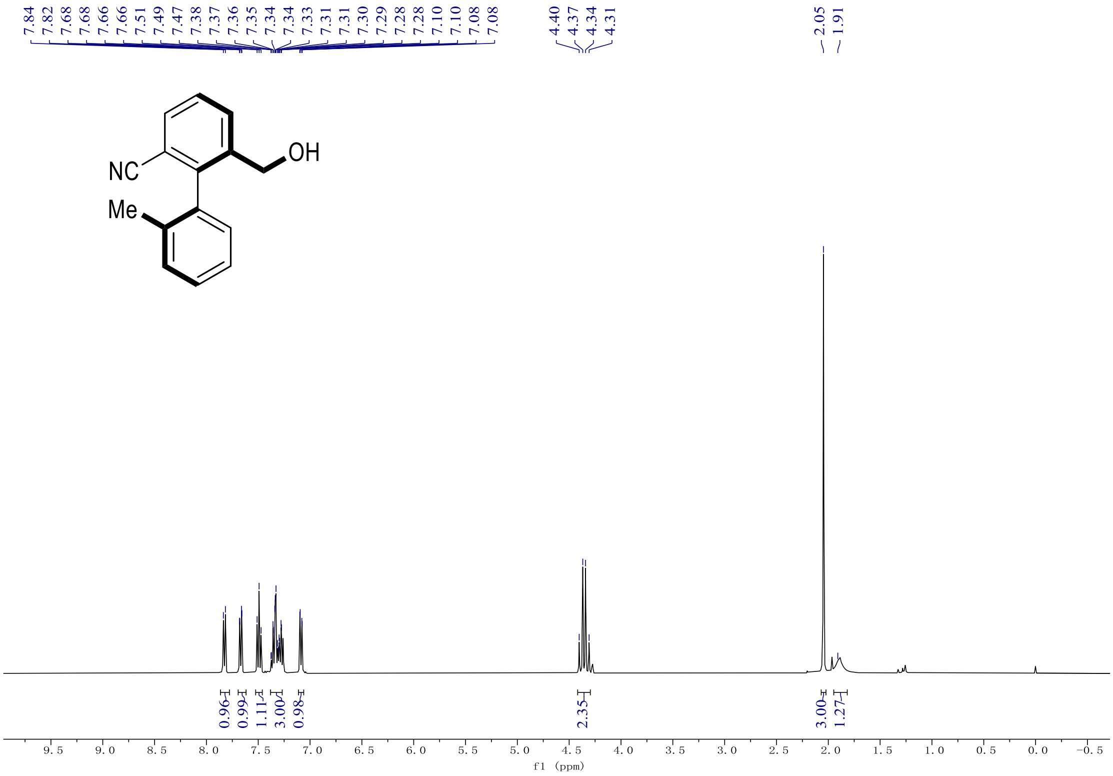

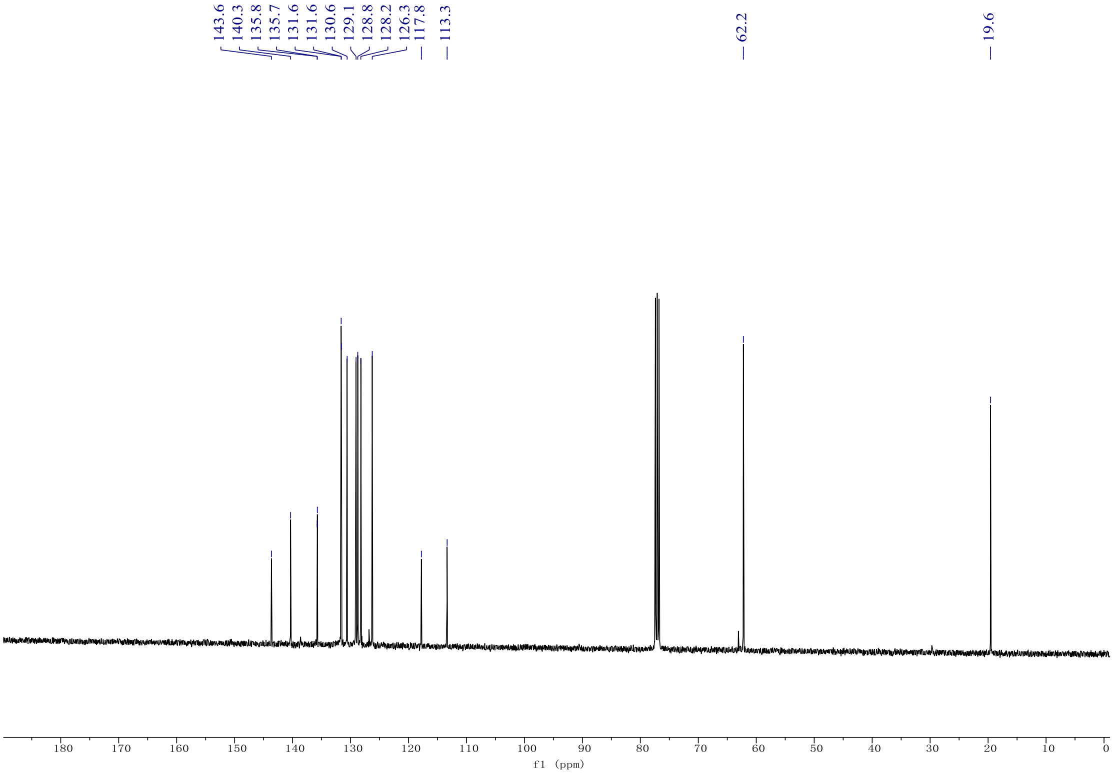

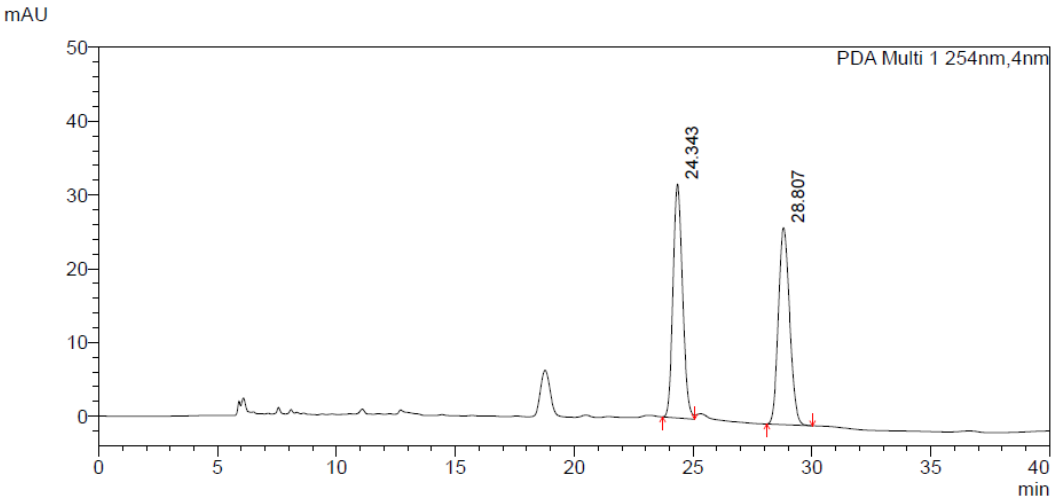

PDA Ch1 254nm

Ret. Time	Height	Area	Area%
24.343	31692	886954	50.102
28.807	26712	883337	49.898
	58404	1770291	100.000

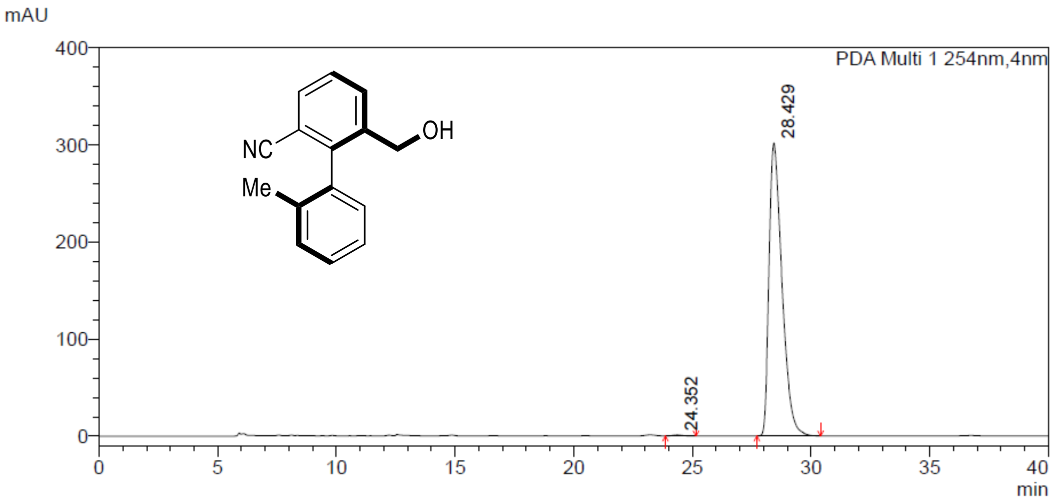

PDA Ch1 254nm

Ret. Time	Height	Area	Area%
24.352	1008	29341	0.255
28.429	301718	11464953	99.745
	302726	11494294	100.000

(*S*)-6-(chloromethyl)-2'-methyl-[1,1'-biphenyl]-2-carbonitrile (8)

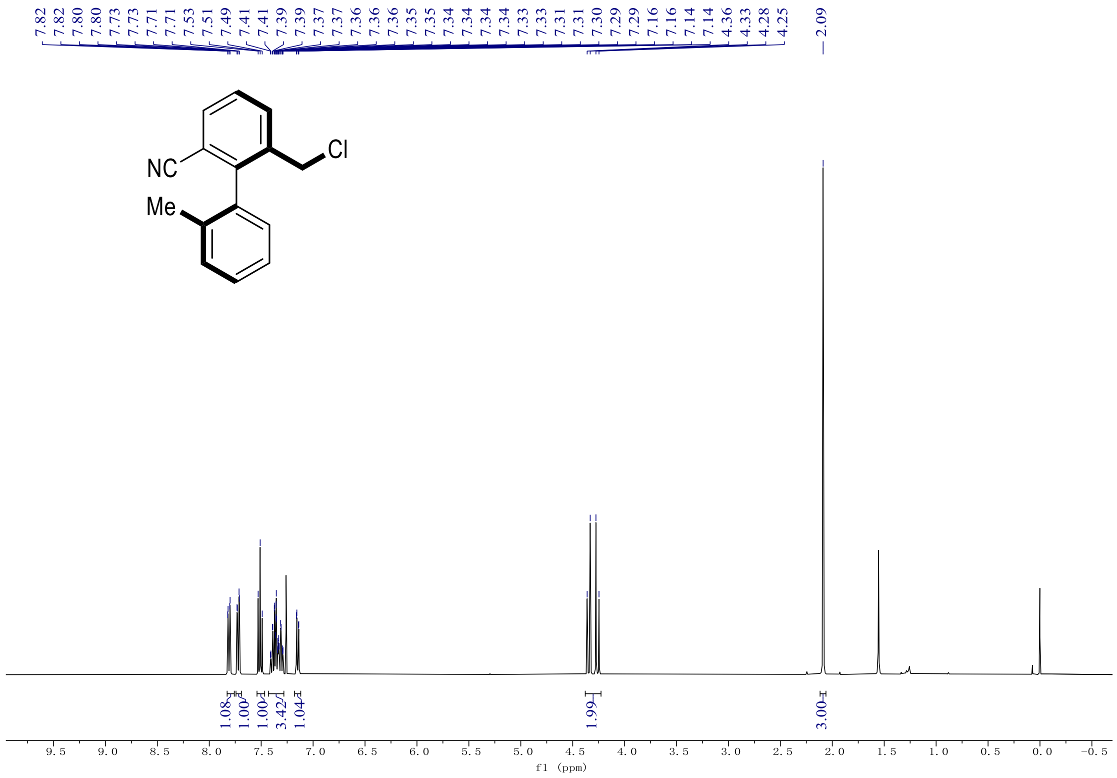

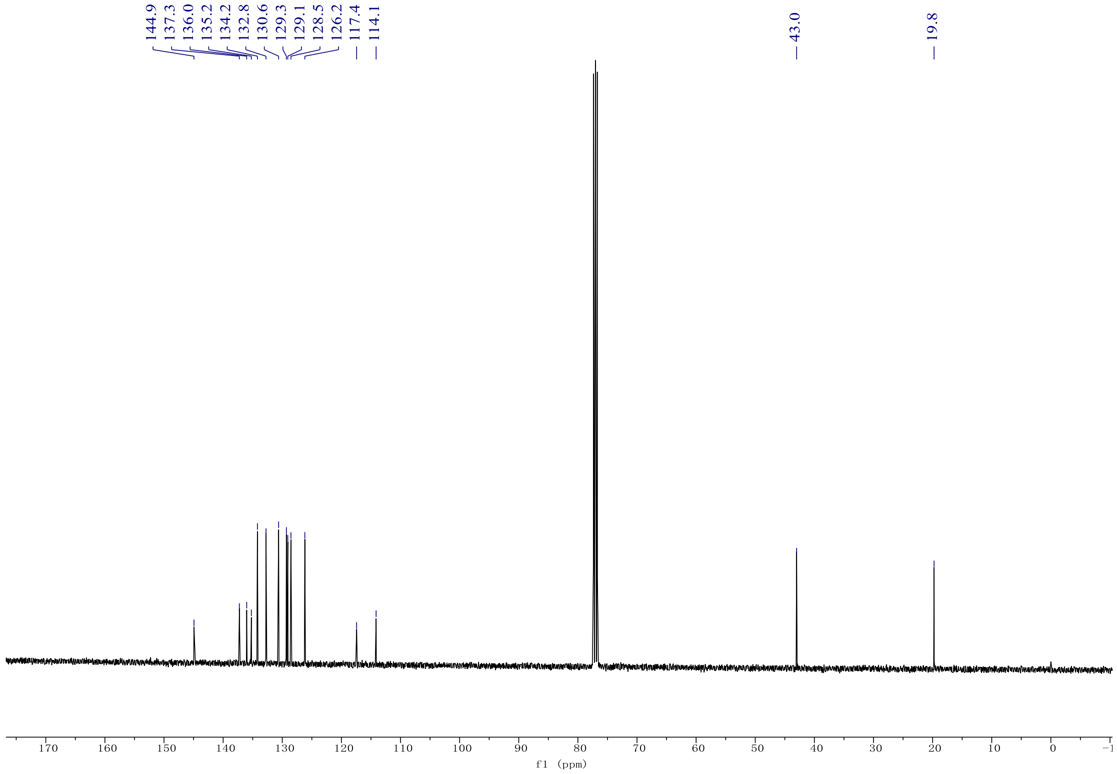

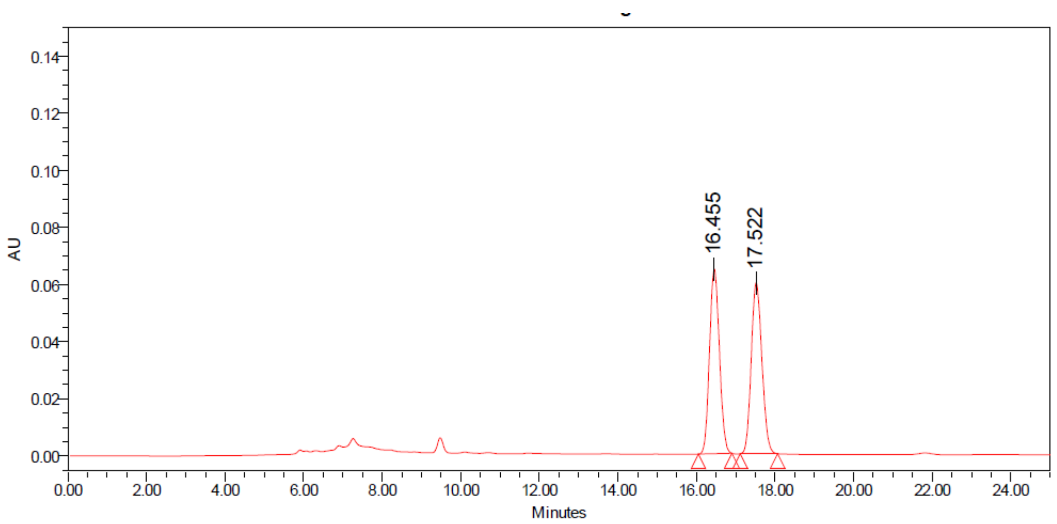

Peak Results

	RT	Height	Area	% Area
1	16.455	64786	1128842	49.88
2	17.522	59841	1134469	50.12

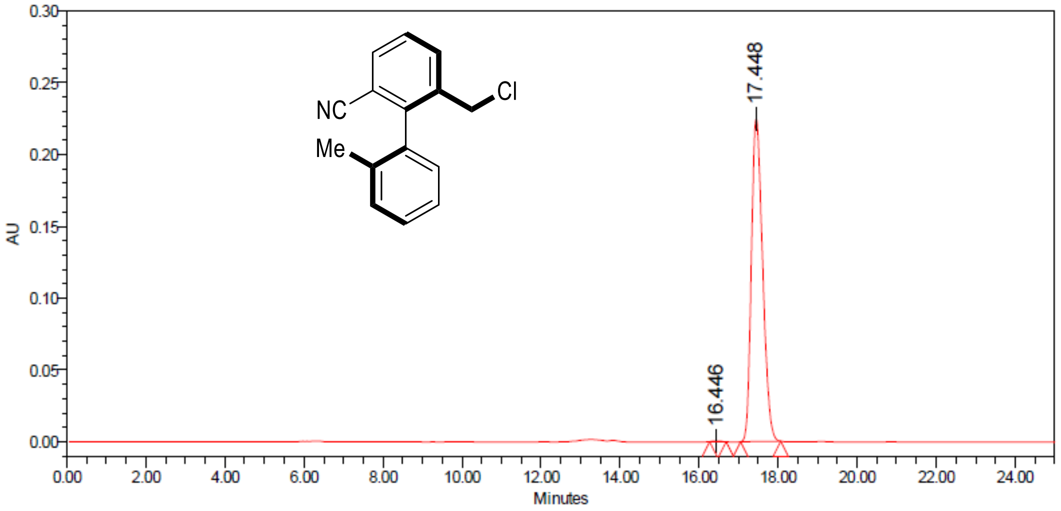

Peak Results

	RT	Height	Area	% Area
1	16.446	530	7247	0.16
2	17.448	224730	4460132	99.84

(*S*)-6-cyano-2'-methyl-[1,1'-biphenyl]-2-carboxylic acid (9)

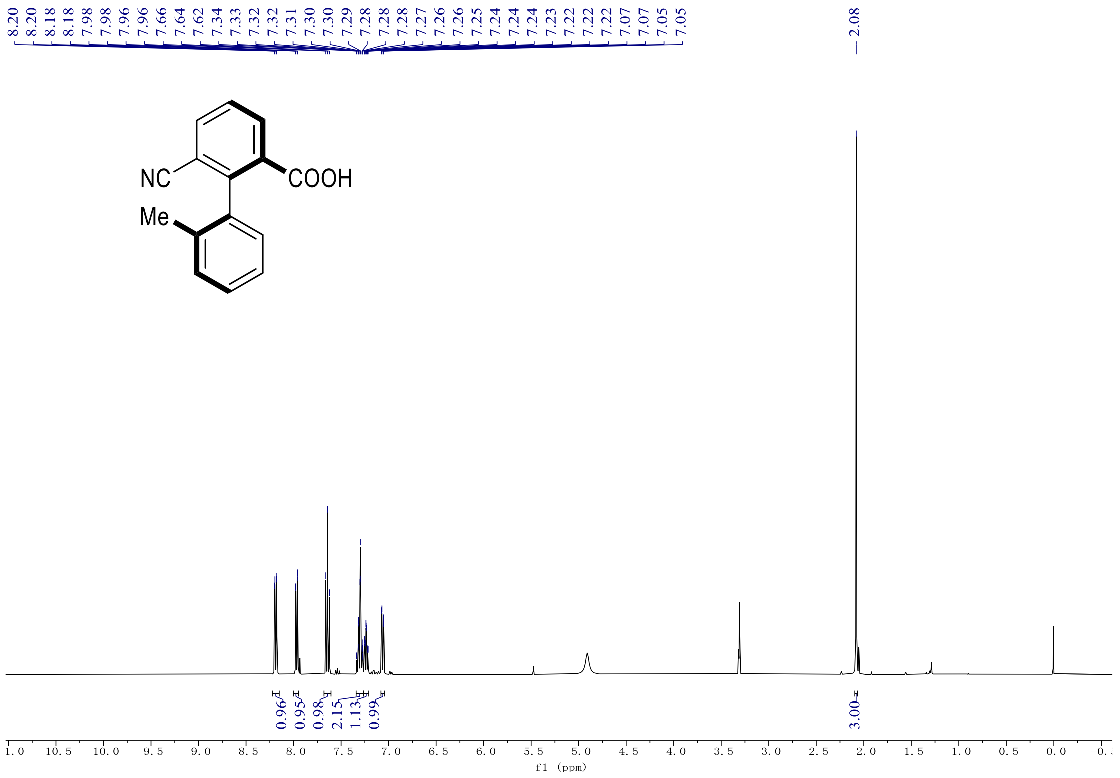

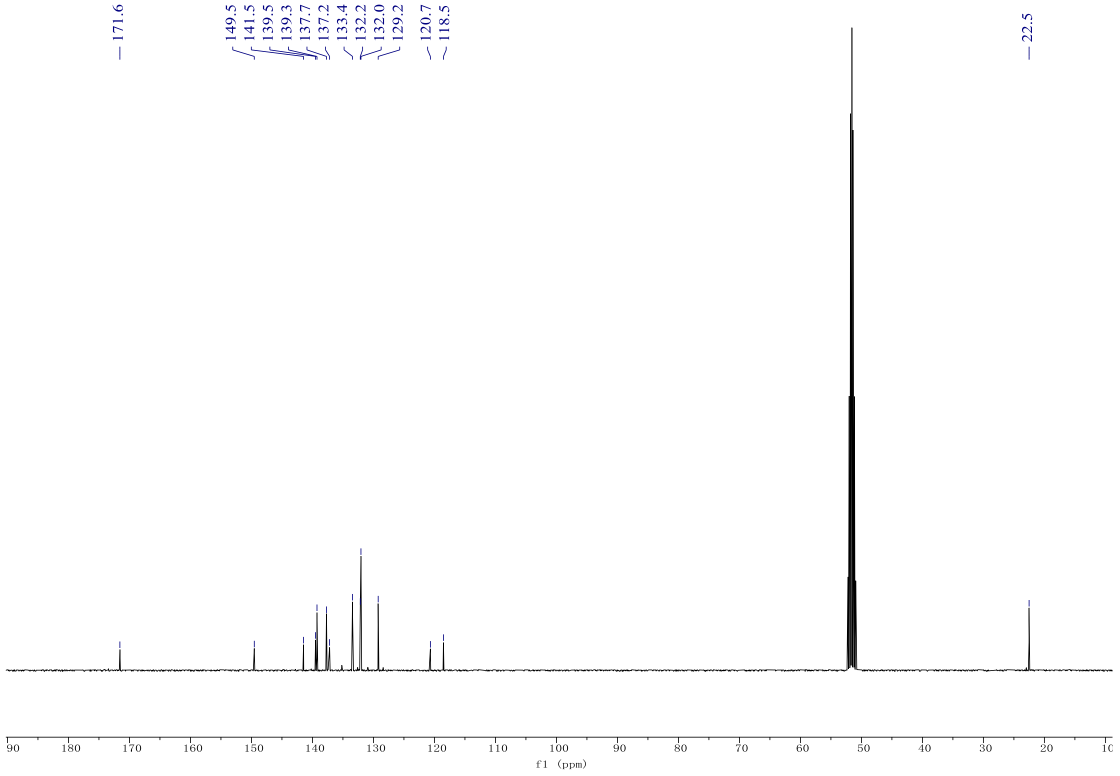

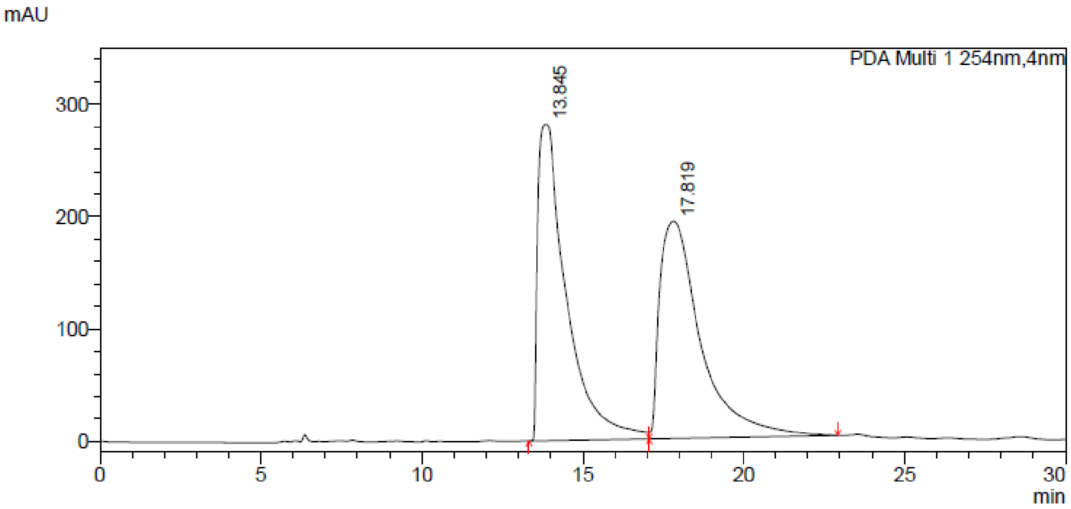

PDA Ch1 254nm

Ret. Time	Height	Area	Area%
13.845	281355	17477716	50.379
17.819	193418	17214417	49.621
	474773	34692133	100.000

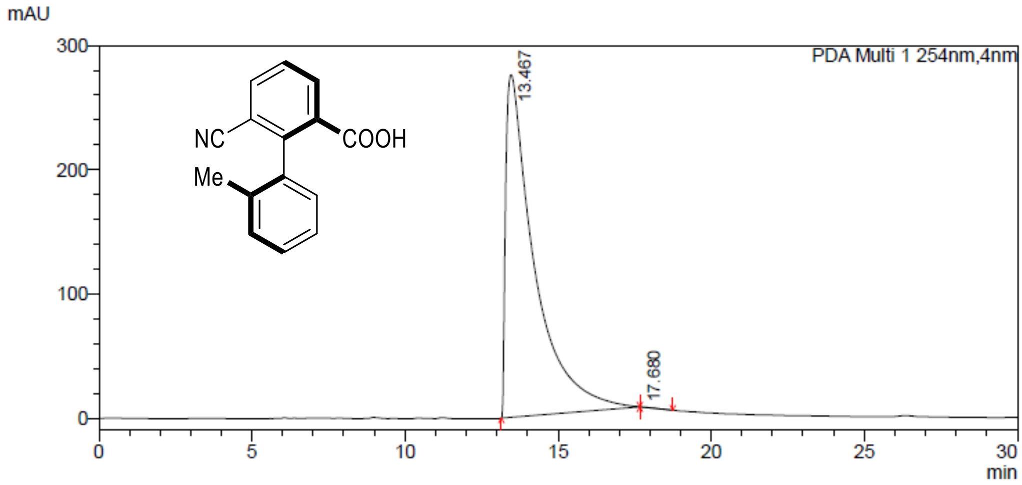

PDA Ch1 254nm

Ret. Time	Height	Area	Area%
13.467	275698	17939441	99.994
17.680	14	1145	0.006
	275712	17940586	100.000
